# Dehydromicrosclerodermin B and Microsclerodermin J: Total Synthesis and Structural Revision

**DOI:** 10.1002/anie.201604764

**Published:** 2016-07-15

**Authors:** Ekaterina Y. Melikhova, Robert D. C. Pullin, Christian Winter, Timothy J. Donohoe

**Affiliations:** ^1^ Department of ChemistryUniversity of Oxford, Chemistry Research LaboratoryMansfield RoadOxfordOX1 3TAUK

**Keywords:** cross-coupling, cyclic peptides, pyrrolidinones, structure elucidation, total synthesis

## Abstract

The total synthesis of dehydromicrosclerodermin B and microsclerodermin J is described. Efficient approaches to the unusual amino acids in the target molecules were developed on the basis of a Negishi coupling (for Trp‐2‐CO_2_H) and Blaise reaction (for Pyrr). An incorrect assignment of the pyrrolidinone stereochemistry of both compounds was confirmed by synthesizing epimers of the proposed structures. The spectroscopic data of these epimers were in complete agreement with those for the naturally derived material.

Microsclerodermins A–M are a family of 13 macrocyclic peptides comprising a 23‐membered ring, which contains six amino acids, three or four of which (depending on the family member) are unique to the microsclerodermins. Microsclerodermins A–I were isolated by Faulkner and co‐workers from the marine sponges *Microscleroderma* and *Theonella* between 1994 and 2000.^1^ In 2012, Li and co‐workers reported the structures of microsclerodermins J and K, isolated from the marine sponge *Microscleroderma herdmani*,^2^ as well as the concomitant isolation of microsclerodermins A and B. Moreover, in a personal communication, Li also reported that microsclerodermin B was readily dehydrated during HPLC purification to dehydromicrosclerodermin B. Recently, intriguing studies on the isolation of microsclerodermins L, M, and D from the terrestrial myxobacteria *Jahnella*, *Chondromyces*, and *Sorangium* were reported by Müller and co‐workers, together with a coherent biosynthesis.^3^ Members of the family show strong antifungal activity against *Candida albicans* and cytotoxicity against the HCT‐116 cell line.

The microsclerodermin macrocycle contains six amino acids: a tryptophan derivative (Trp), sarcosine (Sar), a pyrrolidinone unit (Pyrr), a polyhydroxylated β‐amino acid, γ‐amino‐β‐hydroxybutanoic acid (GABOB), and glycine (Gly; Scheme 1). Throughout the family, the tryptophan, β‐amino acid, and GABOB units possess stereocenters with the same configurations. However, the pyrrolidinone fragment is reported to have different configurations depending on the family member. Microsclerodermins A and B are the only members of the family with the proposed 44*S*,45*S* configuration, and microsclerodermins J and K are the only others with the 45*S* configuration (for this comparison, numbering is taken from microsclerodermin B). In all nine other natural products, both C44 and C45 have been assigned as having the *R* configuration by both NMR and degradation experiments analogous to those performed on microsclerodermin A. Furthermore the *R* configuration of the pyrrolidinone stereocenter of microsclerodermin E was confirmed by total synthesis.^4^

**Scheme 1:**
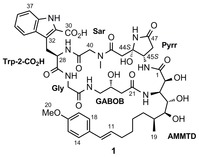
Proposed structure of microsclerodermin B.

The assignments of the C44 and C45 stereocenters of microsclerodermins B, J, and K were made by analogy to microsclerodermin A, and degradation experiments were not performed on these compounds. Another point related to the pyrrolidinone stereochemistry is that an inversion of the configuration at C45 during the biosynthesis of microsclerodermin M has been noted by Müller and co‐workers.^3^ With these observations in mind, we suspected that the stereochemical assignment of the pyrrolidinone unit in microsclerodermins B, J, and K might be incorrect.

The most challenging unit to prepare of any family member is the polyhydroxylated β‐amino acid residue, as this unit possesses four or five contiguous stereocenters and variation in the side chains. Several groups have reported studies towards the synthesis of these β‐amino acids.^5^ For example, we used tethered aminohydroxylation (TA) as the key step in a synthesis of the AMMTD fragment.^6^ To date the only total synthesis of any microsclerodermin was reported by Zhu and Ma for microsclerodermin E, which possesses a simpler side chain than AMMTD (AETD) and an unsaturated *R*‐configured pyrrolidinone core.^4^ The pyrrolidinone aminal moiety at C44 is extremely sensitive to basic or acidic conditions^1^ and as yet has not been incorporated into a total synthesis. Therefore, the original target of our research was microsclerodermin B (proposed structure **1**; Scheme 1).

After preliminary studies, we planned to introduce the sensitive C44 hydroxy group at a late stage of the synthesis of **1** by using a two‐step procedure to hydrolyze the pyrrolidinone double bond: hydroxybromination of the alkene and subsequent debromination (Scheme 2). To perform this process selectively, it was decided that the terminal styrene group should be installed by cross‐metathesis after hydration of the pyrrolidinone unit to avoid competing hydroxybromination of the styrene alkene. The remaining retrosynthesis of **2** involved disconnections at the amide bonds with macrolactamization at the least hindered C25–N24 site.

**Scheme 2:**
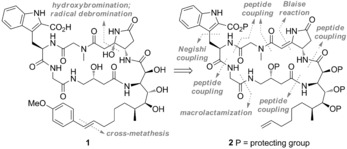
Retrosynthetic analysis of microsclerodermin B (**1**).

Previously we reported the construction of the AMMTD fragment by a Sharpless AD reaction to install the C4–C5 diol and a TA protocol for the C2–C3 amino alcohol.^6^ Substrate **3** was obtained in 16 steps from the *S* Roche ester^6^ and converted into amino alcohol **4**, ready for connection with the GABOB fragment (Scheme 3). The GABOB amino acid was prepared following the route reported by the research groups of Shioiri and Ma, whereby azide **5** was synthesized in four steps from (*R*)‐dimethyl malate.^4^, 5d The methyl ester was then hydrolyzed, followed by the reduction of the azide functionality and subsequent Fmoc protection of the generated amine. Formation of the C21–N3 amide bond was achieved via acid fluoride **7** in high yield (76 %). The primary alcohol of **8** was then oxidized to the carboxylic acid, which was required for coupling of the AMMTD–GABOB dipeptide with the northern hemisphere.

**Scheme 3:**
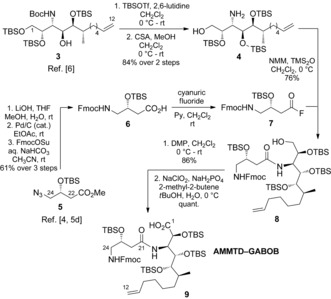
Synthesis of AMMTD–GABOB dipeptide **9**. Boc=*tert*‐butoxycarbonyl, CSA=camphorsulfonic acid, DMP=Dess–Martin periodinane, Fmoc=9‐fluorenylmethoxycarbonyl, NMM=*N*‐methylmorpholine, Py=pyridine, Su=succinimide, TBS=*tert*‐butyldimethylsilyl, Tf=trifluoromethanesulfonyl, TMS=trimethylsilyl.

For construction of the northern hemisphere of **2**, new approaches to target the tryptophan derivative and pyrrolidinone residues were developed. The key step in the synthesis of Trp‐2‐CO_2_R was based on a Negishi coupling between *N*‐Boc‐protected 3‐indole bromide **11** and organozinc reagent **12**, derived from an iodoalanine derivative (Scheme 4).^7^ The effectiveness of our route to Trp‐2‐CO_2_R is twofold:^4^ It does not require harsh reaction conditions and allows potential variation of the indole substitution pattern. The *ee* value of **13** was confirmed by analysis of the corresponding Mosher amides (see the Supporting Information). Trp‐2‐CO_2_R **13** was incorporated into tripeptide **17** by the consecutive removal of the protecting groups and coupling with sarcosine **14** and then glycine **16**, respectively.

**Scheme 4:**
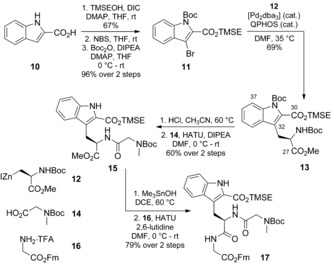
Synthesis of tripeptide **17**. Bn=benzyl, dba=dibenzylideneacetone, DCE=1,2‐dichloroethane, DIC=*N*,*N′*‐diisopropylcarbodiimide, DIPEA=diisopropylethylamine, DMF=*N*,*N*‐dimethylformamide, Fm=9‐fluorenylmethyl, HATU=1‐[bis(dimethylamino)methylene]‐1*H*‐1,2,3‐triazolo[4,5‐*b*]pyridinium 3‐oxide hexafluorophosphate, NBS=*N*‐bromosuccinimide, QPHOS=1,2,3,4,5‐pentaphenyl‐1′‐(di‐*tert*‐butylphosphanyl)ferrocene, TFA=trifluoroacetic acid, TMSE=2‐(trimethylsilyl)ethyl.

For construction of the pyrrolidinone unit, a Blaise reaction was employed as the key step,^8^ which involved the attack of a *tert*‐butyl bromoacetate derived organozinc reagent onto nitrile **19** and subsequent lactamization to give **20** in 68 % yield with 95 % *ee* (determined by HPLC analysis on a chiral stationary phase; see the Supporting Information). This approach provided amino acid **21**, ready for coupling, in 5 steps (Scheme 5), and thus compares favorably to the previously reported approach.^4^

**Scheme 5:**
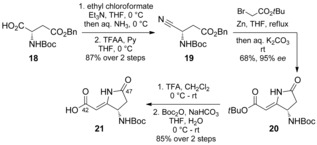
Synthesis of pyrrolidinone **21**. TFAA=trifluoroacetic anhydride.

For completion of the northern hemisphere, the *N*‐Boc group was cleaved from **17** and the amine thus generated coupled with acid **21** (Scheme 6). Coupling of Gly‐Trp‐2‐CO_2_R‐Sar‐Pyrr tetrapeptide **22** with AMMTD–GABOB dipeptide **9** furnished a 1:1 mixture of linear hexapeptides **23** and **24** in 63 % yield, whereby the spontaneous loss of one of the TBS groups had occurred (**23** and **24** were readily separable by column chromatography). Liberation of the C‐ and N‐termini for macrolactamization was achieved in unison with piperidine and was followed by cyclization in the presence of the phosphonium reagent PyAOP.

**Scheme 6:**
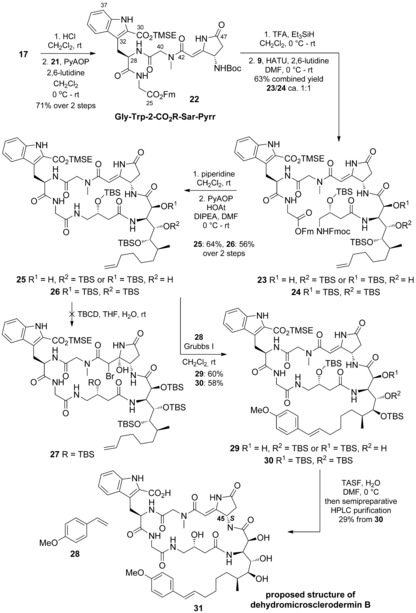
Synthesis of **31**, the originally proposed structure of dehydromicrosclerodermin B. PyAOP=(7‐azabenzotriazol‐1‐yloxy)tripyrrolidinophosphonium hexafluorophosphate, HOAt=1‐hydroxy‐7‐azabenzotriazole, TBCD=2,4,4,6‐tetrabromo‐2,5‐cyclohexadienone, TASF=tris(dimethylamino)sulfonium difluorotrimethylsilicate.

According to our retrosynthetic plan, the next step was hydroxybromination of the pyrrolidinone double bond by a previously developed procedure. Unfortunately, when **26** was subjected to bromination (TBCD) in THF/H_2_O, the desired bromohydrin **27** was not observed. Although the starting material was consumed, only undefined and inseparable side products were obtained. After several unsuccessful attempts, the bromohydrin‐based end game had to be abandoned. However, because of the availability of advanced intermediates, our attention switched to the completion of a synthesis of dehydromicrosclerodermin B (**31**).

To prepare **31**, we introduced the styrene moiety through cross‐metathesis of **25**/**26** with 4‐methoxystyrene, followed by global deprotection. For this cross‐metathesis reaction, a stoichiometric amount of the Grubbs I catalyst and a large excess of the styrene partner were required, probably owing to inhibition of the Ru catalyst as a result of metal ligation to the amide groups. Nonetheless, cross‐metathesis products **29** and **30** were obtained in good yields (60 and 58 %). The global deprotection was then performed with TASF. A sample of dehydromicrosclerodermin B was kindly provided by Li together with the original ^13^C NMR data (see the Supporting Information);^2^ this data allowed us to compare the synthesized structure **31** with the naturally derived compound.

Analysis of the NMR data for **31** provided unexpected results, as two rotameric forms were observed, in a ratio of approximately 3:1, although the NMR data for natural dehydromicrosclerodermin B do not show the presence of rotamers.^9^ When synthetic **31** was compared to dehydromicrosclerodermin B prepared by Li and co‐workers by ^13^C NMR spectroscopy,^10^ a large discrepancy in the data was observed for two regions of the molecule: the pyrrolidinone core and the GABOB unit (Δ*δ*_C_ up to 2.3 ppm; Figure 1). Furthermore, HPLC studies on synthetic **31** and the authentic sample (and a 1:1 mixture) clearly showed that these were two different compounds (see the Supporting Information).

**Figure 1:**
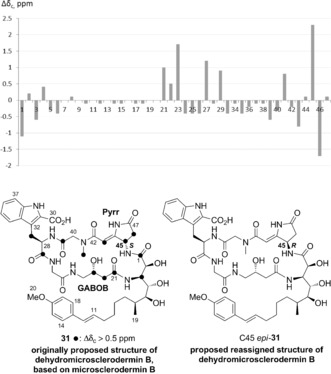
Comparison of the ^13^C NMR spectra of synthetic **31** and naturally derived dehydromicrosclerodermin B (note: C30, C31, and C32 were not identified in the spectrum of natural dehydromicrosclerodermin B; C31 and C32 were not identified in the spectrum of synthetic **31**) and reassigned structure of dehydromicrosclerodermin B, C45 *epi*‐**31**.

Faulkner and co‐workers previously reported NOE correlations on related dehydromicrosclerodermin A.1a Interestingly, weak NOE correlations between the Pyrr and GABOB units were observed, thus suggesting that they are close in space. Consequently, we thought that stereochemical misassignment of either C45 or C23 might result in the observed discrepancy between the ^13^C NMR spectra. Given the lack of clarity regarding the configuration at C44–C45 in the microsclerodermins, we came to the conclusion that the C45 stereocenter may have been assigned incorrectly.1a To validate this idea, we pursued the synthesis of the C45 *R* epimer, C45 *epi*‐**31**.

Therefore, the 45*R* tetrapeptide C45 *epi*‐**22 a** was synthesized by using the protocol described above for the 45*S*‐configured analogue. As the route involving late‐stage selective hydroxybromination of the pyrrolidinone double bond had been discarded, the styrene moiety of the AMMTD unit was introduced earlier in the synthesis to avoid the problematic late‐stage cross‐metathesis (Scheme 7). By the use of the same reagents and conditions as for the AMMTD substrate containing the terminal alkene,^6^ cross‐metathesis product **33** was advanced to **34** and further to C45 *epi*‐**31** (see the Supporting Information for details).

**Scheme 7:**
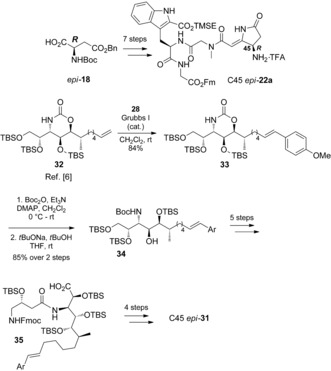
Synthesis of C45 *epi*‐**31**.

The NMR data for synthetic C45 *epi*‐**31** was promising; the presence of rotamers was not observed. Comparison of the ^13^C NMR data of synthetic C45 *epi*‐**31** and authentic dehydromicrosclerodermin B showed all signals in good agreement (Figure 2). For most of the signals, the difference does not exceed 0.1 ppm, including those for the pyrrolidinone and GABOB regions. Some signals, such as those for C28, C36, C38, C39, C40, and C41, differ by 0.2–0.3 ppm.

**Figure 2:**
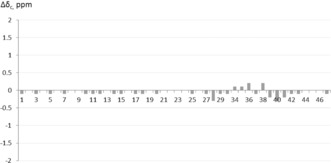
Comparison of the ^13^C NMR spectra of synthetic C45 *epi*‐**31** and natural dehydromicrosclerodermin B.

Further evidence that synthetic C45 *epi*‐**31** matched the naturally derived compound was secured by ^1^H NMR and HPLC analysis of a 1:1 mixture of the two samples; complete overlap of the peaks was observed (see the Supporting Information). On the basis of this analysis, it was concluded that structure **31** proposed for dehydromicrosclerodermin B, and, by extension, structure **1** proposed for microsclerodermin B are incorrect, and that both compounds have the *R*‐configured C45 stereocenter.

The incorrect assignment of microsclerodermin B mandates reassignment of the C44 stereocenter of structurally close microsclerodermin J (proposed structure **36**; Scheme 8);^11^ the configuration of this compound was originally assigned by Li and co‐workers, in part by comparison with the incorrect structure of microsclerodermin B.2b We thus suspected that the correct structure of microsclerodermin J was actually C44 *epi*‐**36**.

**Scheme 8:**
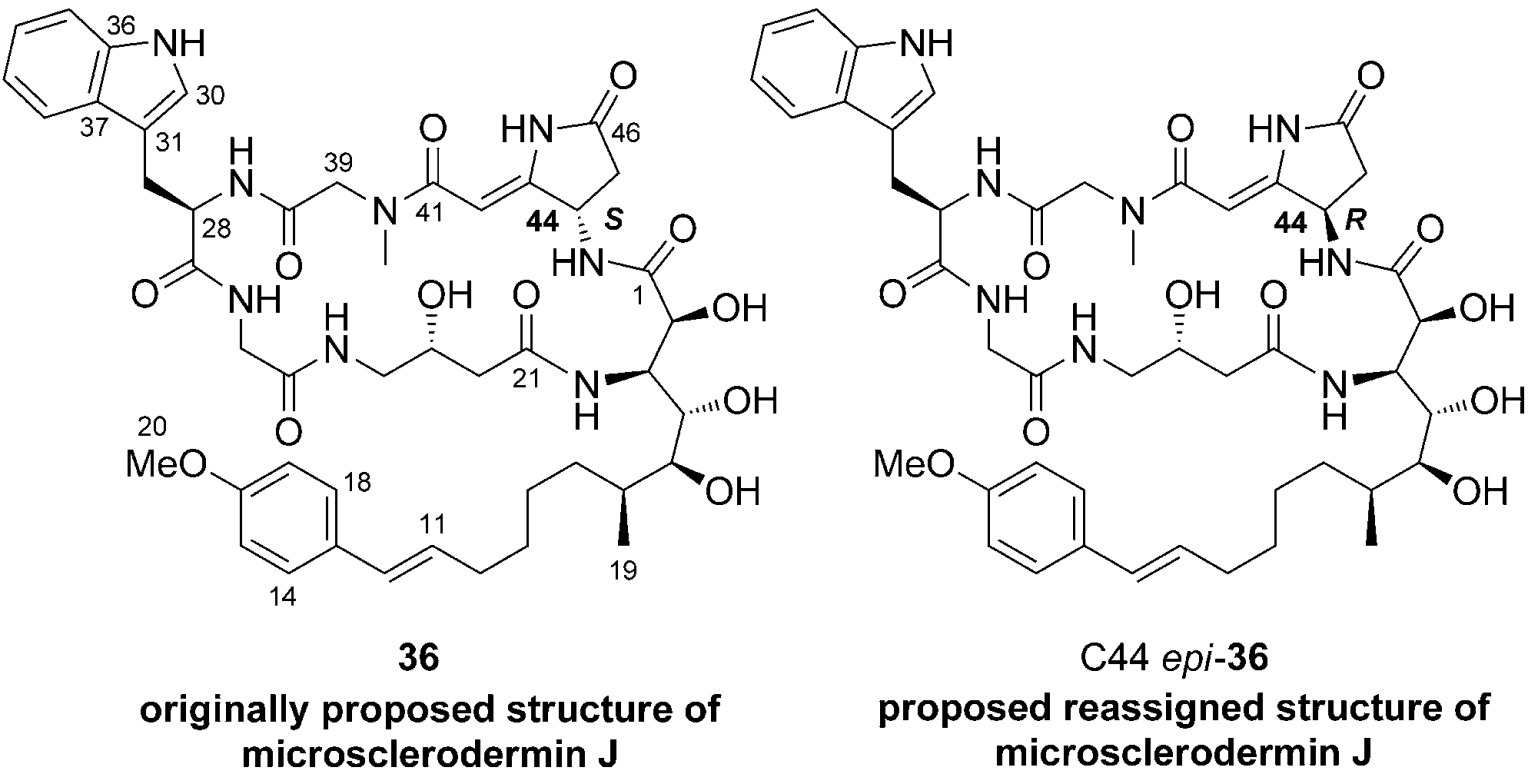
Originally proposed structure of microsclerodermin J, **36**, and proposed reassigned structure of microsclerodermin J, C44 *epi*‐**36**.

The synthesis of C44 *epi*‐**36** was similar to that used for dehydromicrosclerodermin B. Tryptophan derivative **37** was incorporated into the synthesis of Gly‐Trp‐Sar tripeptide **39** by peptide‐bond formation and protecting‐group removal (Scheme 9). The synthesis of C44 *epi*‐**36** was completed by using the protocol described above for C45 *epi*‐**31** (see the Supporting Information for details).

**Scheme 9:**
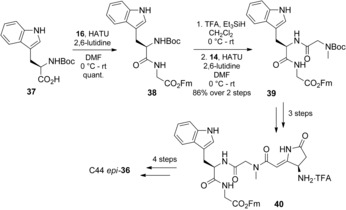
Synthesis of the reassigned structure of microsclerodermin J, C44 epi‐**36**.

No rotameric signals were evident in the NMR spectra of C44 *epi*‐**36**. Direct comparison of the ^13^C NMR data of C44 *epi*‐**36** to that of natural microsclerodermin J was aided by a copy of the ^13^C NMR spectrum provided by Li. This spectrum was critical to our analysis, as there were minor inaccuracies in the data listings given in the original isolation paper (see the Supporting Information for a copy of the ^13^C NMR spectrum and a list of inaccuracies in the published ^13^C NMR data for microsclerodermin J).2a This comparison showed a complete match between the ^13^C NMR spectra of synthetic C44 *epi*‐**36** and the natural material, with the differences in chemical shift not exceeding 0.1 ppm. From these data, it was confirmed that microsclerodermin J also has the *R* configuration at the C44 stereocenter.

To conclude, the first total synthesis of the proposed structure **31** of dehydromicrosclerodermin B was accomplished. The originally proposed C45 configuration for the parent compound **1** was reassigned from 45*S* to 45*R*, and this configuration was confirmed by synthesizing C45 *epi*‐**31**, whose data were in complete agreement with those for naturally derived dehydromicrosclerodermin B. We also reassigned the C44 configuration of an analogous member of the family—microsclerodermin J—as 44*R* by completing the first total synthesis of C44 *epi*‐**36**. Owing to our unsuccessful efforts to construct the sensitive pyrrolidinone aminal moiety through a hydroxybromination sequence, alternative hydration strategies will be pursued in the future for the total synthesis of microsclerodermin B.

## Supporting information

As a service to our authors and readers, this journal provides supporting information supplied by the authors. Such materials are peer reviewed and may be re‐organized for online delivery, but are not copy‐edited or typeset. Technical support issues arising from supporting information (other than missing files) should be addressed to the authors.

Supplementary
