# Supplementary material for: Dehydromicrosclerodermin B and Microsclerodermin J: Total Synthesis and Structural Revision
Source: Angew Chem Int Ed Engl. 2016 Jul 15;55(33):9753–7. doi: 10.1002/anie.201604764 (PMC5132153; doi:10.1002/anie.201604764)
Supplement: Supplementary file 1 — Supplementary [file ANIE-55-9753-s001.pdf]

Angewandte Chemie
Eine Zeitschrift der Gesellschaft Deutscher Chemiker

Supporting Information

Dehydromicrosclerodermin B and Microsclerodermin J: Total Synthesis and Structural Revision

*Ekaterina Y. Melikhova, Robert D. C. Pullin, Christian Winter, and Timothy J. Donohoe**

anie_201604764_sm_miscellaneous_information.pdf

Supporting Information

Table of contents

1. Experimental techniques 2
2. Experimental procedures 4
3. References 71
4. NMR and HPLC data of novel compounds 72

1. Experimental techniques.

General: All non-aqueous reactions were carried out under an argon atmosphere in flame-dried glassware unless otherwise stated. All reactions were followed by analytical thin layer chromatography (TLC) and procedure times represent reaction completion as judged accordingly. Reaction temperatures (unless otherwise stated) represent bath temperatures.

Solvents: Anhydrous DMF and 1,2-dichloroethane were used as commercially supplied. CH_2Cl_2, Et_2O, MeCN, MeOH, THF and toluene were purified by filtration through two activated alumina purification columns. In cases where mixtures of solvents were used, the ratios refer to the component volumes. Petrol refers to petroleum ether in the boiling range 30-60 °C.

Reagents: All reagents were used as supplied commercially unless otherwise stated.

Chromatography: Flash column chromatography was carried out using silica gel 40-63 µm unless otherwise stated. Analytical thin layer chromatography (TLC) was performed using aluminium backed pre-coated silica gel 60 F_{254} plates and visualised under UV radiation at 254 nm and staining with phosphomolybdic acid in ethanol, potassium permanganate in water or vanillin in ethanol.

Optical Rotation: Specific rotations (α') were recorded on a Perkin Elmer 341 Polarimeter, with a cell pathlength (l) of 1.0 dm, at the stated temperature (°C) and concentration (*c*) (measured in units of g/100 mL); specific rotations were converted to optical rotations, $[\alpha]_D$, via the equation: $[\alpha]_D = (100\cdot\alpha')/(l\cdot c)$.

Melting Points: Melting points were obtained using a Leica Galen III heated-stage microscope and are uncorrected.

Mass Spectrometry: Mass spectrometry was carried out using CI^+ (NH_3), ES^+ or EI^+ and recorded using a Brucker MicroTof or Fisons Platform II spectrometer. Only molecular ions and fragments from molecular ions are reported with relative intensities of peaks quoted as a percentage value. Accurate mass (HRMS) were recorded on a Brucker MicroTof spectrometer under conditions of electrospray ionisation (ESI).

NMR Spectroscopy: ^1H NMR spectra were recorded at 200, 400 and 500 MHz in $CDCl_3$, CD_2Cl_2, D_6-DMSO or D_4-MeOD, on a Bruker Advance 200, 250, 300, 400 or 500 spectrometer respectively, at the stated temperature, and reported as follows; chemical shift (δ) (reported to the nearest 0.01 ppm), (number of protons, multiplicity (singlet (s), doublet (d), triplet (t), quartet (q), multiplet (m) and/or broad (br.)), coupling constant, *J*

(reported in Hertz (Hz) to the nearest 0.1 Hz), assignment). Residual protic solvent was used as the internal reference: $CDCl_3$ (δ_H = 7.26 ppm), D_6-DMSO (δ_H = 2.50 ppm), D_4-MeOD (δ_H = 3.31 ppm). ^{13}C NMR spectra were recorded at 75, 100 and 126 MHz, with complete proton decoupling, in $CDCl_3$, D_6-DMSO or D_4-MeOD, on a Bruker Advance 400 or 500 spectrometer respectively with the chemical shift, δ, reported to the nearest 0.1 ppm. The internal reference used: $CDCl_3$ (δ_C = 77.0 ppm), D_6-DMSO (δ_C = 39.5 ppm), D_4-MeOD (δ_C = 49.1 ppm). ^{19}F NMR spectra were recorded at 376 MHz on a Bruker Advance 400 spectrometer with the chemical shift reported to the nearest 0.1 ppm. All chemical shifts are quoted in parts per million relative to tetramethylsilane (δ_H = 0.00 ppm). Assignments were based upon COSY, DEPT, HSQC and HMBC experiments, nOe enhancement experiments where appropriate, and by comparison to related compounds.

IR Spectroscopy: Infra-red spectra were recorded on a Bruker Tensor 27 FT-IR spectrometer. Absorption maxima (ν_{max}) are reported in wavenumbers (cm^{-1}) and broad (br) signals are specified.

2. Experimental procedures.

Synthesis of AMMTD-GABOB dipeptide 9.

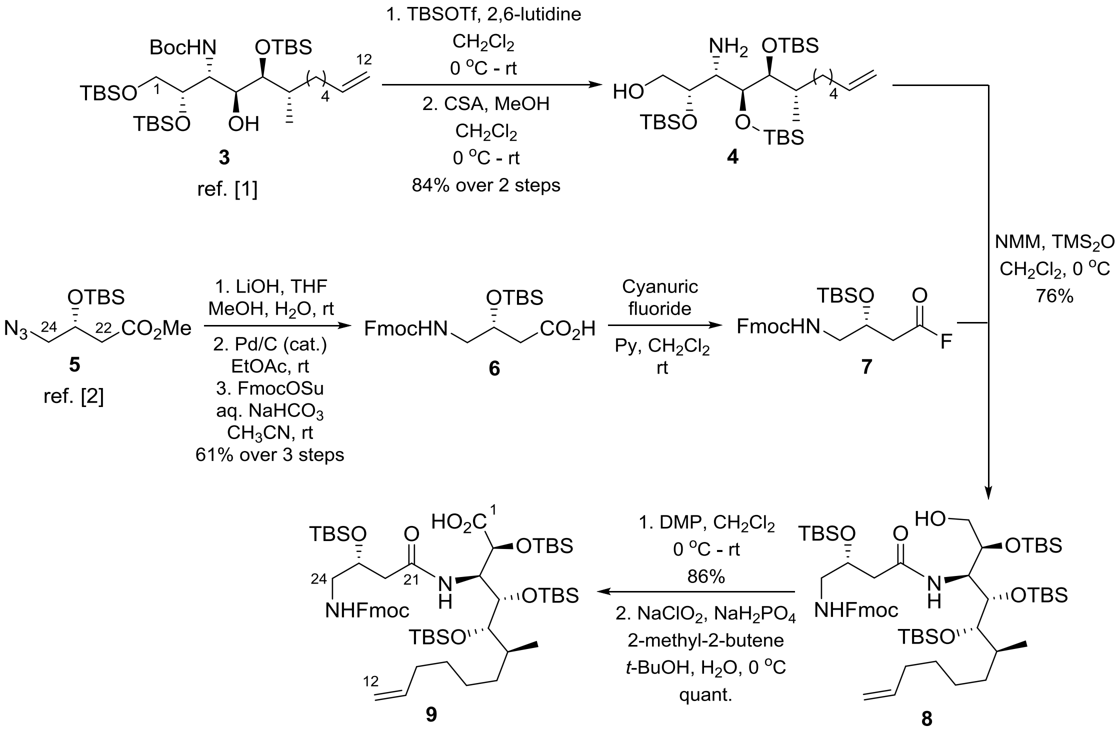

(2*S*,3*S*,4*S*,5*S*,6*S*)-3-Amino-2,4,5-tris((*tert*-butyldimethylsilyl)oxy)-6-methyldodec-11-en-1-ol (4)

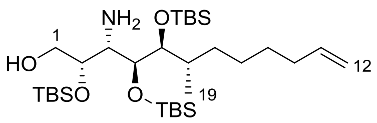

tert-Butyldimethylsilyl trifluoromethanesulfonate (3.20 mL, 14.1 mmol) was added dropwise to a solution of **3**[1] (994 mg, 1.41 mmol) and 2,6-lutidine (3.20 mL, 28.2 mmol) in CH_2Cl_2 (15.0 mL) at 0 °C. The mixture was warmed to room temperature and stirred for 16 h before being quenched with saturated aqueous NH_4Cl. The aqueous layer was separated and extracted with CH_2Cl_2. The organic layer was then washed with 1M HCl (x 2), dried (Na_2SO_4), filtered and concentrated *in vacuo*. The crude mixture was dissolved in CH_2Cl_2 (7.5 mL) and MeOH (7.5 mL) and (±)-camphor 10-sulfonic acid (657 mg, 2.80 mmol) was added in one portion at 0 °C. The mixture was then warmed to room temperature and stirred

for 48 h. Saturated aqueous $NaHCO_3$ was then added and the aqueous layer was then separated and extracted with CH_2Cl_2, dried (Na_2SO_4), filtered and concentrated *in vacuo*. Purification by flash column chromatography (5-10% EtOAc/petrol) afforded aminoalcohol **4** (686 mg, 84% over 2 steps) as a colorless oil; R_f: 0.30 (10% EtOAc/hexane); $[\alpha]^{20}_{D}$ −38.8 (*c* 0.08, $CHCl_3$); ν_{max}/cm^{-1} (neat) 2955, 2929, 2858, 1472, 1253, 1066, 909, 834, 775, 669; δ_H (400 MHz, $CDCl_3$) 5.82 (1H, m, 11-*H*), 5.03-4.93 (2H, m, 12-*H*), 4.04 (1H, m, 2-*H*), 3.89 (1H, dd, *J* 11.7 and 2.7, 1-*H*H), 3.80 (1H, dd, *J* 11.7 and 2.0, 1-H*H*), 3.64 (1H, dd, *J* 9.5 and 2.9, 4-*H*), 3.53 (1H, dd, *J* 7.6 and 2.9, 5-*H*), 3.10 (1H, d, *J* 9.5, 3-*H*), 2.09-2.04 (2H, m, 10-*H*), 1.86-1.58 (4H, m, 6-*H*, 7-*H*H and NH_2), 1.46-1.33 (3H, m, 9-*H* and 8-*H*H), 1.21 (1H, m, 8-H*H*), 1.02 (1H, m, 7-H*H*), 0.98 (3H, d, *J* 6.6, 19-*H*), 0.93-0.87 (28H, m, 3 x SiC($CH_3)_3$ and 7-H*H*), 0.21, 0.13, 0.11, 0.11, 0.10 and 0.08 (6 x 3H, s, 6 x SiCH_3); δ_C (100 MHz, $CDCl_3$) 139.0 (11-*C*), 114.3 (12-*C*), 79.6 (5-*C*), 74.5 (4-*C*), 69.7 (2-*C*), 69.0 (1-*C*), 58.5 (3-*C*), 34.8 (6-*C*), 33.8 (10-*C*), 33.1 (7-*C*), 29.2 (9-*C*), 26.4 (8-*C*), 26.0 (SiC(*C*$H_3)_3$), 26.0 (SiC(*C*$H_3)_3$), 25.9 (SiC(*C*$H_3)_3$), 18.4 (Si*C*($CH_3)_3$), 18.1 (Si*C*($CH_3)_3$), 18.0 (Si*C*($CH_3)_3$), 17.4 (19-*C*), −2.6 (Si*C*H_3), −3.3 (Si*C*H_3), −3.7 (Si*C*H_3), −4.3 (Si*C*H_3), −4.6 (Si*C*H_3), −4.8 (Si*C*H_3); *m/z* (ES^+) 604 (MH^+, 100%), 605 ((MH_2^+, 15); *m/z* HRMS (ES^+) MH^+ calculated for $C_{31}H_{70}NO_4Si_3$ 604.4607, observed 604.4607.

(*R*)-4-((((9*H*-Fluoren-9-yl)methoxy)carbonyl)amino)-3-((*tert*-butyldimethylsilyl)oxy)butanoic acid (6)

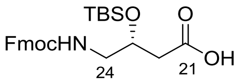

Lithium hydroxide (168 mg, 7.02 mmol) was added to a solution of ester[2] **5** (640 mg, 2.34 mmol) in THF/MeOH/H_2O (23.4 mL, 2:2:3) at room temperature. The mixture was stirred for 2 h and then acidified to pH 3-4 by the slow addition of aqueous HCl (1 M) solution. The aqueous layer was then separated and extracted with EtOAc, dried (Na_2SO_4), filtered and concentrated *in vacuo*. EtOAc (HPLC grade) (10 mL) was added to the crude carboxylic acid and palladium on carbon (170 mg), and the mixture was stirred under an atmospheric (balloon) pressure of hydrogen at room temperature for 48 h. The solution was then filtered over a plug of Celite, washed with $CHCl_3$/MeOH (1:1) and concentrated *in vacuo*. Fmoc *N*-hydroxysuccinimide ester (829 mg, 2.46 mmol) was added to a solution of crude amino acid in CH_3CN/saturated aqueous $NaHCO_3$ (12.0 mL, 1:1) at room temperature.

The mixture was stirred for 16 h and then acidified to pH 3-4 by the slow addition of aqueous HCl (1 M) solution. The aqueous layer was then separated and extracted with EtOAc, dried (Na_2SO_4), filtered and concentrated *in vacuo*. Purification by flash column chromatography (20-30-60-100% EtOAc/petrol) afforded acid **6** (650 mg, 61% over 3 steps) as a colorless foam; R_f: 0.50 (EtOAc); $[\alpha]^{20}_D$ +3.2 (*c* 0.28, MeOH); ν_{max}/cm^{-1} (neat) 2929, 1710, 1520, 1450, 1252, 1109, 1004, 835, 777, 740; δ_H (400 MHz, $CDCl_3$) 7.77 (2H, d, *J* 7.6, 2 x Ar*H*), 7.61-7.58 (2H, m, 2 x Ar*H*), 7.41 (2H, dd, *J* 7.6, 2 x Ar*H*), 7.32 (2H, d, *J* 7.6, 2 x Ar*H*), 5.04 (1H, t, *J* 5.9, N*H*), 4.43 (2H, d, *J* 6.9, OCH_2CH), 4.26-4.21 (2H, m, OCH_2C*H* and 23-*H*), 3.34-3.30 (2H, m, 24-*H*), 2.51 (2H, d, *J* 5.9, 22-*H*), 0.90 (9H, s, SiC(CH_3)$_3$), 0.10 (3H, s, SiCH_3), 0.09 (3H, s, SiCH_3); δ_C (100 MHz, $CDCl_3$) 175.4 (21-*C*), 156.6 (*C*(O)), 143.8 ($C_{(Ar)}$), 141.3 ($C_{(Ar)}$), 127.7 ($CH_{(Ar)}$), 127.0 ($CH_{(Ar)}$), 125.0 ($CH_{(Ar)}$), 120.0 ($CH_{(Ar)}$), 68.00 (OCH_2*C*H or 23-*C*), 66.8 (O*C*H_2CH), 47.2 (OCH_2*C*H or 23-*C*), 46.2 (24-*C*), 39.8 (22-*C*), 26.7 (SiC(*C*H_3)$_3$), 17.9 (Si*C*(CH_3)$_3$), −4.8 (Si*C*H_3), −4.9 (Si*C*H_3); *m/z* (ES^+) 456 (MH^+, 100%); *m/z* HRMS (ES^+) MH^+ calculated for $C_{25}H_{34}NO_5Si$ 456.2201, observed 456.2195.

(9*H*-Fluoren-9-yl)methyl ((*R*)-2-((*tert*-butyldimethylsilyl)oxy)-4-(((5*S*,6*S*,7*S*,8*S*)-7-((*tert*-butyldimethylsilyl)oxy)-5-(hydroxymethyl)-2,2,3,3,10,10,11,11-octamethyl-8-((*S*)-oct-7-en-2-yl)-4,9-dioxa-3,10-disiladodecan-6-yl)amino)-4-oxobutyl)carbamate (8)

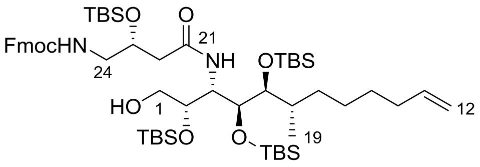

Cyanuric fluoride (0.14 mL, 1.67 mmol) was added dropwise to a solution of **6** (634 mg, 1.39 mmol) and pyridine (0.11 mL, 1.39 mmol) in CH_2Cl_2 (13.0 mL) at room temperature. The mixture was stirred for 3 h. The reaction was diluted with CH_2Cl_2 and washed with ice-cold water (x 1), dried (Na_2SO_4), filtered and concentrated *in vacuo* to afford acid fluoride **7** as a yellow solid, which was used without further purification in the subsequent step. 4-Methylmorpholine (0.24 mL, 2.18 mmol), hexamethyldisiloxane (0.46 mL, 2.18 mmol), a solution of freshly prepared acid fluoride **7** in CH_2Cl_2 (4.20 mL) were added sequentially to a solution of **4** (453 mg, 0.73 mmol) in CH_2Cl_2 (3.00 mL) at room temperature. The mixture was stirred for 20 mins and then concentrated *in vacuo*. Purification by flash column chromatography (5-10% EtOAc/petrol) afforded amide **8** (576 mg, 76%) as a colorless solid; R_f: 0.20 (10% EtOAc/petrol); m.p. 46-49 °C; $[\alpha]^{20}_D$ −3.6 (*c* 0.08, $CHCl_3$); ν_{max}/cm^{-1} (neat)

2956, 2929, 2857, 1658, 1518, 1471, 1406, 1254, 1089, 1058, 938, 876, 833, 775, 740, 674; δ_H (400 MHz, $CDCl_3$) 7.77 (2H, d, *J* 7.3, 2 x Ar*H*), 7.61 (2H, d, *J* 7.3, 2 x Ar*H*), 7.41 (2H, dd, *J* 7.3, 2 x Ar*H*), 7.32 (2H, dd, *J* 7.3, 2 x Ar*H*), 6.31 (1H, br s, N*H*), 5.79 (1H, m, 11-*H*), 5.17 (1H, t, *J* 5.6, N*H*), 5.01-4.93 (2H, m, 12-*H*), 4.44 (1H, dd, *J* 9.8 and 6.9, OC*H*HCH), 4.34-4.17 (5H, m, OCH*H*CH, OCH_2CH, O*H*, 23- and 3-*H*), 4.10-4.01 (2H, m, 4- and 2-*H*), 3.61 (1H, ddd, *J* 11.0 and 5.6, 1-*H*H), 3.50 (1H, dd, *J* 9.3 and 2.2, 5-*H*), 3.37 (2H, dd, *J* 5.4, 24-*H*), 3.23 (1H, ddd, *J* 11.0 and 4.9, 1-H*H*), 2.46 (1H, dd, *J* 15.4 and 3.9, 22-*H*H), 2.35 (1H, dd, *J* 15.4 and 8.6, 22-H*H*), 2.07-2.02 (2H, m, 10-*H*), 1.64 (1H, m, 7-*H*H), 1.54 (1H, m, 6-*H*), 1.44-1.27 (3H, m, 9-*H* and 8-*H*H), 1.11 (1H, m, 8-H*H*), 1.01 (3H, d, *J* 6.6, 19-*H*), 0.95, 0.91, 0.91 and 0.87 (4 x 9H, s, 4 x $SiC(CH_3)_3$), 0.82 (1H, m, 7-H*H*), 0.17, 0.16, 0.15, 0.12, 0.11, 0.11, 0.10, 0.10 (8 x 3H, s, 8 x $SiCH_3$); δ_C (100 MHz, $CDCl_3$) 171.0 (21-*C*), 156.4 (*C*(O)), 144.1 ($C_{(Ar)}$), 144.0 ($C_{(Ar)}$), 141.3 (2 x $C_{(Ar)}$), 138.9 (11-*C*), 127.6 ($CH_{(Ar)}$), 127.0 ($CH_{(Ar)}$), 125.1 ($CH_{(Ar)}$), 119.9 ($CH_{(Ar)}$), 114.4 (12-*C*), 79.6 (5-*C*), 72.6 (4-*C*), 71.3 (2-*C*), 68.0 (23-*C*), 66.8 (OCH_2CH), 62.7 (1-*C*), 52.9 (3-*C*), 47.2 (OCH_2CH), 46.1 (24-*C*), 41.9 (22-*C*), 35.0 (6-*C*), 33.9 (7-*C*), 33.9 (10-*C*), 29.3 (9-*C*), 26.5 (8-*C*), 26.1 (2 x $SiC(CH_3)_3$), 25.9 ($SiC(CH_3)_3$), 25.8 ($SiC(CH_3)_3$), 18.3 ($SiC(CH_3)_3$), 18.0 (2 x $SiC(CH_3)_3$), 17.9 ($SiC(CH_3)_3$), 17.0 (19-*C*), −2.4 ($SiCH_3$), −3.1 ($SiCH_3$), −3.4 ($SiCH_3$), −3.7 ($SiCH_3$), −4.4 ($SiCH_3$), −4.6 ($SiCH_3$), −4.7 ($SiCH_3$), −4.9 ($SiCH_3$); *m/z* (ES^+) 1064 ((MNa^+, 100); *m/z* HRMS (ES^+) MNa^+ calculated for $C_{56}H_{100}N_2O_8Si_4Na$ 1063.6449, observed 1063.6455.

(9*H*-Fluoren-9-yl)methyl ((*R*)-2-((*tert*-butyldimethylsilyl)oxy)-4-(((5*S*,6*R*,7*S*,8*S*)-7-((*tert*-butyldimethylsilyl)oxy)-5-formyl-2,2,3,3,10,10,11,11-octamethyl-8-((*S*)-oct-7-en-2-yl)-4,9-dioxa-3,10-disiladodecan-6-yl)amino)-4-oxobutyl)carbamate (8a)

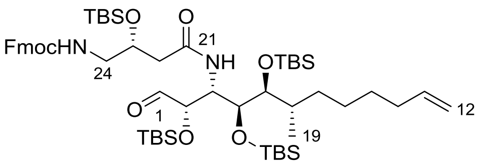

Dess-Martin periodinane (352 mg, 0.83 mmol) was added to a solution of alcohol **8** (246 mg, 0.24 mmol) in wet CH_2Cl_2 (2.40 mL) at 0 °C. The solution was then warmed to room temperature and stirred for 3 h. Saturated aqueous $NaHCO_3$ was then added and the aqueous layer was then separated and extracted with CH_2Cl_2, dried (Na_2SO_4), filtered and concentrated *in vacuo*. Purification by flash column chromatography (5-10% EtOAc/petrol) afforded aldehyde **8a** (212 mg, 86%) as a colorless solid; R_f: 0.50 (10% EtOAc/petrol);

m.p. 42-44 ^{o}C; $[\alpha]^{20}_{D}$ −9.0 (*c* 0.1, $CHCl_3$); ν_{max} /cm^{-1} (neat) 2953, 2929, 2857, 1673, 1515, 1471, 1390, 1252, 1097, 1005, 938, 872, 832, 813, 775, 758, 674; δ_H (400 MHz, $CDCl_3$) 9.55 (1H, s, 1-*H*), 7.77 (2H, d, *J* 7.6, 2 x Ar*H*), 7.62 (2H, d, *J* 7.6, 2 x Ar*H*), 7.41 (2H, dd, *J* 7.6, 2 x Ar*H*), 7.32 (2H, dd, *J* 7.6, 2 x Ar*H*), 6.87 (1H, d, *J* 4.9, N*H*), 5.80 (1H, m, 11-*H*), 5.22 (1H, t, *J* 5.4, N*H*), 5.01-4.93 (2H, m, 12-*H*), 4.41 (1H, dd, *J* 10.3 and 7.3, OC*H*HCH), 4.31-4.20 (4H, m, OCH*H*CH, OCH_2CH, 2- and 23-*H*), 4.02 (1H, dd, *J* 9.5 and 5.4, 3-*H*), 3.91 (1H, dd, *J* 9.5 and 2.0, 4-*H*), 3.56 (1H, dd, *J* 9.1 and 2.0, 5-*H*), 3.32-3.31 (2H, m, 24-*H*), 2.35 (2H, d, *J* 6.1, 22-*H*), 2.07-2.02 (2H, m, 10-*H*), 1.74 (1H, m, 6-*H*), 1.63 (1H, m, 7-*H*H), 1.42-1.27 (3H, m, 9-*H* and 8-*H*H), 1.13 (1H, m, 8-H*H*), 1.01 (3H, d, *J* 6.9, 19-*H*), 0.96, 0.91, 0.91 and 0.88 (4 x 9H, s, 4 x $SiC(CH_3)_3$), 0.95 (1H, m, 7-H*H*), 0.18, 0.17, 0.14, 0.13, 0.13, 0.09, 0.08, 0.06 (8 x 3H, s, 8 x $SiCH_3$); δ_C (100 MHz, $CDCl_3$) 200.0 (1-*C*), 169.3 (21-*C*), 156.4 (*C*(O)), 144.1 ($C_{(Ar)}$), 144.0 ($C_{(Ar)}$), 141.3 ($C_{(Ar)}$), 141.2 ($C_{(Ar)}$), 138.9 (11-*C*), 127.6 ($CH_{(Ar)}$), 127.0 ($CH_{(Ar)}$), 125.2 ($CH_{(Ar)}$), 119.9 ($CH_{(Ar)}$), 114.4 (12-*C*), 79.3 (5-*C*), 76.9 (2-*C*), 73.2 (4-*C*), 68.0 (23-*C*), 66.8 (OCH_2CH), 55.3 (3-*C*), 47.2 (OCH_2CH), 46.7 (24-*C*), 42.1 (22-*C*), 35.1 (6-*C*), 33.9 (7- or 10-*C*), 33.8 (7- or 10-*C*), 29.4 (9-*C*), 26.3 (8-*C*), 26.1 (2 x $SiC(CH_3)_3$), 25.8 (2 x $SiC(CH_3)_3$), 18.4 ($SiC(CH_3)_3$), 18.0 (3 x $SiC(CH_3)_3$), 16.9 (19-*C*), −2.4 ($SiCH_3$), −3.4 ($SiCH_3$), −3.5 ($SiCH_3$), −4.0 ($SiCH_3$), −4.6 ($SiCH_3$), −4.6 ($SiCH_3$), −4.7 ($SiCH_3$), −4.9 ($SiCH_3$); *m/z* HRMS (ES$^+$) MNa$^+$ calculated for $C_{56}H_{98}N_2O_8Si_4Na$ 1061.6292, observed 1061.6294.

(2*S*,3*R*,4*S*,5*S*,6*S*)-3-((*R*)-4-((((9*H*-Fluoren-9-yl)methoxy)carbonyl)amino)-3-((*tert*-butyldimethylsilyl)oxy)butanamido)-2,4,5-tris((*tert*-butyldimethylsilyl)oxy)-6-methyldodec-11-enoic acid (9)

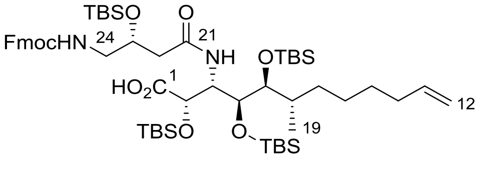

2-Methyl-2-butene (64.0 μL, 0.61 mmol), NaH_2PO_4 (97.0 mg, 0.81 mmol) and $NaClO_2$ (24.0 mg, 0.26 mmol) were added sequentially to a solution of aldehyde **8a** (212 mg, 0.20 mmol) in *tert*-butanol/water (1.44 mL, 2.5:1) at 0 ^{o}C. The solution was then slowly warmed to room temperature and stirred for 2.5 h. The solution was then diluted with EtOAc, water was added and the aqueous layer was then separated and extracted with EtOAc, dried (Na_2SO_4), filtered and concentrated *in vacuo* to afford crude acid **9** (217 mg, quant.) as a

colorless solid. R_f: 0.50 (30% EtOAc/petrol); δ_H (400 MHz, CD_2Cl_2) 7.76 (2H, d, *J* 7.3, 2 x Ar*H*), 7.61 (2H, d, *J* 7.6, 2 x Ar*H*), 7.38 (2H, dd, *J* 7.6, 2 x Ar*H*), 7.31-7.26 (2H, m, 2 x Ar*H*), 6.51 (1H, br s, N*H*), 5.80 (1H, m, 11-*H*), 5.37 (1H, m, N*H*), 5.01-4.89 (2H, m, 12-*H*), 4.53-4.21 (6H, m, OCH_2CH, OCH$_2$C*H*, 2-, 3- and 23-*H*), 3.90 (1H, dd, *J* 9.8 and 2.0, 4-*H*), 3.52 (1H, dd, *J* 8.8 and 2.2, 5-*H*), 3.36-3.21 (2H, m, 24-*H*), 2.41 (1H, dd, *J* 15.4 and 4.7, 22-*H*H), 2.26 (1H, dd, *J* 15.4 and 9.5, 22-H*H*), 2.05-2.00 (2H, m, 10-*H*), 1.69-1.58 (2H, m, 6-*H* and 7-*H*H), 1.43-1.29 (3H, m, 9-*H* and 8-*H*H), 1.14 (1H, m, 8-H*H*), 1.01 (3H, d, *J* 6.6, 19-*H*), 0.93, 0.93, 0.91 and 0.86 (4 x 9H, s, 4 x SiC(CH_3)$_3$), 0.88 (1H, m, 7-H*H*), 0.19, 0.18, 0.17, 0.16, 0.11, 0.11, 0.07, 0.06 (8 x 3H, s, 8 x SiCH_3). The compound was used immediately and without further purification in the subsequent step.

Synthesis of tripeptide 17.

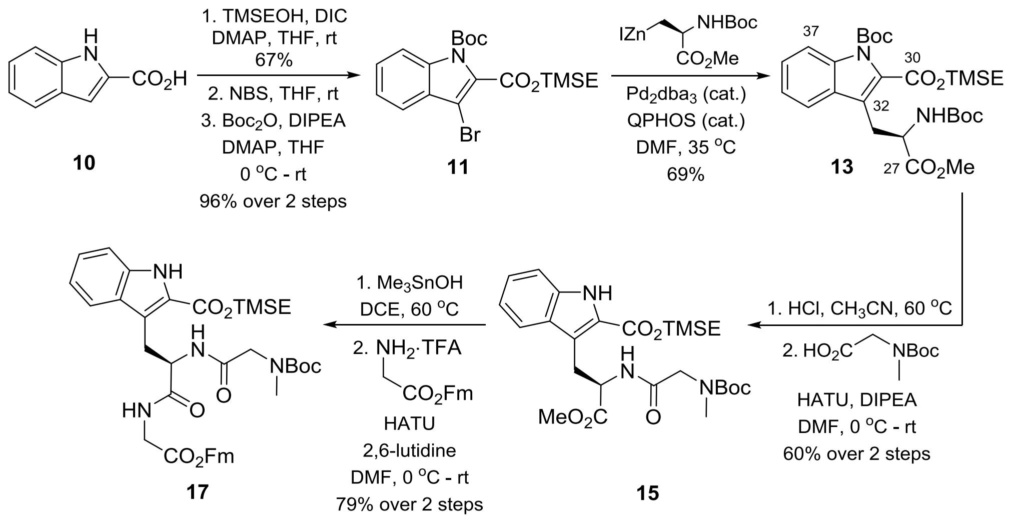

2-(Trimethylsilyl)ethyl 1*H*-indole-2-carboxylate (10a)

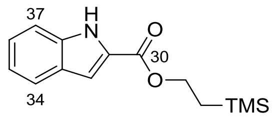

N,*N*′-Diisopropylcarbodiimide (0.60 mL, 3.90 mmol), DMAP (79.0 mg, 0.65 mmol) and 2-(trimethylsilyl)ethanol (0.56 mL, 3.90 mmol) were added sequentially to a solution of indole-2-carboxylic acid (524 mg, 3.25 mmol) in THF (32.0 mL) at room temperature. The mixture was stirred for 2 h. Additional portions of *N*,*N*′-diisopropylcarbodiimide (0.50 mL, 3.25 mmol), DMAP (40.0 mg, 0.33 mmol) and 2-(trimethylsilyl)ethanol (0.47 mL,

3.25 mmol) were then added, and the mixture was stirred for 16 h. The solution was then filtered over a plug of Celite, washed with CH_2Cl_2 and concentrated *in vacuo* onto silica gel. Purification by flash column chromatography (5% EtOAc/petrol) afforded ester **10a** (571 mg, 67%) as a colorless solid; R_f: 0.40 (10% EtOAc/petrol); m.p. 122-124 ^{o}C; ν_{max}/cm^{-1} (neat) 3340, 1686, 1531, 1454, 1364, 1314, 1251, 1198, 1167, 1040, 978, 839, 771, 739, 703, 645; δ_H (400 MHz, $CDCl_3$) 9.22 (1H, br s, N*H*), 7.75 (1H, d, *J* 7.8, 34-*H*), 7.49 (1H, d, *J* 8.1, 37-*H*), 7.38 (1H, dd, *J* 7.8, 36-*H*), 7.27 (1H, s, 32-*H*), 7.21 (1H, dd, *J* 7.8, 35-*H*), 4.53 (2H, t, *J* 8.3, TMSCH$_2$C*H$_2$*), 1.22 (2H, t, *J* 8.3, TMSC*H$_2$*CH$_2$), 0.16 (9H, s, Si(C*H$_3$*)$_3$); δ_C (100 MHz, $CDCl_3$) 162.3 (30-*C*), 136.8 (*C*$_{(Ar)}$), 127.7 (*C*$_{(Ar)}$), 127.5 (*C*$_{(Ar)}$), 125.2 (36-*C*), 122.6 (34-*C*), 120.7 (35-*C*), 111.9 (37-*C*), 108.4 (32-*C*), 63.4 (TMSCH$_2$*C*H$_2$), 17.5 (TMS*C*H$_2$CH$_2$), −1.4 (Si(*C*H$_3$)$_3$); *m/z* (ES$^+$) 284 (MNa$^+$, 100%); *m/z* HRMS (ES$^-$) M−H calculated for $C_{14}H_{18}O_2NSi$ 260.1112, observed 260.1114.

1-*tert*-Butyl 2-(2-(trimethylsilyl)ethyl) 3-bromo-1*H*-indole-1,2-dicarboxylate (11)

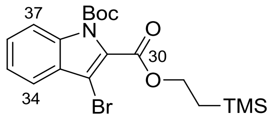

N-Bromosuccinimide (3.13 g, 17.6 mmol) was added in one portion to a solution ester **10a** (2.00 g, 7.66 mmol) in THF (76.0 mL) at room temperature. The mixture was stirred for 2.5 h. Saturated aqueous Na_2SO_3 was then added, the aqueous layer was separated and extracted with EtOAc, dried (Na_2SO_4), filtered and concentrated *in vacuo*. *N,N*-Diisopropylethylamine (2.70 mL, 15.3 mmol), DMAP (468 mg, 3.83 mmol) and di-*tert*-butyl dicarbonate (8.80 mL, 38.3 mmol) were added sequentially to a solution of the crude bromide in THF (51.0 mL) at 0 ^{o}C. The solution was then warmed to room temperature and stirred for 16 h. Water was added and aqueous layer was separated and extracted with EtOAc, dried (Na_2SO_4), filtered and concentrated *in vacuo* onto silica gel. Purification by flash column chromatography (petrol-1% EtOAc/petrol) afforded bromide **11** (3.25 g, 96% over 2 steps) as a viscous colorless oil, which solidified in the freezer; R_f: 0.40 (5% EtOAc/petrol); m.p. 44-46 ^{o}C; ν_{max}/cm^{-1} (neat) 2950, 1727, 1558, 1448, 1363, 1326, 1250, 1230, 1151, 1114, 1074, 973, 858, 835, 740, 697, 642; δ_H (400 MHz, $CDCl_3$) 8.11 (1H, d, *J* 8.3, 34-*H*), 7.59 (1H, d, *J* 7.6, 37-*H*), 7.46 (1H, ddd, *J* 8.3, 7.6 and 1.5, 35-*H*), 7.35 (1H, ddd, *J* 8.3, 7.6 and 1.0, 36-*H*), 4.50-4.46 (2H, m, TMSCH$_2$C*H$_2$*), 1.64 (9H, s, CO$_2$C(C*H$_3$*)$_3$),

1.21-1.16 (2H, m, TMSCH_2CH$_2$), 0.10 (9H, s, Si(CH_3)$_3$); δ_C (100 MHz, CDCl$_3$) 161.6 (30-*C*), 148.5 (*C*(O)), 135.5 ($C_{(Ar)}$), 127.9 (2 x $C_{(Ar)}$), 127.4 (35-*C*), 123.8 (36-*C*), 120.7 (37-*C*), 115.1 (34-*C*), 102.4 ($C_{(Ar)}$), 85.3 (CO$_2$*C*(CH$_3$)$_3$), 64.4 (TMSCH$_2$*C*H$_2$), 27.9 (CO$_2$C(*C*H$_3$)$_3$), 17.4 (TMS*C*H$_2$CH$_2$), −1.5 (Si(*C*H$_3$)$_3$); *m/z* (ES$^+$) 462 (MNa$^+$, 100%); *m/z* HRMS (ES$^+$) MNa$^+$ calculated for $C_{19}H_{26}O_4NSiBrNa$ 462.0707, observed 462.0697.

(*R*)-1-*tert*-Butyl 2-(2-(trimethylsilyl)ethyl) 3-(2-((*tert*-butoxycarbonyl)amino)-3-methoxy-3-oxopropyl)-1*H*-indole-1,2-dicarboxylate (13)

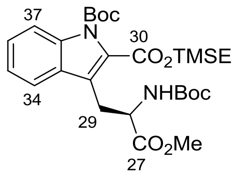

1,2-Dibromoethane (0.16 mL, 1.82 mmol) was added to a suspension of zinc dust (2.32 g, 35.4 mmol) in DMF (8.00 mL) at room temperature. The mixture was heated to 50 ^oC and stirred for 30 mins. Then the reaction mixture was allowed to cool to room temperature afterwhich chlorotrimethylsilane (50.0 μL, 0.36 mmol) was added, and the mixture was stirred for further 30 mins. *N*-(*tert*-Butoxycarbonyl)-3-iodo-D-alanine methyl ester (1.94 g, 5.90 mmol) in DMF (8.00 mL) was added to the mixture, which was then stirred for 2 h at room temperature. The reaction mixture was then standing for another 30 mins, and the supernatant liquid was transferred to a solution of bromide **11** (2.00 g, 4.54 mmol), Pd$_2$(dba)$_3$ (208 mg, 0.23 mmol) and QPhos (260 mg, 0.37 mmol) in DMF (2.00 mL). The resultant mixture was heated to 35 ^oC, stirred for 2 h and then concentrated *in vacuo* onto silica gel. Purification by flash column chromatography (2-5-10% EtOAc/petrol) afforded protected amino acid **13** (1.76 g, 69%) as a colorless foam; R_f: 0.30 (10% EtOAc/petrol); $[\alpha]^{20}_D$ +4.0 (*c* 0.48, CHCl$_3$); ν_{max}/cm^{-1} (neat) 2978, 1716, 1502, 1453, 1366, 1328, 1247, 1215, 1154, 1103, 1061, 939, 837, 748, 697; δ_H (400 MHz, CDCl$_3$) 8.05 (1H, d, *J* 8.6, 34-*H*), 7.66 (1H, d, *J* 7.8, 37-*H*), 7.42 (1H, dd, *J* 7.8, 35-*H*), 7.30 (1H, dd, *J* 7.8, 36-*H*), 5.77 (1H, d, *J* 7.1, N*H*), 4.51 (1H, m, 28-*H*), 4.46-4.40 (2H, m, TMSCH$_2$CH_2), 3.69 (3H, s, CO$_2$CH_3), 3.30 (1H, dd, *J* 13.9 and 5.9, 29-*H*H), 3.23 (1H, dd, *J* 13.9 and 8.3, 29-H*H*), 1.64 (9H, s, CO$_2$C(CH_3)$_3$), 1.40 (9H, s, CO$_2$C(CH_3)$_3$), 1.16 (2H, t, *J* 9.5, TMSCH_2CH$_2$), 0.09 (9H, s, Si(CH_3)$_3$); δ_C (100 MHz, CDCl$_3$) 172.5 (27-*C*), 162.6 (30-*C*), 155.4 (*C*(O)), 149.2 (*C*(O)), 136.5 ($C_{(Ar)}$), 128.7 ($C_{(Ar)}$), 128.3 ($C_{(Ar)}$), 126.9 (35-*C*), 123.3 (36-*C*), 121.3 ($C_{(Ar)}$), 120.2 (37-*C*), 115.0

(34-*C*), 84.6 ($CO_2C(CH_3)_3$), 79.7 ($CO_2C(CH_3)_3$), 64.3 ($TMSCH_2CH_2$), 53.8 (28-*C*), 52.3 (CO_2CH_3), 28.3 ($CO_2C(CH_3)_3$), 28.0 ($CO_2C(CH_3)_3$), 27.2 (29-*C*), 17.3 ($TMSCH_2CH_2$), −1.6 ($Si(CH_3)_3$); *m/z* (ES^+) 585 (MNa^+, 100%); *m/z* HRMS (ES^+) MNa^+ calculated for $C_{28}H_{42}O_8N_2SiNa$ 585.2603, observed 585.2596.

(*R*)-2-(Trimethylsilyl)ethyl 3-(2-(2-((*tert*-butoxycarbonyl)(methyl)amino)acetamido)-3-methoxy-3-oxopropyl)-1*H*-indole-2-carboxylate (15)

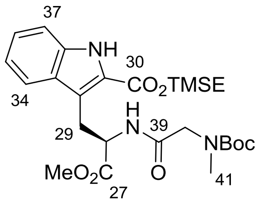

Hydrogen chloride (3.00 mL of a 4 M solution in dioxane, 12.0 mmol) was added to a solution of protected amino acid **13** (0.83 g, 1.47 mmol) in CH_3CN (10.0 mL) at room temperature. The mixture was heated to 60 °C and stirred for 1 h 40 mins. After this time the reaction was then cooled to room temperature and slowly quenched with saturated aqueous $NaHCO_3$. EtOAc was added and the aqueous layer separated and extracted with EtOAc, dried (Na_2SO_4), filtered and concentrated *in vacuo* to afford the crude amine as a pale yellow oil. The amine was dissolved in DMF (10.0 mL) and cooled to 0 °C. *N*-Boc-Sar (324 mg, 1.71 mmol), HATU (732 mg, 1.93 mmol) and *N,N*-diisopropylethylamine (0.75 mL, 4.28 mmol) were then added sequentially. The mixture was warmed slowly to room temperature and stirred for 3 h. The reaction was then diluted with EtOAc and washed with brine solution (x 2), dried (Na_2SO_4), filtered and concentrated *in vacuo*. Purification by flash column chromatography (25-40% EtOAc/petrol) afforded dipeptide **15** (471 mg, 60% over 2 steps) as a colorless foam; R_f: 0.50 (50% EtOAc/petrol); m.p. 43-45 °C; $[\alpha]^{20}_D$ −13.5 (*c* 0.43, $CHCl_3$); ν_{max}/cm^{-1} (neat) 3335, 3066, 2955, 1747, 1699, 1670, 1544, 1526, 1446, 1394, 1383, 1322, 1247, 1145, 1095, 1053, 935, 867, 837, 747, 697; δ_H (400 MHz, $CDCl_3$) 9.03 (1H, br s, N*H*), 7.69 (1H, d, *J* 8.1, 34-*H*), 7.37 (1H, d, *J* 8.1, 37-*H*), 7.31 (1H, dd, *J* 7.8, 36-*H*), 7.16 (1H, dd, *J* 7.8, 35-*H*), 4.82 (1H, br s, 28-*H*), 4.48 (2H, br s, $TMSCH_2CH_2$), 3.86-3.82 (2H, m, 40-*H*), 3.68 (3H, br s, CO_2CH_3), 3.60 (2H, d, *J* 5.6, 29-*H*), 2.73 (3H, s, 41-*H*), 1.38 (9H, s, $CO_2C(CH_3)_3$), 1.17 (2H, t, *J* 8.8, $TMSCH_2CH_2$), 0.10 (9H, s, $Si(CH_3)_3$); δ_C (100 MHz, $CDCl_3$) 172.0 (27-*C*), 169.6 (39-*C*), 162.3 (30-*C*), 155.1 (*C*(O)), 135.6 ($C_{(Ar)}$), 127.8 ($C_{(Ar)}$), 125.8 (36-*C*), 124.4 ($C_{(Ar)}$), 120.7 (35-*C*), 120.2 (34-*C*), 118.4 ($C_{(Ar)}$), 111.9

(37-*C*), 80.3 ($CO_2C(CH_3)_3$), 63.8 ($TMSCH_2CH_2$), 53.8 (28-*C*), 53.0 (40-*C*), 52.2 (CO_2CH_3), 35.2 (41-*C*), 28.1 ($CO_2C(CH_3)_3$), 26.5 (29-*C*), 17.6 ($TMSCH_2CH_2$), −1.6 ($Si(CH_3)_3$); *m/z* (ES^+) 534 (MH^+, 100%); *m/z* HRMS (ES^+) MH^+ calculated for $C_{26}H_{40}O_7N_3Si$ 534.2630, observed 534.2613.

(*R*)-2-(Trimethylsilyl)ethyl 3-(3-((2-((9*H*-fluoren-9-yl)methoxy)-2-oxoethyl)amino)-2-(2-((*tert*-butoxycarbonyl)(methyl)amino)acetamido)-3-oxopropyl)-1*H*-indole-2-carboxylate (17)

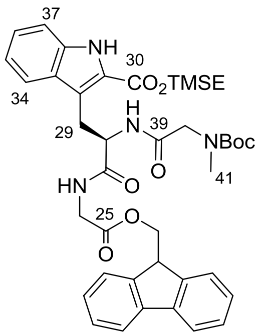

Trimethyltin hydroxide (1.00 g, 5.53 mmol) was added to a solution of dipeptide **15** (378 mg, 0.71 mmol) in 1,2-DCE (7.00 mL) at room temperature. The mixture was heated to 60 °C and stirred for 5.5 h. After this time the reaction was then cooled to room temperature, diluted with EtOAc, washed with 1M $KHSO_4$ (x 3), dried (Na_2SO_4), filtered and concentrated *in vacuo* to afford the crude acid as a colorless foam. The acid was dissolved in DMF (7.00 mL) and cooled to 0 °C. 2,6-Lutidine (0.25 mL, 2.12 mmol), HATU (296 mg, 0.78 mmol) and Gly-OFm·TFA (273 mg, 0.74 mmol) were then added sequentially. The mixture was warmed slowly to room temperature and stirred for 16 h. The reaction was then diluted with EtOAc and washed with brine solution (x 2), dried (Na_2SO_4), filtered and concentrated *in vacuo*. Purification by flash column chromatography (30-50% EtOAc/petrol) afforded tripeptide **17** (423 mg, 79% over 2 steps) as a colorless solid; R_f: 0.50 (60% EtOAc/petrol); m.p. 112-114 °C; $[\alpha]^{20}_D$ +5.3 (*c* 0.68, MeOH); ν_{max}/cm^{-1} (neat) 2949, 1748, 1669, 1543, 1448, 1396, 1320, 1249, 1152, 1098, 1039, 839, 744; δ_H (400 MHz, D_4-MeOD (* denotes major rotamer)) 7.75-7.74 (3H, m, 3 x Ar*H*), 7.61 (2H, d, *J* 7.3, 2 x Ar*H*), 7.41 (1H, d, *J* 8.3, Ar*H*), 7.37-7.24 (5H, m, 5 x Ar*H*), 7.10 (1H, dd, *J* 7.6, Ar*H*), 4.82 (1H, m, 28-*H*), 4.52-4.47 (2H, m, $TMSCH_2CH_2$), 4.43-4.39 (2H, m, OCH_2CH), 4.20 (1H, t, *J* 6.9, OCH_2CH), 3.96* and 3.95 (2H, s, 26-*H*), 3.78-3.44 (4H, m, 40- and 29-*H*), 2.67* and 2.59 (3H, s, 41-*H*), 1.41 and 1.22* (9H, s, $CO_2C(CH_3)_3$), 1.27-1.22 (2H, m,

TMSCH_2CH$_2$), 0.09 (9H, s, Si(CH_3)$_3$); δ_C (126 MHz, D$_4$-MeOD (chemical shifts reported for the major rotamer only)) 174.4 (27-*C*), 171.1 (25- and 39-*C*), 164.4 (30-*C*), 157.3 (*C*(O)), 145.1 (2 x $C_{(Ar)}$), 142.7 ($C_{(Ar)}$), 138.0 ($C_{(Ar)}$), 129.0 ($CH_{(Ar)}$), 128.4 ($CH_{(Ar)}$ and $C_{(Ar)}$), 126.5 ($CH_{(Ar)}$), 126.3 ($CH_{(Ar)}$), 125.9 ($C_{(Ar)}$), 121.4 ($CH_{(Ar)}$), 121.3 ($CH_{(Ar)}$), 121.1 ($CH_{(Ar)}$), 120.0 ($C_{(Ar)}$), 113.5 ($CH_{(Ar)}$), 81.7 ($CO_2C(CH_3)_3$), 68.2 (O*C*H$_2$CH), 64.6 (TMSCH$_2$*C*H$_2$), 56.6 (28-*C*), 53.5 (40-*C*), 48.0 (OCH$_2$*C*H), 42.3 (26-*C*), 36.2 (41-*C*), 28.8 (CO$_2$C(*C*H$_3$)$_3$), 28.6 (29-*C*), 18.8 (TMS*C*H$_2$CH$_2$), −1.3 (Si(*C*H$_3$)$_3$); δ_H (500 MHz, D$_6$-DMSO, 363 K) 11.21 (1H, s, N*H*), 8.01 (1H, m, N*H*), 7.85 (2H, d, *J* 7.6, 2 x Ar*H*), 7.79 (1H, d, *J* 8.2, 34-*H*), 7.67 (2H, d, *J* 7.6, 2 x Ar*H*), 7.53 (1H, d, *J* 7.9, N*H*), 7.44-7.39 (3H, m, 2 x Ar*H* and 37-*H*), 7.33 (2H, dd, *J* 7.6, 2 x Ar*H*), 7.23 (1H, dd, *J* 7.9, 36-*H* or 35-*H*), 7.06 (1H, dd, *J* 7.9, 35-*H* or 36-*H*), 4.73 (1H, m, 28-*H*), 4.46-4.42 (2H, m, TMSCH$_2$CH_2), 4.38 (2H, d, *J* 7.3, OCH_2CH), 4.25 (1H, dd, *J* 7.0, OCH$_2$C*H*), 3.91 (1H, dd, *J* 17.3 and 6.0, 26-*H*H), 3.85 (1H, dd, *J* 17.3 and 5.7, 26-H*H*), 3.72 (1H, d, *J* 16.4, 40-*H*H), 3.56 (1H, d, *J* 16.4, 40-H*H*), 3.53 (1H, dd, *J* 13.6 and 4.7, 29-*H*H), 3.38 (1H, dd, *J* 13.6 and 9.1, 29-H*H*), 2.54 (3H, s, 41-*H*), 1.32 (9H, s, CO$_2$C(CH_3)$_3$), 1.21-1.17 (2H, m, TMSCH_2CH$_2$), 0.08 (9H, s, Si(CH_3)$_3$); *m/z* (ES$^+$) 756 (MH$^+$, 100%), 777 (MNa$^+$, 75%); *m/z* HRMS (ES$^+$) MH$^+$ calculated for $C_{41}H_{51}O_8N_4Si$ 755.3471, observed 755.3454.

The enantiopurity of amino acid 13 was confirmed by removal the Boc groups and formation of the corresponding Mosher's amides:

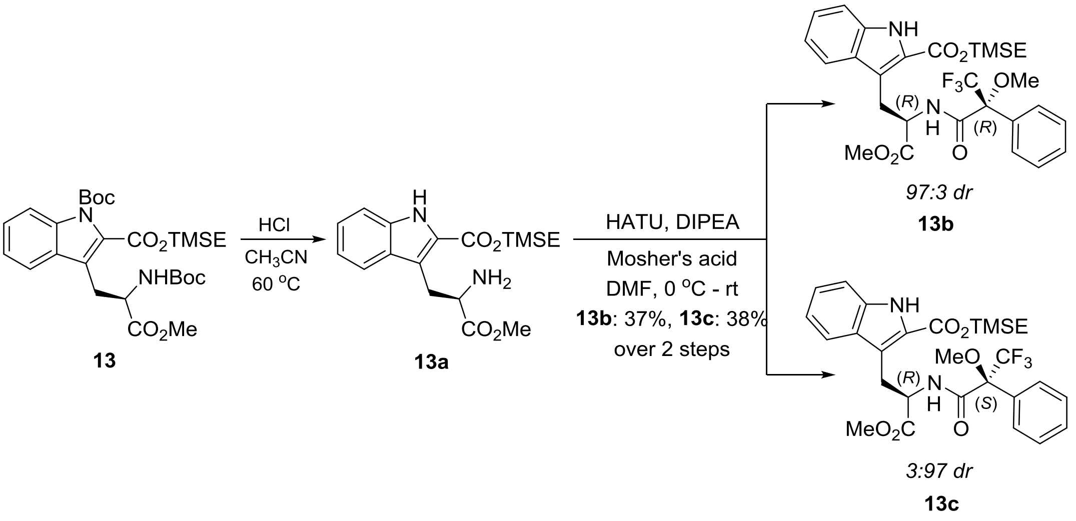

2-(Trimethylsilyl)ethyl 3-((*R*)-3-methoxy-3-oxo-2-((*R*)-3,3,3-trifluoro-2-methoxy-2-phenylpropanamido)propyl)-1*H*-indole-2-carboxylate (13b)

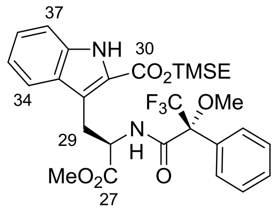

Hydrogen chloride (0.17 mL of a 4 M solution in dioxane, 0.68 mmol) was added to a solution of protected amino acid **13** (38.5 mg, 68.4 μmol) in CH_3CN (0.5 mL) at room temperature. The mixture was heated to 60 ^oC and stirred for 1 h. After this time the reaction was then cooled to room temperature and slowly quenched with saturated aqueous $NaHCO_3$. EtOAc was added and the aqueous layer separated and extracted with EtOAc, dried (Na_2SO_4), filtered and concentrated *in vacuo* to afford the crude amine as a pale yellow oil. The amine was dissolved in DMF (0.5 mL) and cooled to 0 ^oC. (*R*)-(+)-α-methoxy-α-(trifluoromethyl)phenylacetic acid (16.5 mg, 70.4 μmol), HATU (30.2 mg, 79.0 μmol) and *N*,*N*-diisopropylethylamine (31.0 μL, 0.18 mmol) were then added sequentially. The mixture was warmed slowly to room temperature and stirred for 16 h. The reaction was then diluted with EtOAc and washed with brine solution (x 2), dried (Na_2SO_4), filtered and concentrated *in vacuo*. Purification by flash column chromatography (20% EtOAc/petrol) afforded amide **13b** (14.5 mg, 37% over 2 steps) as a colorless oil; R_f: 0.40 (20% EtOAc/petrol); $[\alpha]^{25}_D$ −7.5 (*c* 1.13, $CHCl_3$); ν_{max}/cm^{-1} (neat) 3333, 2954, 1747, 1687, 1520, 1438, 1319, 1249, 1165, 1120, 1098, 996, 942, 837, 744, 720, 696; δ_H (400 MHz, $CDCl_3$) 8.53 (1H, s, N*H*), 7.71 (1H, d, *J* 8.1, 34-*H*), 7.64 (1H, d, *J* 8.1, N*H*), 7.40-7.36 (2H, m, 37-*H* and Ar*H*), 7.29 (1H, dd, *J* 7.1, 36-*H*), 7.18 (1H, ddd, *J* 8.1 and 1.7, 35-*H*), 7.15-7.06 (4H, m, 4 xAr*H*), 4.93 (1H, m, 28-*H*), 4.35-4.23 (2H, m, $TMSCH_2CH_2$), 3.78 (3H, s, CO_2CH_3), 3.59-3.56 (2H, m, 29-*H*), 3.52 (3H, d, *J* 1.7, OCH_3), 1.16-1.11 (2H, m, $TMSCH_2CH_2$), 0.11 (9H, s, $Si(CH_3)_3$); δ_C (100 MHz, $CDCl_3$) 171.8 (27-*C*), 166.6 (*C*(O)), 161.9 (30-*C*), 135.5 ($C_{(Ar)}$), 133.1 ($C_{(Ar)}$), 128.9 ($CH_{(Ar)}$), 128.0 ($CH_{(Ar)}$), 127.4 ($C_{(Ar)}$), 127.0 ($CH_{(Ar)}$), 126.1 (36-*C*), 124.3 ($C_{(Ar)}$), 123.5 (q, $^1J_{C-F}$ 290.9, CF_3), 120.9 (35-*C*), 120.2 (34-*C*), 118.1 ($C_{(Ar)}$), 111.8 (37-*C*), 84.0 (q, $^2J_{C-F}$ 26.2, $C(OCH_3)(CF_3)Ph$), 63.7 ($TMSCH_2CH_2$), 55.2 (OCH_3), 53.2 (28-*C*), 52.5 (CO_2CH_3), 26.5 (29-*C*), 17.5 ($TMSCH_2CH_2$), −1.5 ($Si(CH_3)_3$); δ_F (376 MHz, $CDCl_3$) −69.2; *m/z* (ES^+) 579 (MH^+, 100%); *m/z* HRMS (ES^+) MH^+ calculated for $C_{28}H_{34}O_6N_2F_3Si$ 579.2133, observed 579.2120.

2-(Trimethylsilyl)ethyl 3-((*R*)-3-methoxy-3-oxo-2-((*S*)-3,3,3-trifluoro-2-methoxy-2-phenylpropanamido)propyl)-1*H*-indole-2-carboxylate (13c)

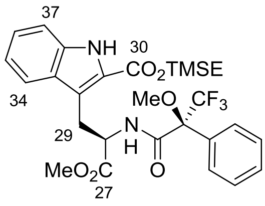

Hydrogen chloride (0.17 mL of a 4 M solution in dioxane, 0.68 mmol) was added to a solution of protected amino acid **13** (38.5 mg, 68.4 μmol) in CH_3CN (0.5 mL) at room temperature. The mixture was heated to 60 ^{o}C and stirred for 1 h. After this time the reaction was then cooled to room temperature and slowly quenched with saturated aqueous $NaHCO_3$. EtOAc was added and the aqueous layer separated and extracted with EtOAc, dried (Na_2SO_4), filtered and concentrated *in vacuo* to afford the crude amine as a pale yellow oil. The amine was dissolved in DMF (0.5 mL) and cooled to 0 ^{o}C. (*S*)-(−)-α-methoxy-α-(trifluoromethyl)phenylacetic acid (16.5 mg, 70.4 μmol), HATU (30.2 mg, 79.0 μmol) and *N,N*-diisopropylethylamine (31.0 μL, 0.18 mmol) were then added sequentially. The mixture was warmed slowly to room temperature and stirred for 16 h. The reaction was then diluted with EtOAc and washed with brine solution (x 2), dried (Na_2SO_4), filtered and concentrated *in vacuo*. Purification by flash column chromatography (20% EtOAc/petrol) afforded amide **13c** (15.1 mg, 38% over 2 steps) as a colorless oil; R_f: 0.40 (20% EtOAc/petrol); $[\alpha]^{25}_D$ −35.5 (*c* 1.00, $CHCl_3$); ν_{max}/cm^{-1} (neat) 3337, 2953, 1745, 1686, 1515, 1439, 1318, 1249, 1164, 1120, 1098, 993, 947, 837, 744, 721, 696; δ_H (400 MHz, $CDCl_3$) 8.82 (1H, s, N*H*), 7.84 (1H, d, *J* 6.9, N*H*), 7.74 (1H, d, *J* 8.1, 34-*H*), 7.56-7.54 (2H, m, 2 x Ar*H*), 7.41-7.36 (5H, m, 3 x Ar*H*, 36- and 37-*H*), 7.20 (1H, ddd, *J* 8.1 and 1.5, 35-*H*), 4.81 (1H, m, 28-*H*), 4.51-4.38 (2H, m, TMS$CH_2C\mathit{H_2}$), 3.70 (3H, s, $CO_2C\mathit{H_3}$), 3.66-3.64 (2H, m, 29-*H*), 3.16 (3H, d, *J* 1.2, $OC\mathit{H_3}$), 1.22-1.08 (2H, m, TMS$C\mathit{H_2}CH_2$), 0.10 (9H, s, $Si(C\mathit{H_3})_3$); δ_C (100 MHz, $CDCl_3$) 171.7 (27-*C*), 166.6 (*C*(O)), 162.3 (30-*C*), 135.7 ($C_{(Ar)}$), 132.3 ($C_{(Ar)}$), 129.4 ($CH_{(Ar)}$), 128.3 ($CH_{(Ar)}$), 128.1 ($CH_{(Ar)}$), 127.5 ($C_{(Ar)}$), 126.1 (36-*C*), 124.4 ($C_{(Ar)}$), 123.6 (q, $^1J_{C-F}$ 289.3, CF_3), 120.9 (35-*C*), 120.3 (34-*C*), 118.2 ($C_{(Ar)}$), 111.9 (37-*C*), 83.9 (q, $^2J_{C-F}$ 27.0, $C(OCH_3)(CF_3)Ph$), 63.9 (TMS$CH_2\mathit{C}H_2$), 54.6 ($O\mathit{C}H_3$), 53.9 (28-*C*), 52.4 ($CO_2\mathit{C}H_3$), 26.4 (29-*C*), 17.5 (TMS$\mathit{C}H_2CH_2$), −1.6 ($Si(\mathit{C}H_3)_3$); δ_F (376 MHz, $CDCl_3$) −69.4; *m/z* (ES^+) 579 (MH^+, 100%); *m/z* HRMS (ES^+) MH^+ calculated for $C_{28}H_{34}O_6N_2F_3Si$ 579.2133, observed 579.2125.

Synthesis of pyrrolidinone 21.

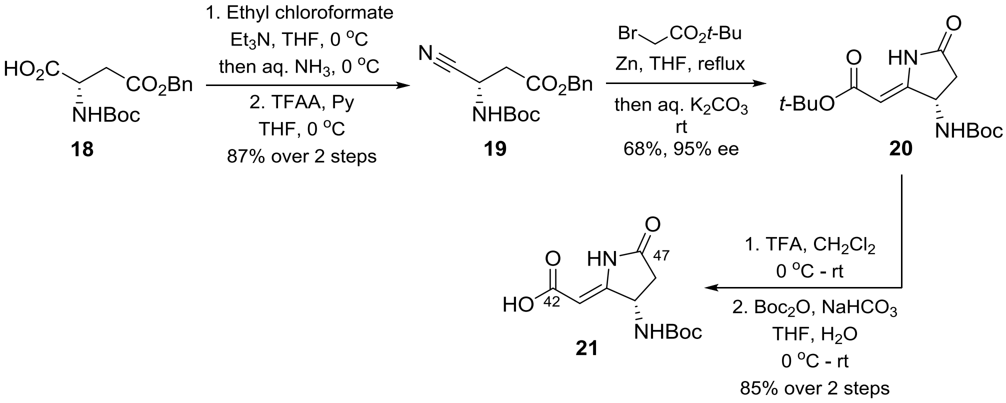

(*S*)-Benzyl 4-amino-3-((*tert*-butoxycarbonyl)amino)-4-oxobutanoate (18a)[3]

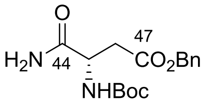

Ethyl chloroformate (0.57 mL, 6.01 mmol) was slowly added to an ice-cold solution of triethylamine (0.84 mL, 6.01 mmol) and (*S*)-4-(benzyloxy)-2-((*tert*-butoxycarbonyl)amino)-4-oxobutanoic acid (1.62 g, 5.01 mmol) in THF (25.0 mL) and the solution was stirred for 2 h at 0 °C. The resulting suspension was treated with ammonia (4.00 mL of a 35% aqueous solution) and stirred for 30 min at 0 °C. The aqueous phase was then separated and extracted with ethyl acetate, dried (Na_2SO_4), filtered and concentrated *in vacuo* to afford amide **18a** (1.62 g, quant.) as a colorless solid; R_f: 0.40 (50% EtOAc/petrol); m.p. 163-165 °C; $[\alpha]^{20}_D$ +15.4 (*c* 0.41, $CHCl_3$); ν_{max} /cm^{-1} (neat) 3344, 3033, 2978, 2360, 2341, 1676, 1499, 1455, 1366, 1249, 1161, 1049, 750, 697; δ_H (400 MHz, D_6-DMSO) 7.37-7.30 (5H, m, 5 x Ph*H*), 7.27 (1H, br s, N*H*), 7.10 (1H, br s, N*H*), 7.05 (1H, d, *J* 8.6, N*H*), 5.08 (2H, d, *J* 2.7, OCH_2Ph), 4.29 (1H, m, 45-*H*), 2.76 (1H, dd, *J* 16.1 and 5.4, 46-*H*H), 4.58 (1H, dd, *J* 16.1 and 8.8, 46-H*H*), 1.37 (9H, s, $CO_2C(CH_3)_3$); δ_C (100 MHz, D_6-DMSO) 172.6 (44- or 47-*C*), 170.3 (44- or 47-*C*), 155.2 (*C*(O)), 136.1 ($C_{(Ar)}$), 128.4 ($CH_{(Ar)}$), 127.9 ($CH_{(Ar)}$), 127.8 ($CH_{(Ar)}$), 78.2 ($CO_2C(CH_3)_3$), 65.6 (OCH_2Ph), 50.8 (45-*C*), 36.4 (46-*C*), 28.2 ($CO_2C(CH_3)_3$). *m/z* (ES^+) 323 (MH^+, 28%), 345 (MNa^+, 100); *m/z* HRMS (ES^+) MNa^+ calculated for $C_{16}H_{22}N_2O_5Na$ 345.1420, observed 345.1421. All data were in agreement with those previously reported.[3]

(*S*)-Benzyl 3-((*tert*-butoxycarbonyl)amino)-3-cyanopropanoate (19)[4]

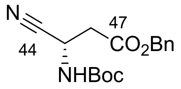

Amide **18a** (1.62 g, 5.01 mmol) was suspended in THF (25.0 mL) and pyridine (2.02 mL, 25.0 mmol) and freshly distilled trifluoroacetic anhydride (1.39 mL, 10.0 mmol) were added sequentially at 0 °C. The red solution was stirred for 2 h at 0 °C and then diluted with EtOAc. After warming to room temperature the solution was washed with aqueous saturated $NaHCO_3$ (x 2), 1 M HCl and brine solutions and then dried (Na_2SO_4), filtered and concentrated *in vacuo*. Purification by flash column chromatography (20% EtOAc/petrol) afforded nitrile **19** (1.33 g, 87%) as a pale yellow oil; R_f: 0.30 (20% EtOAc/petrol); $[\alpha]^{20}_D$ –22.6 (*c* 1.58, $CHCl_3$); ν_{max}/cm^{-1} (neat) 3351, 3034, 2979, 2360, 2341, 1719, 1499, 1456, 1368, 1251, 1161, 1049, 750, 698; δ_H (400 MHz, $CDCl_3$) 7.38-7.32 (5H, m, 5 x Ph*H*), 5.86 (1H, d, *J* 8.8, N*H*), 5.17 (2H, s, OC*H*$_2$Ph), 4.94 (1H, br s, 45-*H*), 2.91 (1H, dd, *J* 17.1 and 5.6, 46-*H*H), 2.85 (1H, dd, *J* 17.1 and 6.1, 46-H*H*), 1.45 (9H, s, $CO_2C(CH_3)_3$); δ_C (100 MHz, $CDCl_3$) 168.8 (47-*C*), 154.0 (*C*(O)), 134.7 ($C_{(Ar)}$), 128.4 ($CH_{(Ar)}$), 128.3 ($CH_{(Ar)}$), 128.1 ($CH_{(Ar)}$), 117.7 (44-*C*), 81.0 ($CO_2C(CH_3)_3$), 67.1 (O*C*H$_2$Ph), 38.2 (45-*C*), 37.1 (46-*C*), 27.9 ($CO_2C(CH_3)_3$).); *m/z* (ES^+) 327 (MNa^+, 94%), 631 (M_2Na^+, 100); *m/z* HRMS (ES^+) MNa^+ calculated for $C_{16}H_{20}N_2O_4Na$ 327.1315, observed 327.1320. All data were in agreement with those previously reported.[4]

(*S*,*Z*)-*tert*-Butyl 2-(3-((*tert*-butoxycarbonyl)amino)-5-oxopyrrolidin-2-ylidene)acetate (20)

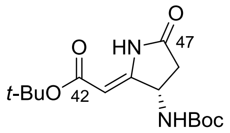

Activated zinc was prepared by washing zinc powder with 3 M HCl, water, ethanol and diethyl ether. After drying under reduced pressure, activated zinc powder (3.22 g, 49.3 mmol) was suspended in THF (43.0 mL) and heated to reflux. Following the reported procedure by Kishi,[5] four drops of *tert*-butyl bromoacetate and two drops of 1,2-dibromoethane were added and the mixture was stirred for 15 min. After addition of nitrile **19** (3.00 g, 9.86 mmol), dissolved in THF (21.0 mL)), *tert*-butyl bromoacetate (5.82 mL, 39.4 mmol), dissolved in

THF (21.0 mL)), was added over a 40 min period *via* syringe pump. The black colored reaction mixture was stirred for further 10 min at reflux and then cooled to room temperature. After the addition of THF (43.0 mL) and K_2CO_3 (14.0 mL of a 50% aqueous solution) the mixture was vigorously stirred for 15 min, filtered over Celite and washed with diethyl ether. The solution was then dried (Na_2SO_4), filtered and concentrated *in vacuo*. Purification by flash column chromatography (15-20% EtOAc/petrol) afforded pyrrolidinone **20** (2.08 g, 68%) as a colorless foam; R_f: 0.25 (20% EtOAc/petrol); $[\alpha]^{20}_{D}$ –66.0 (*c* 0.62, $CHCl_3$); ν_{max} /cm^{-1} (neat) 3340, 2978, 2933, 2360, 2253, 1643, 1516, 1456, 1269, 1163, 1136, 1052, 1026, 730, 648; δ_H (400 MHz, $CDCl_3$) 9.74 (1H, s, N*H*), 5.10 (1H, d, *J* 1.5, 43-*H*), 5.08 (1H, d, *J* 8.6, N*H*), 4.94 (1H, m, 45-*H*), 2.90 (1H, dd, *J* 17.9 and 9.1, 46-*H*H), 2.37 (1H, dd, *J* 17.9 and 5.9, 46-H*H*), 1.46 (9H, s, $CO_2C(CH_3)_3$), 1.45 (9H, s, $CO_2C(CH_3)_3$); δ_C (100 MHz, $CDCl_3$) 174.0 (47-*C*), 167.4 (42-*C*), 156.6 (44-*C*), 155.0 (*C*(O)), 92.5 (43-*C*), 80.7 (2 x $CO_2C(CH_3)_3$), 48.7 (45-*C*), 36.4 (46-*C*), 28.2 (2 x $CO_2C(CH_3)_3$). *m/z* (ES$^+$) 335 (MNa$^+$, 100); *m/z* HRMS (ES$^+$) MNa$^+$ calculated for $C_{15}H_{24}N_2O_5Na$ 335.1577, observed 335.1583; Assay of enantiomeric excess (conditions: 90:10 heptane/*i*-PrOH, 1.2 mL/min.): 95% ee (t_R (minor) = 14.0 min, t_R (major) = 18.1 min.). The *Z*-geometry of **20** was shown by nOe correlations between the C43 C-H and the C45 C-H.

(*S,Z*)-2-(3-((*tert*-Butoxycarbonyl)amino)-5-oxopyrrolidin-2-ylidene)acetic acid (21)

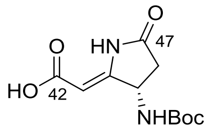

Trifluoroacetic acid (5.00 mL, 65.3 mmol) was added to a solution of (*S,Z*)-*tert*-butyl 2-(3-((*tert*-butoxycarbonyl)amino)-5-oxopyrrolidin-2-ylidene)acetate **20** (800 mg, 2.56 mmol) in CH_2Cl_2 (25.0 mL) at room temperature. The reaction was stirred for 4 h. The solution was concentrated *in vacuo* and the residue then dissolved in toluene and again concentrated *in vacuo* (x 3) to afford the corresponding free amino acid salt as an orange foam. The amino acid was subsequently dissolved in THF/H_2O (18.0 mL, 1:1) and $NaHCO_3$ (1.20 g, 14.3 mmol) and di-*tert*-butyl dicarbonate (1.20 mL, 5.12 mmol) were added at 0 ^oC. The mixture was warmed to room temperature and stirred for 48 h. The solution was acidified to pH 2-3 with aqueous HCl solution (1 M) and then diluted with EtOAc. The aqueous layer was separated and extracted with EtOAc, dried (Na_2SO_4), filtered and

concentrated *in vacuo* to afford acid **21** (557 mg, 85% over 2 steps) as a colorless solid; R_f: 0.25 (5% MeOH/EtOAc); m.p. 132-134 °C; $[\alpha]^{20}_D$ –123.2 (*c* 1.25, EtOAc); ν_{max}/cm^{-1} (neat) 3358, 3247, 2980, 1677, 1521, 1445, 1390, 1366, 1330, 1252, 1153, 1052, 947, 819, 779, 721, 667, 630; δ_H (400 MHz, D_6-DMSO) 9.96 (1H, s, N*H*), 7.48 (1H, d, *J* 8.3, N*H*), 4.88 (1H, s, 43-*H*), 4.78 (1H, m, 45-*H*), 2.74 (1H, dd, *J* 17.4 and 9.5, 46-*H*H), 2.33 (1H, dd, *J* 17.4 and 5.9, 46-H*H*), 1.40 (9H, s, $CO_2C(CH_3)_3$); δ_C (100 MHz, D_6-DMSO) 174.6 (47-*C*), 168.6 (42-*C*), 160.0 (44-*C*), 155.2 (*C*(O)), 89.0 (43-*C*), 78.7 ($CO_2C(CH_3)_3$), 48.3 (45-*C*), 34.4 (46-*C*), 28.1 ($CO_2C(CH_3)_3$). *m/z* (ES^-) 255 (M–H, 100%); *m/z* HRMS (ES^+) MNa^+ calculated for $C_{11}H_{16}N_2O_5Na$ 279.0951, observed 279.0957.

The enantiopurity of amino acid 21 was confirmed by chiral HPLC analysis of the corresponding methyl ester:

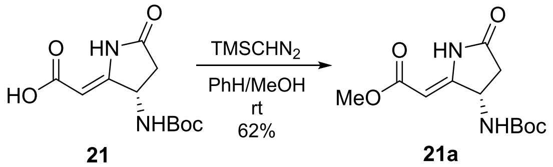

(*S*,*Z*)-Methyl 2-(3-((*tert*-butoxycarbonyl)amino)-5-oxopyrrolidin-2-ylidene)acetate (21a)

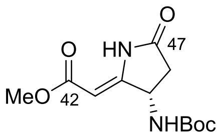

Carboxylic acid **21** (3.00 mg, 12.0 µmol) was dissolved in toluene (0.20 mL) and methanol (0.05 mL) and treated with trimethylsilyl diazomethane (7.00 µL of a 2 M solution in hexanes, 14.4 µmol). After stirring the mixture for 15 mins acetic acid (1.00 mL of 2 M aqueous solution) was added. The aqueous layer was separated and extracted with EtOAc, dried (Na_2SO_4), filtered and concentrated *in vacuo*. Purification by flash column chromatography (30-50% EtOAc/petrol) gave methyl ester **21a** (2.00 mg, 62%) as a colorless solid; R_f: 0.50 (50% EtOAc/petrol); ν_{max} /cm^{-1} (neat) 3361, 3281, 2981, 2939, 1762, 1685, 1655; δ_H (400 MHz, $CDCl_3$**)** 9.70 (1H, s, N*H*), 5.27 (1H, d, *J* 5.9, N*H*), 5.16 (1H, s, 43-*H*), 4.96-4.86 (1H, m, 45-*H*), 3.68 (3H, s, CO_2CH_3), 2.86 (1H, dd, *J* 17.9 and 9.3, 46-*H*H), 2.39 (1H, dd, *J* 17.9 and 5.4, 46-H*H*), 1.41 (9H, s, $CO_2C(CH_3)_3$); δ_C (100 MHz, $CDCl_3$**)** 174.3 (47-*C*), 168.4 (42-*C*), 158.1 (44-*C*), 155.2 (*C*(O)), 90.3 (43-*C*), 80.8 ($CO_2C(CH_3)_3$), 51.5

(CO_2CH_3), 48.8 (45-*C*), 36.1 (46-*C*), 28.3 ($CO_2C(CH_3)_3$); *m/z* HRMS (ES^+) MNa^+ calculated for $C_{12}H_{18}N_2O_5Na$ 293.1108, observed 293.1109; Assay of enantiomeric excess (conditions: 80:20 heptane/*i*-PrOH, 1.3 mL/min.): 94% ee (99% conservation of ee) (t_R (minor) = 9.5 min, t_R (major) = 15.8 min.).

Synthesis of the originally proposed structure of dehydromicrosclerodermin B 31.

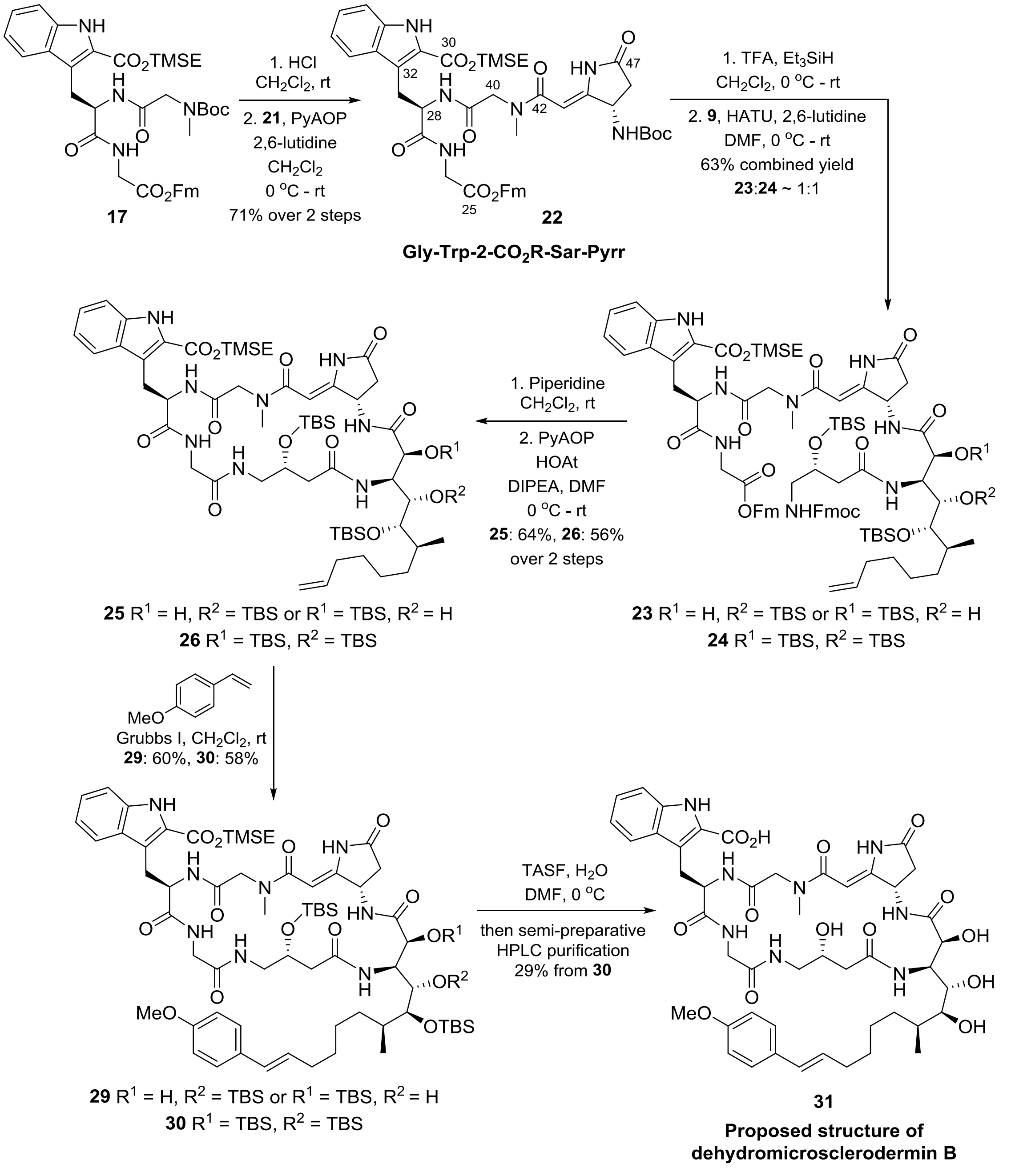

2-(Trimethylsilyl)ethyl 3-((*R*)-3-((2-((9*H*-fluoren-9-yl)methoxy)-2-oxoethyl)amino)-2-(2-((*Z*)-2-((*S*)-3-((*tert*-butoxycarbonyl)amino)-5-oxopyrrolidin-2-ylidene)-*N*-methylacetamido)acetamido)-3-oxopropyl)-1*H*-indole-2-carboxylate (22)

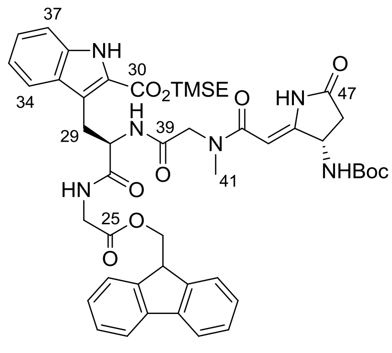

Hydrogen chloride (0.58 mL of a 4 M solution in dioxane, 2.33 mmol) was added to a solution of protected tripeptide **17** (117 mg, 0.16 mmol) in CH_2Cl_2 (1.82 mL) at room temperature. The mixture was stirred for 4 h, concentrated *in vacuo* and the residue then dissolved in toluene and again concentrated *in vacuo* (x 2) to afford the corresponding amine salt, as an amorphous colorless solid, which was used directly in the subsequent step. Carboxylic acid **21** (47.8 mg, 0.19 mmol) was dissolved in CH_2Cl_2 (1.00 mL) and cooled to 0 ^{o}C. 2,6-Lutidine (72.0 μL, 0.62 mmol) and PyAOP (97.5 mg, 0.19 mmol) were then added sequentially. The mixture was stirred at 0 ^{o}C for 2 h. The amine salt was dissolved in CH_2Cl_2 (1.10 mL) and added to the solution. The mixture was warmed slowly to room temperature, stirred for 16 h and then concentrated *in vacuo*. Purification by flash column chromatography (60-70-80-100% EtOAc/petrol) afforded tetrapeptide **22** (98.4 mg, 71% over 2 steps) as a colorless solid; R_f: 0.30 (80% EtOAc/petrol); m.p. 124-126 ^{o}C; $[\alpha]^{20}_{D}$ −20.1 (*c* 1.00, acetone); ν_{max}/cm^{-1} (neat) 3279, 2951, 1745, 1654, 1515, 1451, 1367, 1333, 1248, 1168, 1130, 1058, 943, 839, 759, 741; δ_H (500 MHz, D_6-DMSO (* denotes major rotamer)) 11.52 (1H, s, N*H*), 10.35 and 10.24* (1H, br s, N*H*), 8.37 (1H, t, *J* 5.4, N*H*), 8.17 and 8.02* (1H, d, *J* 7.3, N*H*), 7.89 (2H, d, *J* 7.6, 2 x Ar*H*), 7.79 (1H, d, *J* 7.7, 34-*H*), 7.69 (2H, d, *J* 7.4, 2 x Ar*H*), 7.48 (1H, d, *J* 7.3, N*H*), 7.43-7.32 (5H, m, 37-*H* and 4 x Ar*H*), 7.22 (1H, dd, *J* 7.4, 36-*H*), 7.05 (1H, dd, *J* 7.7, 35-*H*), 5.30* and 5.16 (1H, s, 43-*H*), 4.78-4.69 (2H, m, 28- and 45-*H*), 4.40-4.34 (4H, m, OCH_2CH and TMSCH_2CH_2), 4.25 (1H, t, *J* 7.1, OCH_2C*H*), 4.02 (1H, d, *J* 16.4, 40-*H*H), 3.92-3.83 (2H, m, 26-*H*), 3.74-3.53 (2H, m, 40-H*H* and 29-*H*H), 3.26 (1H, m, 29-H*H*), 2.73 (1H, m, 46-*H*H), 2.56* and 2.38 (3H, br s, 41-*H*), 2.29 (1H, m, 46-H*H*), 1.40* and 1.32 (9H, s, $CO_2C(CH_3)_3$), 1.16-1.13 (2H, m, TMSCH_2CH_2), 0.04 (9H, s, Si(CH_3)$_3$); δ_C (126 MHz,

D_6-DMSO (chemical shifts reported for the major rotamer only)) 174.5 (47-*C*), 171.6 (27-*C*), 169.6 (25-*C*), 167.8 (42- or 39-*C*), 167.5 (42- or 39-*C*), 162.3 (30-*C*), 157.8 (*C*(O)), 155.4 (44-*C*), 143.9 ($C_{(Ar)}$), 140.7 ($C_{(Ar)}$), 136.1 ($C_{(Ar)}$), 127.8 ($CH_{(Ar)}$), 127.7 ($C_{(Ar)}$), 127.2 ($CH_{(Ar)}$), 125.3 ($CH_{(Ar)}$), 124.6 (36-*C*), 124.2 ($C_{(Ar)}$), 120.7 (34-*C*), 120.2 ($CH_{(Ar)}$), 119.4 (35-*C*), 118.5 ($C_{(Ar)}$), 112.3 (37-*C*), 87.2 (43-*C*), 78.6 ($CO_2C(CH_3)_3$), 66.2 (O*C*H_2CH), 62.4 (TMSCH_2*C*H_2), 53.7 (28-*C*), 49.8 (40-*C*), 48.4 (45-*C*), 46.1 (OCH_2*C*H), 40.9 (26-*C*), 35.5 (41-*C*), 34.3 (46-*C*), 28.1 ($CO_2C(CH_3)_3$), 27.4 (29-*C*), 17.2 (TMS*C*H_2CH_2), −1.6 (Si(CH_3)$_3$); δ_H (500 MHz, D_6-DMSO, 363 K) 11.19 (1H, s, N*H*), 10.22 (1H, s, N*H*), 7.97 (1H, m, N*H*), 7.86 (2H, d, *J* 7.6, 2 x Ar*H*), 7.78 (1H, d, *J* 7.9, 34-*H*), 7.67 (2H, d, *J* 7.6, 2 x Ar*H*), 7.67 (1H, m, N*H*), 7.43-7.40 (3H, m, 2 x Ar*H* and 37-*H*), 7.34 (2H, dd, *J* 7.6, 2 x Ar*H*), 7.23 (1H, dd, *J* 7.9, 36-*H*), 7.07 (1H, m, N*H*), 7.06 (1H, dd, *J* 7.9, 35-*H*), 5.32 (1H, s, 43-*H*), 4.73-4.72 (2H, m, 28- and 45-*H*), 4.45-4.38 (4H, m, OCH_2CH and TMSCH_2CH_2), 4.26 (1H, dd, *J* 6.9, OCH_2C*H*), 3.96-3.88 (3H, m, 40-*H*H and 26-*H*), 3.71 (1H, d, *J* 16.4, 40-H*H*), 3.56 (1H, dd, *J* 13.9 and 5.4, 29-*H*H), 3.36 (1H, dd, *J* 13.9 and 9.1, 29-H*H*), 2.72 (1H, m, 46-*H*H), 2.65 (3H, s, 41-*H*), 2.34 (1H, dd, *J* 17.7 and 5.7, 46-H*H*), 1.40 (9H, s, $CO_2C(CH_3)_3$), 1.20-1.17 (2H, m, TMSCH_2CH_2), 0.08 (9H, s, Si(CH_3)$_3$); *m/z* (ES^+) 893 (MH^+, 100%); *m/z* HRMS (ES^+) MH^+ calculated for $C_{47}H_{57}O_{10}N_6Si$ 893.3899, observed 893.3869.

2-(Trimethylsilyl)ethyl 3-((*R*)-3-((2-((9*H*-fluoren-9-yl)methoxy)-2-oxoethyl)amino)-2-(2-((*Z*)-2-((*S*)-3-((2*S*,3*R*,4*S*,5*S*,6*S*)-3-((*R*)-4-((((9*H*-fluoren-9-yl)methoxy)carbonyl)amino)-3-((*tert*-butyldimethylsilyl)oxy)butanamido)-2,5-bis((*tert*-butyldimethylsilyl)oxy)-4-hydroxy-6-methyldodec-11-enamido)-5-oxopyrrolidin-2-ylidene)-*N*-methylacetamido)acetamido)-3-oxopropyl)-1*H*-indole-2-carboxylate (23)

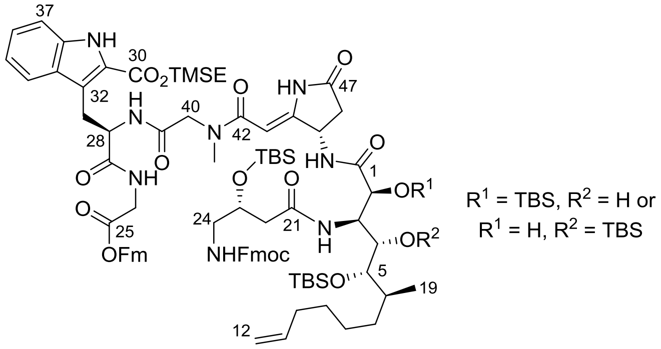

Trifluoroacetic acid (0.32 mL, 4.18 mmol) and triethylsilane (0.14 mL, 0.88 mmol) were added sequentially to a solution of tetrapeptide **22** (382 mg, 0.43 mmol) in CH_2Cl_2 (4.30 mL)

at 0 °C. The reaction was warmed to room temperature and stirred for 2 h. The solution was then concentrated *in vacuo* and the residue then dissolved in toluene and again concentrated *in vacuo* (x 2) to afford the corresponding amine salt, as a colorless solid, which was used directly in the subsequent step. HATU (137 mg, 0.36 mmol), 2,6-lutidine (0.13 mL, 1.11 mmol) and the amine salt of the deprotected tetrapeptide (328 mg, 0.36 mmol) were added sequentially to a solution of carboxylic acid **9** (293 mg, 0.28 mmol) in DMF (2.80 mL) at 0 °C. The mixture was warmed to room temperature and stirred for 16 h. The solution was then diluted with EtOAc and washed with brine solution (x 2), dried (Na_2SO_4), filtered and concentrated *in vacuo*. Purification by flash column chromatography (40-50-70% EtOAc/petrol) afforded linear hexapeptides **23** (160 mg, 34% from acid **9**) and **24** (148 mg, 29% from acid **9**) as colorless solids. Data for the linear hexapeptide **23**: R_f 0.50 (70% EtOAc/petrol); m.p. 91-93 °C; $[\alpha]^{20}_{D}$ −15.4 (*c* 1.08, EtOAc); ν_{max} /cm^{-1} (neat) 3324, 2927, 2855, 1659, 1528, 1470, 1323, 1249, 1196, 1096, 941, 835, 777, 741, 671; δ_H (500 MHz, D_6-DMSO) 11.51 (1H, s, N*H*), 10.34 (1H, s, N*H*), 8.35 (2H, br s, 2 x N*H*), 8.03 (1H, d, *J* 7.9, N*H*), 7.87 (4H, dd, *J* 7.4, 4 x Ar*H*), 7.78 (1H, d, *J* 7.9, 34-*H*), 7.67 (4H, d, *J* 7.4, 4 x Ar*H*), 7.42-7.37 (5H, m, 37-*H* and 4 x Ar*H*), 7.34-7.27 (4H, m, 4 x Ar*H*), 7.21 (1H, dd, *J* 7.4, 36-*H*), 7.14 (1H, m, N*H*), 7.04 (1H, dd, *J* 7.4, 35-*H*), 6.63 (1H, br s, O*H*), 5.73 (1H, m, 11-*H*), 5.60 (1H, s, 43-*H*), 5.33 (1H, d, *J* 6.3, N*H*), 5.03 (1H, m, 45-*H*), 4.96-4.88 (2H, m, 12-*H*), 4.66 (1H, m, 28-*H*), 4.39-4.23 (7H, m, 2-*H*, OCH_2CH, OC*H*HCH, OCH_2CH and $TMSCH_2CH_2$), 4.19-4.14 (3H, m, 3-*H*, OCH*H*CH and OCH_2CH), 4.05-3.81 (5H, m, 40-*H*H, 26-, 23- and 4-*H*), 3.67 (1H, d, *J* 16.1, 40-H*H*), 3.53 (1H, m, 29-*H*H), 3.32-3.24 (2H, m, 29-H*H* and 5-*H*), 3.07 (1H, m, 24-*H*H), 2.96 (1H, m, 24-H*H*), 2.71 (3H, s, 41-*H*), 2.63 (1H, dd, *J* 17.5 and 10.1, 46-*H*H), 2.30-2.15 (3H, m, 46-H*H* and 22-*H*), 1.99-1.94 (2H, m, 10-*H*), 1.65-1.56 (2H, m, 6-*H* and 7-*H*H), 1.30-1.23 (3H, m, 8-*H*H and 9-*H*), 1.16-1.12 (2H, m, $TMSCH_2CH_2$), 1.06 (1H, m, 8-H*H*), 0.87-0.79 (31H, m, 3 x $SiC(CH_3)_3$, 19-*H* and 7-H*H*), 0.13-−0.02 (27H, m, 6 x $SiCH_3$ and $Si(CH_3)_3$); δ_C (126 MHz, D_6-DMSO) 174.8 (47-*C*), 172.7 (1-*C*), 171.5 (27-*C*), 169.6 (25-*C*), 168.2 (21-*C*), 167.8 (42-*C*), 167.7 (39-*C*), 162.0 (30-*C*), 156.7 (44-*C*), 156.3 (*C*(O)), 143.9 ($C_{(Ar)}$), 143.9 ($C_{(Ar)}$), 143.5 (2 x $C_{(Ar)}$), 140.7 (4 x $C_{(Ar)}$), 138.7 (11-*C*), 136.1 (38-*C*), 127.8 ($CH_{(Ar)}$), 127.7 (33-*C*), 127.6 ($CH_{(Ar)}$), 127.2 ($CH_{(Ar)}$), 127.0 ($CH_{(Ar)}$), 125.2 ($CH_{(Ar)}$), 125.2 ($CH_{(Ar)}$), 124.6 (36-*C*), 124.2 (32-*C*), 120.7 (34-*C*), 120.2 ($CH_{(Ar)}$), 120.1 ($CH_{(Ar)}$), 119.4 (35-*C*), 118.4 (31-*C*), 114.6 (12-*C*), 112.3 (37-*C*), 88.5 (43-*C*), 78.1 (5-*C*), 72.8 (4-*C*), 69.9 (3-*C*), 68.2 (23-*C*), 66.2 (OCH_2CH), 65.5 (OCH_2CH), 62.4 ($TMSCH_2CH_2$), 53.8 (28-*C*), 52.9 (2-*C*), 49.7 (40-*C*), 46.7 (45-*C* and OCH_2CH), 46.1 (OCH_2CH), 45.6 (24-*C*), 42.1 (22-*C*), 40.9 (26-*C*), 35.7 (41-*C*), 35.1 (46-*C*), 34.7 (6-*C*), 33.2

(10-*C*), 32.9 (7-*C*), 28.5 (9-*C*), 27.6 (29-*C*), 26.1 (SiC(*C*H_3)$_3$), 25.9 (SiC(*C*H_3)$_3$), 25.7 (SiC(*C*H_3)$_3$), 25.4 (8-*C*), 18.0 (Si*C*(CH_3)$_3$), 17.9 (Si*C*(CH_3)$_3$), 17.7 (Si*C*(CH_3)$_3$), 17.2 (TMS*C*H_2CH_2), 16.2 (19-*C*), −1.5 (Si(*C*H_3)$_3$), −3.1 (Si*C*H_3), −3.3 (Si*C*H_3), −4.9 (Si*C*H_3), −4.9 (Si*C*H_3), −4.9 (Si*C*H_3), −5.2 (Si*C*H_3); *m/z* (ES^+) 1717 (MH^+, 100); *m/z* HRMS (ES^+) MNa^+ calculated for $C_{92}H_{130}N_8O_{16}Si_4Na$ 1737.8533, observed 1737.8532.

2-(Trimethylsilyl)ethyl 3-((*R*)-3-((2-((9*H*-fluoren-9-yl)methoxy)-2-oxoethyl)amino)-2-(2-((*Z*)-2-((*S*)-3-((2*S*,3*R*,4*S*,5*S*,6*S*)-3-((*R*)-4-((((9*H*-fluoren-9-yl)methoxy)carbonyl)amino)-3-((*tert*-butyldimethylsilyl)oxy)butanamido)-2,4,5-tris((*tert*-butyldimethylsilyl)oxy)-6-methyldodec-11-enamido)-5-oxopyrrolidin-2-ylidene)-*N*-methylacetamido)acetamido)-3-oxopropyl)-1*H*-indole-2-carboxylate (24)

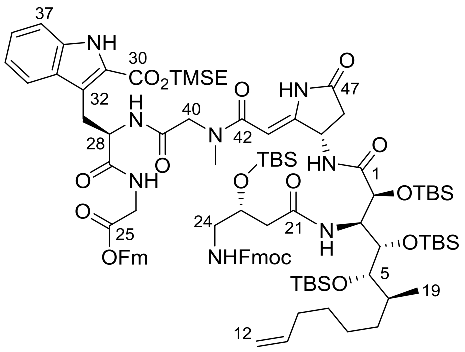

R_f 0.60 (70% EtOAc/petrol); m.p. 138-140 °C; $[\alpha]^{20}_D$ −9.4 (*c* 0.83, EtOAc); ν_{max} /cm^{-1} (neat) 2951, 2931, 1739, 1661, 1523, 1450, 1365, 1251, 1230, 1096, 834, 777, 741, 620; δ_H (500 MHz, D_6-DMSO) 11.51 (1H, s, N*H*), 10.39 (1H, s, N*H*), 8.38 (1H, br s, N*H*), 8.03 (1H, d, *J* 8.2, N*H*), 7.87 (4H, dd, *J* 11.4 and 7.6, 4 x Ar*H*), 7.79 (1H, d, *J* 8.0, 34-*H*), 7.68-7.67 (3H, m, N*H* and 2 x Ar*H*), 7.63 (1H, d, *J* 7.6, Ar*H*), 7.57 (1H, d, *J* 7.6, Ar*H*), 7.42-7.25 (11H, m, 37-*H*, 2 x N*H* and 8 x Ar*H*), 7.19 (1H, dd, *J* 7.3, 36-*H*), 7.03 (1H, dd, *J* 7.3, 35-*H*), 5.75 (1H, m, 11-*H*), 5.42 (1H, s, 43-*H*), 5.09 (1H, br s, 45-*H*), 4.97-4.90 (2H, m, 12-*H*), 4.67 (1H, m, 28-*H*), 4.39-4.31 (5H, m, 2- or 4-*H*, OC*H*$_2$CH and TMSCH_2C*H*$_2$), 4.26-4.17 (3H, m, OC*H*HCH, OCH_2C*H* and 40-*H*H), 4.14-4.08 (3H, m, OCH*H*CH, OCH_2C*H* and 3-*H*), 4.01 (1H, m, 23-*H*), 3.86 (2H, dd, *J* 5.0, 26-*H*), 3.76 (1H, d, *J* 9.5, 4- or 2-*H*), 3.55-3.51 (2H, m, 29-*H*H and 5-*H*), 3.38 (1H, m, 40-H*H*), 3.26-3.16 (2H, m, 29-H*H* and 24-*H*H), 2.95 (1H, br s, 24-H*H*), 2.65-2.60 (4H, m, 46-*H*H and 41-*H*), 2.36-2.29 (2H, m, 46-H*H* and 22-*H*H), 2.11 (1H, d, *J* 11.8, 22-H*H*), 1.99-1.97 (2H, m, 10-*H*), 1.58-1.54 (2H, m, 6-*H* and 7-*H*H), 1.32-1.23 (3H, m, 8-*H*H and 9-*H*), 1.15-1.12 (2H, m, TMSC*H*$_2$$CH_2$), 1.05

(1H, m, 8-H*H*), 0.89-0.75 (40H, m, 4 x SiC(C*H₃*)*₃*, 19-*H* and 7-H*H*), 0.20-0.02 (33H, m, 8 x SiC*H₃* and Si(C*H₃*)*₃*); δ_C (126 MHz, D_6-DMSO) 175.0 (47-*C*), 171.5 (1- and 27-*C*), 169.6 (25-*C*), 168.3 (21-*C*), 167.7 (42- and 39-*C*), 162.0 (30-*C*), 157.0 (44-*C*), 156.7 (*C*(O)), 143.9 ($C_{(Ar)}$), 143.6 ($C_{(Ar)}$), 143.5 (2 x $C_{(Ar)}$), 140.8 ($C_{(Ar)}$), 140.7 (3 x $C_{(Ar)}$), 136.1 (11-*C*), 136.1 (38-*C*), 127.7 ($CH_{(Ar)}$), 127.5 ($CH_{(Ar)}$ and 33-*C*), 127.2 ($CH_{(Ar)}$), 126.9 ($CH_{(Ar)}$), 125.2 ($CH_{(Ar)}$), 125.1 ($CH_{(Ar)}$), 124.9 ($CH_{(Ar)}$), 124.5 (36-*C*), 124.2 (32-*C*), 120.7 (34-*C*), 120.2 ($CH_{(Ar)}$), 120.0 ($CH_{(Ar)}$), 119.4 (35-*C*), 118.5 (31-*C*), 114.7 (12-*C*), 112.3 (37-*C*), 87.7 (43-*C*), 71.8 (4- or 2-*C*), 70.3 (5-*C*), 68.3 (23-*C*), 66.2 (OCH_2CH), 65.4 (OCH_2CH), 62.3 ($TMSCH_2CH_2$), 54.0 (3-*C*), 53.7 (28-*C*), 51.7 (4- or 2-*C*), 49.7 (40-*C*), 46.9 (45-*C*), 46.5 (OCH_2CH), 46.1 (OCH_2CH), 45.7 (24-*C*), 42.2 (22-*C*), 40.8 (26-*C*), 35.4 (41-*C*), 34.6 (46-*C*), 33.3 (10- and 7-*C*), 28.5 (9-*C*), 27.6 (6- and 29-*C*), 26.2 (8-*C* and $SiC(CH_3)_3$), 26.0 ($SiC(CH_3)_3$), 25.8 ($SiC(CH_3)_3$), 25.7 ($SiC(CH_3)_3$), 18.2 (2 x $SiC(CH_3)_3$), 17.9 ($SiC(CH_3)_3$), 17.7 ($SiC(CH_3)_3$), 17.1 ($TMSCH_2CH_2$), 16.0 (19-*C*), −1.6 ($Si(CH_3)_3$), −2.5 ($SiCH_3$), −3.4 ($SiCH_3$), −4.2 ($SiCH_3$), −4.4 ($SiCH_3$), −4.8 (2 x $SiCH_3$), −4.9 ($SiCH_3$), −5.1 ($SiCH_3$); *m/z* (ES^+) 1830 (MH^+, 100), 1853 (MNa^+, 80); *m/z* HRMS (ES^+) MH^+ calculated for $C_{98}H_{145}N_8O_{16}Si_5$ 1830.9653, observed 1830.9620.

2-(Trimethylsilyl)ethyl 3-(((3a*S*,6*S*,7*R*,11*R*,18*R*,*Z*)-6,11-bis((*tert*-butyldimethylsilyl)oxy)-7-((1*S*,2*S*,3*S*)-2-((*tert*-butyldimethylsilyl)oxy)-1-hydroxy-3-methylnon-8-en-1-yl)-22-methyl-2,5,9,14,17,20,23-heptaoxo-1,2,3,3a,4,5,6,7,8,9,10,11,12,13,14,15,16,17,18,19,20,21,22,23-tetracosahydropyrrolo[2,3-*m*][1,4,7,10,15,19]hexaazacyclotricosin-18-yl)methyl)-1*H*-indole-2-carboxylate (25)

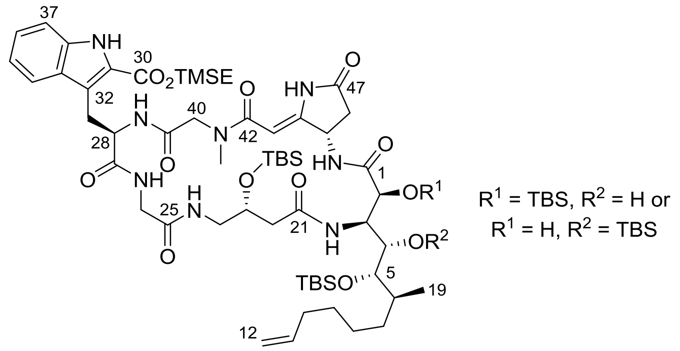

Piperidine (1.00 mL, 10.1 mmol) was added to a solution of linear hexapeptide **23** (153 mg, 89.0 µmol) in CH_2Cl_2 (15.0 mL) at room temperature. The reaction was stirred for 2 h. The solution was then concentrated *in vacuo* and the crude mixture was dried under high vacuum

for 4 h at 40 °C. Purification by flash column chromatography (2-5-25% MeOH/$CHCl_3$) afforded piperidine salt of linear amino acid as a colorless solid. The piperidine salt of amino acid was dissolved in DMF (89.0 mL) and cooled to 0 °C. PyAOP (464 mg, 0.89 mmol), HOAt (121 mg, 0.89 mmol) and *N,N*-diisopropylethylamine (0.16 mL, 0.89 mmol) were then added sequentially. The mixture was warmed slowly to room temperature, stirred for 7 days and concentrated *in vacuo*. The residue was then redissolved in 2% MeOH/$CHCl_3$, washed with H_2O, dried (Na_2SO_4), filtered and concentrated *in vacuo*. Purification by flash column chromatography (80-90% EtOAc/petrol) afforded cyclic hexapeptide **25** (74.2 mg, 64% over 2 steps) as a colorless solid; R_f 0.30 (EtOAc); m.p. 180-182 °C; $[\alpha]^{20}_D$ −44.6 (*c* 0.83, EtOAc); ν_{max} /cm^{-1} (neat) 2930, 2857, 2360, 2341, 1654, 1541, 1250, 1099, 835, 777, 746, 668; δ_H (500 MHz, D_4-MeOD (chemical shifts reported for the major rotamer only)) 7.66 (1H, d, *J* 8.2, 34-*H*), 7.41 (1H, d, *J* 8.4, 37-*H*), 7.27 (1H, dd, *J* 8.0, 36-*H*), 7.10 (1H, dd, *J* 8.0, 35-*H*), 5.78 (1H, m, 11-*H*), 5.68 (1H, s, 43-*H*), 5.04 (1H, dd, *J* 9.0 and 3.3, 45-*H*), 4.99-4.90 (3H, m, 28- and 12-*H*), 4.47 (2H, t, *J* 8.7, $TMSCH_2CH_2$), 4.40-4.34 (3H, m, 26-*H*H, 2- and 4-*H*), 4.05 (1H, d, *J* 9.5, 3-*H*), 4.00-3.95 (2H, m, 29-*H*H and 23-*H*), 3.88 (2H, d, *J* 16.2, 40-*H*), 3.61-3.55 (2H, m, 26-H*H* and 5-*H*), 3.24 (1H, dd, *J* 13.7 and 11.5, 29-H*H*), 3.21 (3H, s, 41-*H*), 3.04-2.93 (2H, m, 24-*H*), 2.84 (1H, dd, *J* 18.6 and 9.0, 46-*H*H), 2.47 (1H, dd, *J* 18.6 and 3.3, 46-H*H*), 2.36-2.26 (2H, m, 22-*H*), 2.06-2.01 (2H, m, 10-*H*), 1.72-1.66 (2H, m, 6-*H* and 7-*H*H), 1.40-1.32 (3H, m, 8-*H*H and 9-*H*), 1.24-1.17 (3H, m, $TMSCH_2CH_2$ and 8-H*H*), 1.02 (3H, d, *J* 6.6, 19-*H*), 0.98-0.92 (28H, m, 3 x $SiC(CH_3)_3$ and 7-H*H*), 0.23-0.10 (27H, m, 6 x $SiCH_3$ and $Si(CH_3)_3$); δ_C (126 MHz, D_4-MeOD (chemical shifts reported for the major rotamer only)) 178.2 (47-*C*), 176.2 (1-*C*), 173.6 (27-*C*), 172.4 (25-*C*), 171.9 (39-*C*), 171.3 (42-*C*), 171.0 (21-*C*), 164.0 (30-*C*), 158.2 (44-*C*), 140.0 (11-*C*), 137.9 (38-*C*), 129.7 (33-*C*), 126.3 (36-*C*), 125.7 (32-*C*), 121.1 (34-*C*), 121.0 (35-*C*), 120.7 (31-*C*), 115.1 (12-*C*), 113.6 (37-*C*), 91.2 (43-*C*), 81.0 (5-*C*), 74.8 (3-*C*), 71.6 (2- or 4-*C*), 70.9 (23-*C*), 64.1 ($TMSCH_2CH_2$), 56.7 (2- or 4-*C*), 55.2 (24-*C*), 55.0 (40-*C*), 54.7 (28-*C*), 49.2 (45-*C*), 45.1 (22-*C*), 42.8 (26-*C*), 38.8 (41-*C*), 36.9 (46-*C*), 36.4 (6-*C*), 35.0 (10-*C*), 34.8 (7-*C*), 30.4 (9-*C*), 27.1 (8-*C*), 27.0 ($SiC(CH_3)_3$), 26.6 ($SiC(CH_3)_3$), 26.6 ($SiC(CH_3)_3$), 26.8 (29-*C*), 19.4 ($SiC(CH_3)_3$), 19.2 ($SiC(CH_3)_3$), 19.0 ($SiC(CH_3)_3$), 18.8 ($TMSCH_2CH_2$), 17.7 (19-*C*), −1.3 ($Si(CH_3)_3$), −2.4 ($SiCH_3$), −2.4 ($SiCH_3$), −4.1 ($SiCH_3$), −4.2 ($SiCH_3$), −4.5 ($SiCH_3$), −4.9 ($SiCH_3$); *m/z* (ES^+) 1297 (MH^+, 100), 1319 (MNa^+, 80%); *m/z* HRMS (ES^+) MH^+ calculated for $C_{63}H_{109}N_8O_{13}Si_4$ 1297.7186, observed 1297.7163.

2-(Trimethylsilyl)ethyl 3-(((3a*S*,6*S*,7R,11*R*,18*R*,*Z*)-6,11-bis((*tert*-butyldimethylsilyl)oxy)-22-methyl-7-((5*S*,6*S*)-2,2,3,3,8,8,9,9-octamethyl-6-((*S*)-oct-7-en-2-yl)-4,7-dioxa-3,8-disiladecan-5-yl)-2,5,9,14,17,20,23-heptaoxo-1,2,3,3a,4,5,6,7,8,9,10,11,12,13,14,15,16,17,18,19,20,21,22,23-tetracosahydropyrrolo[2,3-*m*][1,4,7,10,15,19]hexaazacyclotricosin-18-yl)methyl)-1*H*-indole-2-carboxylate (26)

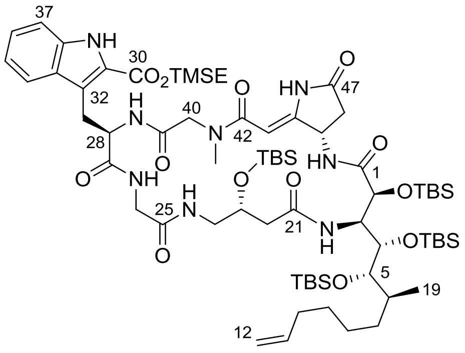

Piperidine (0.50 mL, 5.05 mmol) was added to a solution of linear hexapeptide **24** (87.0 mg, 47.5 μmol) in CH_2Cl_2 (10.0 mL) at room temperature. The reaction was stirred for 2 h. The solution was then concentrated *in vacuo* and the crude mixture was dried under high vacuum for 4 h at 40 °C. Purification by flash column chromatography (2-5-25% MeOH/$CHCl_3$) afforded piperidine salt of linear amino acid as a colorless solid. The piperidine salt of amino acid was dissolved in DMF (50.0 mL) and cooled to 0 °C. PyAOP (248 mg, 0.48 mmol), HOAt (65.0 mg, 0.48 mmol) and *N*,*N*-diisopropylethylamine (83.0 μL, 0.48 mmol) were then added sequentially. The mixture was warmed slowly to room temperature, stirred for 7 days and concentrated *in vacuo*. The residue was then redissolved in 2% MeOH/$CHCl_3$, washed with H_2O, dried (Na_2SO_4), filtered and concentrated *in vacuo*. Purification by flash column chromatography (70-80-90% EtOAc/petrol) afforded cyclic hexapeptide **26** (37.2 mg, 56% over 2 steps) as a colorless solid; R_f 0.60 (EtOAc); m.p. 188-190 °C; $[\alpha]^{20}_D$ −38.2 (*c* 2.50, EtOAc); ν_{max} /cm^{-1} (neat) 3021, 2933, 2857, 1657, 1518, 1427, 1312, 1249, 1196, 1098, 834, 777, 746, 670; δ_H (500 MHz, D_4-MeOD (chemical shifts reported for the major rotamer only)) 7.74 (1H, d, *J* 8.2, 34-*H*), 7.43 (1H, d, *J* 8.4, 37-*H*), 7.30 (1H, dd, *J* 7.9, 36-*H*), 7.12 (1H, dd, *J* 7.9, 35-*H*), 5.78 (1H, m, 11-*H*), 5.66 (1H, s, 43-*H*), 5.27 (1H, m, 45-*H*), 4.99-4.88 (2H, m, 12-*H*), 4.67 (1H, d, *J* 11.7, 28-*H*), 4.60 (1H, m, $TMSCH_2C$*H*H), 4.52 (1H, s, 2-*H*), 4.42 (1H, m, $TMSCH_2CH$*H*), 4.37-4.29 (2H, m, 26-*H*H and 3-*H*), 4.02 (1H, m, 23-*H*), 4.01 (1H, d, *J* 15.8, 40-*H*H), 3.92 (1H, d, *J* 8.2, 4-*H*), 3.77 (1H, dd, *J* 14.0 and 3.0, 29-*H*H), 3.71 (1H, d, *J* 13.6, 24-*H*H), 3.63 (1H, d, *J* 9.0, 5-*H*), 3.46-3.39 (3H, m, 29-, 26- and 40-H*H*), 3.33

(3H, s, 41-*H*), 2.86-2.75 (3H, m, 24-H*H* and 46-*H*), 2.33 (1H, m, 22-*H*H), 2.16 (1H, m, 22-H*H*), 2.05-2.01 (2H, m, 10-*H*), 1.64-1.55 (2H, m, 6-*H* and 7-*H*H), 1.40-1.32 (3H, m, 8-*H*H and 9-*H*), 1.25-1.21 (2H, m, TMSCH_2CH$_2$), 1.14 (1H, m, 8-H*H*), 1.04-0.86 (40H, m, 19-*H*, 4 x SiC(CH_3)$_3$ and 7-H*H*), 0.24-0.09 (33H, m, 8 x SiCH_3 and Si(CH_3)$_3$); δ_C (126 MHz, D_4-MeOD (chemical shifts reported for the major rotamer only)) 178.8 (47-*C*), 175.2 (1-*C*), 173.0 (27-*C*), 172.9 (39-*C*), 171.6 (42-*C*), 170.6 (21-*C*), 170.1 (25-*C*), 164.8 (30-*C*), 159.5 (44-*C*), 140.0 (11-*C*), 138.1 (38-*C*), 128.5 (33-*C*), 126.9 (36-*C*), 125.7 (32-*C*), 121.5 (34-*C*), 120.8 (31-*C*), 120.7 (35-*C*), 115.1 (12-*C*), 113.7 (37-*C*), 91.4 (43-*C*), 80.5 (5-*C*), 76.6 (4-*C*), 74.5 (2-*C*), 70.6 (23-*C*), 64.6 (TMSCH$_2$*C*H$_2$), 57.7 (3-*C*), 56.4 (28-*C*), 55.6 (40-*C*), 49.0 (45-*C*), 46.9 (24-*C*), 45.4 (22-*C*), 43.2 (26-*C*), 38.7 (41-*C*), 36.1 (46-*C*), 35.6 (6-*C*), 35.1 (7-*C*), 35.1 (10-*C*), 30.9 (9-*C*), 27.5 (8-*C*), 27.1 (29-*C*), 27.2 (SiC(*C*H$_3$)$_3$), 27.0 (SiC(*C*H$_3$)$_3$), 26.6 (2 x SiC(*C*H$_3$)$_3$), 19.5 (Si*C*(CH$_3$)$_3$), 19.3 (Si*C*(CH$_3$)$_3$), 19.1 (Si*C*(CH$_3$)$_3$), 19.0 (Si*C*(CH$_3$)$_3$), 18.9 (TMS*C*H$_2$CH$_2$), 17.9 (19-*C*), −1.3 (Si(*C*H$_3$)$_3$), −1.9 (Si*C*H$_3$), −2.7 (Si*C*H$_3$), −3.5 (Si*C*H$_3$), −3.6 (Si*C*H$_3$), −3.6 (Si*C*H$_3$), −4.1 (Si*C*H$_3$), −4.2 (Si*C*H$_3$), −4.6 (Si*C*H$_3$); *m/z* (ES^+) 1413 (MH^+, 100), 1435 (MNa^+, 30%); *m/z* HRMS (ES^+) MH^+ calculated for $C_{69}H_{123}N_8O_{13}Si_5$ 1411.8050, observed 1411.8053.

2-(Trimethylsilyl)ethyl 3-(((3a*S*,6*S*,7*R*,11*R*,18*R*,*Z*)-6,11-bis((*tert*-butyldimethylsilyl)oxy)-7-((1*S*,2*S*,3*S*,*E*)-2-((*tert*-butyldimethylsilyl)oxy)-1-hydroxy-9-(4-methoxyphenyl)-3-methylnon-8-en-1-yl)-22-methyl-2,5,9,14,17,20,23-heptaoxo-1,2,3,3a,4,5,6,7,8,9,10,11,12,13,14,15,16,17,18,19,20,21,22,23-tetracosahydropyrrolo[2,3-*m*][1,4,7,10,15,19]hexaazacyclotricosin-18-yl)methyl)-1*H*-indole-2-carboxylate (29)

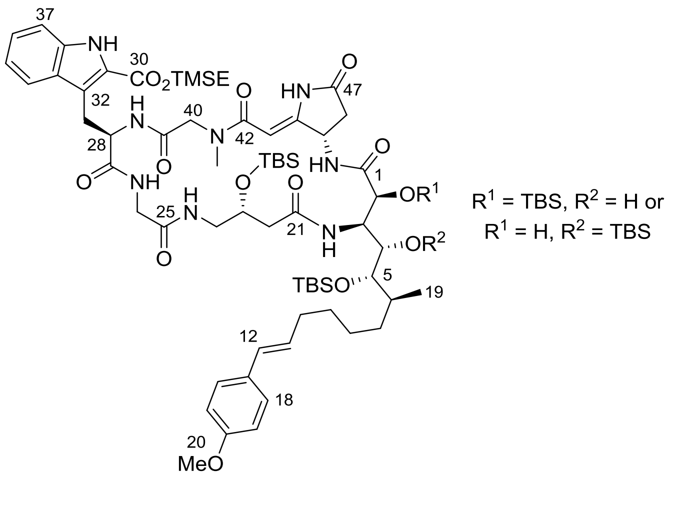

Alkene **25** (64.5 mg, 49.7 μmol) and 4-vinylanisole (70.0 μL, 0.50 mmol) were added to dry degassed CH_2Cl_2 (1.00 mL). Grubbs I catalyst (80.0 mg, 0.10 mmol) was then added to the solution and the reaction was stirred at room temperature for a total period of 48 h. One additional injection of 4-vinylanisole (70.0 μL, 0.50 mmol) and Grubbs I catalyst (80.0 mg, 0.10 mmol) was made after 24 h. Afterwhich DMSO (100 μL) was added and the solution was stirred at room temperature for 10 mins. The mixture was then concentrated *in vacuo* and purified by flash column chromatography (50-80-100% EtOAc/petrol) to afford styrene **29** (41.8 mg, 60%) as a colorless solid; R_f 0.30 (EtOAc); m.p. 119-121 ^oC; $[\alpha]^{20}_D$ −27.2 (*c* 1.65, EtOAc); ν_{max} /cm^{-1} (neat) 3022, 2933, 2856, 2390, 2348, 2286, 1659, 1445, 1326, 1249, 1098, 835, 776, 749; δ_H (500 MHz, D_4-MeOD (chemical shifts reported for the major rotamer only)) 7.67 (1H, d, *J* 8.0, 34-*H*), 7.42 (1H, d, *J* 8.4, 37-*H*), 7.28-7.21 (3H, m, 36-, 14- and 18-*H*), 7.09 (1H, dd, *J* 7.6, 35-*H*), 6.82 (2H, d, *J* 8.8, 15- and 17-*H*), 6.30 (1H, d, *J* 15.8, 12-*H*), 6.05 (1H, dt, *J* 15.8 and 6.9, 11-*H*), 5.69 (1H, s, 43-*H*), 5.07 (1H, dd, *J* 8.4 and 3.5, 45-*H*), 4.92 (1H, dd, *J* 10.7 and 3.5, 28-*H*), 4.47 (2H, t, *J* 8.7, TMS$CH_2C\mathit{H}_2$), 4.40-4.24 (3H, m, 26-*H*H, 2- and 4-*H*), 4.05 (1H, d, *J* 9.0, 3-*H*), 3.99-3.95 (2H, m, 29-*H*H and 23-*H*), 3.85 (2H, d, *J* 17.2, 40-*H*), 3.76 (3H, s, 20-*H*), 3.62-3.56 (2H, m, 26-H*H* and 5-*H*), 3.24 (1H, dd, *J* 13.7 and 11.5, 29-H*H*), 3.20 (3H, s, 41-*H*), 3.06-3.01 (2H, m, 24-*H*), 2.84 (1H, dd, *J* 18.4 and 9.0, 46-*H*H), 2.46 (1H, dd, *J* 18.4 and 2.5, 46-H*H*), 2.35-2.29 (2H, m, 22-*H*), 2.17-2.13 (2H, m, 10-*H*), 1.72-1.68 (2H, m, 6-*H* and 7-*H*H), 1.45-1.38 (3H, m, 8-*H*H and 9-*H*), 1.26-1.20 (3H, m, TMS$C\mathit{H}_2CH_2$ and 8-H*H*), 1.03 (3H, d, *J* 6.5, 19-*H*), 0.97-0.86 (28H, m, 3 x SiC$(C\mathit{H}_3)_3$ and 7-H*H*), 0.23-0.09 (27H, m, 6 x SiCH_3 and Si$(C\mathit{H}_3)_3$); δ_C (126 MHz, D_4-MeOD (chemical shifts reported for the major rotamer only)) 178.1 (47-*C*), 176.1 (1-*C*), 173.7 (27-*C*), 172.4 (25-*C*), 171.9 (39-*C*), 171.4 (42-*C*), 170.9 (21-*C*), 164.0 (30-*C*), 160.4 (16-*C*), 158.2 (44-*C*), 137.9 (38-*C*), 132.1 (13-*C*), 130.9 (12-*C*), 129.8 (33-*C*), 129.4 (11-*C*), 128.3 (14- and 18-*C*), 126.3 (36-*C*), 125.8 (32-*C*), 121.2 (34-*C*), 121.0 (35-*C*), 120.6 (31-*C*), 115.0 (15- and 17-*C*), 113.6 (37-*C*), 91.2 (43-*C*), 81.1 (5-*C*), 74.9 (3-*C*), 71.6 (2-*C*), 70.9 (23-*C*), 64.1 (TMS$CH_2\mathit{C}H_2$), 56.7 (4-*C*), 55.8 (20-*C*), 55.1 (24-*C*), 55.1 (40-*C*), 54.9 (28-*C*), 49.2 (45-*C*), 45.0 (22-*C*), 42.8 (26-*C*), 38.8 (41-*C*), 37.0 (46-*C*), 36.2 (6-*C*), 34.9 (7-*C*), 34.4 (10-*C*), 31.0 (9-*C*), 27.5 (8-*C*), 27.0 (SiC$(\mathit{C}H_3)_3$), 26.7 (SiC$(\mathit{C}H_3)_3$), 26.6 (SiC$(\mathit{C}H_3)_3$), 26.8 (29-*C*), 19.4 (Si*C*$(CH_3)_3$), 19.2 (Si*C*$(CH_3)_3$), 19.0 (Si*C*$(CH_3)_3$), 18.8 (TMS$\mathit{C}H_2CH_2$), 17.8 (19-*C*), −1.3 (Si$(\mathit{C}H_3)_3$), −2.3 (Si$\mathit{C}H_3$), −3.0 (Si$\mathit{C}H_3$), −4.0 (Si$\mathit{C}H_3$), −4.2 (Si$\mathit{C}H_3$), −4.4 (Si$\mathit{C}H_3$), −4.8 (Si$\mathit{C}H_3$); *m/z* (ES^+) 1404 (MH^+, 100), 1426 (MNa^+, 30%); *m/z* HRMS (ES^+) MH^+ calculated for $C_{70}H_{115}N_8O_{14}Si_4$ 1404.7638, observed 1404.7682.

2-(Trimethylsilyl)ethyl 3-(((3a*S*,6*S*,7*R*,11*R*,18*R*,*Z*)-6,11-bis((*tert*-butyldimethylsilyl)oxy)-7-((5*S*,6*S*)-6-((*S*,*E*)-8-(4-methoxyphenyl)oct-7-en-2-yl)-2,2,3,3,8,8,9,9-octamethyl-4,7-dioxa-3,8-disiladecan-5-yl)-22-methyl-2,5,9,14,17,20,23-heptaoxo-1,2,3,3a,4,5,6,7,8,9,10,11,12,13,14,15,16,17,18,19,20,21,22,23-tetracosahydropyrrolo[2,3-*m*][1,4,7,10,15,19]hexaazacyclotricosin-18-yl)methyl)-1*H*-indole-2-carboxylate (30)

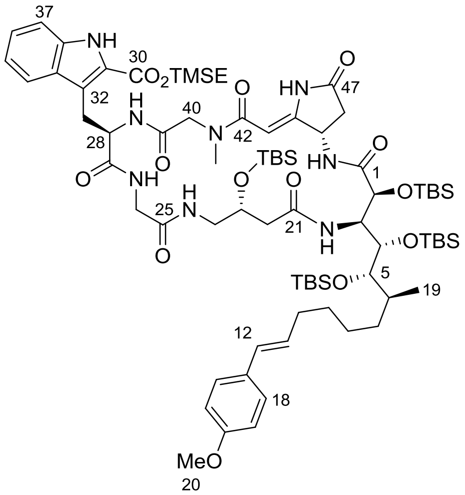

Alkene **26** (50.0 mg, 35.0 μmol) and 4-vinylanisole (45.0 μL, 0.35 mmol) were added to dry degassed CH_2Cl_2 (2.00 mL). Grubbs I catalyst (58.0 mg, 71.0 μmol) was then added to the solution and the reaction was stirred at room temperature for a total period of 48 h. One additional injection of 4-vinylanisole (45.0 μL, 0.35 mmol) and Grubbs I catalyst (58.0 mg, 71.0 μmol) was made after 24 h. Afterwhich DMSO (100 μL) was added and the solution was stirred at room temperature for 10 mins. The mixture was then concentrated *in vacuo* and purified by flash column chromatography (50-60-70-80-100% EtOAc/petrol) to afford styrene **30** (30.5 mg, 58%) as a colorless solid; R_f 0.30 (80% EtOAc/petrol); m.p. 162-164 °C; $[\alpha]^{20}_D$ −24.0 (*c* 1.53, EtOAc); ν_{max} /cm^{-1} (neat) 2953, 2929, 2856, 1657, 1511, 1316, 1250, 1097, 834, 776, 745, 673; δ_H (500 MHz, D_4-MeOD (chemical shifts reported for the major rotamer only)) 7.74 (1H, d, *J* 8.0, 34-*H*), 7.43 (1H, d, *J* 8.4, 37-*H*), 7.30 (1H, dd, *J* 7.6, 36-*H*), 7.25 (2H, d, *J* 8.7, 14- and 18-*H*), 7.12 (1H, dd, *J* 7.6, 35-*H*), 6.83 (2H, d, *J* 8.7, 15- and 17-*H*), 6.30 (1H, d, *J* 15.9, 12-*H*), 6.06 (1H, dt, *J* 15.9 and 6.9, 11-*H*), 5.65 (1H, s, 43-*H*), 5.26 (1H, m, 45-*H*), 4.68 (1H, d, *J* 11.2, 28-*H*), 4.60 (1H, m, $TMSCH_2C$*H*H), 4.52 (1H, s, 2-*H*), 4.43-4.35 (2H, m, 3-*H* and $TMSCH_2CH$*H*), 4.26 (1H, dd, *J* 10.7, 26-*H*H), 4.02-3.96 (2H, m, 23-*H* and 40-*H*H), 3.91 (1H, d, *J* 6.8, 4-*H*), 3.78 (1H, m, 29-*H*H), 3.76 (3H, s, 20-*H*), 3.71 (1H, m, 24-*H*H), 3.64 (1H, d, *J* 9.0, 5-*H*), 3.46-3.41 (3H, m,

29-, 26- and 40-H*H*), 3.31 (3H, s, 41-*H*), 2.80-2.78 (3H, m, 24-H*H* and 46-*H*), 2.34 (1H, m, 22-*H*H), 2.19-2.15 (3H, m, 22-H*H* and 10-*H*), 1.72-1.62 (2H, m, 6-*H* and 7-*H*H), 1.45-1.34 (3H, m, 8-*H*H and 9-*H*), 1.24-1.20 (3H, m, 8-H*H* and TMSCH_2CH$_2$), 1.04 (3H, d, *J* 5.4, 19-*H*), 0.99-0.86 (37H, m, 4 x SiC(CH_3)$_3$ and 7-H*H*), 0.40-0.09 (33H, m, 8 x SiCH_3 and Si(CH_3)$_3$); δ_C (126 MHz, D$_4$-MeOD (chemical shifts reported for the major rotamer only)) 178.7 (47-*C*), 175.2 (1-*C*), 173.0 (27-*C*), 172.8 (39-*C*), 171.6 (42-*C*), 170.6 (21-*C*), 170.3 (25-*C*), 164.7 (30-*C*), 160.4 (16-*C*), 159.6 (44-*C*), 138.1 (38-*C*), 132.1 (13-*C*), 131.0 (12-*C*), 129.3 (11-*C*), 128.5 (33-*C*), 128.3 (14- and 18-*C*), 126.9 (36-*C*), 125.7 (32-*C*), 121.5 (34-*C*), 120.9 (31-*C*), 120.8 (35-*C*), 115.1 (15- and 17-*C*), 113.7 (37-*C*), 91.4 (43-*C*), 80.7 (5-*C*), 76.6 (4-*C*), 74.5 (2-*C*), 70.6 (23-*C*), 64.6 (TMSCH$_2$*C*H$_2$), 57.7 (3-*C*), 56.4 (28-*C*), 55.8 (20-*C*), 55.5 (40-*C*), 49.2 (45-*C*), 47.0 (24-*C*), 45.3 (22-*C*), 43.3 (26-*C*), 38.6 (41-*C*), 36.1 (46-*C*), 35.4 (6-*C*), 35.1 (7-*C*), 34.4 (10-*C*), 30.9 (9-*C*), 27.6 (8-*C*), 27.1 (29-*C*), 27.2 (SiC(*C*H$_3$)$_3$), 27.0 (SiC(*C*H$_3$)$_3$), 26.6 (2 x SiC(*C*H$_3$)$_3$), 19.6 (Si*C*(CH$_3$)$_3$), 19.4 (Si*C*(CH$_3$)$_3$), 19.1 (Si*C*(CH$_3$)$_3$), 19.0 (Si*C*(CH$_3$)$_3$), 18.9 (TMS*C*H$_2$CH$_2$), 18.0 (19-*C*), −1.3 (Si(*C*H$_3$)$_3$), −0.7 (Si*C*H$_3$), −1.9 (Si*C*H$_3$), −2.7 (Si*C*H$_3$), −3.5 (Si*C*H$_3$), −3.6 (Si*C*H$_3$), −4.0 (Si*C*H$_3$), −4.2 (Si*C*H$_3$), −4.5 (Si*C*H$_3$); *m/z* (ES$^+$) 1517 (MH$^+$, 100); *m/z* HRMS (ES$^+$) MH$^+$ calculated for $C_{76}H_{129}N_8O_{14}Si_5$ 1517.8469, observed 1517.8535.

3-(((3a*S*,6*S*,7*R*,11*R*,18*R*,*Z*)-7-((1*S*,2*S*,3*S*,*E*)-1,2-Dihydroxy-9-(4-methoxyphenyl)-3-methylnon-8-en-1-yl)-6,11-dihydroxy-22-methyl-2,5,9,14,17,20,23-heptaoxo-1,2,3,3a,4,5,6,7,8,9,10,11,12,13,14,15,16,17,18,19,20,21,22,23-tetracosahydropyrrolo[2,3-*m*][1,4,7,10,15,19]hexaazacyclotricosin-18-yl)methyl)-1*H*-indole-2-carboxylic acid (31)

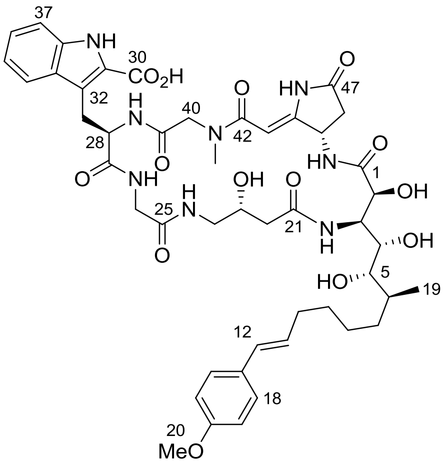

H_2O (11 μL, 0.58 mmol) and TASF (100 μL of a 2.9 M solution in DMF, 0.29 mmol) were added sequentially to a solution of protected cyclic peptide **30** (29.0 mg, 19.1 μmol) in DMF (0.50 mL) at 0 °C. The reaction was stirred for 10 h at 0 °C. Afterwhich TMS_2O (100 μL) was added and the solution was stirred at room temperature for 10 mins. The solution was then concentrated using high vacuum centrifugal evaporator. Purification by flash column chromatography (10-20-25% MeOH/$CHCl_3$) and then semi preparative reverse phase HPLC (XBridge Prep C18 5.0 μm 10 mm x 250 mm column, at 4.4 mL/min with UV detection 214 nm: 2 mins – 5% CH_3CN in 0.05% aqueous TFA, linear increase; 3 mins – 42% CH_3CN in 0.05% aqueous TFA, isocratic; 23 mins – 42% CH_3CN in 0.05% aqueous TFA, linear increase; 24 mins – 95% CH_3CN in 0.05% aqueous TFA, isocratic) afforded deprotected cyclic peptide **31** (5.3 mg, 29%) as a colorless solid; R_f 0.30 (30% MeOH/$CHCl_3$); $[\alpha]^{20}_D$ −26.4 (*c* 0.23, 0.1 M NH_4HCO_3, pH 7); ν_{max} /cm^{-1} (neat) 3299, 2926, 2853, 1651, 1537, 1511, 1339, 1246, 1201, 1177, 1132, 1025, 968, 839, 800, 745, 720, 664; δ_H (500 MHz, D_6-DMSO (chemical shifts reported for the major conformer only)) 11.44 (1H, br s, N*H*), 10.38 (1H, s, N*H*), 8.53 (1H, d, *J* 6.9, N*H*), 8.43 (1H, br s, N*H*), 7.64 (1H, d, *J* 6.0, N*H*), 7.50 (1H, d, *J* 8.2, 34-*H*), 7.47 (1H, d, *J* 9.0, N*H*), 7.36 (1H, d, *J* 8.4, 37-*H*), 7.29 (2H, d, *J* 8.7, 14- and 18-*H*), 7.18 (1H, dd, *J* 7.4, 36-*H*), 6.99 (1H, dd, *J* 7.4, 35-*H*), 6.85 (2H, d, *J* 8.7, 15- and 17-*H*), 6.30 (1H, d, *J* 15.8, 12-*H*), 6.10 (1H, dt, *J* 15.8 and 6.8, 11-*H*), 6.05 (1H, m, N*H*), 5.49 (1H, s, 43-*H*), 5.07 (1H, d, *J* 4.3, O*H*), 4.59 (1H, m, 45-*H*), 4.49 (1H, d, *J* 16.6, 40-*H*H), 4.46 (1H, d, *J* 3.8, O*H*), 4.39 (1H, s, 2-*H*), 4.17 (1H, d, *J* 9.9, O*H*), 4.07-4.04 (2H, m, 28- and 3-*H*), 3.76 (1H, m, 24-*H*H), 3.72 (3H, s, 20-*H*), 3.56-3.35 (5H, m, 26-*H*H, 29-*H*H, 40-H*H*, 23- and 4-*H*), 3.18 (1H, m, 29-H*H*), 3.07 (3H, s, 41-*H*), 3.07-3.03 (2H, m, 26-H*H* and 5-*H*), 2.66 (1H, dd, *J* 17.8 and 5.2, 46-*H*H), 2.58 (1H, dd, *J* 17.8 and 9.6, 46-H*H*), 2.50 (1H, m, 22-*H*H), 2.32 (1H, m, 24-H*H*), 2.14-2.07 (3H, m, 22-H*H* and 10-*H*), 1.73 (1H, m, 7-*H*H), 1.58 (1H, m, 6-*H*), 1.38-1.37 (3H, m, 8-*H*H and 9-*H*), 1.17 (1H, m, 8-H*H*), 1.00 (1H, m, 7-H*H*), 0.72 (3H, d, *J* 6.6, 19-*H*); δ_C (126 MHz, D_6-DMSO (chemical shifts reported for the major conformer only), 31- and 32-*C* were not identified) 175.1 (47-*C*), 173.6 (21-*C*), 172.5 (1-*C*), 172.3 (27-*C*), 169.9 (39-*C*), 168.7 (25-*C*), 167.8 (42-*C*), 163.7 (30-*C*), 158.3 (16-*C*), 157.7 (44-*C*), 135.6 (38-*C*), 130.1 (13-*C*), 128.9 (12-*C*), 128.5 (11-*C*), 127.6 (33-*C*), 126.9 (14- and 18-*C*), 124.1 (36-*C*), 120.1 (34-*C*), 119.1 (35-*C*), 113.9 (15- and 17-*C*), 112.2 (37-*C*), 86.9 (43-*C*), 72.7 (5-*C*), 69.6 (2-*C*), 68.8 (4-*C*), 68.6 (23-*C*), 56.4 (28-*C*), 55.1 (20-*C*), 53.2 (3-*C*), 49.7 (40-*C*), 48.2 (45-*C*), 45.0 (24-*C*), 42.5 (26-*C*), 41.9 (22-*C*), 37.3 (41-*C*), 34.0 (6-*C*), 32.8 (7-*C*), 32.5 (10- and 46-*C*), 29.6 (9-*C*), 26.2 (8-*C*), 26.0 (29-*C*), 15.5 (19-*C*);

m/z (ES^+) 962 (MH^+, 100), 984 (MNa^+, 30), 1923 (M_2H^+, 30), 1945 (M_2Na^+, 10); *m/z* HRMS (ES^-) M-H^- calculated for $C_{47}H_{59}N_8O_{14}$ 959.4156, observed 959.4150.

Synthesis of dipeptide 35.

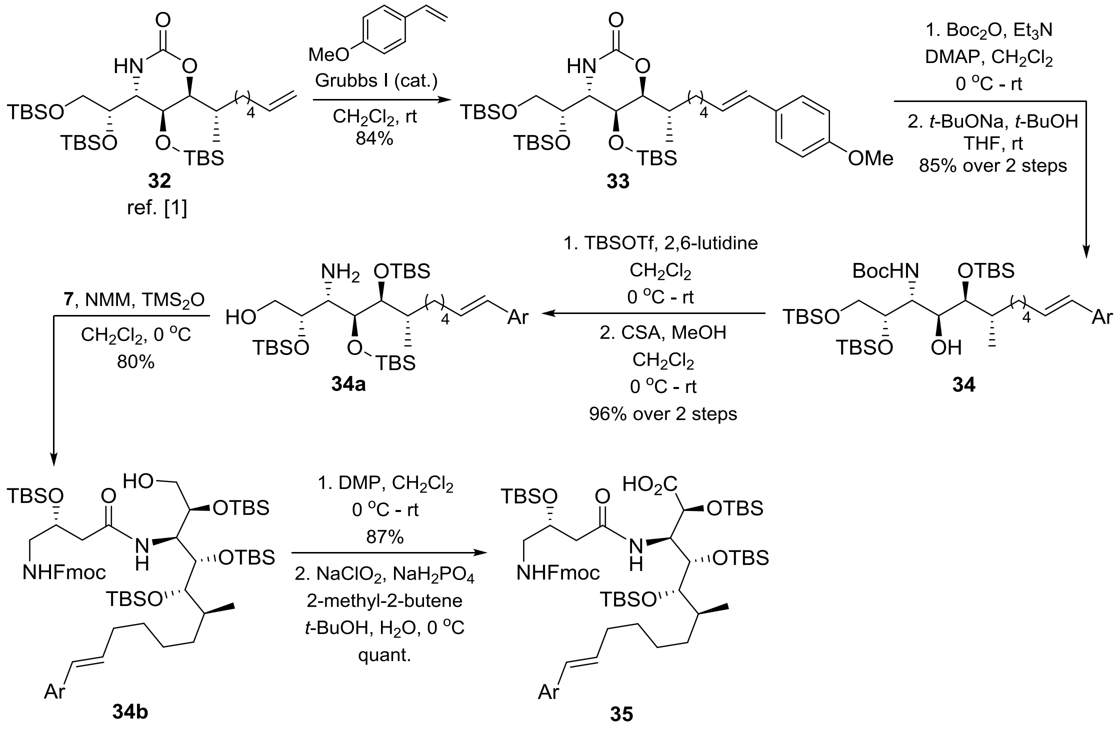

(4*S*,5*S*,6*S*)-5-((*tert*-Butyldimethylsilyl)oxy)-6-((*S*,*E*)-8-(4-methoxyphenyl)oct-7-en-2-yl)-4-((*S*)-2,2,3,3,8,8,9,9-octamethyl-4,7-dioxa-3,8-disiladecan-5-yl)-1,3-oxazinan-2-one (33)

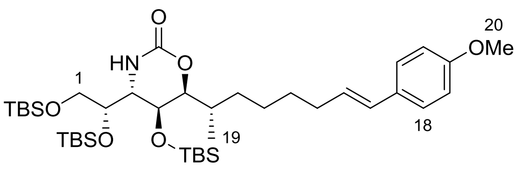

Alkene[1] **32** (300 mg, 0.48 mmol) and 4-vinylanisole (0.32 mL, 2.38 mmol) were added to dry degassed CH_2Cl_2 (1.60 mL). Grubbs I catalyst (12.0 mg, 14.3 µmol) was then added to the solution and the reaction was stirred at room temperature for a total period of 48 h. Four additional injections of Grubbs I catalyst (12.0 mg, 14.3 µmol (per injection)) were made at regular intervals over this period. Afterwhich DMSO (100 µL) was added and the solution was stirred at room temperature for 10 mins. The mixture was then concentrated *in vacuo* and purified by flash column chromatography (5-10-15% EtOAc/petrol) to afford styrene **33**

(293 mg, 84%) as a colorless solid; R_f: 0.25 (10% EtOAc/petrol); m. p. 94-96 ^{o}C; $[\alpha]^{25}_{D}$ −48.7 (*c* 1.04, $CHCl_3$); ν_{max}/cm^{-1} (neat) 2952, 2928, 2856, 1714, 1695, 1511, 1462, 1452, 1362, 1303, 1249, 1173, 1108, 1080, 926, 836, 777; δ_H (400 MHz, $CDCl_3$) 7.28 (2H, d, *J* 8.8, 14- and 18-*H*), 6.83 (2H, d, *J* 8.8, 15- and 17-*H*), 6.32 (1H, d, *J* 15.7, 12-*H*), 6.07 (1H, dt, *J* 15.7 and 6.9, 11-*H*), 5.07 (1H, d, *J* 2.9, N*H*), 4.15 (1H, d, *J* 0.7, 4-*H*), 3.91 (1H, d, *J* 9.8, 5-*H*), 3.80 (3H, s, 20-*H*), 3.63 (1H, d, *J* 6.4, 1-*H*H), 3.57-3.49 (3H, m, 1-H*H*, 2-*H* and 3-*H*), 2.22-2.17 (2H, m, 10-*H*),1.99-1.89 (2H, m, 6-*H* and 7-*H*H), 1.49-1.42 (3H, m, 9-*H* and 8-*H*H), 1.28 (1H, m, 8-H*H*), 1.18 (1H, m, 7-H*H*), 0.90-0.88 (30H, m, 19-*H* and 3 x $SiC(CH_3)_3$), 0.11, 0.10, 0.09, 0.09, 0.08, 0.08 (6 x 3H, s, $SiCH_3$); δ_C (100 MHz, $CDCl_3$) 158.5 (16-*C*), 153.9 (*C*(O)), 130.8 (13-*C*), 129.0 (12-*C*), 128.9 (11-*C*), 126.9 (14- and 18-*C*), 113.8 (15- and 17-*C*), 81.6 (5-*C*), 73.6 (2-*C*), 64.6 (1-*C*), 64.1 (4-*C*), 59.9 (3-*C*), 55.2 (20-*C*), 33.0 (10-*C*), 32.9 (6-*C*), 31.9 (7-*C*), 29.6 (9-*C*), 26.0 (8-*C*), 25.9 ($SiC(CH_3)_3$), 25.8 ($SiC(CH_3)_3$), 25.6 ($SiC(CH_3)_3$), 18.3 ($SiC(CH_3)_3$), 18.0 ($SiC(CH_3)_3$), 17.9 ($SiC(CH_3)_3$), 14.9 (19-*C*), −4.1 ($SiCH_3$), −4.2 ($SiCH_3$), −4.4 ($SiCH_3$), −4.7 ($SiCH_3$), −5.4 (2 x $SiCH_3$); *m/z* (ES$^+$) 658 (MNa$^+$, 100%); *m/z* HRMS (ES$^+$) MH$^+$ calculated for $C_{39}H_{74}NO_6Si_3$ 736.4818, observed 736.4816.

***tert*-Butyl ((5*S*,6*S*,7*R*,8*S*)-8-((*tert*-butyldimethylsilyl)oxy)-6-hydroxy-5-((*S*,*E*)-8-(4-methoxyphenyl)oct-7-en-2-yl)-2,2,3,3,11,11,12,12-octamethyl-4,10-dioxa-3,11-disilatridecan-7-yl)carbamate (34)**

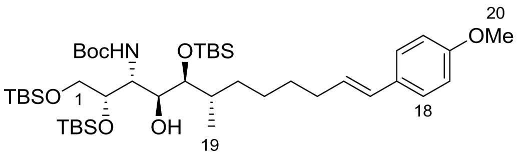

Triethylamine (2.50 ml, 18.1 mmol), di-*tert*-butyl dicarbonate (16.6 mL, 72.4 mmol) and DMAP (442 mg, 3.62 mmol) were added sequentially to a solution of carbamate **33** (2.66 g, 3.62 mmol) in CH_2Cl_2 (24.0 mL) at 0 ^{o}C. The mixture was then warmed to room temperature and stirred for a total period of 40 h. Two additional injections of di-*tert*-butyl dicarbonate (8.30 mL, 36.2 mmol) and DMAP (442 mg, 3.62 mmol) (per injection) were made at regular intervals over this period. Water was added and aqueous layer was separated and extracted with CH_2Cl_2, dried (Na_2SO_4), filtered and concentrated *in vacuo* onto silica gel. Purification by flash column chromatography (petrol-2-5% EtOAc/petrol) afforded the *N*-Boc-carbamate as a viscous colorless oil (in mixture with Boc_2O, which was inseparable from the desired

product). Sodium *tert*-butoxide (1.74 g, 18.1 mmol) was added in one portion to a stirred solution of the *N*-Boc-carbamate in *t*-BuOH/THF (72.0 mL, 1:1) at room temperature. The reaction was stirred for 2.5 h before the addition of saturated aqueous ammonium chloride solution. The aqueous layer was separated and extracted with EtOAc, dried ($MgSO_4$), filtered and concentrated *in vacuo*. Purification by flash column chromatography (1-2% EtOAc/petrol) afforded silyl ether **34** (2.49 g, 85% over 2 steps), as a viscous colorless oil; R_f: 0.40 (2% EtOAc/petrol); $[\alpha]^{25}_D$ −4.6 (*c* 1.78, $CHCl_3$); ν_{max}/cm^{-1} (neat) 3445 br, 2954, 2929, 2857, 1718, 1493, 1472, 1366, 1292, 1249, 1173, 1145, 1068, 937, 905, 834, 777, 676; δ_H (400 MHz, $CDCl_3$) 7.28 (2H, d, *J* 8.8, 14- and 18-*H*), 6.84 (2H, d, *J* 8.8, 15- and 17-*H*), 6.34 (1H, d, *J* 15.7, 12-*H*), 6.09 (1H, dt, *J* 15.7 and 6.9, 11-*H*), 4.68 (1H, d, *J* 9.8, N*H*), 4.27 (1H, t, *J* 6.9, 2-*H*), 3.85 (1H, d, *J* 3.2, 5-*H*), 3.80 (3H, s, 20-*H*), 3.60 (1H, dd, *J* 9.8, 3-*H*), 3.48 (2H, d, *J* 6.9, 1-*H*), 3.28 (1H, dd, *J* 8.8, 4-*H*), 3.17 (1H, d, *J* 8.8, O*H*), 2.23-2.18 (2H, m, 10-*H*), 1.60 (1H, m, 6-*H*), 1.49-1.44 (13H, m, $CO_2C(CH_3)_3$, 9- and 8-*H*), 1.28 (1H, m, 7-*H*H), 1.15 (1H, m, 7-H*H*), 0.94-0.91 (30H, m 3 x $SiC(CH_3)_3$ and 19-*H*), 0.25, 0.17, 0.16 and 0.13 (4 x 3H, s, $SiCH_3$), 0.06 (6H, s, 2 x $SiCH_3$); δ_C (100 MHz, $CDCl_3$) 158.6 (16-*C*), 156.6 (*C*(O)), 130.7 (13-*C*), 129.2 (12-*C*), 128.6 (11-*C*), 126.9 (14- and 18-*C*), 113.8 (15- and 17-*C*), 78.6 ($CO_2C(CH_3)_3$), 72.3 (5-*C*), 70.7 (2-*C*), 67.0 (4-*C*), 64.1 (1-*C*), 55.1 (20-*C*), 53.2 (3-*C*), 39.5 (6-*C*), 32.8 (10-*C*), 32.7 (7-*C*), 29.9 (9-*C*), 27.8 ($CO_2C(CH_3)_3$), 26.9 (8-*C*), 26.0 ($SiC(CH_3)_3$), 25.9 (2 x $SiC(CH_3)_3$), 18.4 ($SiC(CH_3)_3$), 18.2 ($SiC(CH_3)_3$), 18.1 ($SiC(CH_3)_3$), 14.6 (19-*C*), −4.3 ($SiCH_3$), −4.3 ($SiCH_3$), −5.0 ($SiCH_3$), −5.1 ($SiCH_3$), −5.5 ($SiCH_3$), −5.7 ($SiCH_3$); *m/z* (ES^+) 833 (MNa^+, 100%), 811 (MH^+, 25); *m/z* HRMS (ES^+) MNa^+ calculated for $C_{43}H_{84}NO_7Si_3$ 810.5550, observed 810.5543.

(2*S*,3*S*,4*S*,5*S*,6*S*,*E*)-3-Amino-2,4,5-tris((*tert*-butyldimethylsilyl)oxy)-12-(4-methoxyphenyl)-6-methyldodec-11-en-1-ol (34a)

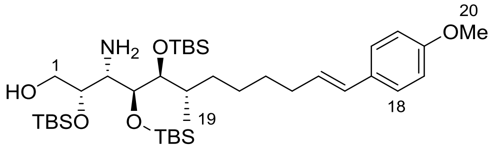

tert-Butyldimethylsilyl trifluoromethanesulfonate (7.00 mL, 30.5 mmol) was added dropwise to a solution of **34** (2.47 g, 3.05 mmol) and 2,6-lutidine (7.10 mL, 61.0 mmol) in CH_2Cl_2 (30.0 mL) at 0 °C. The mixture was warmed to room temperature and stirred for 16 h before being quenched with saturated aqueous NH_4Cl. The aqueous layer was separated and

extracted with CH_2Cl_2. The organic layer was then washed with 1M HCl (x 2), dried (Na_2SO_4), filtered and concentrated *in vacuo*. The crude mixture was dissolved in CH_2Cl_2 (12.0 mL) and MeOH (12.0 mL) and (±)-camphor 10-sulfonic acid (1.42 g, 6.10 mmol) was added in one portion at 0 °C. The mixture was then warmed to room temperature and stirred for 46 h. Saturated aqueous $NaHCO_3$ was then added and the aqueous layer was then separated and extracted with CH_2Cl_2, dried (Na_2SO_4), filtered and concentrated *in vacuo*. Purification by flash column chromatography (5-7.5-10-20% EtOAc/petrol) afforded aminoalcohol **34a** (2.07 g, 96% over 2 steps) as a colorless oil; R_f: 0.30 (20% EtOAc/hexane); $[\alpha]^{25}_D$ −24.5 (*c* 2.58, $CHCl_3$); ν_{max}/cm^{-1} (neat) 2954, 2929, 2857, 1511, 1464, 1249, 1065, 939, 879, 833, 774, 671; δ_H (400 MHz, $CDCl_3$) 7.29 (2H, d, *J* 8.5, 14- and 18-*H*), 6.84 (2H, d, *J* 8.5, 15- and 17-*H*), 6.33 (1H, d, *J* 15.7, 12-*H*), 6.09 (1H, dt, *J* 15.7 and 6.9, 11-*H*), 4.05 (1H, br s, 2-*H*), 3.90 (1H, dd, *J* 11.8 and 2.4, 1-*H*H), 3.83-3.81 (4H, m, 1-H*H* and 20-*H*), 3.65 (1H, dd, *J* 9.6 and 2.9, 4-*H*), 3.55 (1H, dd, *J* 7.5 and 2.9, 5-*H*), 3.11 (1H, d, *J* 9.6, 3-*H*), 2.23-2.18 (2H, m, 10-*H*), 1.80-1.69 (2H, m, 6-*H* and 7-*H*H), 1.50-1.41 (3H, m, 9-*H* and 8-*H*H), 1.25 (1H, m, 8-H*H*), 1.05 (1H, m, 7-H*H*), 0.99 (3H, d, *J* 6.6, 19-*H*), 0.94-0.87 (28H, m, 3 x SiC(C*H*$_3$)$_3$ and 7-H*H*), 0.22, 0.14, 0.12, 0.12, 0.11 and 0.09 (6 x 3H, s, 6 x SiC*H*$_3$); δ_C (100 MHz, $CDCl_3$) 158.6 (16-*C*), 130.8 (13-*C*), 129.2 (12-*C*), 128.9 (11-*C*), 127.0 (14- and 18-*C*), 113.9 (15- and 17-*C*), 79.7 (5-*C*), 74.5 (4-*C*), 69.8 (2-*C*), 69.1 (1-*C*), 58.6 (3-*C*), 55.3 (20-*C*), 34.8 (6-*C*), 33.1 (10- or 7-*C*), 33.0 (10- or 7-*C*), 29.8 (9-*C*), 26.5 (8-*C*), 26.0 (SiC(*C*H$_3$)$_3$), 26.0 (SiC(*C*H$_3$)$_3$), 25.9 (SiC(*C*H$_3$)$_3$), 18.4 (Si*C*(CH$_3$)$_3$), 18.1 (Si*C*(CH$_3$)$_3$), 18.0 (Si*C*(CH$_3$)$_3$), 17.5 (6-(*C*H$_3$)), −2.6 (Si*C*H$_3$), −3.3 (Si*C*H$_3$), −3.7 (Si*C*H$_3$), −4.2 (Si*C*H$_3$), −4.5 (Si*C*H$_3$), −4.8 (Si*C*H$_3$); *m/z* (ES^+) 711 (MH^+, 100%); *m/z* HRMS (ES^+) MH^+ calculated for $C_{38}H_{76}NO_5Si_3$ 710.5026, observed 710.5003.

(9*H*-Fluoren-9-yl)methyl ((*R*)-2-((*tert*-butyldimethylsilyl)oxy)-4-(((5*S*,6*S*,7*S*,8*S*)-7-((*tert*-butyldimethylsilyl)oxy)-5-(hydroxymethyl)-8-((*S*,*E*)-8-(4-methoxyphenyl)oct-7-en-2-yl)-2,2,3,3,10,10,11,11-octamethyl-4,9-dioxa-3,10-disiladodecan-6-yl)amino)-4-oxobutyl)carbamate (34b)

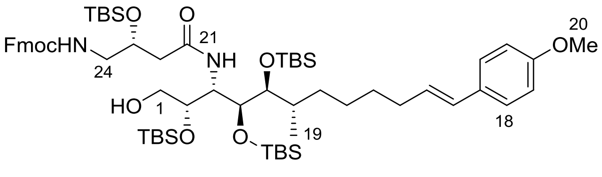

Cyanuric fluoride (0.60 mL, 6.98 mmol) was added dropwise to a solution of **6** (2.26 g, 4.97 mmol) and pyridine (0.40 mL, 4.97 mmol) in CH_2Cl_2 (50.0 mL) at room temperature. The mixture was stirred for 3 h. The reaction was diluted with CH_2Cl_2 and washed with ice-cold water (x 1), dried (Na_2SO_4), filtered and concentrated *in vacuo* to afford acid fluoride **7** as a yellow solid, which was used without further purification in the subsequent step. 4-Methylmorpholine (0.94 mL, 8.57 mmol), hexamethyldisiloxane (1.82 mL, 8.57 mmol), a solution of freshly prepared acid fluoride **7** in CH_2Cl_2 (17.0 mL) were added sequentially to a solution of **34a** (2.03 g, 2.86 mmol) in CH_2Cl_2 (12.0 mL) at 0 ^oC. The solution was stirred for 20 mins at 0 ^oC and then concentrated *in vacuo*. Purification by flash column chromatography (7.5-10-20% EtOAc/petrol) afforded amide **34b** (2.63 g, 80%) as a colorless solid; R_f: 0.30 (10% EtOAc/petrol); m.p. 44-46 ^oC; $[\alpha]^{25}_D$ −4.1 (*c* 3.00, $CHCl_3$); ν_{max}/cm^{-1} (neat) 2929, 2856, 1730, 1657, 1511, 1470, 1249, 1092, 1056, 876, 834, 775, 740, 676; δ_H (400 MHz, $CDCl_3$) 7.77 (2H, d, *J* 7.3, 2 x Ar*H*), 7.61 (2H, d, *J* 7.3, 2 x Ar*H*), 7.41 (2H, dd, *J* 7.3, 2 x Ar*H*), 7.32 (2H, dd, *J* 7.3, 2 x Ar*H*), 7.26 (2H, d, *J* 8.2, 14- and 18-*H*), 6.83 (2H, d, *J* 8.2, 15- and 17-*H*), 6.31 (1H, d, *J* 15.7, 12-*H*), 6.31 (1H, m, N*H*), 6.06 (1H, dt, *J* 15.7 and 6.9, 11-*H*), 5.17 (1H, m, N*H*), 4.43 (1H, dd, *J* 9.5, OC*H*HCH), 4.30-4.18 (5H, m, OCH*H*CH, OCH_2CH, O*H*, 23- and 3-*H*), 4.08 (1H, m, 2-*H*), 4.03 (1H, d, *J* 9.6, 4-*H*), 3.80 (3H, s, 20-*H*), 3.62 (1H, m, 1-*H*H), 3.51 (1H, dd, *J* 9.2 and 1.8, 5-*H*), 3.38-3.36 (2H, m, 24-*H*), 3.23 (1H, m, 1-H*H*), 2.47 (1H, dd, *J* 15.1 and 3.1, 22-*H*H), 2.35 (1H, dd, *J* 15.1 and 8.5, 22-H*H*), 2.20-2.15 (2H, m, 10-*H*), 1.66 (1H, m, 7-*H*H), 1.55 (1H, m, 6-*H*), 1.49-1.35 (3H, m, 9-*H* and 8-*H*H), 1.15 (1H, m, 8-H*H*), 1.02 (3H, d, *J* 6.6, 19-*H*), 0.95-0.87 (37H, m, 7-H*H* and 4 x $SiC(CH_3)_3$), 0.17, 0.16, 0.15, 0.12, 0.10, 0.10, 0.09, 0.08 (8 x 3H, s, 8 x $SiCH_3$); δ_C (100 MHz, $CDCl_3$) 171.0 (21-*C*), 158.6 (16-*C*), 156.4 (*C*(O)), 144.1 ($C_{(Ar)}$), 144.0 ($C_{(Ar)}$), 141.3 (2 x $C_{(Ar)}$), 130.7 (13-*C*), 129.2 (12-*C*), 128.7 (11-*C*), 127.6 ($CH_{(Ar)}$), 127.0 ($CH_{(Ar)}$ or 14- and 18-*C*), 126.9 ($CH_{(Ar)}$ or 14- and 18-*C*), 125.1 ($CH_{(Ar)}$), 119.9 ($CH_{(Ar)}$), 113.9 (15- and 17-*C*), 79.6 (5-*C*), 72.7 (4-*C*), 71.3 (2-*C*), 68.0 (23-*C*), 66.8 (OCH_2CH), 62.7 (1-*C*), 55.3 (20-*C*), 52.9 (3-*C*), 47.2 (OCH_2CH), 46.1 (24-*C*), 41.8 (22-*C*), 35.0 (6-*C*), 34.0 (7-*C*), 33.1 (10-*C*), 30.0 (9-*C*), 26.6 (8-*C*), 26.2 (2 x $SiC(CH_3)_3$), 25.9 ($SiC(CH_3)_3$), 25.8 ($SiC(CH_3)_3$), 18.3 ($SiC(CH_3)_3$), 18.0 (3 x $SiC(CH_3)_3$), 17.1 (19-*C*), −2.4 ($SiCH_3$), −3.1 ($SiCH_3$), −3.4 ($SiCH_3$), −3.7 ($SiCH_3$), −4.4 ($SiCH_3$), −4.5 ($SiCH_3$), −4.7 ($SiCH_3$), −4.9 ($SiCH_3$); *m/z* (ES^+) 1148 (MH^+, 100%); *m/z* HRMS (ES^+) MH^+ calculated for $C_{63}H_{107}N_2O_9Si_4$ 1147.7048, observed 1147.7024.

(9*H*-Fluoren-9-yl)methyl ((*R*)-2-((*tert*-butyldimethylsilyl)oxy)-4-(((5*S*,6*R*,7*S*,8*S*)-7-((*tert*-butyldimethylsilyl)oxy)-5-formyl-8-((*S*,*E*)-8-(4-methoxyphenyl)oct-7-en-2-yl)-

2,2,3,3,10,10,11,11-octamethyl-4,9-dioxa-3,10-disiladodecan-6-yl)amino)-4-oxobutyl)carbamate (34c)

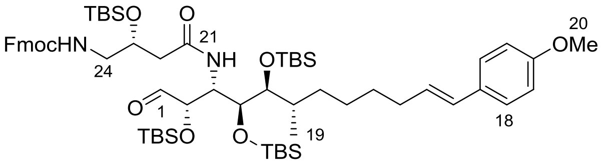

Dess-Martin periodinane (4.79 g, 11.3 mmol) was added to a solution of alcohol **34b** (2.59 g, 2.26 mmol) in wet CH_2Cl_2 (22.6 mL) at 0 ^oC. The solution was then warmed to room temperature and stirred for 5 h. Saturated aqueous $NaHCO_3$ was then added and the aqueous layer was then separated and extracted with CH_2Cl_2, dried (Na_2SO_4), filtered and concentrated *in vacuo*. Purification by flash column chromatography (5-7.5-10% EtOAc/petrol) afforded aldehyde **34c** (2.26 g, 87%) as a colorless solid; R_f: 0.40 (10% EtOAc/petrol); m.p. 48-50 ^oC; $[\alpha]^{25}_D$ +13.3 (*c* 2.58, $CHCl_3$); ν_{max} /cm^{-1} (neat) 2953, 2929, 2856, 1730, 1511, 1464, 1249, 1103, 1041, 1005, 873, 833, 776, 758, 740, 673; δ_H (400 MHz, $CDCl_3$) 9.55 (1H, s, 1-*H*), 7.77 (2H, d, *J* 7.3, 2 x Ar*H*), 7.62 (2H, d, *J* 7.3, 2 x Ar*H*), 7.41 (2H, dd, *J* 7.6, 2 x Ar*H*), 7.32 (2H, dd, *J* 7.3, 2 x Ar*H*), 7.27 (2H, d, *J* 8.8, 14- and 18-*H*), 6.89 (1H, d, *J* 4.2, N*H*), 6.84 (2H, d, *J* 8.8, 15- and 17-*H*), 6.31 (1H, d, *J* 15.7, 12-*H*), 6.06 (1H, dt, *J* 15.7 and 6.9, 11-*H*), 5.23 (1H, t, *J* 5.9, N*H*), 4.41 (1H, dd, *J* 10.3 and 7.1, OC*H*HCH), 4.31-4.19 (4H, m, OCH*H*CH, OCH_2CH, 2- and 23-*H*), 4.02 (1H, dd, *J* 9.5 and 5.6, 3-*H*), 3.91 (1H, d, *J* 8.8, 4-*H*), 3.80 (3H, s, 20-*H*), 3.57 (1H, dd, *J* 8.8 and 2.5, 5-*H*), 3.32-3.31 (2H, m, 24-*H*), 2.35 (2H, d, *J* 6.1, 22-*H*), 2.20-2.15 (2H, m, 10-*H*), 1.77 (1H, m, 6-*H*), 1.65 (1H, m, 7-*H*H), 1.47-1.35 (3H, m, 9-*H* and 8-*H*H), 1.17 (1H, m, 8-H*H*), 1.02 (3H, d, *J* 6.6, 19-*H*), 0.96, 0.91, 0.91 and 0.87 (4 x 9H, s, 4 x $SiC(CH_3)_3$), 0.95 (1H, m, 7-H*H*), 0.18, 0.17, 0.14, 0.14, 0.13, 0.08, 0.07, 0.04 (8 x 3H, s, 8 x $SiCH_3$); δ_C (100 MHz, $CDCl_3$) 200.0 (1-*C*), 169.4 (21-*C*), 158.6 (16-*C*), 156.4 (*C*(O)), 144.1 ($C_{(Ar)}$), 144.0 ($C_{(Ar)}$), 141.3 (2 x $C_{(Ar)}$), 130.6 (13-*C*), 129.2 (12-*C*), 128.6 (11-*C*), 127.6 ($CH_{(Ar)}$), 127.0 ($CH_{(Ar)}$ or 14- and 18-*C*), 127.0 ($CH_{(Ar)}$ or 14- and 18-*C*), 125.2 ($CH_{(Ar)}$), 119.9 ($CH_{(Ar)}$), 113.9 (15- and 17-*C*), 79.4 (5-*C*), 76.8 (2-*C*), 73.2 (4-*C*), 68.0 (23-*C*), 66.8 (OCH_2CH), 55.2 (20- and 3-*C*), 47.2 (OCH_2CH), 46.7 (24-*C*), 42.1 (22-*C*), 35.0 (6-*C*), 33.8 (7-*C*), 33.1 (10-*C*), 30.1 (9-*C*), 26.4 (8-*C*), 26.1 (2 x $SiC(CH_3)_3$), 25.8 (2 x $SiC(CH_3)_3$), 18.4 ($SiC(CH_3)_3$), 18.0 (3 x $SiC(CH_3)_3$), 16.9 (19-*C*), −2.4 ($SiCH_3$), −3.4 ($SiCH_3$), −3.5 ($SiCH_3$), −4.0 ($SiCH_3$), −4.6 ($SiCH_3$), −4.6 ($SiCH_3$), −4.7 ($SiCH_3$), −5.0 ($SiCH_3$); *m/z* (ES^+) 1146

(MH$^+$, 100%); *m/z* HRMS (ES$^+$) MH$^+$ calculated for $C_{63}H_{105}N_2O_9Si_4$ 1145.6892, observed 1145.6882.

(2*S*,3*R*,4*S*,5*S*,6*S*,*E*)-3-((*R*)-4-((((9*H*-Fluoren-9-yl)methoxy)carbonyl)amino)-3-((*tert*-butyldimethylsilyl)oxy)butanamido)-2,4,5-tris((*tert*-butyldimethylsilyl)oxy)-12-(4-methoxyphenyl)-6-methyldodec-11-enoic acid (35)

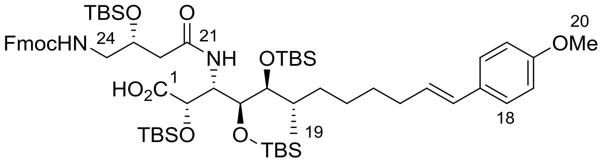

2-Methyl-2-butene (0.82 mL, 7.74 mmol), NaH_2PO_4 (933 mg, 7.78 mmol) and $NaClO_2$ (308 mg, 2.72 mmol) were added sequentially to a solution of aldehyde **34c** (2.23 g, 1.95 mmol) in *tert*-butanol/water (16.0 mL, 3:1) at 0 ^{o}C. The solution was then slowly warmed to room temperature and stirred for 2.5 h. The solution was then diluted with EtOAc, water was added and the aqueous layer was then separated and extracted with EtOAc, dried (Na_2SO_4), filtered and concentrated *in vacuo* to afford crude acid **35** (2.26 g, quant.) as a colorless solid. R_f: 0.60 (50% EtOAc/petrol); 7.76 (2H, d, *J* 7.2, 2 x Ar*H*), 7.60 (2H, d, *J* 7.5, 2 x Ar*H*), 7.39 (2H, dd, *J* 7.5, 2 x Ar*H*), 7.31-7.25 (4H, m, 2 x Ar*H*, 14- and 18-*H*), 6.83 (2H, d, *J* 8.4, 15- and 17-*H*), 6.30 (1H, d, *J* 15.7, 12-*H*), 6.06 (1H, dt, *J* 15.7 and 7.0, 11-*H*), 5.33 (1H, br s, N*H*), 4.55-4.20 (6H, m, OCH_2CH, OCH$_2$C*H*, 2-, 3- and 23-*H*), 3.91 (1H, m, 4-*H*), 3.80 (3H, s, 20-*H*), 3.52 (1H, d, *J* 9.2, 5-*H*), 3.34 (2H, br s, 24-*H*), 2.41 (1H, dd, *J* 15.3 and 4.1, 22-*H*H), 2.28 (1H, dd, *J* 15.3 and 9.0, 22-H*H*), 2.19-2.14 (2H, m, 10-*H*), 1.72-1.61 (2H, m, 6-*H* and 7-*H*H), 1.49-1.35 (3H, m, 9-*H* and 8-*H*H), 1.19 (1H, m, 8-H*H*), 1.00 (3H, d, *J* 6.7, 19-*H*), 0.94-0.87 (37H, m, 7-H*H* and 4 x SiC(CH_3)$_3$), 0.18, 0.17, 0.16, 0.16, 0.10, 0.10, 0.06, 0.06 (8 x 3H, s, 8 x SiCH_3). The compound was used immediately and without further purification in the subsequent step.

Synthesis of pyrrolidinone *epi*-21.

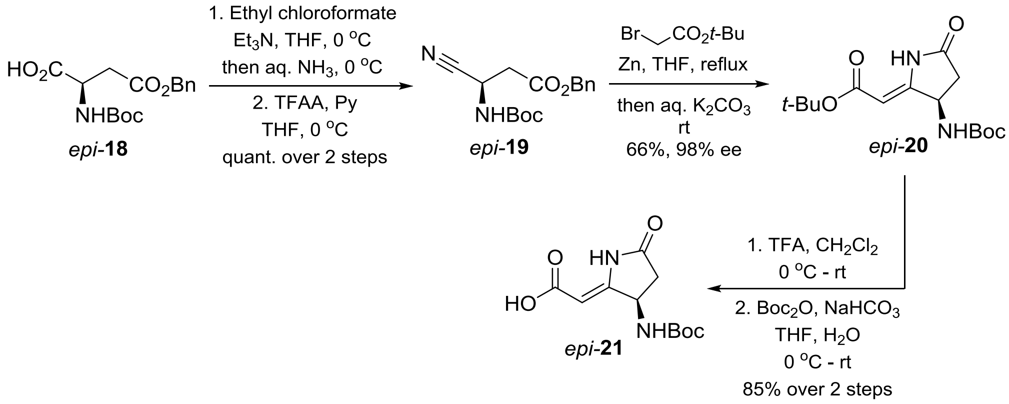

(*R*)-Benzyl 4-amino-3-((*tert*-butoxycarbonyl)amino)-4-oxobutanoate (*epi*-18a)

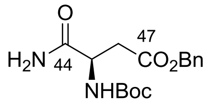

Ethyl chloroformate (1.74 mL, 18.2 mmol) was slowly added to an ice-cold solution of triethylamine (2.53 mL, 18.2 mmol) and (*R*)-4-(benzyloxy)-2-((*tert*-butoxycarbonyl)amino)-4-oxobutanoic acid (4.89 g, 15.1 mmol) in THF (75.0 mL) and the solution was stirred for 2 h at 0 °C. The resulting suspension was treated with ammonia (12.0 mL of a 35% aqueous solution) and stirred for 30 min at 0 °C. The aqueous phase was then separated and extracted with ethyl acetate, dried (Na_2SO_4), filtered and concentrated *in vacuo* to afford amide *epi*-**18a** (4.88 g, quant.) as a colorless solid; R_f: 0.40 (50% EtOAc/petrol); m.p. 146-148 °C; $[\alpha]^{25}_D$ −8.5 (*c* 0.58, $CHCl_3$); ν_{max}/cm^{-1} (neat) 2359, 2174, 2103, 1976, 1915, 1734, 1673, 1637, 1518, 1295, 1161, 982, 754, 724, 663, 626; δ_H (400 MHz, D_6-DMSO) 7.37-7.32 (5H, m, 5 x Ph*H*), 7.25 (1H, br s, N*H*), 7.09 (1H, br s, N*H*), 7.03 (1H, d, *J* 8.4, N*H*), 5.08 (2H, d, *J* 2.0, OCH_2Ph), 4.29 (1H, m, 45-*H*), 2.77 (1H, dd, *J* 16.0 and 5.3, 46-*H*H), 4.58 (1H, dd, *J* 16.0 and 8.7, 46-H*H*), 1.38 (9H, s, $CO_2C(CH_3)_3$); δ_C (100 MHz, D_6-DMSO) 172.6 (44- or 47-*C*), 170.3 (44- or 47-*C*), 155.1 (*C*(O)), 136.1 ($C_{(Ar)}$), 128.4 ($CH_{(Ar)}$), 127.9 ($CH_{(Ar)}$), 127.7 ($CH_{(Ar)}$), 78.2 ($CO_2C(CH_3)_3$), 65.6 (O*C*H_2Ph), 50.8 (45-*C*), 36.4 (46-*C*), 28.1 ($CO_2C(CH_3)_3$); *m/z* (ES^+) 345 (MNa^+, 50%); *m/z* HRMS (ES^+) MH^+ calculated for $C_{16}H_{23}O_5N_2$ 323.1602, observed 323.1602.

(*R*)-Benzyl 3-((*tert*-butoxycarbonyl)amino)-3-cyanopropanoate (*epi*-19)

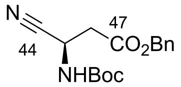

Amide *epi*-**18a** (4.88 g, 15.1 mmol) was suspended in THF (76.0 mL) and pyridine (6.12 mL, 75.7 mmol) and freshly distilled trifluoroacetic anhydride (4.21 mL, 30.3 mmol) were added sequentially at 0 °C. The red solution was stirred for 45 mins at 0 °C and then diluted with EtOAc. After warming to room temperature the solution was washed with aqueous saturated $NaHCO_3$ (x 2), 1 M HCl and brine solutions and then dried (Na_2SO_4), filtered and concentrated *in vacuo*. Purification by flash column chromatography (20% EtOAc/petrol) afforded nitrile *epi*-**18a** (4.69 g, quant.) as a pale yellow oil, which then solidified in the freezer; R_f: 0.30 (20% EtOAc/petrol); m.p. 43-45 ^{o}C; $[\alpha]^{25}_{D}$ +25.0 (*c* 0.92, $CHCl_3$); ν_{max}/cm^{-1} (neat) 3337, 2362, 2334, 1735, 1689, 1519, 1253, 1226, 1186, 1152, 1053, 1030, 971, 753, 695, 668, 650, 634, 612; δ_H (400 MHz, $CDCl_3$) 7.42-7.36 (5H, m, 5 x Ph*H*), 5.60 (1H, d, *J* 6.9, N*H*), 5.21 (2H, s, OCH_2Ph), 4.95 (1H, br s, 45-*H*), 2.94 (1H, dd, *J* 17.4 and 5.1, 46-*H*H), 2.86 (1H, dd, *J* 17.4 and 5.4, 46-H*H*), 1.46 (9H, s, $CO_2C(CH_3)_3$); δ_C (100 MHz, $CDCl_3$) 169.1 (47-*C*), 154.1 (*C*(O)), 134.8 ($C_{(Ar)}$), 128.7 ($CH_{(Ar)}$), 128.7 ($CH_{(Ar)}$), 128.5 ($CH_{(Ar)}$), 117.7 (44-*C*), 81.5 ($CO_2C(CH_3)_3$), 67.5 (O*C*H_2Ph), 38.3 (45-*C*), 37.4 (46-*C*), 28.2 ($CO_2C(CH_3)_3$); *m/z* HRMS (ES^+) MH^+ calculated for $C_{16}H_{21}O_4N_2$ 305.1496, observed 305.1495.

(*R,Z*)-*tert*-Butyl 2-(3-((*tert*-butoxycarbonyl)amino)-5-oxopyrrolidin-2-ylidene)acetate (*epi*-20)

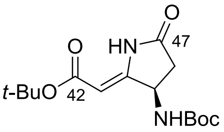

Activated zinc was prepared by washing zinc powder with 3 M HCl, water, ethanol and diethyl ether. After drying under reduced pressure, activated zinc powder (4.95 g, 75.7 mmol) was suspended in THF (66.0 mL) and heated to reflux. Following the reported procedure by Kishi[5] a few drops of *tert*-butyl bromoacetate and 1,2-dibromoethane were added and the mixture was stirred for 15 min. After addition of nitrile *epi*-**19** (4.67 g, 15.1 mmol), dissolved in THF (33.0 mL)), *tert*-butyl bromoacetate (8.94 mL, 60.6 mmol), dissolved in THF

(33.0 mL)), was added over a 40 min period *via* syringe pump. The black colored reaction mixture was stirred for further 10 min at reflux and then cooled to room temperature. After the addition of THF (44.0 mL) and K_2CO_3 (22.0 mL of a 50% aqueous solution) the mixture was vigorously stirred for 15 min, filtered over Celite and washed with diethyl ether. The solution was then dried (Na_2SO_4), filtered and concentrated *in vacuo*. Purification by flash column chromatography (15-20% EtOAc/petrol) afforded pyrrolidinone *epi*-**20** (3.11 g, 66%) as a colorless foam; R_f: 0.25 (20% EtOAc/petrol); m.p. 53-55 ^{o}C; $[\alpha]^{25}_{D}$ +69.8 (*c* 1.65, $CHCl_3$); ν_{max}/cm^{-1} (neat) 1756, 1679, 1643, 1518, 1367, 1270, 1253,1208, 1165, 1138, 816, 755, 675, 654, 627, 608; δ_H (400 MHz, $CDCl_3$) 9.74 (1H, s, N*H*), 5.11 (1H, d, *J* 1.0, 43-*H*), 5.01-4.94 (2H, m, N*H* and 45-*H*), 2.91 (1H, dd, *J* 18.1 and 9.1, 46-*H*H), 2.37 (1H, dd, *J* 17.9 and 5.6, 46-H*H*), 1.47 (9H, s, $CO_2C(CH_3)_3$), 1.45 (9H, s, $CO_2C(CH_3)_3$); δ_C (100 MHz, $CDCl_3$) 173.9 (47-*C*), 167.5 (42-*C*), 156.6 (44-*C*), 155.1 (*C*(O)), 92.5 (43-*C*), 80.8 (2 x $CO_2C(CH_3)_3$), 48.8 (45-*C*), 36.5 (46-*C*), 28.3 ($CO_2C(CH_3)_3$), 28.2 ($CO_2C(CH_3)_3$); *m/z* (ES^+) 335 (MNa^+, 100%); *m/z* HRMS (ES^+) MH^+ calculated for $C_{15}H_{25}O_5N_2$ 313.1758, observed 313.1754; Assay of enantiomeric excess (conditions: 90:10 heptane/*i*-PrOH, 1.0 mL/min.): 98% ee (t_R (minor) = 28.8 min., t_R (major) = 18.5 min.).

(*R*,*Z*)-2-(3-((*tert*-Butoxycarbonyl)amino)-5-oxopyrrolidin-2-ylidene)acetic acid (*epi*-21)

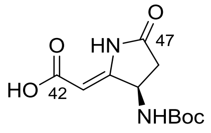

Trifluoroacetic acid (9.50 mL, 124 mmol) was added to a solution of (*R*,*Z*)-*tert*-butyl 2-(3-((*tert*-butoxycarbonyl)amino)-5-oxopyrrolidin-2-ylidene)acetate *epi*-**20** (1.87 g, 5.99 mmol) in CH_2Cl_2 (50.0 mL) at room temperature. The reaction was stirred for 4 h. The solution was then concentrated *in vacuo* and the residue then dissolved in toluene and again concentrated *in vacuo* (x 3) to afford the corresponding free amino acid salt as an orange foam. The amino acid was subsequently dissolved in THF/H_2O (41.0 mL, 1:1) and $NaHCO_3$ (1.76 g, 21.0 mmol) and di-*tert*-butyl dicarbonate (2.75 mL, 12.0 mmol) were added at 0 ^{o}C. The mixture was warmed to room temperature and stirred for 20 h. The solution was acidified to pH 2-3 with aqueous HCl solution (1 M) and then diluted with EtOAc. The aqueous layer was separated and extracted with EtOAc, dried (Na_2SO_4), filtered and concentrated *in vacuo* to afford acid *epi*-**21** (1.31 g, 85% over 2 steps) as a colorless solid;

R_f: 0.25 (5% MeOH/EtOAc); m.p. 95-97 °C; $[\alpha]^{20}_D$ +102.7 (*c* 2.33, EtOAc); ν_{max}/cm^{-1} (neat) 2362, 2035, 1682, 1515, 1258, 1165, 753, 712, 661; δ_H (400 MHz, D_6-DMSO) 12.08 (1H, br s, CO_2*H*), 9.98 (1H, s, N*H*), 7.49 (1H, d, *J* 8.6, N*H*), 4.88 (1H, s, 43-*H*), 4.78 (1H, m, 45-*H*), 2.73 (1H, dd, *J* 17.4 and 9.5, 46-*H*H), 2.32 (1H, dd, *J* 17.4 and 5.9, 46-H*H*), 1.40 (9H, s, $CO_2C(CH_3)_3$); δ_C (100 MHz, D_6-DMSO) 174.7 (47-*C*), 168.8 (42-*C*), 160.0 (44-*C*), 155.3 (*C*(O)), 89.1 (43-*C*), 78.7 (CO_2*C*$(CH_3)_3$), 48.3 (45-*C*), 34.4 (46-*C*), 28.2 ($CO_2C(CH_3)_3$). *m/z* (ES^-) 255 (M-H, 100%); *m/z* HRMS (ES^+) MH^+ calculated for $C_{11}H_{17}O_5N_2$ 257.1132, observed 257.1143.

The enantiopurity of amino acid *epi*-21 was confirmed by chiral HPLC analysis of the corresponding methyl ester:

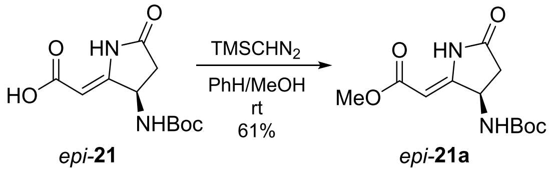

(*R*,*Z*)-Methyl 2-(3-((*tert*-butoxycarbonyl)amino)-5-oxopyrrolidin-2-ylidene)acetate (*epi*-21a)

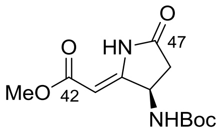

Carboxylic acid *epi*-**21** (100 mg, 0.39 mmol) was dissolved in toluene (6.50 mL) and methanol (1.63 mL) and treated with trimethylsilyl diazomethane (0.39 mL of a 2 M solution in hexanes, 0.78 mmol). After stirring the mixture for 1 h acetic acid (10.0 mL of 2 M aqueous solution) was added. The aqueous layer was separated and extracted with EtOAc, dried (Na_2SO_4), filtered and concentrated *in vacuo*. Purification by flash column chromatography (30-50% EtOAc/petrol) gave methyl ester *epi*-**21a** (64.3 mg, 61%) as a colorless solid; R_f: 0.50 (50% EtOAc/petrol); m.p. 178-180 °C; $[\alpha]^{20}_D$ +123.9 (*c* 2.00, EtOAc); ν_{max} /cm^{-1} (neat) 2362, 2162, 2065, 2014, 1689, 1524, 1173, 691, 667, 633, 612; δ_H (400 MHz, $CDCl_3$) 9.72 (1H, br s, N*H*), 5.22 (1H, d, *J* 7.9, N*H*), 5.18 (1H, s, 43-*H*), 4.94 (1H, m, 45-*H*), 3.70 (3H, s, CO_2CH_3), 2.89 (1H, dd, *J* 18.0 and 9.3, 46-*H*H), 2.41 (1H, dd, *J* 18.0 and 5.3, 46-H*H*), 1.43 (9H, s, $CO_2C(CH_3)_3$); δ_C (100 MHz, $CDCl_3$) 174.1 (47-*C*),

168.2 (42-*C*), 157.9 (44-*C*), 155.0 (*C*(O)), 90.2 (43-*C*), 80.7 ($CO_2C(CH_3)_3$), 51.3 (CO_2CH_3), 48.7 (45-*C*), 36.0 (46-*C*), 28.2 ($CO_2C(CH_3)_3$); *m/z* (ES^+) 293 (MNa^+, 100%); *m/z* HRMS (ES^+) MNa^+ calculated for $C_{12}H_{18}N_2O_5Na$ 293.1108, observed 293.1108; Assay of enantiomeric excess (conditions: 80:20 heptane/*i*-PrOH, 1.0 mL/min.): 98% ee (t_R (major) = 11.6 min., t_R (minor) = 21.3 min.).

Synthesis of *epi*-31.

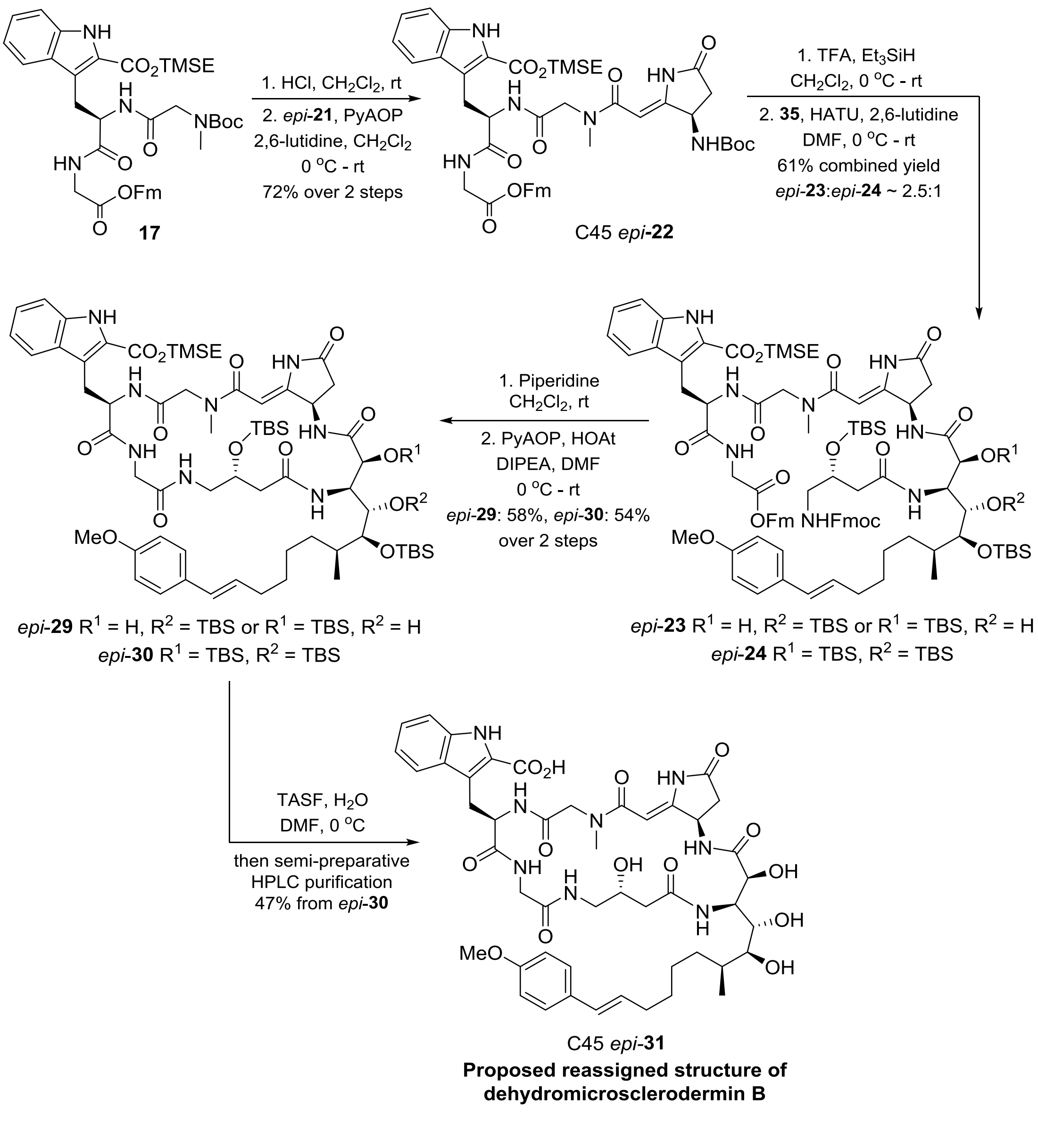

2-(Trimethylsilyl)ethyl 3-((*R*)-3-((2-((9*H*-fluoren-9-yl)methoxy)-2-oxoethyl)amino)-2-(2-((*Z*)-2-((*R*)-3-((*tert*-butoxycarbonyl)amino)-5-oxopyrrolidin-2-ylidene)-*N*-methylacetamido)acetamido)-3-oxopropyl)-1*H*-indole-2-carboxylate (*epi*-22)

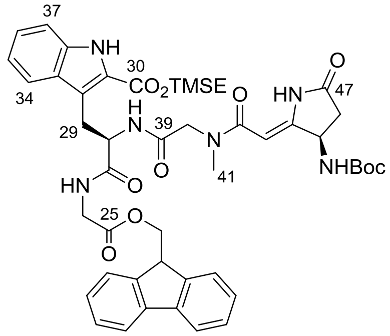

Hydrogen chloride (0.99 mL of a 4 M solution in dioxane, 3.98 mmol) was added to a solution of protected tripeptide **17** (200 mg, 0.27 mmol) in CH_2Cl_2 (3.12 mL) at room temperature. The mixture was stirred for 4 h, concentrated *in vacuo* and the residue then dissolved in toluene and again concentrated *in vacuo* (x 2) to afford the corresponding amine salt, as an amorphous colorless solid, which was used directly in the subsequent step. Carboxylic acid *epi*-**21** (81.5 mg, 0.32 mmol) was dissolved in CH_2Cl_2 (1.5 mL) and cooled to 0 ^{o}C. 2,6-Lutidine (0.15 mL, 1.27 mmol) and PyAOP (166 mg, 0.32 mmol) were then added sequentially. The mixture was stirred at 0 ^{o}C for 2 h. The amine salt was dissolved in CH_2Cl_2 (2.0 mL) and added to the solution. The mixture was warmed slowly to room temperature, stirred for 16 h and then concentrated *in vacuo*. Purification by flash column chromatography (60-70-80-100% EtOAc/petrol) afforded tetrapeptide *epi*-**22** (170 mg, 72% over 2 steps) as a colorless solid; R_f: 0.30 (80% EtOAc/petrol); m.p. 118-120 ^{o}C; $[\alpha]^{25}_{D}$ +49.0 (*c* 0.61, acetone); ν_{max}/cm^{-1} (neat) 3656, 2978, 2890, 2363, 1466, 1386, 1252, 1154, 1076, 956, 668; δ_H (500 MHz, D_6-DMSO (* denotes major rotamer)) 11.52 (1H, s, N*H*), 10.35 and 10.29* (1H, br s, N*H*), 8.50 and 8.37* (1H, br s, N*H*), 8.06 (1H, d, *J* 8.2, N*H*), 7.89 (2H, d, *J* 7.6, 2 x Ar*H*), 7.79 (1H, d, *J* 7.9, 34-*H*), 7.69 (2H, d, *J* 7.6, 2 x Ar*H*), 7.48 (1H, d, *J* 8.0, N*H*), 7.43-7.33 (5H, m, 37-*H* and 4 x Ar*H*), 7.22 (1H, dd, *J* 7.1, 36-*H*), 7.05 (1H, dd, *J* 7.1, 35-*H*), 5.30* and 5.07 (1H, s, 43-*H*), 4.81-4.69 (2H, m, 28- and 45-*H*), 4.40-4.35 (4H, m, OCH_2CH and $TMSCH_2CH_2$), 4.26 (1H, t, *J* 6.9, OCH_2CH), 4.01 (1H, m, 40-*H*H), 3.93-3.83 (2H, m, 26-*H*), 3.64-3.49 (2H, m, 40-H*H* and 29-*H*H), 3.26 (1H, m, 29-H*H*), 2.73 (1H, dd, *J* 18.0 and 10.3, 46-*H*H), 2.50* and 2.43 (3H, br s, 41-*H*), 2.29 (1H, dd, *J* 18.0 and 4.9, 46-H*H*), 1.40* and 1.29 (9H, s, $CO_2C(CH_3)_3$), 1.16-1.13 (2H, m,

TMSCH_2CH_2), 0.04 (9H, s, Si(CH_3)$_3$); δ_C (126 MHz, D_6-DMSO (chemical shifts reported for the major rotamer only)) 174.5 (47-*C*), 171.5 (27-*C*), 169.6 (25-*C*), 167.7 (42- or 39-*C*), 167.5 (42- or 39-*C*), 162.0 (30-*C*), 157.8 (*C*(O)), 155.5 (44-*C*), 143.5 ($C_{(Ar)}$), 140.7 ($C_{(Ar)}$), 136.1 ($C_{(Ar)}$), 127.8 ($CH_{(Ar)}$), 127.8 ($C_{(Ar)}$), 127.2 ($CH_{(Ar)}$), 125.3 ($CH_{(Ar)}$), 124.6 (36-*C*), 124.1 ($C_{(Ar)}$), 120.7 (34-*C*), 120.2 ($CH_{(Ar)}$), 119.4 (35-*C*), 118.6 ($C_{(Ar)}$), 112.3 (37-*C*), 87.3 (43-*C*), 79.2 ($CO_2C(CH_3)_3$), 66.2 (O*C*H_2CH), 62.4 (TMSCH_2*C*H_2), 53.6 (28-*C*), 49.8 (40-*C*), 48.4 (45-*C*), 46.1 (OCH_2*C*H), 40.9 (26-*C*), 35.4 (41-*C*), 34.3 (46-*C*), 28.1 ($CO_2C(CH_3)_3$), 27.5 (29-*C*), 17.1 (TMS*C*H_2CH_2), −1.6 (Si(*C*H_3)$_3$); δ_H (500 MHz, D_6-DMSO, 363 K) 11.21 (1H, s, N*H*), 10.24 (1H, s, N*H*), 8.01 (1H, br s, N*H*), 7.86 (2H, d, *J* 7.6, 2 x Ar*H*), 7.79 (1H, d, *J* 8.2, 34-*H*), 7.68-7.67 (3H, m, N*H* and 2 x Ar*H*), 7.43-7.40 (3H, m, 2 x Ar*H* and 37-*H*), 7.34 (2H, dd, *J* 7.6, 2 x Ar*H*), 7.23 (1H, dd, *J* 7.9, 36-*H*), 7.11 (1H, br s, N*H*), 7.06 (1H, dd, *J* 7.9, 35-*H*), 5.29 (1H, s, 43-*H*), 4.75-4.68 (2H, m, 28- and 45-*H*), 4.45-4.38 (4H, m, OCH_2CH and TMSCH_2CH_2), 4.26 (1H, dd, *J* 7.3, OCH_2C*H*), 3.94-3.90 (3H, m, 40-*H*H and 26-*H*), 3.74 (1H, d, *J* 16.4, 40-H*H*), 3.56 (1H, dd, *J* 13.6 and 5.0, 29-*H*H), 3.36 (1H, dd, *J* 13.6 and 9.1, 29-H*H*), 2.73 (1H, dd, *J* 17.3 and 9.5, 46-*H*H), 2.63 (3H, s, 41-*H*), 2.33 (1H, dd, *J* 17.3 and 5.4, 46-H*H*), 1.39 (9H, s, $CO_2C(CH_3)_3$), 1.20-1.17 (2H, m, TMSCH_2CH_2), 0.08 (9H, s, Si(CH_3)$_3$); *m/z* (ES^+) 729 (50%), 751 (100%), 893 (MH^+, 100%); *m/z* HRMS (ES^+) MH^+ calculated for $C_{47}H_{57}O_{10}N_6Si$ 893.3899, observed 893.3907.

2-(Trimethylsilyl)ethyl 3-((*R*)-3-((2-((9*H*-fluoren-9-yl)methoxy)-2-oxoethyl)amino)-2-(2-((*Z*)-2-((*R*)-3-((2*S*,3*R*,4*S*,5*S*,6*S*,*E*)-3-((*R*)-4-((((9*H*-fluoren-9-yl)methoxy)carbonyl)amino)-3-((*tert*-butyldimethylsilyl)oxy)butanamido)-2,5-bis((*tert*-butyldimethylsilyl)oxy)-4-hydroxy-12-(4-methoxyphenyl)-6-methyldodec-11-enamido)-5-oxopyrrolidin-2-ylidene)-*N*-methylacetamido)acetamido)-3-oxopropyl)-1*H*-indole-2-carboxylate (*epi*-23)

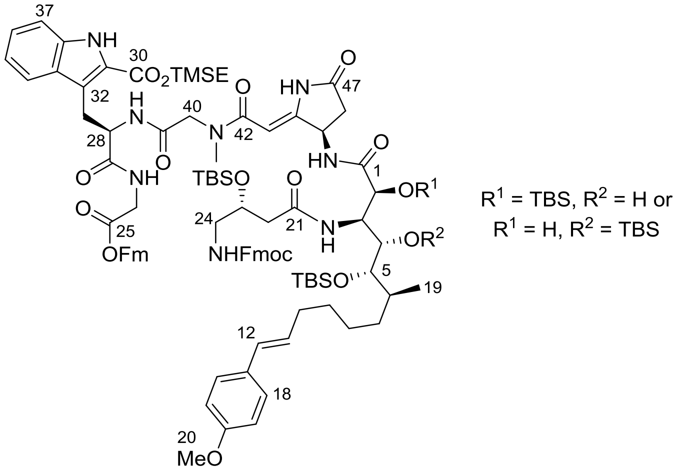

Trifluoroacetic acid (0.22 mL, 2.80 mmol) and triethylsilane (89.0 μL, 0.56 mmol) were added sequentially to a solution of tetrapeptide *epi*-**22** (250 mg, 0.28 mmol) in CH_2Cl_2 (2.80 mL) at 0 °C. The reaction was warmed to room temperature and stirred for 2 h. The solution was then concentrated *in vacuo* and the residue then dissolved in toluene and again concentrated *in vacuo* (x 2) to afford the corresponding amine salt, as a colorless solid, which was used directly in the subsequent step. HATU (55.9 mg, 0.15 mmol), 2,6-lutidine (53.0 μL, 0.45 mmol) and the amine salt of the deprotected tetrapeptide (121 mg, 0.15 mmol) were added sequentially to a solution of carboxylic acid **35** (118 mg, 0.10 mmol) in DMF (1.13 mL) at 0 °C. The mixture was warmed to room temperature and stirred for 16 h. The solution was then diluted with EtOAc and washed with brine solution (x 2), dried (Na_2SO_4), filtered and concentrated *in vacuo*. Purification by flash column chromatography (40-50-60-70-80% EtOAc/petrol) afforded linear hexapeptides *epi*-**23** (85.1 mg, 46% from acid **35**) and *epi*-**24** (30.2 mg, 15% from acid **35**) as colorless solids. Data for the linear hexapeptide *epi*-**23**: R_f 0.30 (60% EtOAc/petrol); m.p. 97-99 °C; $[\alpha]^{25}_D$ −3.8 (*c* 3.19, EtOAc); ν_{max} /cm^{-1} (neat) 2974, 2361, 2297, 1660, 1512, 1468, 1246, 1031, 1005, 779, 760, 741, 668; δ_H (500 MHz, D_6-DMSO) 11.51 (1H, s, N*H*), 10.33 (1H, s, N*H*), 8.34-8.31 (2H, m, 2 x N*H*), 8.07 (1H, d, *J* 8.2, N*H*), 7.87 (4H, dd, *J* 8.0, 4 x Ar*H*), 7.80 (1H, d, *J* 7.9, 34-*H*), 7.69-7.67

(5H, m, N*H* and 4 x Ar*H*), 7.42-7.25 (11H, m, 14-, 18-, 37-*H* and 8 x Ar*H*), 7.21 (1H, dd, *J* 7.4, 36-*H*), 7.10 (1H, br s, N*H*), 7.04 (1H, dd, *J* 7.4, 35-*H*), 6.83 (2H, d, *J* 8.7, 15- and 17-*H*), 6.26 (1H, d, *J* 15.9, 12-*H*), 6.05 (1H, dt, *J* 15.9 and 6.8, 11-*H*), 5.60 (1H, br s, O*H*), 5.26 (1H, s, 43-*H*), 4.76-4.69 (2H, m, 45- and 28-*H*), 4.39-4.16 (9H, m, 2-*H*, 2 x OCH_2CH, 2 x OCH_2CH and $TMSCH_2CH_2$), 4.10 (1H, m, 3-*H*), 3.98 (1H, m, 23-*H*), 3.87-3.81 (4H, m, 40-*H*H, 26- and 4-*H*), 3.71 (3H, s, 20-*H*), 3.71 (1H, m, 40-H*H*), 3.58 (1H, m, 29-*H*H), 3.33 (1H, m, 5-*H*), 3.28 (1H, m, 29-H*H*), 3.02-2.96 (2H, m, 24-*H*), 2.62 (1H, m, 46-*H*H), 2.53 (3H, s, 41-*H*), 2.50 (1H, m, 46-H*H*), 2.25 (1H, m, 22-*H*H), 2.15-2.10 (3H, m, 22-H*H* and 10-*H*), 1.60-1.54 (2H, m, 6-*H* and 7-*H*H), 1.35-1.22 (3H, m, 8-*H*H and 9-*H*), 1.15-1.11 (2H, m, $TMSCH_2CH_2$), 1.09 (1H, m, 8-H*H*), 0.87-0.77 (31H, m, 3 x $SiC(CH_3)_3$, 19-*H* and 7-H*H*), 0.13-−0.05 (27H, m, 6 x $SiCH_3$ and $Si(CH_3)_3$); δ_C (126 MHz, D_6-DMSO) 175.4 (47-*C*), 172.9 (1-*C*), 171.5 (27-*C*), 169.6 (25-*C*), 168.2 (21-*C*), 167.8 (42-*C*), 167.8 (39-*C*), 162.0 (30-*C*), 158.3 (16-*C*), 157.1 (44-*C*), 156.4 (*C*(O)), 143.9 ($C_{(Ar)}$), 143.8 ($C_{(Ar)}$), 143.5 (2 x $C_{(Ar)}$), 140.7 (4 x $C_{(Ar)}$), 136.1 (38-*C*), 130.0 (13-*C*), 129.1 (12-*C*), 128.1 (11-*C*), 127.8 ($CH_{(Ar)}$), 127.6 (33-*C*), 127.6 ($CH_{(Ar)}$), 127.2 ($CH_{(Ar)}$), 127.0 ($CH_{(Ar)}$), 126.9 (14- and 18-*C*), 125.3 ($CH_{(Ar)}$), 125.1 ($CH_{(Ar)}$), 124.6 (36-*C*), 124.1 (32-*C*), 120.8 (34-*C*), 120.2 ($CH_{(Ar)}$), 120.1 ($CH_{(Ar)}$), 119.5 (35-*C*), 118.6 (31-*C*), 113.9 (15- and 17-*C*), 112.3 (37-*C*), 87.9 (43-*C*), 78.3 (5-*C*), 72.7 (4-*C*), 70.2 (3-*C*), 68.0 (23-*C*), 66.2 (OCH_2CH), 65.6 (OCH_2CH), 62.4 ($TMSCH_2CH_2$), 55.0 (20-*C*), 53.7 (28-*C*), 52.9 (2-*C*), 49.8 (40-*C*), 47.4 (45-*C*), 46.7 (OCH_2CH), 46.1 (OCH_2CH), 45.6 (24-*C*), 42.0 (22-*C*), 40.9 (26-*C*), 35.8 (41-*C*), 33.5 (46-*C*), 33.3 (7-*C*), 32.5 (10-*C*), 29.4 (9-*C*), 27.5 (29- and 6-*C*), 26.1 ($SiC(CH_3)_3$), 25.9 (8-*C*), 25.7 (2 x $SiC(CH_3)_3$), 18.0 ($SiC(CH_3)_3$), 17.9 ($SiC(CH_3)_3$), 17.6 ($SiC(CH_3)_3$), 17.1 ($TMSCH_2CH_2$), 16.4 (19-*C*), −1.6 ($Si(CH_3)_3$), −3.3 (2 x $SiCH_3$), −4.9 ($SiCH_3$), −5.0 (2 x $SiCH_3$), −5.3 ($SiCH_3$); *m/z* (ES^+) 1845 (MNa^+, 100), 1823 (MH^+, 90); *m/z* HRMS (ES^+) MNa^+ calculated for $C_{99}H_{136}N_8O_{17}Si_4Na$ 1844.9026, observed 1844.8948.

2-(Trimethylsilyl)ethyl 3-((*R*)-3-((2-((9*H*-fluoren-9-yl)methoxy)-2-oxoethyl)amino)-2-(2-((*Z*)-2-((*R*)-3-((2*S*,3*R*,4*S*,5*S*,6*S*,*E*)-3-((*R*)-4-((((9*H*-fluoren-9-yl)methoxy)carbonyl)amino)-3-((*tert*-butyldimethylsilyl)oxy)butanamido)-2,4,5-tris((*tert*-butyldimethylsilyl)oxy)-12-(4-methoxyphenyl)-6-methyldodec-11-enamido)-5-oxopyrrolidin-2-ylidene)-*N*-methylacetamido)acetamido)-3-oxopropyl)-1*H*-indole-2-carboxylate (*epi*-24)

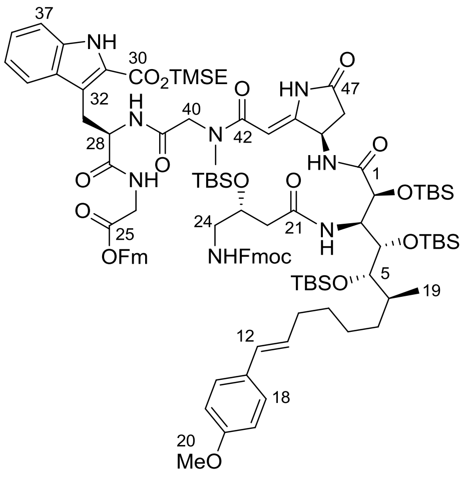

R_f 0.50 (60% EtOAc/petrol); m.p. 88-90 ^{o}C; $[\alpha]^{25}_{D}$ +27.2 (*c* 1.27, EtOAc); ν_{max} /cm^{-1} (neat) 3022, 2930, 2857, 2362, 1701, 1679, 1659, 1512, 1249, 1196, 1179, 1101, 1035, 1007, 856, 835, 777, 759, 741, 623; δ_H (500 MHz, D_6-DMSO) 11.51 (1H, s, N*H*), 10.42 (1H, s, N*H*), 8.26 (1H, br s, N*H*), 8.00 (1H, d, *J* 8.7, N*H*), 7.87 (4H, dd, *J* 7.9, 4 x Ar*H*), 7.87 (1H, m, N*H*), 7.77 (1H, d, *J* 7.9, 34-*H*), 7.68-7.64 (5H, m, N*H* and 4 x Ar*H*), 7.42-7.26 (12H, m, 8 x Ar*H*, N*H*, 14-, 18- and 37-*H*), 7.19 (1H, dd, *J* 7.4, 36-*H*), 7.03 (1H, dd, *J* 7.4, 35-*H*), 6.84 (1H, d, *J* 8.4, 15 and 17-*H*), 6.28 (1H, d, *J* 15.9, 12-*H*), 6.07 (1H, dt, *J* 15.9 and 6.8, 11-*H*), 5.20 (1H, br s, 43-*H*), 4.79-4.68 (2H, m, 45- and 28-*H*), 4.39-4.19 (9H, m, , 2 x OCH_2CH, 2 x OCH_2C*H* and TMSCH_2CH_2), 4.13 (1H, dd, *J* 9.9, 3-*H*), 4.01 (1H, m, 23-*H*), 3.90-3.85 (3H, m, and 26-*H*), 3.74-3.71 (5H, m, 40-H*H*, 2- or 4-*H* and 20-*H*), 3.55 (1H, m, 29-*H*H), 3.46 (1H, m, 5-*H*), 3.28 (1H, m, 29-H*H*), 3.10 (1H, m, 24-*H*H), 2.96 (1H, m, 24-H*H*), 2.63-2.52 (5H, m, 46- and 41-*H*), 2.30 (1H, m, 22-*H*H), 2.12-2.10 (3H, m, 10-*H* and 22-H*H*), 1.58-1.53 (2H, m, 6-*H* and 7-*H*H), 1.37-1.30 (3H, m, 8-*H*H and 9-*H*), 1.16-1.12 (2H, m, TMSC$H_2$$CH_2$), 1.09 (1H, m, 8-H*H*), 0.86-0.70 (40H, m, 4 x SiC(CH_3)$_3$, 19-*H* and 7-H*H*), 0.17-−0.05 (33H, m, 8 x SiCH_3 and Si(CH_3)$_3$); δ_C (126 MHz, D_6-DMSO) 175.1 (47-*C*), 171.5 (1- and 27-*C*), 169.6 (25-*C*), 167.8 (42-, 21- and 39-*C*), 162.0 (30-*C*), 158.3 (16-*C*), 157.0 (44-*C* and *C*(O)), 143.8 (*C*$_{(Ar)}$), 143.7 (*C*$_{(Ar)}$), 143.5 (2 x *C*$_{(Ar)}$), 140.8 (*C*$_{(Ar)}$), 140.7 (3 x *C*$_{(Ar)}$), 136.1 (38-*C*), 129.9

(13-*C*), 129.1 (12-*C*), 128.0 (11-*C*), 127.7 ($CH_{(Ar)}$), 127.5 ($CH_{(Ar)}$ and 33-*C*), 127.2 ($CH_{(Ar)}$), 126.9 ($CH_{(Ar)}$), 126.9 (14- and 18-*C*), 125.3 ($CH_{(Ar)}$), 125.2 ($CH_{(Ar)}$), 125.1 ($CH_{(Ar)}$), 124.6 (36-*C*), 124.2 (32-*C*), 120.7 (34-*C*), 120.1 (2 x $CH_{(Ar)}$), 119.4 (35-*C*), 118.5 (31-*C*), 113.9 (15- and 17-*C*), 112.3 (37-*C*), 87.6 (43-*C*), 72.2 (4- or 2-*C*), 70.0 (5-*C*), 68.2 (23-*C*), 66.2 (OCH_2CH), 65.8 (OCH_2CH), 62.4 (TMSCH_2CH_2), 55.0 (20-*C*), 53.7 (3- and 28-*C*), 52.0 (4- or 2-*C*), 50.0 (40-*C*), 47.6 (45-*C*), 46.6 (OCH_2*C*H), 46.1 (OCH_2*C*H), 45.3 (24-*C*), 41.7 (22-*C*), 40.9 (26-*C*), 35.5 (41-*C*), 33.5 (7-*C*), 33.3 (46-*C*), 32.6 (10-*C*), 29.4 (9-*C*), 27.3 (6- and 29-*C*), 26.1 (8-*C*), 25.8 (2 x SiC(CH_3)$_3$), 25.7 (2 x SiC(CH_3)$_3$), 17.9 (2 x Si*C*(CH_3)$_3$), 17.7 (2 x Si*C*(CH_3)$_3$), 17.1 (TMS*C*H_2CH_2), 16.1 (19-*C*), −1.6 (Si(CH_3)$_3$), −2.3 (SiCH_3), −3.2 (SiCH_3), −3.5 (SiCH_3), −3.5 (SiCH_3), −4.2 (SiCH_3), −4.9 (SiCH_3), −4.9 (SiCH_3), −5.2 (SiCH_3); *m/z* (ES^+) 1936 (MH^+, 100); *m/z* HRMS (ES^+) MNa^+ calculated for $C_{105}H_{151}N_8O_{17}Si_5$ 1937.0072, observed 1937.0034.

2-(Trimethylsilyl)ethyl 3-(((3a*R*,6*S*,7*R*,11*R*,18*R*,*Z*)-6,11-bis((*tert*-butyldimethylsilyl)oxy)-7-((1*S*,2*S*,3*S*,*E*)-2-((*tert*-butyldimethylsilyl)oxy)-1-hydroxy-9-(4-methoxyphenyl)-3-methylnon-8-en-1-yl)-22-methyl-2,5,9,14,17,20,23-heptaoxo-1,2,3,3a,4,5,6,7,8,9,10,11,12,13,14,15,16,17,18,19,20,21,22,23-tetracosahydropyrrolo[2,3-*m*][1,4,7,10,15,19]hexaazacyclotricosin-18-yl)methyl)-1*H*-indole-2-carboxylate (*epi*-29)

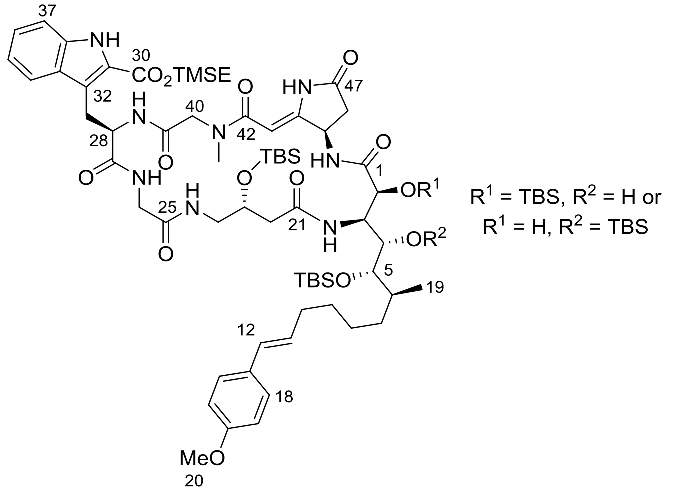

Piperidine (0.25 mL, 2.53 mmol) was added to a solution of linear hexapeptide *epi*-**23** (35.0 mg, 19.2 μmol) in CH_2Cl_2 (5.00 mL) at room temperature. The reaction was stirred for 2 h. The solution was then concentrated *in vacuo* and the crude mixture was dried under high vacuum for 4 h at 40 ^{o}C. Purification by flash column chromatography (2-5-25% MeOH/$CHCl_3$) afforded piperidine salt of linear amino acid as a colorless solid. The

piperidine salt of amino acid was dissolved in DMF (19.2 mL) and cooled to 0 ^{o}C. PyAOP (100 mg, 0.19 mmol), HOAt (26.1 mg, 0.19 mmol) and *N*,*N*-diisopropylethylamine (33.0 μL, 0.19 μmol) were then added sequentially. The mixture was warmed slowly to room temperature, stirred for 6 days and concentrated *in vacuo*. The residue was then redissolved in 2% MeOH/$CHCl_3$, washed with H_2O, dried (Na_2SO_4), filtered and concentrated *in vacuo*. Purification by flash column chromatography (60-100% EtOAc/petrol) afforded cyclic hexapeptide *epi*-**29** (15.7 mg, 58% over 2 steps) as a colorless solid; R_f 0.25 (80% EtOAc/petrol); m.p. 170-172 ^{o}C; $[\alpha]^{25}_{D}$ −42.1 (*c* 1.40, EtOAc); ν_{max} /cm^{-1} (neat) 2929, 2858, 2157, 1659, 1528, 1511, 1250, 1096, 836, 776, 669; δ_H (500 MHz, D_4-MeOD) 7.73 (1H, d, *J* 8.2, 34-*H*), 7.44 (1H, d, *J* 8.4, 37-*H*), 7.30 (1H, ddd, *J* 8.2 and 0.8, 36-*H*), 7.22 (2H, d, *J* 8.7, 14- and 18-*H*), 7.11 (1H, ddd, *J* 8.2 and 0.8, 35-*H*), 6.76 (2H, d, *J* 8.7, 15- and 17-*H*), 8.37 (1H, d, *J* 9.3, N*H* or O*H*), 6.27 (1H, d, *J* 15.8, 12-*H*), 6.04 (1H, dt, *J* 15.8 and 6.9, 11-*H*), 5.61 (1H, d, *J* 1.4, 43-*H*), 5.24 (1H, ddd, *J* 9.5, 6.3 and 1.1, 45-*H*), 4.56-4.20 (5H, m, TMSCH$_2$C*H*$_2$, 23-, 28- and 4-*H*), 4.35 (1H, s, 2-*H*), 4.29 (1H, d, *J* 17.7, 29-*H*H), 4.16 (1H, d, *J* 14.7, 40-*H*H), 4.01 (1H, dd, *J* 9.5 and 2.1, 3-*H*), 3.66 (3H, s, 20-*H*), 3.62 (2H, d, *J* 6.8, 26-*H*), 3.51 (1H, dd, *J* 9.3 and 2.5, 5-*H*), 3.32-3.27 (5H, m, 41-*H*, 29-H*H* and 40-H*H*), 3.09 (1H, m, 24-*H*H), 2.76-2.70 (2H, m, 46-*H*H and 24-H*H*), 2.63 (1H, dd, *J* 18.0 and 6.5, 46-H*H*), 2.48 (1H, dd, *J* 14.5 and 4.7, 22-*H*H), 2.21-2.12 (2H, m, 10-*H*), 2.08 (1H, dd, *J* 14.5 and 2.1, 22-H*H*), 1.72-1.67 (2H, m, 6-*H* and 7-*H*H), 1.45-1.39 (2H, m, 9-*H*H and 8-*H*H), 1.33 (1H, m, 9-H*H*), 1.27-1.24 (3H, m, 8-H*H* and TMSC*H*$_2$CH$_2$), 0.99 (3H, d, *J* 6.6, 19-*H*), 0.97 (9H, s, SiC(C*H*$_3$)$_3$), 0.94 (9H, s, SiC(C*H*$_3$)$_3$), 0.94 (1H, m, 7-H*H*), 0.82 (9H, s, SiC(C*H*$_3$)$_3$), 0.22, 0.18, 0.16, 0.13 (4 x 3H, s, 4 x SiC*H*$_3$), 0.11 (9H, s, Si(C*H*$_3$)$_3$), 0.10, 0.06 (2 x 3H, s, 2 x SiC*H*$_3$); δ_C (126 MHz, D_4-MeOD) 177.3 (47-*C*), 177.3 (1-*C*), 174.5 (39-*C*), 173.3 (27-*C*), 172.8 (25-*C*), 171.0 (42-*C*), 169.8 (21-*C*), 165.2 (30-*C*), 160.3 (16-*C*), 157.7 (44-*C*), 138.1 (38-*C*), 132.2 (13-*C*), 130.8 (12-*C*), 129.5 (11-*C*), 129.0 (33-*C*), 128.3 (14- and 18-*C*), 126.9 (36-*C*), 126.0 (32-*C*), 121.4 (34-*C*), 121.4 (35-*C*), 119.5 (31-*C*), 115.0 (15- and 17-*C*), 113.6 (37-*C*), 91.5 (43-*C*), 81.1 (5-*C*), 75.4 (3-*C*), 71.8 (2-*C*), 68.1 (23-*C*), 64.9 (TMSCH$_2$*C*H$_2$), 59.1 (4-*C*), 55.8 (20-*C*), 55.2 (28-*C*), 54.8 (40-*C*), 48.7 (45-*C*), 48.1 (24-*C*), 46.3 (22-*C*), 43.1 (29-*C*), 39.9 (41-*C*), 35.8 (6-*C*), 35.0 (7-*C*), 34.7 (10- or 46-*C*), 34.6 (10- or 46-*C*), 31.0 (9-*C*), 27.5 (8-*C*), 27.1 (SiC(*C*H$_3$)$_3$), 26.8 (SiC(*C*H$_3$)$_3$), 26.6 (SiC(*C*H$_3$)$_3$), 26.6 (26-*C*), 19.4 (Si*C*(CH$_3$)$_3$), 19.1 (Si*C*(CH$_3$)$_3$), 19.0 (Si*C*(CH$_3$)$_3$), 18.9 (TMS*C*H$_2$CH$_2$), 17.5 (19-*C*), −1.3 (Si(*C*H$_3$)$_3$), −2.4 (Si*C*H$_3$), −2.8 (Si*C*H$_3$), −3.7 (Si*C*H$_3$), −4.0 (Si*C*H$_3$), −4.2 (Si*C*H$_3$), −5.1 (Si*C*H$_3$); *m/z* (ES$^+$) 1404 (MH$^+$, 100), 1426 (MNa$^+$, 80%); *m/z* HRMS (ES$^+$) MH$^+$ calculated for $C_{70}H_{115}N_8O_{14}Si_4$ 1404.7638, observed 1404.7606.

2-(Trimethylsilyl)ethyl 3-(((3a*R*,6*S*,7*R*,11*R*,18*R*,*Z*)-6,11-bis((*tert*-butyldimethylsilyl)oxy)-7-((5*S*,6*S*)-6-((*S*,*E*)-8-(4-methoxyphenyl)oct-7-en-2-yl)-2,2,3,3,8,8,9,9-octamethyl-4,7-dioxa-3,8-disiladecan-5-yl)-22-methyl-2,5,9,14,17,20,23-heptaoxo-1,2,3,3a,4,5,6,7,8,9,10,11,12,13,14,15,16,17,18,19,20,21,22,23-tetracosahydropyrrolo[2,3-*m*][1,4,7,10,15,19]hexaazacyclotricosin-18-yl)methyl)-1*H*-indole-2-carboxylate (*epi*-30)

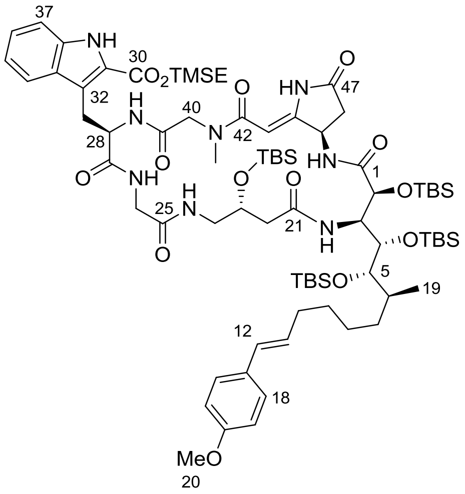

Piperidine (0.25 mL, 2.53 mmol) was added to a solution of linear hexapeptide *epi*-**24** (15.0 mg, 7.74 μmol) in CH_2Cl_2 (5.00 mL) at room temperature. The reaction was stirred for 2 h. The solution was then concentrated *in vacuo* and the crude mixture was dried under high vacuum for 4 h at 40 °C. Purification by flash column chromatography (2-5-25% MeOH/$CHCl_3$) afforded piperidine salt of linear amino acid as a colorless solid. The piperidine salt of amino acid was dissolved in DMF (7.80 mL) and cooled to 0 °C. PyAOP (40.4 mg, 77.4 μmol), HOAt (10.5 mg, 77.4 μmol) and *N*,*N*-diisopropylethylamine (14.0 μL, 77.4 μmol) were then added sequentially. The mixture was warmed slowly to room temperature, stirred for 6 days and concentrated *in vacuo*. The residue was then redissolved in 2% MeOH/$CHCl_3$, washed with H_2O, dried (Na_2SO_4), filtered and concentrated *in vacuo*. Purification by flash column chromatography (50-70% EtOAc/petrol) afforded cyclic hexapeptide *epi*-**30** (6.4 mg, 54% over 2 steps) as a colorless solid; R_f 0.50 (60% EtOAc/petrol); m.p. 141-143 °C; $[\alpha]^{25}_{D}$ −40.4 (*c* 0.70, EtOAc); ν_{max} /cm^{-1} (neat) 2542, 2200, 2158, 2117, 2084, 2050, 2007, 1975, 1948, 1658, 1521, 1251, 1101, 833, 772, 690, 674, 648, 622; δ_H (500 MHz, D_4-MeOD) 7.71 (1H, d, *J* 8.2, 34-*H*), 7.44 (1H, d, *J* 8.4, 37-*H*), 7.30 (1H, ddd, *J* 8.0 and 0.8, 36-*H*), 7.21 (2H, d, *J* 8.7, 14- and 18-*H*), 7.11 (1H, dd, *J* 8.0, 35-*H*), 6.75 (2H, d, *J* 8.7, 15- and 17-*H*), 6.27 (1H, d, *J* 15.8, 12-*H*), 6.04 (1H, dt,

J 15.8 and 7.1, 11-*H*), 5.66 (1H, s, 43-*H*), 5.30 (1H, dd, *J* 7.9 and 1.4, 45-*H*), 4.59-4.40 (6H, m, TMS$CH_2C\mathit{H_2}$, 23-, 28-, 4- and 2-*H*), 4.23 (1H, d, *J* 17.2, 29-*H*H), 4.18 (1H, d, *J* 14.9, 40-*H*H), 3.84 (1H, dd, *J* 9.8 and 2.4, 3-*H*), 3.68 (3H, s, 20-*H*), 3.63-3.60 (3H, m, 26- and 5-*H*), 3.29 (3H, s, 41-*H*), 3.18 (1H, d, *J* 17.2, 29-*H*H), 3.11-3.06 (2H, m, 24-*H*H and 40-H*H*), 2.80-2.74 (2H, m, 46-*H*H and 24-H*H*), 2.54 (1H, dd, *J* 19.6 and 4.4, 22-*H*H), 2.48 (1H, dd, *J* 17.5 and 7.9, 46-H*H*), 2.19-2.07 (3H, m, 22-H*H* and 10-*H*), 1.71-1.67 (2H, m, 6-*H* and 7-*H*H), 1.46-1.39 (2H, m, 9-*H*H and 8-*H*H), 1.33 (1H, m, 9-H*H*), 1.28-1.24 (3H, m, 8-H*H* and TMS$C\mathit{H_2}CH_2$), 1.02 (9H, s, $SiC(C\mathit{H_3})_3$), 1.00 (3H, d, *J* 6.2, 19-*H*), 0.96 (9H, s, $SiC(C\mathit{H_3})_3$), 0.96 (9H, s, $SiC(C\mathit{H_3})_3$), 0.94 (1H, m, 7-H*H*), 0.84 (9H, s, $SiC(C\mathit{H_3})_3$), 0.23-0.07 (33H, m, 8 x $SiC\mathit{H_3}$ and $Si(C\mathit{H_3})_3$); δ_C (126 MHz, D_4-MeOD) 176.3 (47-*C*), 175.3 (1-*C*), 174.0 (25-*C*), 173.3 (27-*C*), 172.3 (39-*C*), 170.8 (42-*C*), 169.4 (21-*C*), 165.2 (30-*C*), 160.4 (16-*C*), 156.4 (44-*C*), 138.1 (38-*C*), 132.1 (13-*C*), 130.9 (12-*C*), 129.5 (11-*C*), 129.1 (33-*C*), 128.3 (14- and 18-*C*), 126.9 (36-*C*), 126.0 (32-*C*), 121.4 (34- and 35-*C*), 119.4 (31-*C*), 115.0 (15- and 17-*C*), 113.5 (37-*C*), 91.6 (43-*C*), 80.6 (5-*C*), 76.9 (3-*C*), 73.7 (2- or 4-*C*), 68.2 (23-*C*), 64.9 (TMS$CH_2\mathit{C}H_2$), 59.1 (2- or 4-*C*), 56.0 (28-*C*), 55.8 (20-*C*), 54.3 (40-*C*), 48.7 (45-*C*), 47.9 (24-*C*), 45.3 (22-*C*), 43.0 (29-*C*), 39.7 (41-*C*), 35.5 (46-*C*), 34.9 (7- and 6-*C*), 34.8 (10-*C*), 30.9 (9-*C*), 27.8 (8-*C*), 27.1 ($SiC(\mathit{C}H_3)_3$), 27.0 ($SiC(\mathit{C}H_3)_3$), 26.7 (2 x $SiC(\mathit{C}H_3)_3$), 26.6 (26-*C*), 19.4 ($Si\mathit{C}(CH_3)_3$), 19.3 ($Si\mathit{C}(CH_3)_3$), 19.0 ($Si\mathit{C}(CH_3)_3$), 18.9 ($Si\mathit{C}(CH_3)_3$), 18.9 (TMS$\mathit{C}H_2CH_2$), 17.6 (19-*C*), −0.9 ($Si\mathit{C}H_3$), −1.3 ($Si\mathit{C}H_3$ and $Si(\mathit{C}H_3)_3$), −2.5 ($Si\mathit{C}H_3$), −3.1 ($Si\mathit{C}H_3$), −3.7 ($Si\mathit{C}H_3$), −3.7 ($Si\mathit{C}H_3$), −4.0 ($Si\mathit{C}H_3$), −4.1 ($Si\mathit{C}H_3$); *m/z* (ES^+) 1518 (MH^+, 100); *m/z* HRMS (ES^+) MH^+ calculated for $C_{76}H_{129}N_8O_{14}Si_5$ 1517.8469, observed 1517.8457.

3-(((3a*R*,6*S*,7*R*,11*R*,18*R*,*Z*)-7-((1*S*,2*S*,3*S*,*E*)-1,2-dihydroxy-9-(4-methoxyphenyl)-3-methylnon-8-en-1-yl)-6,11-dihydroxy-22-methyl-2,5,9,14,17,20,23-heptaoxo-1,2,3,3a,4,5,6,7,8,9,10,11,12,13,14,15,16,17,18,19,20,21,22,23-tetracosahydropyrrolo[2,3-*m*][1,4,7,10,15,19]hexaazacyclotricosin-18-yl)methyl)-1*H*-indole-2-carboxylic acid (*epi*-31)

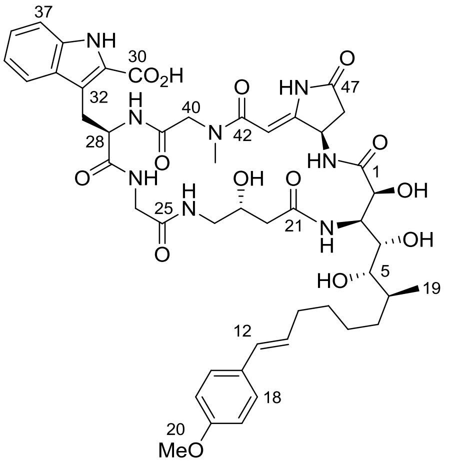

H_2O (1.00 µL, 55.6 µmol) and TASF (79.0 µL of a 0.25 M solution in DMF, 19.8 µmol) were added sequentially to a solution of protected cyclic peptide *epi*-**30** (2.00 mg, 1.32 µmol) in DMF (0.10 mL) at 0 °C. The reaction was stirred for 8 h at 0 °C. Afterwhich TMS_2O (100 µL) was added and the solution was stirred at room temperature for 10 mins. The solution was then concentrated using high vacuum centrifugal evaporator. Purification by flash column chromatography (10-20-25% MeOH/$CHCl_3$) and then semi preparative reverse phase HPLC (XBridge Prep C18 5.0 µm 10 mm x 250 mm column, at 4.4 mL/min with UV detection 214 nm: 2 mins – 5% CH_3CN in 0.05% aqueous TFA, linear increase; 3 mins – 42% CH_3CN in 0.05% aqueous TFA, isocratic; 23 mins – 42% CH_3CN in 0.05% aqueous TFA, linear increase; 24 mins – 95% CH_3CN in 0.05% aqueous TFA, isocratic) afforded deprotected cyclic peptide *epi*-**31** (0.6 mg, 47%) as a colorless solid; R_f 0.30 (30% MeOH/$CHCl_3$); $[\alpha]^{20}_D$ −48.4 (*c* 0.23, 0.1 M NH_4HCO_3, pH 7); ν_{max} /cm^{-1} (neat) 3301, 2923, 2852, 1737, 1531, 1335, 1292, 1247, 1203, 1174, 1024, 997, 824, 763, 692; δ_H (500 MHz, D_6-DMSO) 13.02 (1H, br s, CO_2*H*), 11.54 (1H, s, N*H*), 10.52 (1H, s, N*H*), 8.68 (1H, br s, N*H*), 8.40 (1H, d, *J* 8.8, N*H*), 8.32 (1H, t, *J* 5.2, N*H*), 7.55 (1H, d, *J* 8.0, 34-*H*), 7.43 (1H, t, *J* 6.2, N*H*), 7.39 (1H, d, *J* 8.2, 37-*H*), 7.30 (2H, d, *J* 8.7, 14- and 18-*H*), 7.23 (1H, dd, *J* 7.3, 36-*H*), 7.10-7.07 (2H, m, 35-*H* and N*H*), 6.85 (2H, d, *J* 8.7, 15- and 17-*H*), 6.31 (1H, d, *J* 15.9, 12-*H*), 6.14-6.08 (2H, m, 11-*H* and O*H*), 5.25 (1H, m, 45-*H*), 5.21 (1H, s, 43-*H*), 5.07

(1H, d, *J* 4.7, O*H*), 4.56 (1H, d, *J* 16.1, 40-*H*H), 4.42 (1H, d, *J* 5.4, 2-*H*), 4.32 (1H, d, *J* 9.5, O*H*), 4.11-4.06 (2H, m, 3-*H* and O*H*), 4.00 (1H, m, 28-*H*), 3.73 (3H, s, 20-*H*), 3.68 (1H, m, 23-*H*), 3.60 (1H, dd, *J* 17.0 and 6.3, 26-*H*H), 3.53 (1H, dd, *J* 13.6 and 5.7, 29-*H*H), 3.42-3.37 (3H, m, 24-*H*H, 29-H*H* and 4-*H*), 3.31-3.25 (2H, m, 26-H*H* and 40-H*H*), 3.04 (1H, m, 5-*H*), 2.94 (3H, s, 41-*H*), 2.76 (1H, dd, *J* 17.7 and 9.9, 46-*H*H), 2.60 (1H, m, 24-H*H*), 2.46 (1H, dd, *J* 17.7 and 5.4, 46-H*H*), 2.32 (1H, d, *J* 13.6, 22-*H*H), 2.17-2.12 (2H, m, 10-*H*), 2.03 (1H, dd, *J* 13.7 and 10.9, 22-H*H*), 1.72 (1H, m, 7-*H*H), 1.59 (1H, m, 6-*H*), 1.41-1.38 (3H, m, 8-*H*H and 9-*H*), 1.20 (1H, m, 8-H*H*), 1.01 (1H, m, 7-H*H*), 0.76 (3H, d, *J* 6.6, 19-*H*); δ_C (126 MHz, D_6-DMSO) 174.9 (47-*C*), 173.5 (1-*C*), 172.6 (21-*C*), 171.0 (27-*C*), 170.3 (39-*C*), 169.0 (25-*C*), 167.9 (42-*C*), 163.5 (30-*C*), 158.3 (16-*C*), 157.6 (44-*C*), 135.9 (38-*C*), 130.1 (13-*C*), 128.9 (12-*C*), 128.4 (11-*C*), 127.9 (33-*C*), 126.9 (14- and 18-*C*), 124.8 (32-*C*), 124.5 (36-*C*), 120.3 (34-*C*), 119.6 (35-*C*), 118.1 (31-*C*), 113.9 (15- and 17-*C*), 112.2 (37-*C*), 87.6 (43-*C*), 72.9 (5-*C*), 69.4 (2-*C*), 68.4 (4-*C*), 66.9 (23-*C*), 56.2 (28-*C*), 55.0 (20-*C*), 53.7 (3-*C*), 49.7 (40-*C*), 45.9 (45-*C*), 45.4 (24-*C*), 42.9 (26-*C*), 41.4 (22-*C*), 36.3 (41-*C*), 34.4 (6-*C*), 34.2 (46-*C*), 32.7 (7-*C*), 32.5 (10-*C*), 29.6 (9-*C*), 26.1 (8-*C*), 25.0 (29-*C*), 15.5 (19-*C*); *m/z* (ES^+) 962 (MH^+, 100), 984 (MNa^+, 10); *m/z* HRMS (ES^+) MH^+ calculated for $C_{47}H_{61}N_8O_{14}$ 961.4302, observed 961.4308.

Synthesis of *epi*-36.

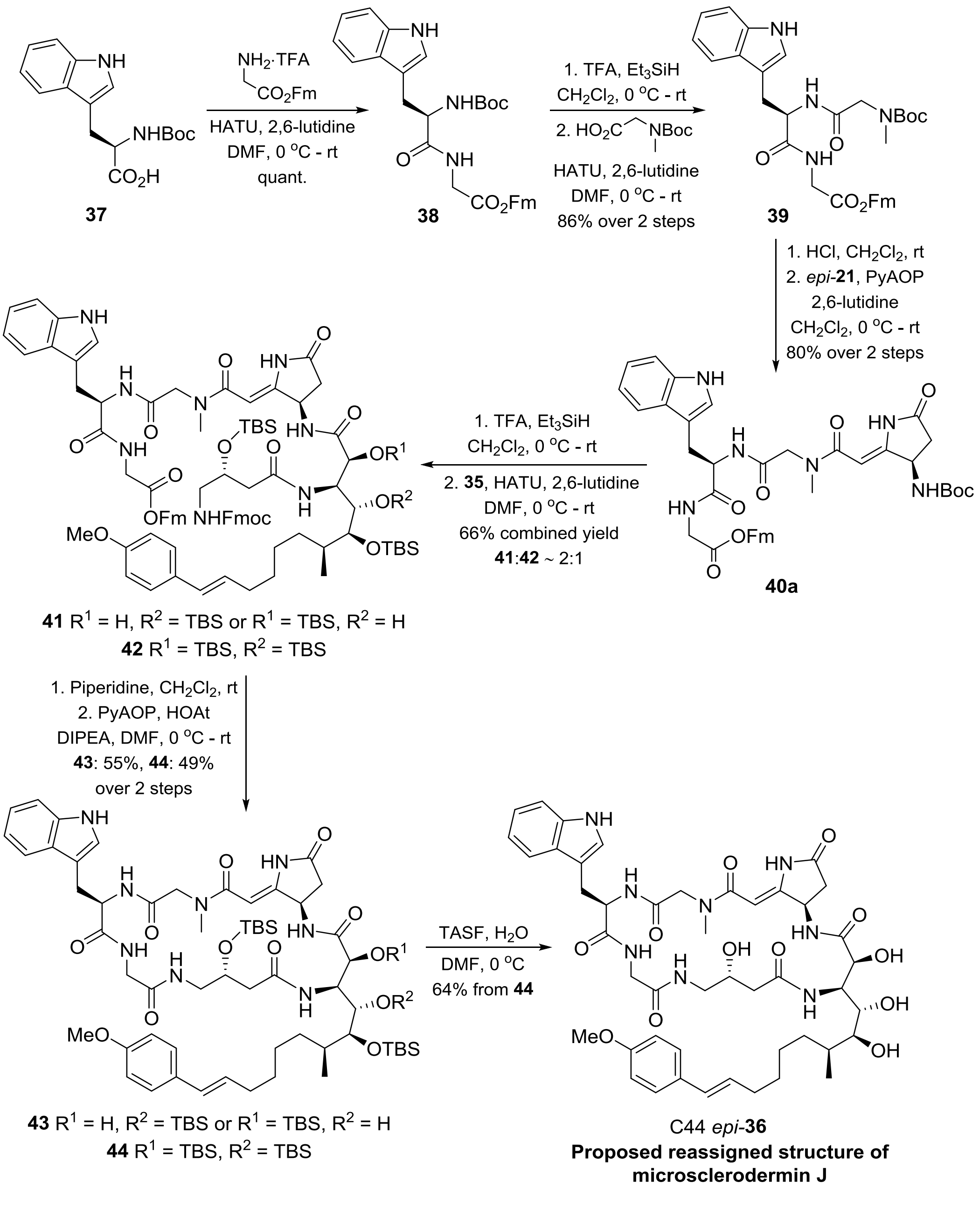

(*R*)-(9*H*-Fluoren-9-yl)methyl 2-(2-((*tert*-butoxycarbonyl)amino)-3-(1*H*-indol-3-yl)propanamido)acetate (38)

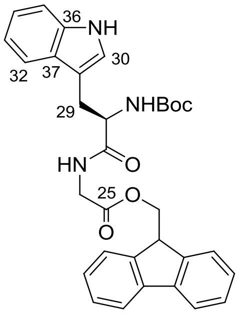

Boc-D-Trp-OH (4.69 g, 15.4 mmol) was dissolved in DMF (160 mL) and cooled to 0 °C, 2,6-lutidine (5.40 mL, 46.4 mmol), HATU (6.44 g, 16.9 mmol) and Gly-OFm·TFA (5.92 g, 16.0 mmol) were then added sequentially. The mixture was then warmed to room temperature and stirred for 16 h. The reaction was then diluted with EtOAc, washed with brine solution (x 2), dried (Na_2SO_4), filtered and concentrated *in vacuo*. Purification by flash column chromatography (30-50% EtOAc/pentane) afforded dipeptide **38** (8.30 g, quant.) as a colorless solid; R_f: 0.20 (50% EtOAc/pentane); m.p. 78-80 °C; $[\alpha]^{25}_D$ +5.5 (*c* 1.83, $CHCl_3$); ν_{max}/cm^{-1} (neat) 3322, 2978, 1746, 1663, 1498, 1450, 1391, 1366, 1164, 1102, 1011, 854, 739, 665, 621; δ_H (400 MHz, $CDCl_3$) 8.15 (1H, s, N*H*), 7.78 (2H, d, *J* 7.6, 2 x Ar*H*), 7.65 (1H, d, *J* 7.6, 32-*H*), 7.55 (2H, dd, *J* 7.6 and 3.7, 2 x Ar*H*), 7.42 (2H, dd, *J* 7.6, 2 x Ar*H*), 7.35-7.31 (3H, m, 2 x Ar*H* and 35-*H*), 7.19-7.10 (2H, m, 33- and 34-*H*), 7.09 (1H, d, *J* 1.2, 30-*H*), 6.36 (1H, t, *J* 5.1, N*H*), 5.16 (1H, br s, N*H*), 4.51 (1H, m, 28-*H*), 4.37 (2H, d, *J* 7.3, OCH_2CH), 4.19 (1H, t, *J* 7.3, OCH_2C*H*), 4.05 (1H, dd, *J* 18.2 and 5.1, 26-*H*H), 3.97 (1H, dd, *J* 18.2 and 4.7, 26-H*H*), 3.34 (1H, m, 29-*H*H), 3.21 (1H, dd, *J* 14.2 and 6.9, 29-H*H*), 1.43 (9H, s, $CO_2C(CH_3)_3$); δ_C (400 MHz, $CDCl_3$) 172.1 (27-*C*), 169.3 (25-*C*), 155.5 (38-*C*), 143.3 (2 x $C_{(Ar)}$), 141.2 (2 x $C_{(Ar)}$), 136.1 (36-*C*), 127.9 (2 x $CH_{(Ar)}$), 127.5 (37-*C*), 127.1 (2 x $CH_{(Ar)}$), 125.0 (2 x $CH_{(Ar)}$), 123.3 (30-*C*), 122.1 (34-*C*), 120.0 (2 x $CH_{(Ar)}$), 119.6 (33-*C*), 118.7 (32-*C*), 111.2 (35-*C*), 110.3 (31-*C*), 80.2 ($CO_2C(CH_3)_3$), 67.2 (OCH_2CH), 55.1 (28-*C*), 46.5 (OCH_2*C*H), 41.3 (26-*C*), 28.2 (29-*C* and $CO_2C(CH_3)_3$); *m/z* (ES^+) 540 (MH^+); *m/z* HRMS (ES^+) MNa^+ calculated for $C_{32}H_{33}O_5N_3Na$ 562.2312, observed 562.2306.

(*R*)-(9*H*-Fluoren-9-yl)methyl 9-((1*H*-indol-3-yl)methyl)-2,2,5-trimethyl-4,7,10-trioxo-3-oxa-5,8,11-triazatridecan-13-oate (39)

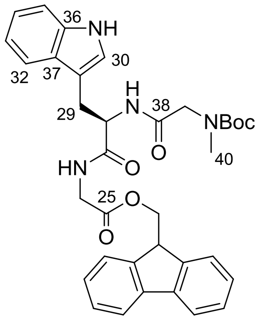

Trifluoroacetic acid (2.10 mL, 27.6 mmol) and triethylsilane (0.90 mL, 5.52 mmol) were added sequentially to a solution of dipeptide **38** (0.99 g, 1.83 mmol) in CH_2Cl_2 (11.0 mL) at 0 ºC. The mixture then was warmed to room temperature and stirred for 2 h. The solution was concentrated *in vacuo* and the residue then re-dissolved in toluene and concentrated *in vacuo* again (x 3) to afford amine salt as a colorless foam. The amine salt then was dissolved in DMF (12.0 mL) and cooled to 0 °C, 2,6-lutidine (0.64 mL, 5.50 mmol), HATU (0.91 g, 2.39 mmol) and Boc-Sar-OH (404 mg, 2.14 mmol) were then added sequentially. The mixture was then warmed to room temperature and stirred for 16 h. The reaction was then diluted with EtOAc and washed with brine solution (x 2), dried (Na_2SO_4), filtered and concentrated *in vacuo*. Purification by flash column chromatography (30-50% EtOAc/pentane) afforded tripeptide **39** (0.97 g, 86% over 2 steps) as a colorless solid; R_f: 0.55 (EtOAc); m.p. 90-92 °C; $[\alpha]^{25}_D$ +30.9 (*c* 2.12, $CHCl_3$); ν_{max}/cm^{-1} (neat) 3301, 3010, 2978, 1750, 1659, 1518, 1479, 1451, 1392, 1367, 1178, 1152, 978, 880, 755, 667, 621; δ_H (400 MHz, D_6-DMSO (* denotes major rotamer)) 10.81 (1H, s, N*H*), 8.65 (1H, m, N*H*), 8.08 (1H, dd, *J* 13.7 and 8.3, N*H*), 7.90 (2H, d, *J* 7.3, 2 x Ar*H*), 7.71 (2H, d, *J* 7.6, 2 x Ar*H*), 7.60 (1H, d, *J* 7.8, 32-*H*), 7.42 (2H, dd, *J* 7.58, 2 x Ar*H*), 7.33 (3H, m, 2 x Ar*H* and 35-*H*), 7.14 (1H, d, *J* 2.0, 30-*H*), 7.05 (1H, dd, *J* 7.3, 34-*H*), 6.97 (1H, dd, *J* 7.3, 33-*H*), 4.66 (1H, m, 28-*H*), 4.38 (2H, d, *J* 7.3, OCH_2CH), 4.27 (1H, t, *J* 7.1, OCH_2C*H*), 4.01-3.91 (2H, m, 39-*H*), 3.85 and 3.68* (2H, d, *J* 16.7, 26-*H*), 3.18 (1H, dd, *J* 14.4 and 3.4, 29-*H*H), 2.95 (1H, dd, *J* 14.4 and 9.0, 29-H*H*), 2.61* and 2.59 (3H, s, 40-*H*), 1.36 and 1.22* (9H, s, $CO_2C(CH_3)_3$); δ_C (400 MHz, D_6-DMSO (chemical shifts reported for the major rotamer only)) 172.2 (27-*C*), 169.8 (38-*C*), 168.3 (25-*C*), 155.1 (41-*C*), 143.5 (2 x $C_{(Ar)}$), 140.7 (2 x $C_{(Ar)}$), 136.1 (36-*C*), 127.8 (2 x $CH_{(Ar)}$), 127.3 (37-*C*), 127.2 (2 x $CH_{(Ar)}$), 125.3 (2 x $CH_{(Ar)}$), 123.6 (30-*C*), 120.8 (24-*C*), 120.2 (2 x $CH_{(Ar)}$), 118.4 (32-*C*), 118.2 (33-*C*), 111.3 (35-*C*), 109.9 (31-*C*), 78.5

($CO_2C(CH_3)_3$), 66.2 (O*C*H$_2$CH), 53.0 (28-*C*), 51.4 (26-*C*), 46.1 (OCH$_2$*C*H), 40.8 (29-*C*), 35.0 (40-*C*), 28.1 (29-*C*), 27.8 ($CO_2C(CH_3)_3$); δ_H (500 MHz, D_6-DMSO, 363 K) 10.55 (1H, s, N*H*), 8.17 (1H, m, N*H*), 7.86 (2H, m, 2 x Ar*H*), 7.68 (2H, d, *J* 7.6, 2 x Ar*H*), 7.60-7.56 (2H, m, 32-*H* and N*H*), 7.42 (2H, dd, *J* 7.4, 2 x Ar*H*), 7.35-7.31 (3H, m, 2 x Ar*H* and 35-*H*), 7.12 (1H, d, *J* 2.5, 30-*H*), 7.05 (1H, dd, *J* 7.5, 34-*H*), 6.97 (1H, dd, *J* 7.4, 33-*H*), 4.70 (1H, m, 28-*H*), 4.40 (2H, d, *J* 7.1, OC*H*$_2$CH), 4.27 (1H, t, *J* 7.0, OCH$_2$C*H*), 3.94 (2H, d, *J* 6.0, 39-*H*), 3.75 (2H, m, 26-*H*), 3.23 (1H, dd, *J* 14.7 and 5.3, 29-*H*H), 3.02 (1H, m, 29-H*H*), 2.67 (3H, s, 40-*H*), 1.34 (9H, s, $CO_2C(CH_3)_3$); *m/z* (ES^+) 633 (MNa^+, 100%), 611 (MH^+, 35%); *m/z* HRMS (ES^+) MNa^+ calculated for $C_{35}H_{38}O_6N_4Na$ 633.2684, observed 633.2679.

(9*H*-Fluoren-9-yl)methyl 2-((*R*)-2-(2-((*Z*)-2-((*R*)-3-((*tert*-butoxycarbonyl)amino)-5-oxopyrrolidin-2-ylidene)-*N*-methylacetamido)acetamido)-3-(1*H*-indol-3-yl)propanamido)acetate (40a)

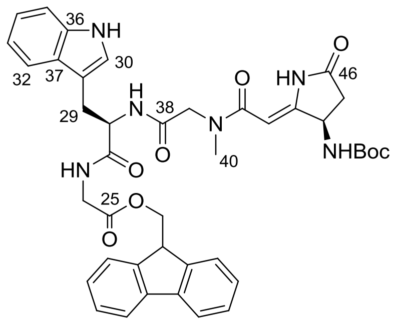

Hydrogen chloride (1.20 mL of a 4 M solution in dioxane, 4.92 mmol) was added to a solution of protected tripeptide **39** (200 mg, 1.47 mmol) in CH_2Cl_2 (1.64 mL) at room temperature. The mixture was stirred for 2 h, concentrated *in vacuo* and the residue then dissolved in toluene and again concentrated *in vacuo* (x 2) to afford the corresponding amine salt, as an amorphous colorless solid, which was used directly in the subsequent step. Carboxylic acid *epi*-**21** (88.3 mg, 0.34 mmol) was dissolved in CH_2Cl_2 (1.50 mL) and cooled to 0 ^oC. 2,6-Lutidine (153 μL, 1.31 mmol) and PyAOP (179 mg, 0.34 mmol) were then added sequentially. The mixture was stirred at 0 ^oC for 2 h. The amine salt was dissolved in CH_2Cl_2 (2.64 mL) and added to the solution. The mixture was warmed slowly to room temperature, stirred for 16 h and then concentrated *in vacuo*. Purification by flash column chromatography (100% EtOAc-2% MeOH/ EtOAc) afforded tetrapeptide **40a** (196 mg, 80% over 2 steps) as a colorless solid; R_f: 0.30 (EtOAc); m.p. 85-87 ^oC; $[\alpha]^{25}_D$ +59.1 (*c* 0.43, acetone); ν_{max}/cm^{-1} (neat) 3299, 2056, 1960, 1741, 1656, 1549, 1492, 1366, 1342, 1249, 1205, 1166, 758, 738,

641, 618; δ_H (500 MHz, D_6-DMSO (* denotes major rotamer)) 10.80 (1H, s, N*H*), 10.38 and 10.34* (1H, s, N*H*), 8.62 and 8.56* (1H, br s, N*H*), 8.24 and 8.15* (1H, d, *J* 7.9, N*H*), 7.90 (2H, d, *J* 7.6, 2 x Ar*H*), 7.71 (2H, d, *J* 7.4, 2 x Ar*H*), 7.60 (1H, d, *J* 7.7, 32-*H*), 7.42 (1H, d, *J* 8.2, N*H*), 7.43 (2H, dd, *J* 7.4, 2 x Ar*H*), 7.34 (2H, dd, *J* 7.4, 2 x Ar*H*), 7.31 (1H, d, *J* 8.0, 35-*H*), 7.14 (1H, s, 30-*H*), 7.05 (1H, ddd, *J* 7.9 and 0.8, 34-*H*), 6.97 (1H, ddd, *J* 7.9 and 0.6, 33-*H*), 5.36* and 5.25 (1H, s, 42-*H*), 4.79 (1H, m, 44-*H*), 4.63 (1H, m, 28-*H*), 4.39 (2H, d, *J* 7.3, OCH_2CH), 4.27 (1H, t, *J* 7.3, OCH_2CH), 4.02-3.97 (3H, m, 26-*H* and 39-*H*H), 3.88 (1H, d, *J* 15.9, 39-H*H*), 3.20 (1H, dd, *J* 14.3 and 3.5, 29-*H*H), 2.95 (1H, dd, *J* 14.3 and 9.6, 29-H*H*), 2.77* and 2.62 (3H, br s, 40-*H*), 2.73 (1H, m, 45-*H*H), 2.30 (1H, dd, *J* 17.5 and 5.2, 45-H*H*), 1.40* and 1.30 (9H, s, $CO_2C(CH_3)_3$); δ_C (126 MHz, D_6-DMSO (chemical shifts reported for the major rotamer only)) 174.6 (46-*C*), 172.1 (38- or 27-*C*), 169.8 (38- or 27-*C*), 168.2 (41-*C*), 162.3 (25-*C*), 157.8 (43-*C*), 155.4 (*C*(O)), 143.5 ($C_{(Ar)}$), 140.7 ($C_{(Ar)}$), 136.0 ($C_{(Ar)}$), 127.8 ($CH_{(Ar)}$), 127.3 ($C_{(Ar)}$), 127.2 ($CH_{(Ar)}$), 125.3 ($CH_{(Ar)}$), 123.7 (30-*C*), 120.8 (34-*C*), 120.2 ($CH_{(Ar)}$), 118.4 (32-*C*), 118.2 (33-*C*), 111.3 (35-*C*), 110.0 ($C_{(Ar)}$), 87.4 (42-*C*), 78.7 ($CO_2C(CH_3)_3$), 66.2 (OCH_2CH), 53.2 (28-*C*), 49.9 (39-*C*), 48.4 (44-*C*), 46.1 (OCH_2CH), 40.8 (26-*C*), 36.0 (40-*C*), 34.3 (45-*C*), 28.1 ($CO_2C(CH_3)_3$), 27.8 (29-*C*); δ_H (500 MHz, D_6-DMSO, 363 K) 10.54 (1H, s, N*H*), 10.26 (1H, s, N*H*), 8.14 (1H, m, N*H*), 7.87 (2H, d, *J* 7.6, 2 x Ar*H*), 7.73 (1H, d, *J* 7.6, N*H*), 7.68 (2H, d, *J* 7.6, 2 x Ar*H*), 7.57 (1H, d, *J* 7.8, 32-*H*), 7.42 (2H, dd, *J* 7.5, 2 x Ar*H*), 7.36-7.32 (3H, m, 2 x Ar*H* and 35-*H*), 7.13-7.12 (2H, m, 30-*H* and N*H*), 7.05 (1H, dd, *J* 7.8, 34-*H*), 6.97 (1H, dd, *J* 7.8, 33-*H*), 5.37 (1H, s, 42-*H*), 4.75 (1H, m, 44-*H*), 4.67 (1H, m, 28-*H*), 4.42-4.39 (2H, m, OCH_2CH), 4.27 (1H, t, *J* 7.0, OCH_2CH), 3.99-3.91 (4H, m, 26- and 39-*H*), 3.24 (1H, dd, *J* 14.8 and 5.3, 29-*H*H), 3.04 (1H, m, 29-H*H*), 2.79 (3H, s, 40-*H*), 2.73 (1H, dd, *J* 17.6 and 7.8, 45-*H*H), 2.34 (1H, dd, *J* 17.6 and 5.5, 45-H*H*), 1.40 (9H, s, $CO_2C(CH_3)_3$); *m/z* (ES^+) 749 (MH^+, 100%), 766 (MNa^+, 50%); *m/z* HRMS (ES^+) MH^+ calculated for $C_{41}H_{45}O_8N_6$ 749.3293, observed 749.3289.

(9*H*-Fluoren-9-yl)methyl 2-((*R*)-2-(2-((*Z*)-2-((*R*)-3-((2*S*,3*R*,4*S*,5*S*,6*S*,*E*)-3-((*R*)-4-((((9*H*-fluoren-9-yl)methoxy)carbonyl)amino)-3-((*tert*-butyldimethylsilyl)oxy)butanamido)-2,5-bis((*tert*-butyldimethylsilyl)oxy)-4-hydroxy-12-(4-methoxyphenyl)-6-methyldodec-11-enamido)-5-oxopyrrolidin-2-ylidene)-*N*-methylacetamido)acetamido)-3-(1*H*-indol-3-yl)propanamido)acetate (41)

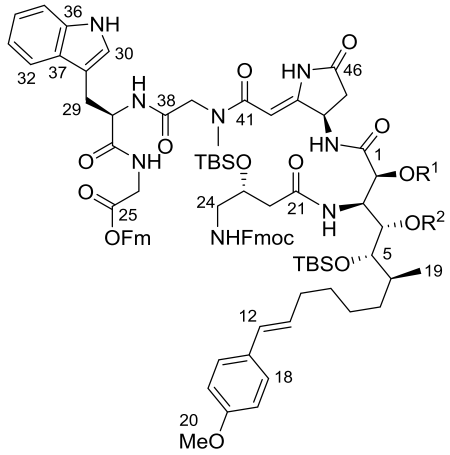

Trifluoroacetic acid (0.38 mL, 4.90 mmol) and triethylsilane (0.16 mL, 0.98 mmol) were added sequentially to a solution of tetrapeptide **40a** (367 mg, 0.49 mmol) in CH_2Cl_2 (5.00 mL) at 0 ^oC. The reaction was warmed to room temperature and stirred for 2 h. The solution was then concentrated *in vacuo* and the residue then dissolved in toluene and again concentrated *in vacuo* (x 2) to afford the corresponding amine salt, as a colorless solid, which was used directly in the subsequent step. HATU (81.0 mg, 0.21 mmol), 2,6-lutidine (76.0 μL, 0.65 mmol) and the amine salt of the deprotected tetrapeptide (162 mg, 0.21 mmol) were added sequentially to a solution of carboxylic acid **35** (174 mg, 0.15 mmol) in DMF (1.63 mL) at 0 ^oC. The mixture was warmed to room temperature and stirred for 16 h. The solution was then diluted with EtOAc and washed with brine solution (x 2), dried (Na_2SO_4), filtered and concentrated *in vacuo*. Purification by flash column chromatography (60-70-80-100% EtOAc/petrol) afforded linear hexapeptides **41** (112 mg, ca. 45% from acid **35**) and **42** (54.1 mg, 21% from acid **35**) as colorless solids. Data for the linear hexapeptide **41** (contaminated with traces of *N,N'*-dimethylurea): R_f 0.60 (EtOAc); m.p. 93-95 ^oC; ν_{max} /cm^{-1} (neat) 3314, 2930, 2856, 1657, 1511, 1451, 1382, 1249, 1198, 1100, 877, 834, 777, 759, 740, 669; δ_H (500 MHz, D_6-DMSO) 10.81 (1H, s, N*H*), 10.38 (1H, s, N*H*), 8.56 (1H, br s, N*H*), 8.38 (1H, d, *J* 5.5, N*H*), 8.16 (1H, d, *J* 7.4, N*H*), 7.88 (2H, d, *J* 7.6, 2 x Ar*H*), 7.86 (2H, d, *J* 7.7, 2 x Ar*H*), 7.71 (2H, d, *J* 7.6, 2 x Ar*H*), 7.68 (2H, dd,

J 7.6 and 3.9, 2 x Ar*H*), 7.60 (1H, d, *J* 7.6, 32-*H*), 7.43-7.25 (12H, m, N*H*, 14-, 18-, 35-*H* and 8 x Ar*H*), 7.16 (1H, s, 30-*H*), 7.11 (1H, br s, N*H*), 7.04 (1H, dd, *J* 7.3, 34-*H*), 6.97 (1H, dd, *J* 7.6, 33-*H*), 6.83 (2H, d, *J* 8.7, 15- and 17-*H*), 6.27 (1H, d, *J* 15.8, 12-*H*), 6.06 (1H, dt, *J* 15.8 and 6.6, 11-*H*), 5.59 (1H, br s, O*H*), 5.33 (1H, s, 42-*H*), 4.83 (1H, m, 44-*H*), 4.65 (1H, m, 28-*H*), 4.39-4.37 (2H, m, OCH_2CH), 4.30 (1H, d, *J* 3.6, OC*H*HCH), 4.28-4.23 (2H, m, 2-*H* and OCH_2C*H*), 4.21-4.17 (2H, m, OCH*H*CH and OCH_2C*H*), 4.12 (1H, m, 3-*H*), 3.98-3.89 (5H, m, 23-, 26- and 39-*H*), 3.82 (1H, m, 4-*H*), 3.71 (3H, s, 20-*H*), 3.33 (1H, m, 5-*H*), 3.22 (1H, d, *J* 11.5, 29-*H*H), 3.02-2.95 (3H, m, 29-H*H* and 24-*H*), 2.82 (3H, s, 40-*H*), 2.63 (1H, m, 45-*H*H), 2.54 (1H, m, 45-H*H*), 2.27 (1H, m, 22-*H*H), 2.17-2.10 (3H, m, 22-H*H* and 10-*H*), 1.65-1.56 (2H, m, 6-*H* and 7-*H*H), 1.41-1.30 (3H, m, 8-*H*H and 9-*H*), 1.12 (1H, m, 8-H*H*), 0.88-0.78 (31H, m, 3 x SiC(CH_3)$_3$, 19-*H* and 7-H*H*), 0.14, 0.10, 0.10, 0.05, 0.03, 0.03 (6 x 3H, s, 6 x SiCH_3); δ_C (126 MHz, D_6-DMSO) 175.3 (46-*C*), 172.9 (1-*C*), 172.0 (27-*C*), 169.8 (25-*C*), 168.3 (38-*C*), 168.1 (21-*C*), 167.9 (41-*C*), 158.3 (16-*C*), 157.1 (43-*C*), 156.4 (*C*(O)), 143.9 ($C_{(Ar)}$), 143.8 ($C_{(Ar)}$), 143.5 (2 x $C_{(Ar)}$), 140.7 (4 x $C_{(Ar)}$), 136.0 (36-*C*), 130.0 (13-*C*), 129.1 (12-*C*), 128.1 (11-*C*), 127.8 ($CH_{(Ar)}$), 127.6 (2 x $CH_{(Ar)}$), 127.4 (37-*C*), 127.2 ($CH_{(Ar)}$), 127.0 ($CH_{(Ar)}$), 126.9 (14- and 18-*C*), 125.3 (2 x $CH_{(Ar)}$), 125.2 ($CH_{(Ar)}$), 125.1 ($CH_{(Ar)}$), 123.7 (30-*C*), 120.8 (34-*C*), 120.2 ($CH_{(Ar)}$), 120.1 ($CH_{(Ar)}$), 118.4 (32-*C*), 118.2 (33-*C*), 113.9 (15- and 17-*C*), 111.3 (35-*C*), 110.0 (31-*C*), 88.0 (42-*C*), 78.4 (5-*C*), 72.8 (4-*C*), 70.1 (3-*C*), 68.0 (23-*C*), 66.2 (OCH_2CH), 65.6 (OCH_2CH), 55.0 (20-*C*), 53.3 (28-*C*), 52.9 (2-*C*), 50.0 (39-*C*), 47.3 (44-*C*), 46.7 (OCH_2*C*H), 46.1 (OCH_2*C*H), 45.6 (24-*C*), 42.1 (22-*C*), 40.8 (26-*C*), 36.1 (40-*C*), 33.6 (45-*C*), 33.0 (7-*C*), 32.6 (10-*C*), 29.4 (9- and 6-*C*), 27.8 (29-*C*), 26.1 (SiC(CH_3)$_3$), 25.9 (SiC(CH_3)$_3$), 25.7 (SiC(CH_3)$_3$), 25.7 (8-*C*), 18.0 (Si*C*(CH_3)$_3$), 17.9 (Si*C*(CH_3)$_3$), 17.6 (Si*C*(CH_3)$_3$), 16.4 (19-*C*), −3.2 (SiCH_3), −3.3 (SiCH_3), −4.9 (SiCH_3), −5.0 (2 x SiCH_3), −5.3 (SiCH_3); *m/z* HRMS (ES^+) MH^+ calculated for $C_{93}H_{125}N_8O_{15}Si_3$ 1678.8600, observed 1678.8595.

(9*H*-Fluoren-9-yl)methyl 2-((*R*)-2-(2-((*Z*)-2-((*R*)-3-((2*S*,3*R*,4*S*,5*S*,6*S*,*E*)-3-((*R*)-4-((((9*H*-fluoren-9-yl)methoxy)carbonyl)amino)-3-((*tert*-butyldimethylsilyl)oxy)butanamido)-2,4,5-tris((*tert*-butyldimethylsilyl)oxy)-12-(4-methoxyphenyl)-6-methyldodec-11-enamido)-5-oxopyrrolidin-2-ylidene)-*N*-methylacetamido)acetamido)-3-(1*H*-indol-3-yl)propanamido)acetate (42)

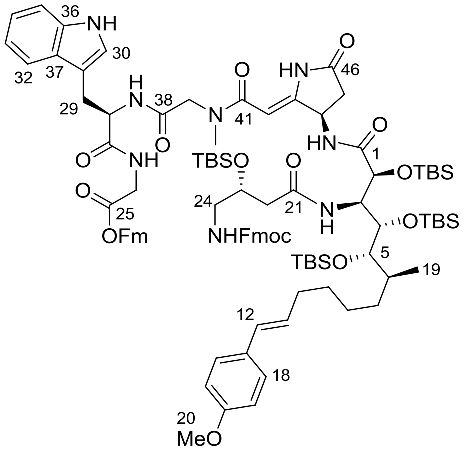

R_f: 0.80 (EtOAc); m.p. 107-109 °C; $[\alpha]^{25}_D$ +32.3 (*c* 1.50, EtOAc); ν_{max}/cm^{-1} (neat) 2928, 2856, 1657, 1511, 1463, 1359, 1250, 1176, 1100, 834, 776, 759, 740, 671, 619; δ_H (500 MHz, D_6-DMSO) 10.81 (1H, s, N*H*), 10.46 (1H, s, N*H*), 8.54 (1H, br s, N*H*), 8.10 (1H, d, *J* 7.6, N*H*), 7.89-7.84 (5H, m, N*H* and 4 x Ar*H*), 7.71-7.65 (5H, m, N*H* and 4 x Ar*H*), 7.59 (1H, d, *J* 7.7, 32-*H*), 7.43-7.25 (12H, m, N*H*, 14-, 18-, 35-*H* and 8 x Ar*H*), 7.15 (1H, s, 30-*H*), 7.03 (1H, dd, *J* 7.4, 34-*H*), 6.96 (1H, dd, *J* 7.4, 33-*H*), 6.84 (2H, d, *J* 8.7, 15- and 17-*H*), 6.28 (1H, d, *J* 15.8, 12-*H*), 6.07 (1H, dt, *J* 15.8 and 6.8, 11-*H*), 5.21 (1H, br s, 42-*H*), 4.72 (1H, m, 44-*H*), 4.64 (1H, m, 28-*H*), 4.38-4.34 (2H, m, OCH_2CH), 4.31-4.25 (3H, m, 2- or 4-*H*, OC*H*HCH and OCH_2C*H*), 4.22-4.17 (2H, m, OCH*H*CH and OCH_2C*H*), 4.15-4.12 (2H, m, 39-*H*H and 3-*H*), 4.02 (1H, m, 23-*H*), 3.97 (2H, d, *J* 4.7, 26-*H*), 3.74 (1H, m, 39-H*H*), 3.72 (1H, m, 2- or 4-*H*), 3.71 (3H, s, 20-*H*), 3.48 (1H, m, 5-*H*), 3.21 (1H, d, *J* 11.0, 29-*H*H), 3.10 (1H, m, 24-*H*H), 2.99-2.94 (2H, m, 29-H*H* and 24-H*H*), 2.79 (3H, s, 40-*H*), 2.63-2.52 (2H, m, 45-*H*), 2.25 (1H, m, 22-*H*H), 2.12-2.08 (3H, m, 22-H*H* and 10-*H*), 1.61-1.56 (2H, m, 6-*H* and 7-*H*H), 1.42-1.31 (3H, m, 8-*H*H and 9-*H*), 1.10 (1H, m, 8-H*H*), 0.86-0.78 (40H, m, 4 x SiC(CH_3)$_3$, 19-*H* and 7-H*H*), 0.17, 0.12, 0.07, 0.03, 0.03, 0.02, −0.02, −0.04 (8 x 3H, s, 8 x SiCH_3); δ_C (126 MHz, D_6-DMSO) 175.3 (46-*C*), 172.1 (27-*C*), 171.6 (1-*C*), 169.8 (25-*C*), 168.2 (38-*C*), 167.8 (41- and 21-*C*), 158.3 (16-*C*), 157.0 (*C*(O) and 43-*C*), 143.8 ($C_{(Ar)}$), 143.7 ($C_{(Ar)}$), 143.5 (2 x $C_{(Ar)}$), 140.7 (4 x $C_{(Ar)}$), 136.0 (36-*C*),

130.0 (13-*C*), 129.1 (12-*C*), 128.0 (11-*C*), 127.8 ($CH_{(Ar)}$), 127.6 (2 x $CH_{(Ar)}$), 127.4 (37-*C*), 127.2 ($CH_{(Ar)}$), 126.9 ($CH_{(Ar)}$, 14- and 18-*C*), 125.3 (2 x $CH_{(Ar)}$), 125.2 ($CH_{(Ar)}$), 125.1 ($CH_{(Ar)}$), 123.7 (30-*C*), 120.8 (34-*C*), 120.2 (2 x $CH_{(Ar)}$), 118.4 (32-*C*), 118.2 (33-*C*), 113.9 (15- and 17-*C*), 111.3 (35-*C*), 110.0 (31-*C*), 87.6 (42-*C*), 72.3 (2- or 4-*C*), 69.7 (5-*C*), 68.0 (23-*C*), 66.2 (OCH_2CH), 65.8 (OCH_2CH), 55.0 (20-*C*), 53.7 (3-*C*), 53.2 (28-*C*), 51.9 (2- or 4-*C*), 50.1 (39-*C*), 47.7 (44-*C*), 46.6 (OCH_2*C*H), 46.1 (OCH_2*C*H), 45.3 (24-*C*), 41.6 (22-*C*), 40.9 (26-*C*), 35.9 (40-*C*), 33.5 (7-*C*), 33.2 (45-*C*), 32.6 (10-*C*), 29.4 (9-*C*), 29.1 (6-*C*), 27.7 (29-*C*), 26.1 (8-*C*), 25.8 (SiC(CH_3)$_3$), 25.8 (SiC(CH_3)$_3$), 25.7 (SiC(CH_3)$_3$), 18.1 (Si*C*(CH_3)$_3$), 17.9 (Si*C*(CH_3)$_3$), 17.7 (Si*C*(CH_3)$_3$), 16.1 (19-*C*), −2.2 (SiCH_3), −3.5 (SiCH_3), −4.2 (SiCH_3), −4.9 (3 x SiCH_3), −5.2 (2 x SiCH_3); *m/z* HRMS (ES^+) MH^+ calculated for $C_{99}H_{139}N_8O_{15}Si_4$ 1792.9465, observed 1792.9447.

(3a*R*,6*S*,7*R*,11*R*,18*R*,*Z*)-18-((1*H*-indol-3-yl)methyl)-6,11-bis((*tert*-butyldimethylsilyl)oxy)-7-((1*S*,2*S*,3*S*,*E*)-2-((*tert*-butyldimethylsilyl)oxy)-1-hydroxy-9-(4-methoxyphenyl)-3-methylnon-8-en-1-yl)-22-methyl-3a,4,7,8,10,11,12,13,15,16,18,19,21,22-tetradecahydropyrrolo[2,3-*m*][1,4,7,10,15,19]hexaazacyclotricosine-2,5,9,14,17,20,23(1*H*,3*H*,6*H*)-heptaone (43)

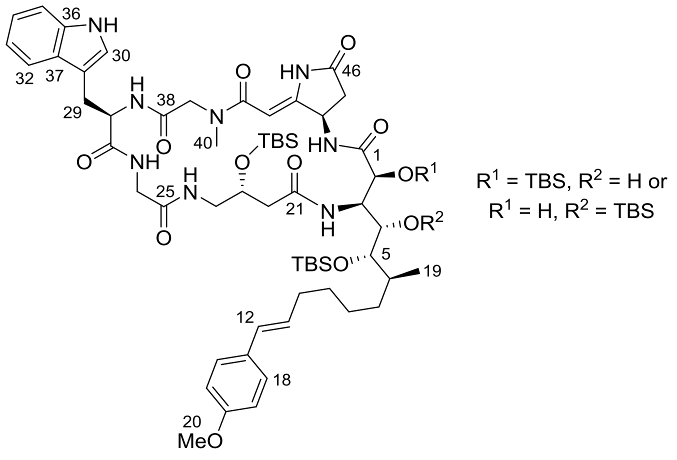

Piperidine (0.25 mL, 2.53 mmol) was added to a solution of linear hexapeptide **41** (111 mg, 66.0 μmol) in CH_2Cl_2 (5.00 mL) at room temperature. The reaction was stirred for 2 h. The solution was then concentrated *in vacuo* and the crude mixture was dried under high vacuum for 2 h at 40 ^oC. Purification by flash column chromatography (5-10-20-25-30% MeOH/$CHCl_3$) afforded piperidine salt of linear amino acid as a colorless solid. The piperidine salt of amino acid was dissolved in DMF (66.0 mL) and cooled to 0 ^oC. PyAOP

(344 mg, 0.66 mmol), HOAt (90.0 mg, 0.66 mmol) and *N*,*N*-diisopropylethylamine (115 μL, 0.66 mmol) were then added sequentially. The mixture was warmed slowly to room temperature, stirred for 5 days and concentrated *in vacuo*. The residue was then redissolved in 2% MeOH/$CHCl_3$, washed with H_2O, dried (Na_2SO_4), filtered and concentrated *in vacuo*. Purification by flash column chromatography (EtOAc-2% MeOH/EtOAc) afforded cyclic hexapeptide **43** (45.6 mg, 55% over 2 steps) as a colorless solid; R_f 0.30 (2% MeOH/EtOAc); m.p. 156-158 ^oC; $[\alpha]^{25}_D$ −18.6 (*c* 3.25, EtOAc); ν_{max} /cm^{-1} (neat) 2981, 2889, 1658, 1512, 1462, 1382, 1250, 1148, 1081, 955, 877, 833, 776, 743, 668; δ_H (500 MHz, D_4-MeOD) 7.62 (1H, d, *J* 8.0, 32-*H*), 7.36 (1H, d, *J* 8.0, 35-*H*), 7.22 (1H, s, 30-*H*), 7.20 (2H, d, *J* 8.7, 14- and 18-*H*), 7.11 (1H, dd, *J* 8.0, 34-*H*), 7.02 (1H, dd, *J* 7.3, 33-*H*), 6.72 (2H, d, *J* 8.7, 15- and 17-*H*), 6.39 (1H, d, *J* 9.0, N*H* or O*H*), 6.26 (1H, d, *J* 15.8, 12-*H*), 6.03 (1H, dt, *J* 15.8 and 7.1, 11-*H*), 5.60 (1H, d, *J* 1.3, 42-*H*), 5.24 (1H, m, 44-*H*), 4.54 (1H, dd, *J* 10.4 and 3.2, 28-*H*), 4.50-4.42 (3H, m, 26-*H*H, 23- and 4-*H*), 4.36 (1H, s, 2-*H*), 4.25 (1H, d, *J* 14.8, 39-*H*H), 4.02 (1H, dd, *J* 9.6 and 2.1, 3-*H*), 3.59 (3H, s, 20-*H*), 3.51 (1H, dd, *J* 9.1 and 2.1, 5-*H*), 3.45 (1H, m, 26-H*H*), 3.42 (1H, m, 29-H*H*), 3.26 (3H, s, 40-*H*), 3.15 (1H, d, *J* 14.8, 39-H*H*), 3.13-3.08 (2H, m, 29-H*H* and 24-*H*H), 2.78 (1H, d, *J* 14.2, 24-H*H*), 2.73 (1H, dd, *J* 18.0 and 9.6, 45-*H*H), 2.64 (1H, dd, *J* 18.0 and 6.5, 45-H*H*), 2.50 (1H, dd, *J* 14.3 and 4.7, 22-*H*H), 2.18-2.08 (3H, m, 22-H*H* and 10-*H*), 1.73-1.64 (2H, m, 6-*H* and 7-*H*H), 1.45-1.42 (2H, m, 9-*H*H and 8-*H*H), 1.34 (1H, m, 9-H*H*), 1.23 (1H, m, 8-H*H*), 0.99 (3H, d, *J* 6.6, 19-*H*), 0.97 (9H, s, SiC(CH_3)$_3$), 0.94 (9H, s, SiC(CH_3)$_3$), 0.91 (1H, m, 7-H*H*), 0.85 (9H, s, SiC(CH_3)$_3$), 0.22, 0.18, 0.17, 0.14, 0.13, 0.07 (6 x 3H, s, 6 x SiCH_3); δ_C (126 MHz, D_4-MeOD) 177.3 (1- or 47-*C*), 177.2 (1- or 47-*C*), 175.1 (38-*C*), 174.1 (27-*C*), 172.8 (25-*C*), 170.9 (41-*C*), 169.8 (21-*C*), 160.3 (16-*C*), 157.6 (43-*C*), 138.4 (36-*C*), 132.1 (13-*C*), 130.8 (12-*C*), 129.5 (11-*C*), 128.4 (37-*C*), 128.3 (14- and 18-*C*), 124.8 (30-*C*), 122.8 (34-*C*), 120.0 (33-*C*), 119.5 (32-*C*), 115.0 (15- and 17-*C*), 112.6 (35-*C*), 111.2 (31-*C*), 91.5 (42-*C*), 81.1 (5-*C*), 75.4 (3-*C*), 71.8 (2-*C*), 68.1 (23-*C*), 57.8 (28-*C*), 55.7 (20-*C*), 55.2 (4-*C*), 54.4 (39-*C*), 48.6 (44-*C*), 48.3 (24-*C*), 46.4 (22-*C*), 43.1 (26-*C*), 39.9 (40-*C*), 35.7 (6-*C*), 35.0 (7-*C*), 34.7 (45-*C*), 34.6 (10-*C*), 31.0 (9-*C*), 27.8 (29-*C*), 27.5 (8-*C*), 27.1 (SiC(*C*H$_3$)$_3$), 26.8 (SiC(*C*H$_3$)$_3$), 26.6 (SiC(*C*H$_3$)$_3$), 19.4 (Si*C*(CH$_3$)$_3$), 19.1 (Si*C*(CH$_3$)$_3$), 19.0 (Si*C*(CH$_3$)$_3$), 17.6 (19-*C*), −2.3 (Si*C*H$_3$), −2.8 (Si*C*H$_3$), −3.7 (Si*C*H$_3$), −3.9 (Si*C*H$_3$), −4.2 (Si*C*H$_3$), −5.1 (Si*C*H$_3$); *m/z* HRMS (ES^+) MNa^+ calculated for $C_{64}H_{102}N_8O_{12}Si_3Na$ 1281.6817, observed 1281.6798.

(3a*R*,6*S*,7*R*,11*R*,18*R*,*Z*)-18-((1*H*-Indol-3-yl)methyl)-6,11-bis((*tert*-butyldimethylsilyl)oxy)-7-((5*S*,6*S*)-6-((*S*,*E*)-8-(4-methoxyphenyl)oct-7-en-2-yl)-

2,2,3,3,8,8,9,9-octamethyl-4,7-dioxa-3,8-disiladecan-5-yl)-22-methyl-3a,4,7,8,10,11,12,13,15,16,18,19,21,22-tetradecahydropyrrolo[2,3-*m*][1,4,7,10,15,19]hexaazacyclotricosine-2,5,9,14,17,20,23(1*H*,3*H*,6*H*)-heptaone (44)

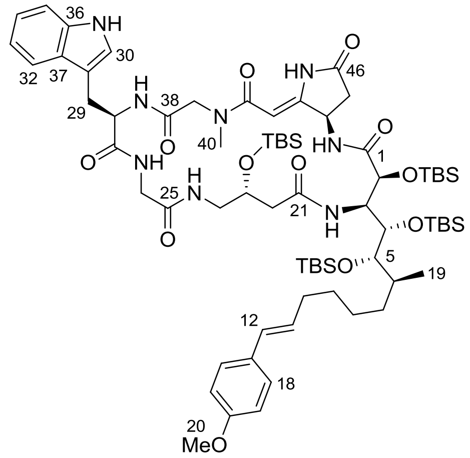

Piperidine (0.25 mL, 2.53 mmol) was added to a solution of linear hexapeptide **42** (54.0 mg, 30.0 μmol) in CH_2Cl_2 (5.00 mL) at room temperature. The reaction was stirred for 2 h. The solution was then concentrated *in vacuo* and the crude mixture was dried under high vacuum for 2 h at 40 ^oC. Purification by flash column chromatography (5-10-20-25-30% MeOH/$CHCl_3$) afforded piperidine salt of linear amino acid as a colorless solid. The piperidine salt of amino acid was dissolved in DMF (30.0 mL) and cooled to 0 ^oC. PyAOP (156 mg, 0.30 mmol), HOAt (40.8 mg, 0.30 mmol) and *N,N*-diisopropylethylamine (52.0 μL, 0.30 mmol) were then added sequentially. The mixture was warmed slowly to room temperature, stirred for 5 days and concentrated *in vacuo*. The residue was then redissolved in 2% MeOH/$CHCl_3$, washed with H_2O, dried (Na_2SO_4), filtered and concentrated *in vacuo*. Purification by flash column chromatography (EtOAc-2% MeOH/EtOAc) afforded cyclic hexapeptide **44** (20.2 mg, 49% over 2 steps) as a colorless solid; R_f 0.50 (2% MeOH/EtOAc); m.p. 165-167 ^oC; $[\alpha]^{25}_D$ −12.9 (*c* 1.67, EtOAc); ν_{max} /cm^{-1} (neat) 2930, 2857, 1740, 1656, 1511, 1462, 1250, 1176, 1099, 834, 776, 741, 671; δ_H (500 MHz, D_4-MeOD) 7.63 (1H, d, *J* 7.9, 32-*H*), 7.36 (1H, d, *J* 8.0, 35-*H*), 7.22 (1H, s, 30-*H*), 7.18 (2H, d, *J* 8.5, 14- and 18-*H*), 7.12 (1H, dd, *J* 8.0, 34-*H*), 7.03 (1H, dd, *J* 7.9, 34-*H*), 6.89 (2H, d, *J* 8.5, 15- and 17-*H*), 6.26 (1H, d, *J* 15.8, 12-*H*), 6.03 (1H, dt, *J* 15.8 and 7.1, 11-*H*), 5.90 (1H, br s, N*H*), 5.66 (1H, s, 42-*H*), 5.32 (1H, dd, *J* 7.4, 44-*H*), 4.56-4.46 (4H, m, 23-, 28-, 4- and 2-*H*), 4.40 (1H, d, *J* 17.3, 26-*H*H), 4.24 (1H, d, *J* 14.8, 39-*H*H), 3.85 (1H, dd, *J* 9.9 and 2.4, 3-*H*), 3.61 (1H, m,

5-*H*), 3.59 (3H, s, 20-*H*), 3.43 (1H, dd, *J* 15.1 and 3.0, 29-*H*H), 3.37 (1H, d, *J* 17.3, 26-H*H*), 3.26 (3H, s, 40-*H*), 3.12 (1H, dd, *J* 15.1 and 10.4, 29-H*H*), 3.07 (1H, m, 24-*H*H), 2.97 (1H, m, 39-H*H*), 2.82 (1H, m, 24-H*H*), 2.79 (1H, dd, *J* 17.3 and 9.6, 45-*H*H), 2.56 (1H, dd, *J* 15.1 and 4.6, 22-*H*H), 2.50 (1H, dd, *J* 17.3 and 7.4, 45-H*H*), 2.21-2.01 (3H, m, 22-H*H* and 10-*H*), 1.74-1.68 (2H, m, 6-*H* and 7-*H*H), 1.42-1.39 (2H, m, 9-*H*H and 8-*H*H), 1.33 (1H, m, 9-H*H*), 1.23 (1H, m, 8-H*H*), 1.03 (9H, s, SiC(CH_3)$_3$), 1.00 (3H, d, *J* 6.6, 19-*H*), 0.96 (9H, s, SiC(CH_3)$_3$), 0.96 (9H, s, SiC(CH_3)$_3$), 0.90 (1H, m, 7-H*H*), 0.86 (9H, s, SiC(CH_3)$_3$), 0.23, 0.23, 0.22, 0.20, 0.16, 0.15, 0.13, 0.09 (8 x 3H, s, 8 x SiCH_3); δ_C (126 MHz, D_4-MeOD) 176.3 (46-*C*), 175.3 (1-*C*), 174.7 (38-*C*), 174.1 (27-*C*), 172.4 (25-*C*), 170.8 (41-*C*), 169.3 (21-*C*), 160.3 (16-*C*), 156.3 (44-*C*), 138.5 (36-*C*), 132.1 (13-*C*), 130.9 (12-*C*), 129.4 (11-*C*), 128.5 (37-*C*), 128.3 (14- and 18-*C*), 124.8 (30-*C*), 122.8 (34-*C*), 120.0 (33-*C*), 119.5 (32-*C*), 115.0 (15- and 17-*C*), 112.6 (35-*C*), 111.1 (31-*C*), 91.6 (42-*C*), 80.5 (5-*C*), 76.9 (3-*C*), 73.7 (2- or 4-*C*), 68.3 (23-*C*), 57.8 (2- or 4-*C*), 55.9 (28-*C*), 55.7 (20-*C*), 54.1 (39-*C*), 48.3 (44-*C*), 48.1 (24-*C*), 45.5 (22-*C*), 43.2 (26-*C*), 39.7 (40-*C*), 35.5 (45-*C*), 35.0 (7-*C*), 34.8 (6- and 10-*C*), 30.9 (9-*C*), 27.9 (8- or 29-*C*), 27.8 (8- or 29-*C*), 27.1 (SiC(CH_3)$_3$), 27.0 (SiC(CH_3)$_3$), 26.8 (SiC(CH_3)$_3$), 26.7 (SiC(CH_3)$_3$), 19.4 (Si*C*(CH_3)$_3$), 19.3 (Si*C*(CH_3)$_3$), 19.3 (Si*C*(CH_3)$_3$), 19.0 (Si*C*(CH_3)$_3$), 17.6 (19-*C*), −0.9 (SiCH_3), −2.5 (SiCH_3), −3.1 (SiCH_3), −3.5 (SiCH_3), −3.7 (SiCH_3), −3.8 (SiCH_3), −3.9 (SiCH_3), −4.1 (SiCH_3); *m/z* (ES^+) 1396 (MNa^+, 100); *m/z* HRMS (ES^+) MNa^+ calculated for $C_{70}H_{116}N_8O_{12}Si_4Na$ 1395.7682, observed 1395.7639.

(3a*R*,6*S*,7*R*,11*R*,18*R*,*Z*)-18-((1*H*-Indol-3-yl)methyl)-7-((1*S*,2*S*,3*S*,*E*)-1,2-dihydroxy-9-(4-methoxyphenyl)-3-methylnon-8-en-1-yl)-6,11-dihydroxy-22-methyl-3a,4,7,8,10,11,12,13,15,16,18,19,21,22-tetradecahydropyrrolo[2,3-*m*][1,4,7,10,15,19]hexaazacyclotricosine-2,5,9,14,17,20,23(1*H*,3*H*,6*H*)-heptaone (*epi*-36)

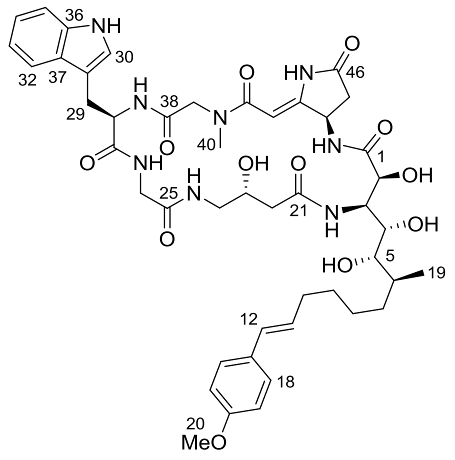

H_2O (8.40 μL, 0.47 mmol) and TASF (100 μL of a 2.35 M solution in DMF, 0.23 mmol) were added sequentially to a solution of protected cyclic peptide **44** (20.0 mg, 14.6 μmol) in DMF (0.80 mL) at 0 ^{o}C. The reaction was stirred for 7 h at 0 ^{o}C. Afterwhich TMS_2O (100 μL) was added and the solution was stirred at room temperature for 10 mins. The solution was then concentrated using high vacuum centrifugal evaporator. Purification by flash column chromatography (5-10-15% MeOH/$CHCl_3$) afforded deprotected cyclic peptide *epi*-**36** (8.5 mg, 64%) as a colorless solid; R_f 0.50 (15% MeOH/$CHCl_3$); $[\alpha]^{25}_D$ −55.5 (*c* 0.12, 1/5 0.1 M NH_4HCO_3/MeOH) (lit.[6] $[\alpha]^{25}_D$ −57.3 (*c* 0.12, 1/5 0.1 M NH_4HCO_3/MeOH)); δ_H (500 MHz, D_6-DMSO) 10.88 (1H, d, *J* 1.9, N*H*), 10.43 (1H, s, N*H*), 8.70 (1H, d, *J* 5.2, N*H*), 8.39 (1H, t, *J* 6.2, N*H*), 8.36 (1H, d, *J* 8.8, N*H*), 7.52 (1H, d, *J* 7.9, 32-*H*), 7.36-7.33 (2H, m, 35-*H* and N*H*), 7.30 (2H, d, *J* 8.8, 14- and 18-*H*), 7.23 (1H, d, *J* 2.4, 30-*H*), 7.11-7.06 (2H, m, 34-*H* and N*H*), 7.00 (1H, ddd, *J* 7.9 and 0.8, 33-*H*), 6.86 (2H, d, *J* 8.8, 15- and 17-*H*), 6.31 (1H, d, *J* 15.9, 12-*H*), 6.14-6.09 (2H, m, 11-*H* and O*H*), 5.27-5.22 (2H, m, 42- and 44-*H*), 4.98 (1H, d, *J* 5.0, O*H*), 4.56 (1H, d, *J* 16.1, 39-*H*H), 4.42 (1H, d, *J* 5.4, 2-*H*), 4.32 (1H, d, *J* 9.5, O*H*), 4.12-4.05 (3H, m, O*H*, 28- and 3-*H*), 3.74 (1H, m, 23-*H*), 3.73 (3H, s, 20-*H*), 3.68 (1H, dd, *J* 16.9 and 6.5, 26-*H*H), 3.52 (1H, dd, *J* 16.9 and 6.0, 26-H*H*), 3.44 (1H, d, *J* 16.1, 39-H*H*), 3.41 (1H, m, 4-*H*), 3.31 (1H, m, 24-*H*H), 3.23 (1H, dd, *J* 14.8 and 4.6, 29-*H*H), 3.08-3.00 (2H, m, 29-H*H* and 5-*H*), 2.98 (3H, s, 40-*H*), 2.78-2.71 (2H, m, 24-H*H* and 45-*H*H), 2.46 (1H, dd, *J* 17.8 and 5.5, 45-H*H*), 2.33

(1H, dd, *J* 14.3 and 1.9, 22-*H*H), 2.20-2.11 (2H, m, 10-*H*), 2.04 (1H, dd, *J* 14.3 and 10.4, 22-H*H*), 1.71 (1H, m, 7-*H*H), 1.59 (1H, m, 6-*H*), 1.43-1.35 (3H, m, 8-*H*H and 9-*H*), 1.21 (1H, m, 8-H*H*), 1.02 (1H, m, 7-H*H*), 0.77 (3H, d, *J* 6.6, 19-*H*); δ_C (126 MHz, D_6-DMSO) 175.0 (46-*C*), 173.7 (1-*C*), 172.5 (21-*C*), 171.5 (27-*C*), 170.9 (38-*C*), 169.0 (25-*C*), 168.1 (41-*C*), 158.3 (16-*C*), 157.5 (43-*C*), 136.1 (36-*C*), 130.1 (13-*C*), 128.9 (12-*C*), 128.4 (11-*C*), 127.0 (37-*C*), 126.9 (14- and 18-*C*), 123.9 (30-*C*), 121.0 (34-*C*), 118.4 (33-*C*), 118.1 (32-*C*), 113.9 (15- and 17-*C*), 111.4 (35-*C*), 110.0 (31-*C*), 87.8 (42-*C*), 72.9 (5-*C*), 69.4 (2-*C*), 68.4 (4-*C*), 66.7 (23-*C*), 55.7 (28-*C*), 55.0 (20-*C*), 53.7 (3-*C*), 50.4 (39-*C*), 45.9 (44-*C*), 45.2 (24-*C*), 42.8 (26-*C*), 41.4 (22-*C*), 36.8 (40-*C*), 34.4 (6-*C*), 34.1 (45-*C*), 32.7 (7-*C*), 32.5 (10-*C*), 29.6 (9-*C*), 26.0 (8-*C*), 25.7 (29-*C*), 15.5 (19-*C*); *m/z* HRMS (ES^+) MH^+ calculated for $C_{46}H_{61}N_8O_{12}$ 917.4404, observed 917.4397.

3. References.

1. Pullin, R. D. C.; Rathi, A. H.; Melikhova, E. Y.; Winter, C.; Thompson, A. L.; Donohoe, T. J. *Org. Lett.* **2013**, *15*, 5492.

2. a) Shioiri, T.; Sasaki, S.; Hamada, Y. *ARKIVOC* **2003**, 103. b) Zhu J. and Ma D. *Angew., Angew. Chem. Int. Ed.* ***2003****, 42, 5348.*

3. Eldo, J.; Cardia, J. P.; O'Day, E. M.; Xia, J.; Tsuruta, H.; Kantrowitz, E. R. *J. Med. Chem.* **2006**, *49*, 5932.

4. Sureshbabu, V. V.; Naik, S. A.; Nagendra, G. *Synth. Comm.* **2009**, *39*, 395.

5. Hannick, S. M.; Kishi, Y. J. *Org. Chem*. **1983**, *48*, 3833.

6. Zhang, X.; Jacob, M. R.; Rao, R. R.; Wang, Y. H.; Agarwall, A. K.; Newman, D. J.; Khan, I. A.; Clark, A. M.; Li, X. C. *Research and Reports in Medicinal Chemistry* **2012**, *2*, 7.

4. NMR and HPLC data of novel compounds.

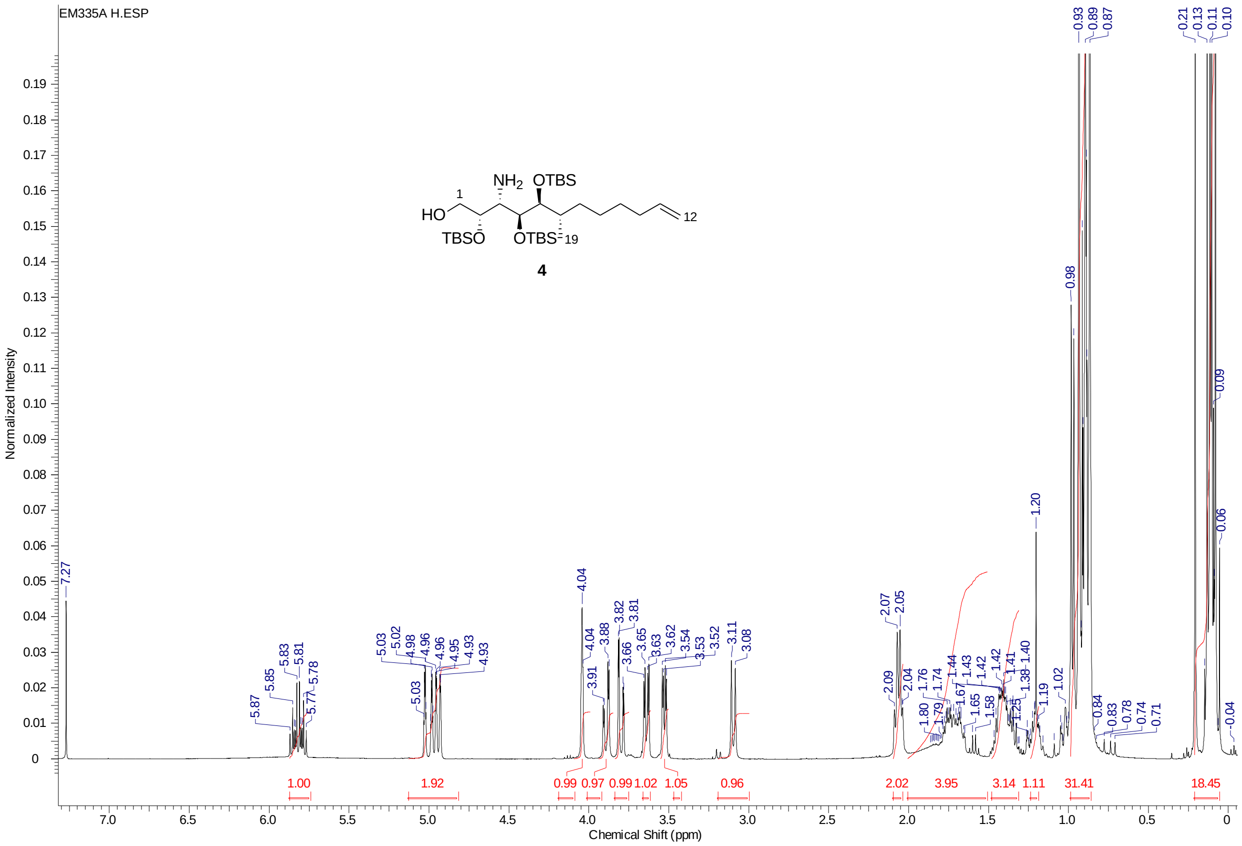

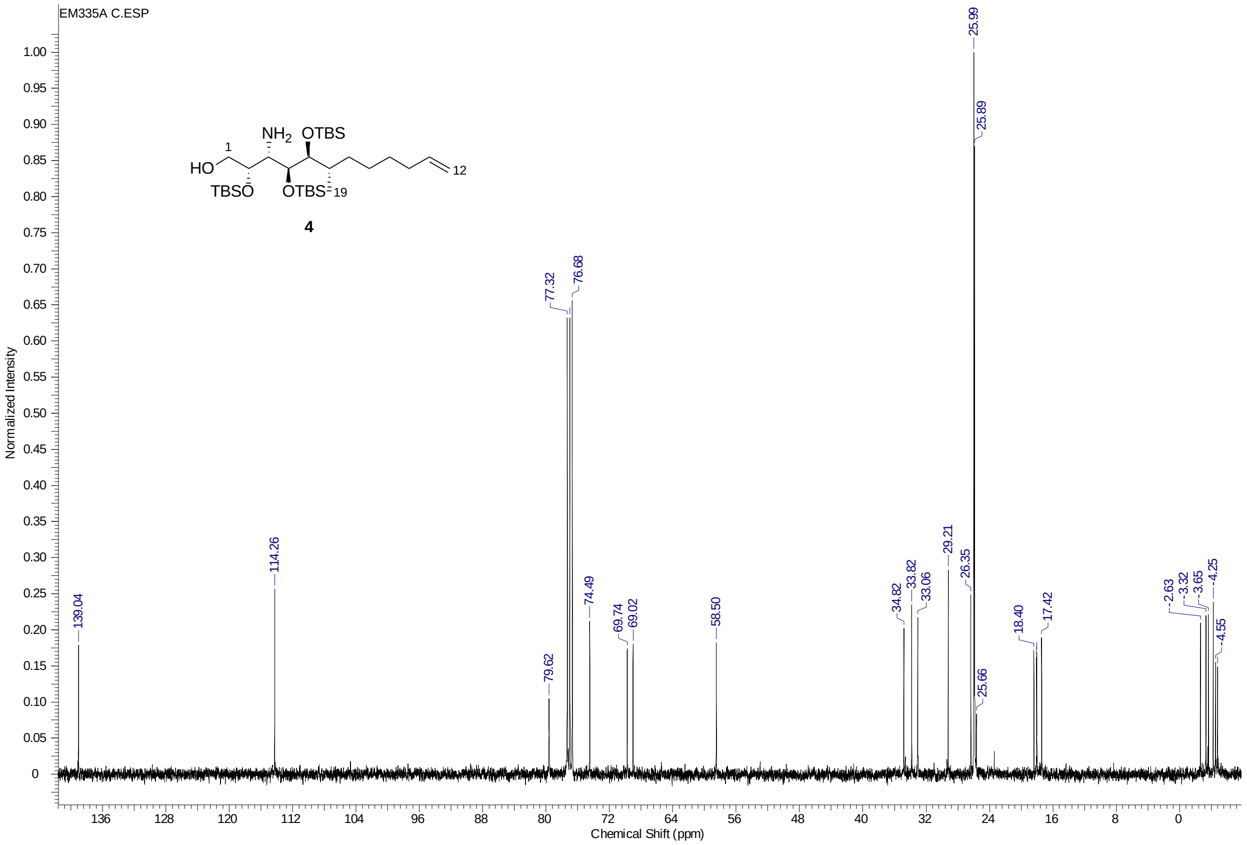

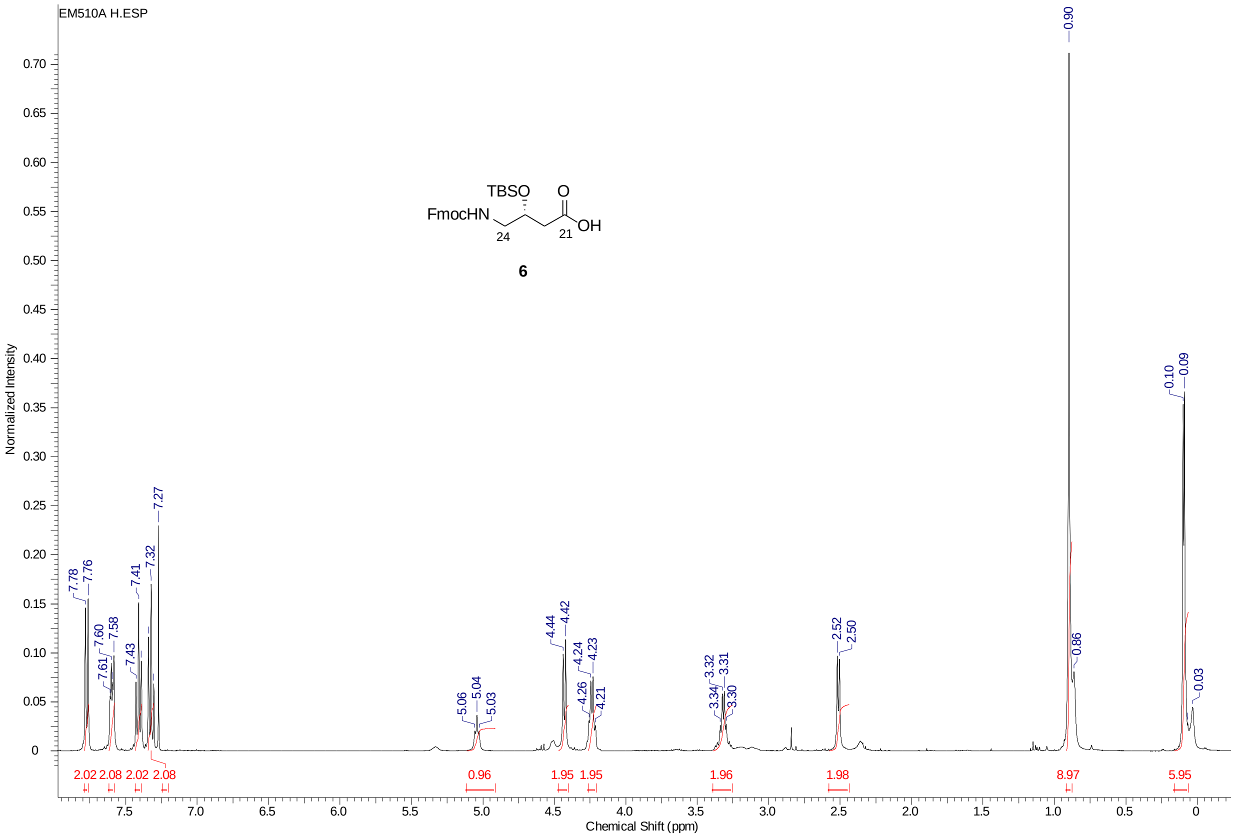
EM510A H.ESP
TBSO
O
FmocHN
OH
24
21
6
Normalized Intensity
Chemical Shift (ppm)
0.90
0.10
0.09
7.78
7.76
7.60
7.58
7.61
7.43
7.41
7.32
7.27
5.06
5.04
5.03
4.44
4.42
4.26
4.24
4.23
4.21
3.34
3.32
3.31
3.30
2.52
2.50
0.86
0.03
2.02
2.08
2.02
2.08
0.96
1.95
1.95
1.96
1.98
8.97
5.95

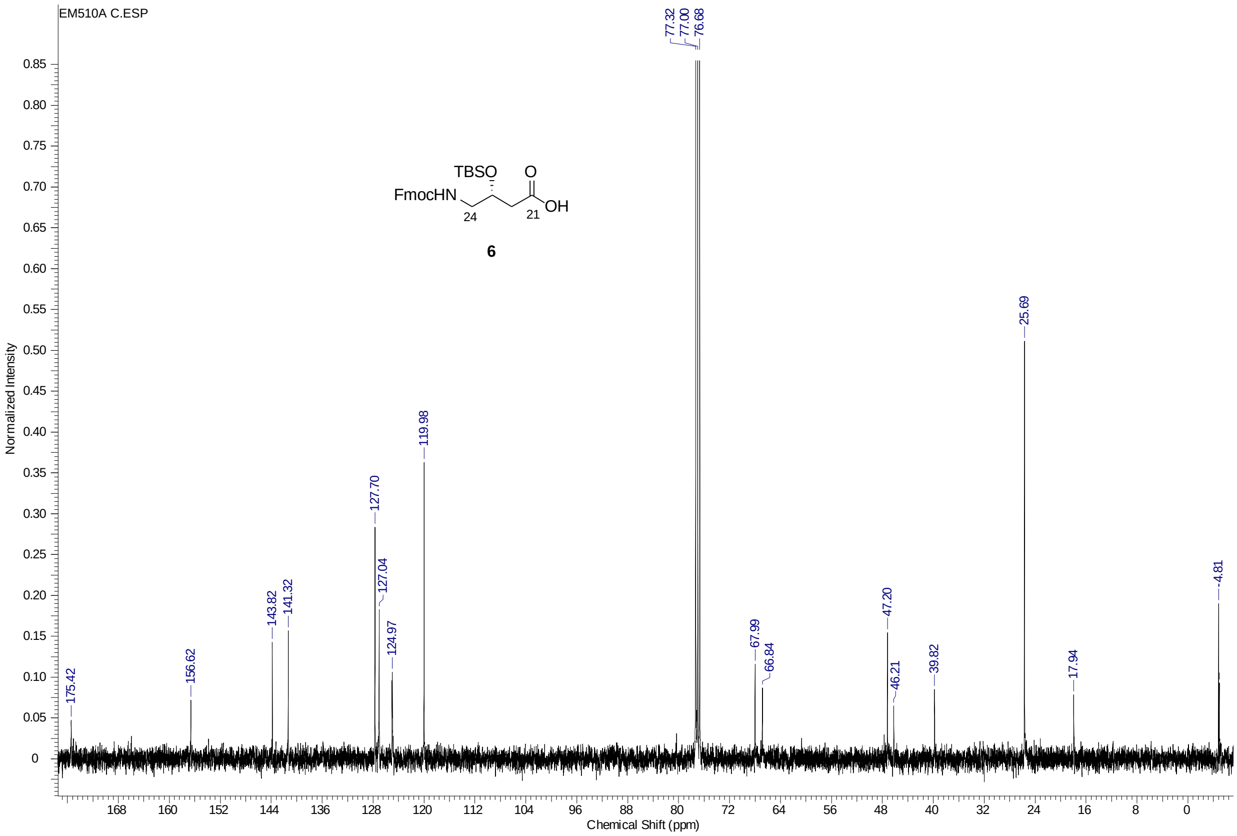
EM510A C.ESP
TBSO
O
FmocHN
OH
24
21
6
Normalized Intensity
Chemical Shift (ppm)
77.32
77.00
76.68
175.42
156.62
143.82
141.32
127.70
127.04
124.97
119.98
67.99
66.84
47.20
46.21
39.82
25.69
17.94
-4.81

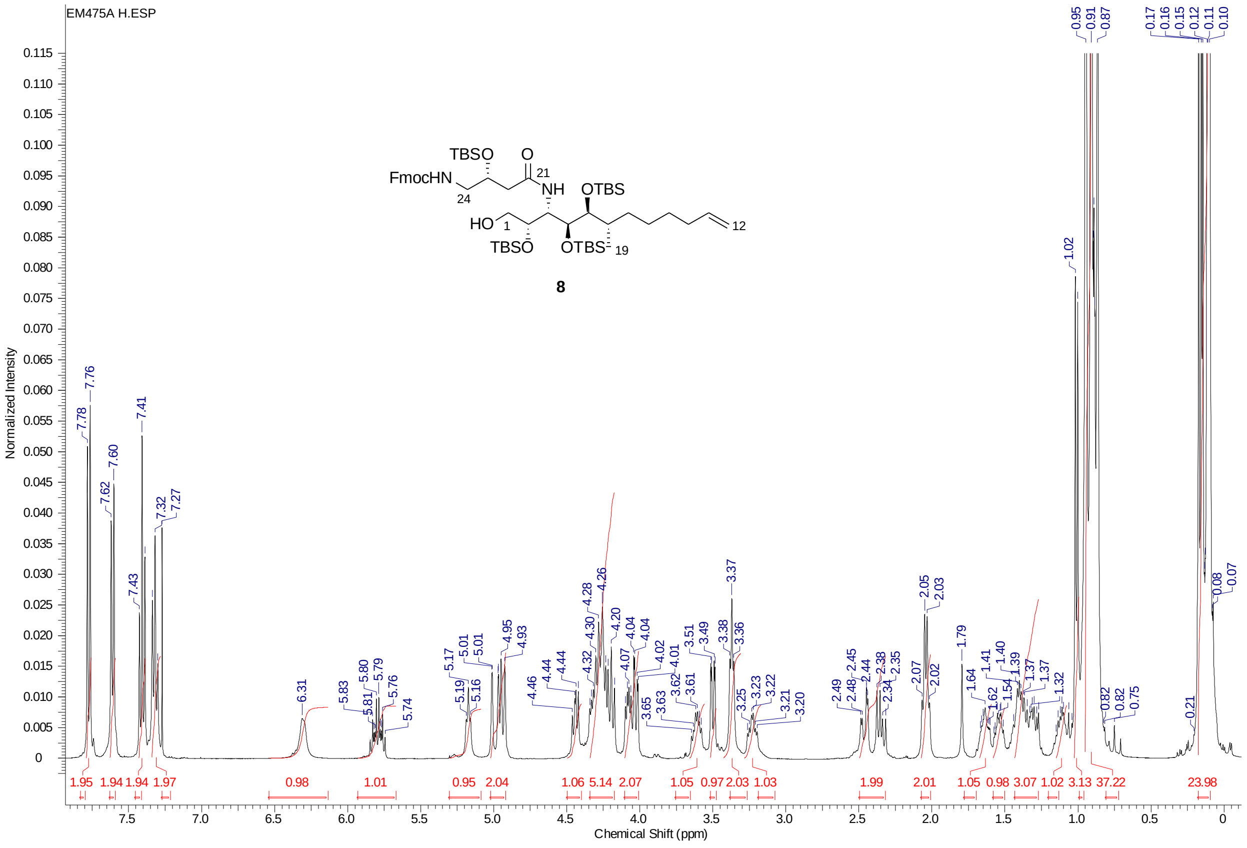
EM475A H.ESP
TBSO
O
21
FmocHN
24
NH
OTBS
HO
1
12
TBSO
OTBS
19
8
Normalized Intensity
Chemical Shift (ppm)

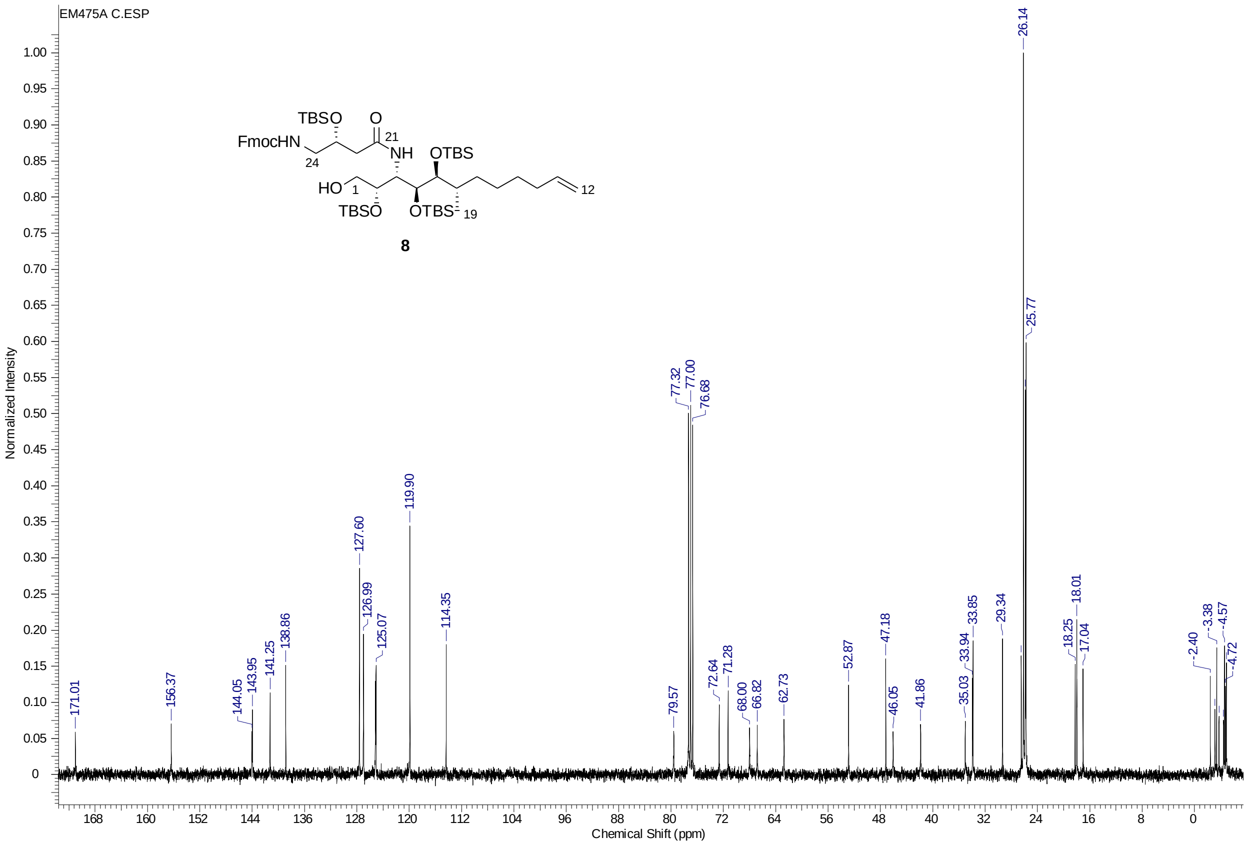
EM475A C.ESP
TBSO
O
21
FmocHN
24
NH
OTBS
HO
1
12
TBSO
OTBS
19
8
Normalized Intensity
Chemical Shift (ppm)

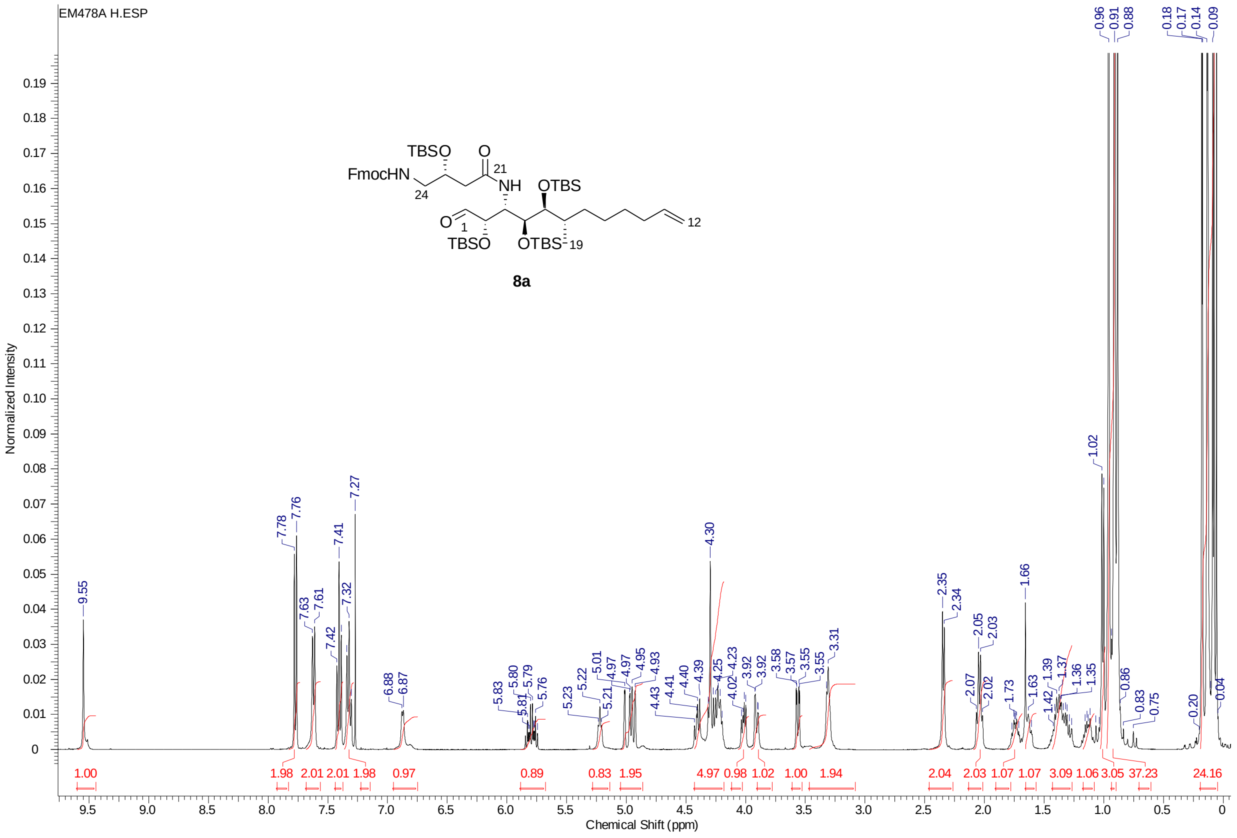
EM478A H.ESP
8a
Normalized Intensity
Chemical Shift (ppm)

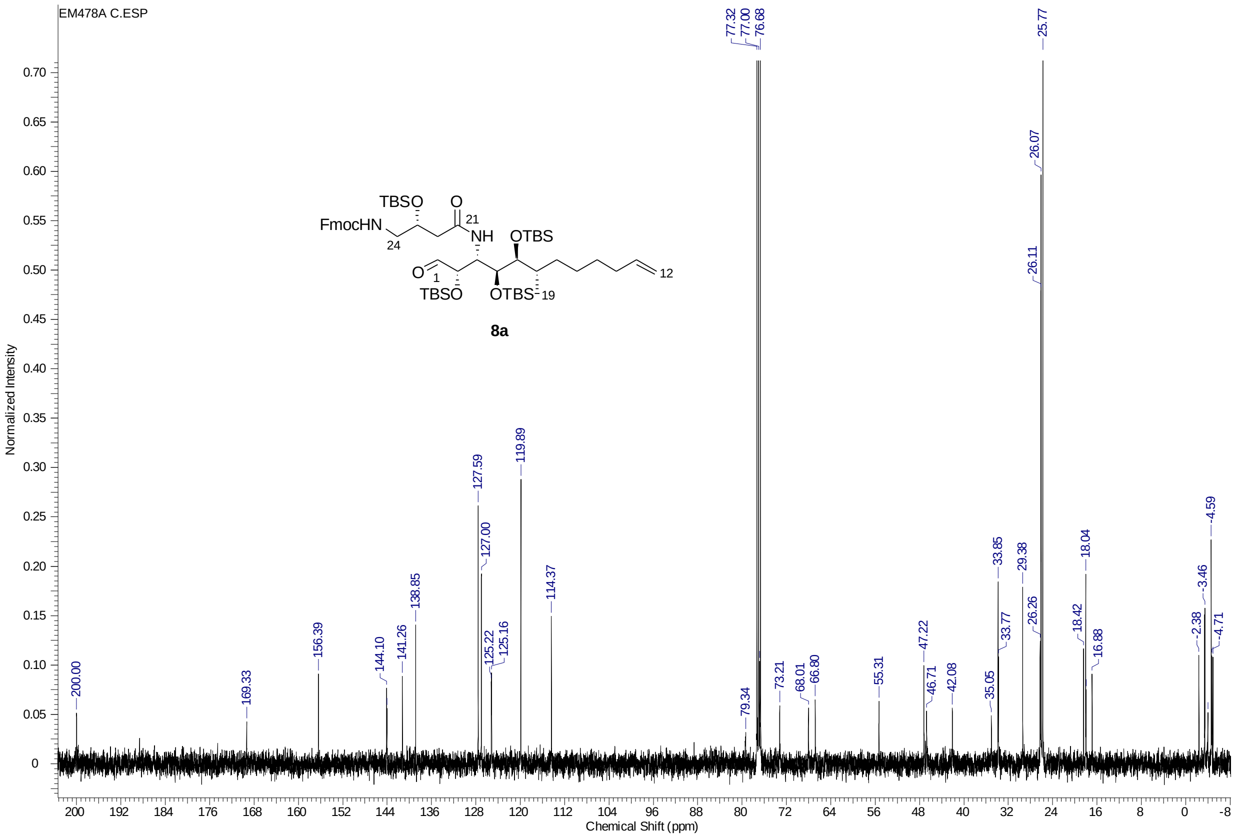
EM478A C.ESP
8a
Normalized Intensity
Chemical Shift (ppm)

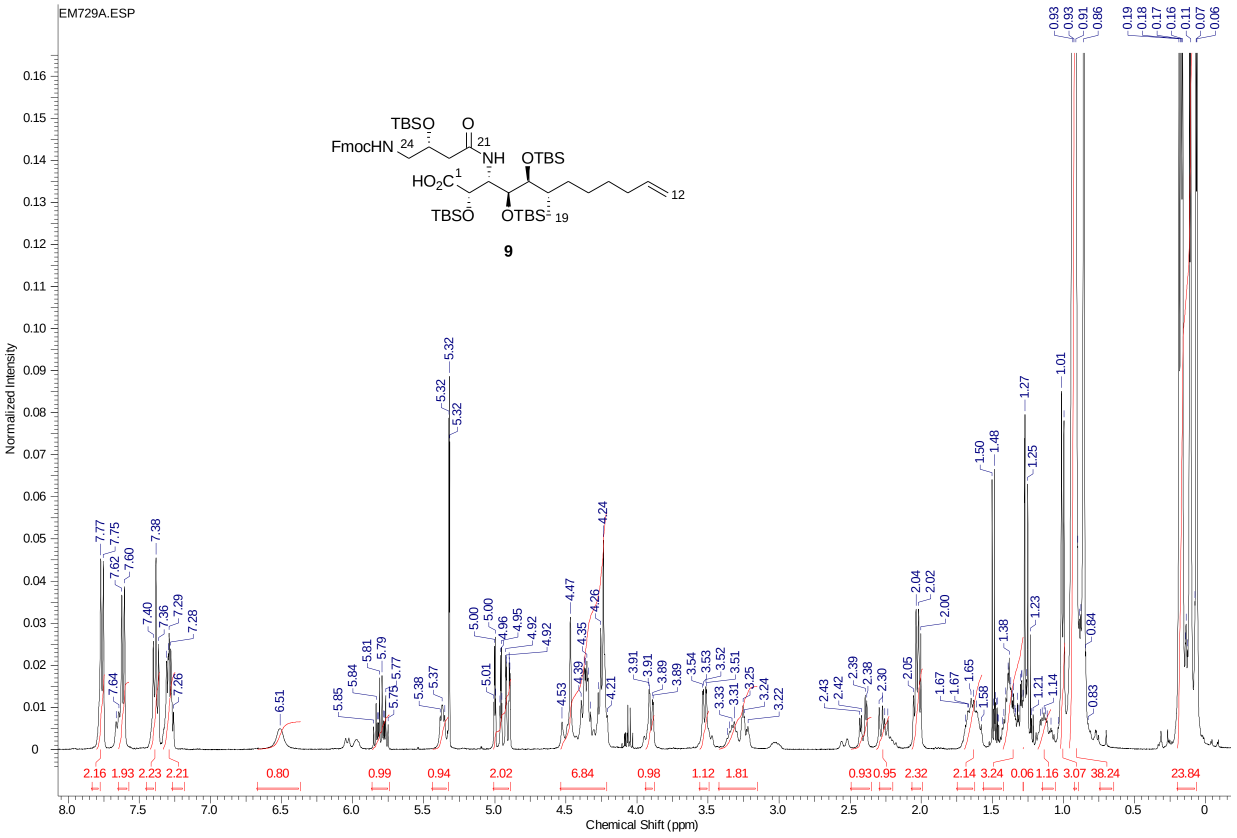
EM729A.ESP
Normalized Intensity
Chemical Shift (ppm)
TBSO
O
FmocHN
24
21
NH
OTBS
HO2C
1
12
TBSO
OTBS
19
9

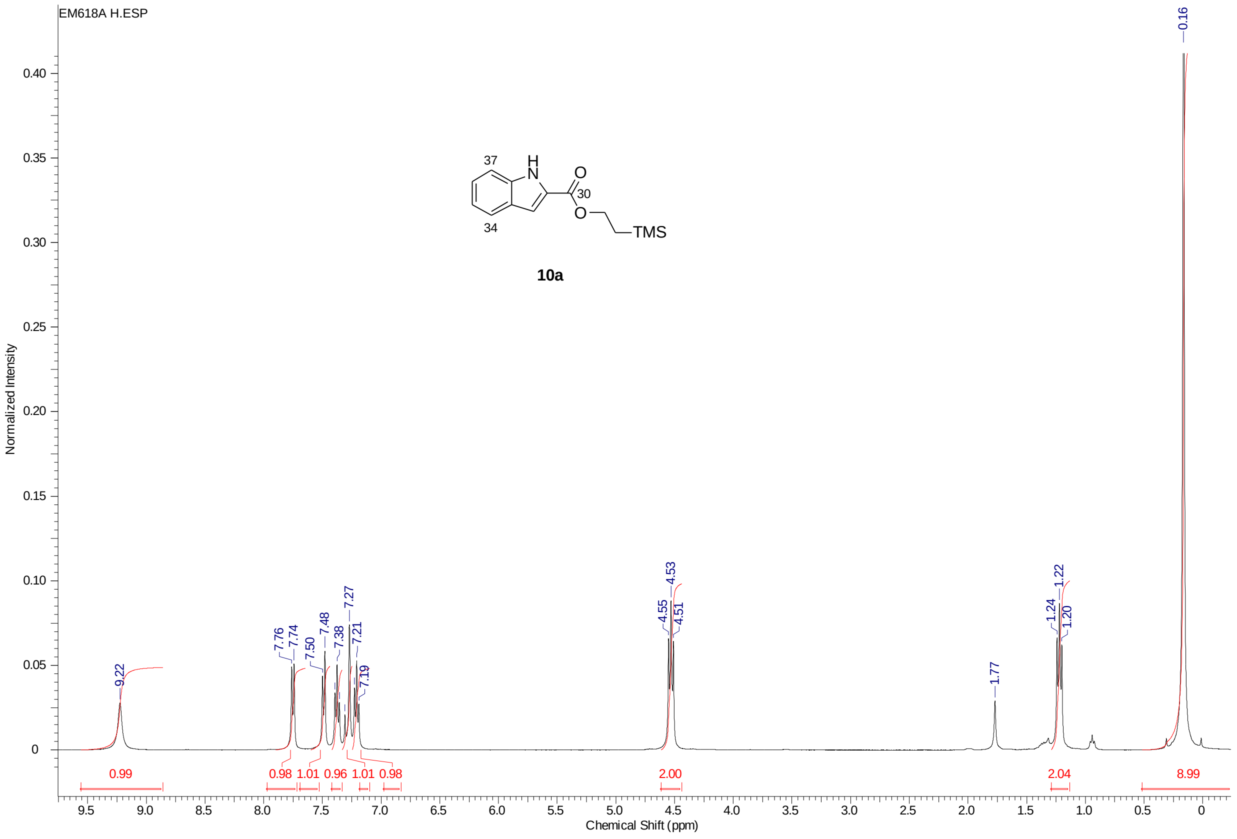
EM618A H.ESP
Normalized Intensity
Chemical Shift (ppm)
37
H
N
O
30
O
TMS
34
10a

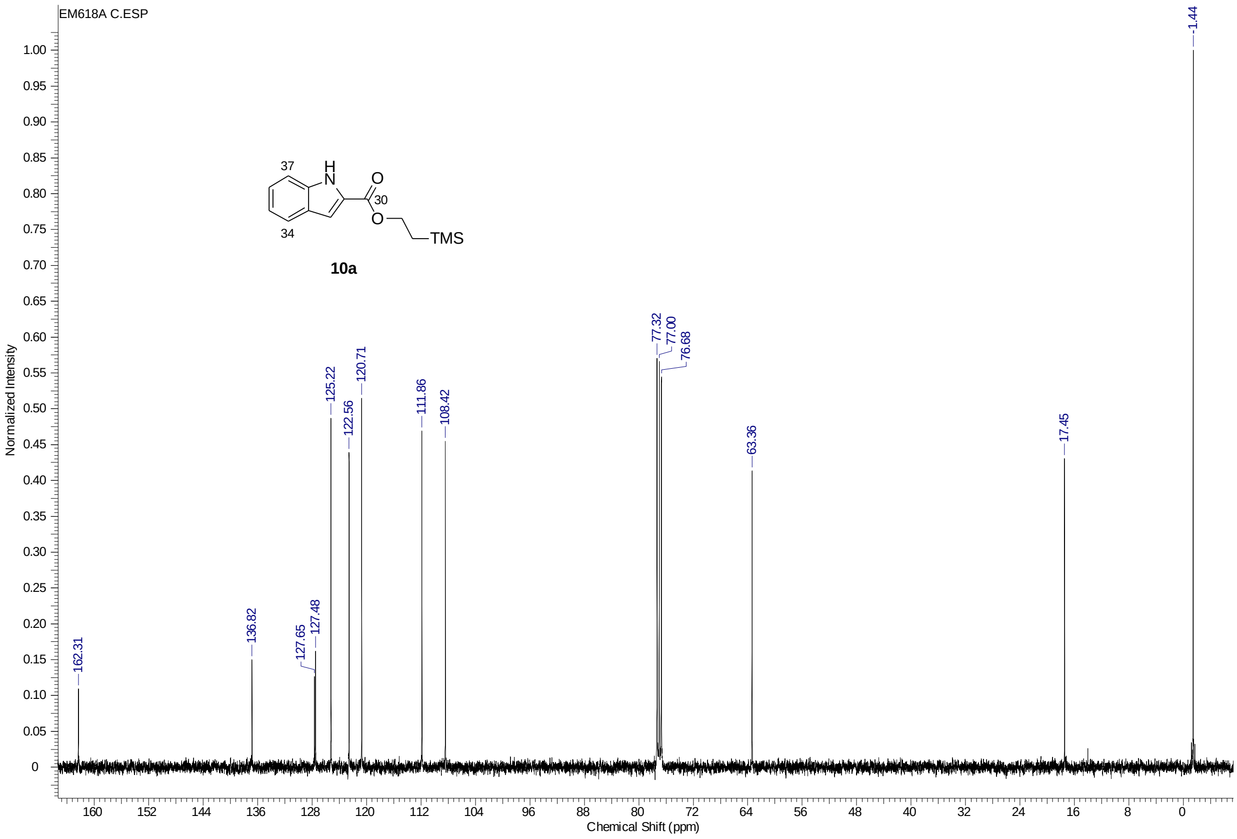
EM618A C.ESP
10a
Normalized Intensity
Chemical Shift (ppm)

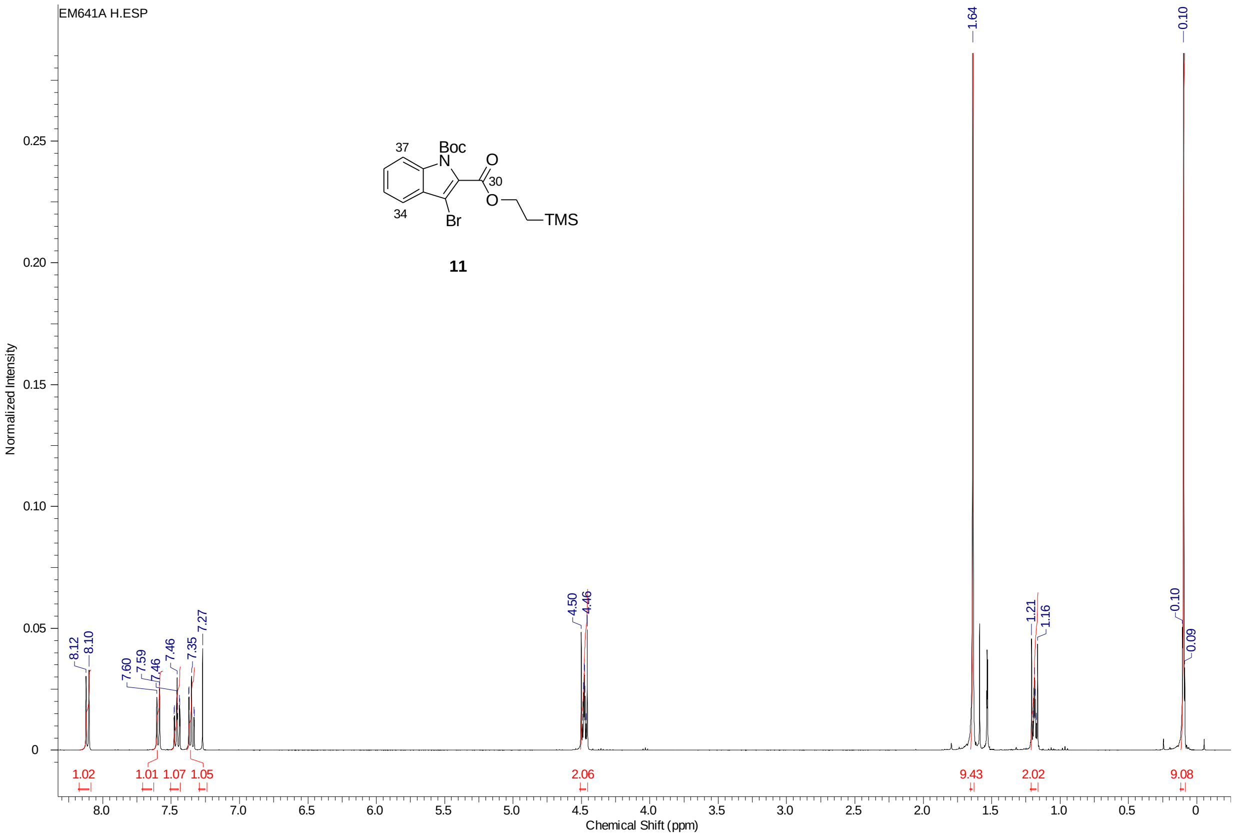
EM641A H.ESP
11
Normalized Intensity
Chemical Shift (ppm)

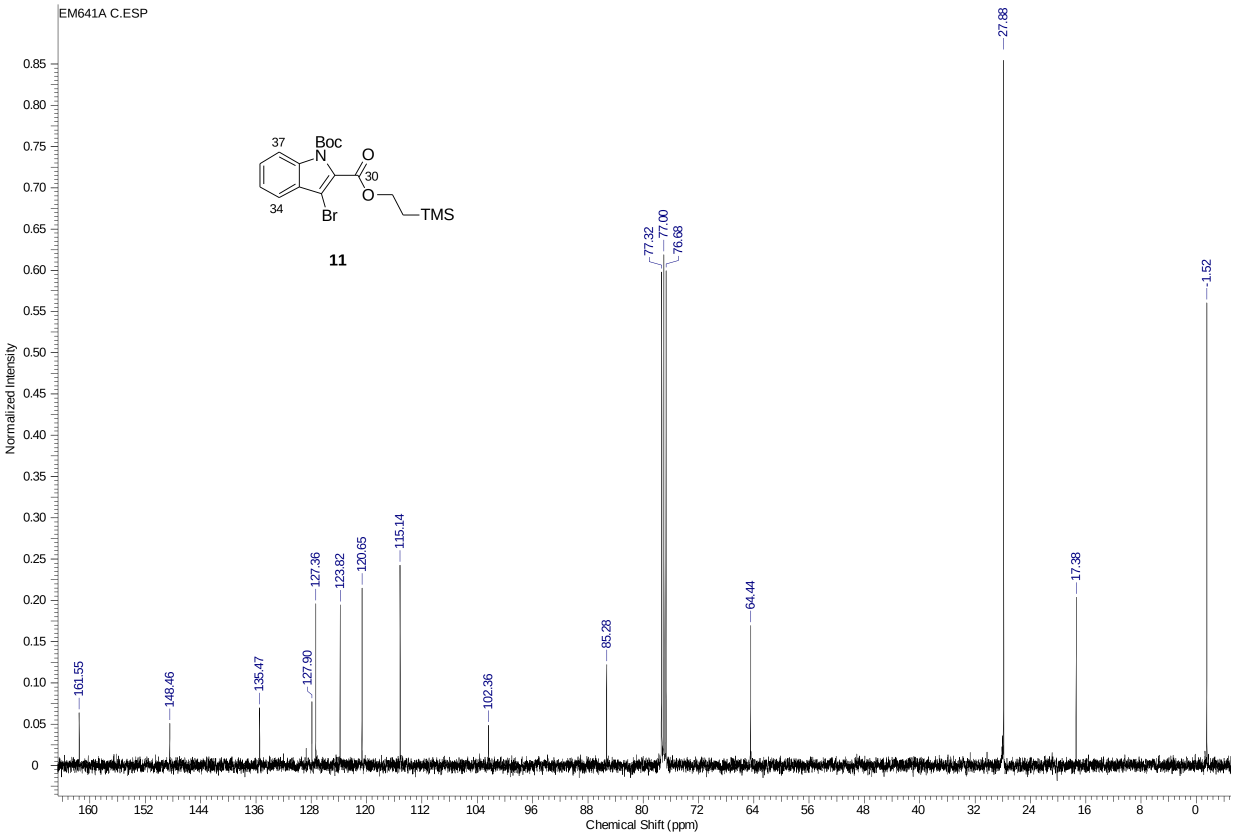
EM641A C.ESP
Normalized Intensity
Chemical Shift (ppm)
Boc
N
O
30
O
TMS
Br
37
34
11
161.55
148.46
135.47
127.90
127.36
123.82
120.65
115.14
102.36
85.28
77.32
77.00
76.68
64.44
27.88
17.38
-1.52

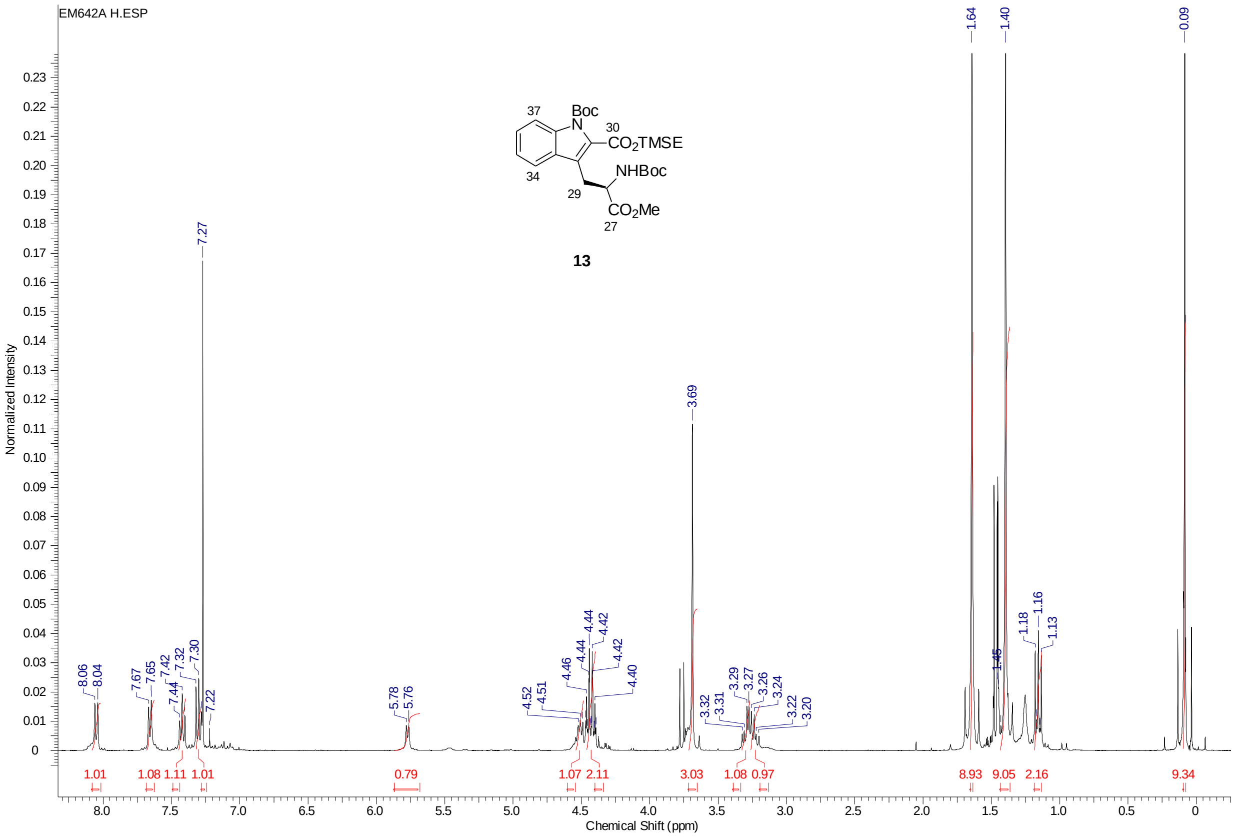
EM642A H.ESP
Normalized Intensity
Chemical Shift (ppm)
Boc
N
30
CO2TMSE
NHBoc
CO2Me
37
34
29
27
13
8.06
8.04
7.67
7.65
7.44
7.42
7.32
7.30
7.27
7.22
5.78
5.76
4.52
4.51
4.46
4.44
4.44
4.42
4.42
4.40
3.69
3.32
3.31
3.29
3.27
3.26
3.24
3.22
3.20
1.64
1.46
1.40
1.18
1.16
1.13
0.09
1.01
1.08
1.11
1.01
0.79
1.07
2.11
3.03
1.08
0.97
8.93
9.05
2.16
9.34

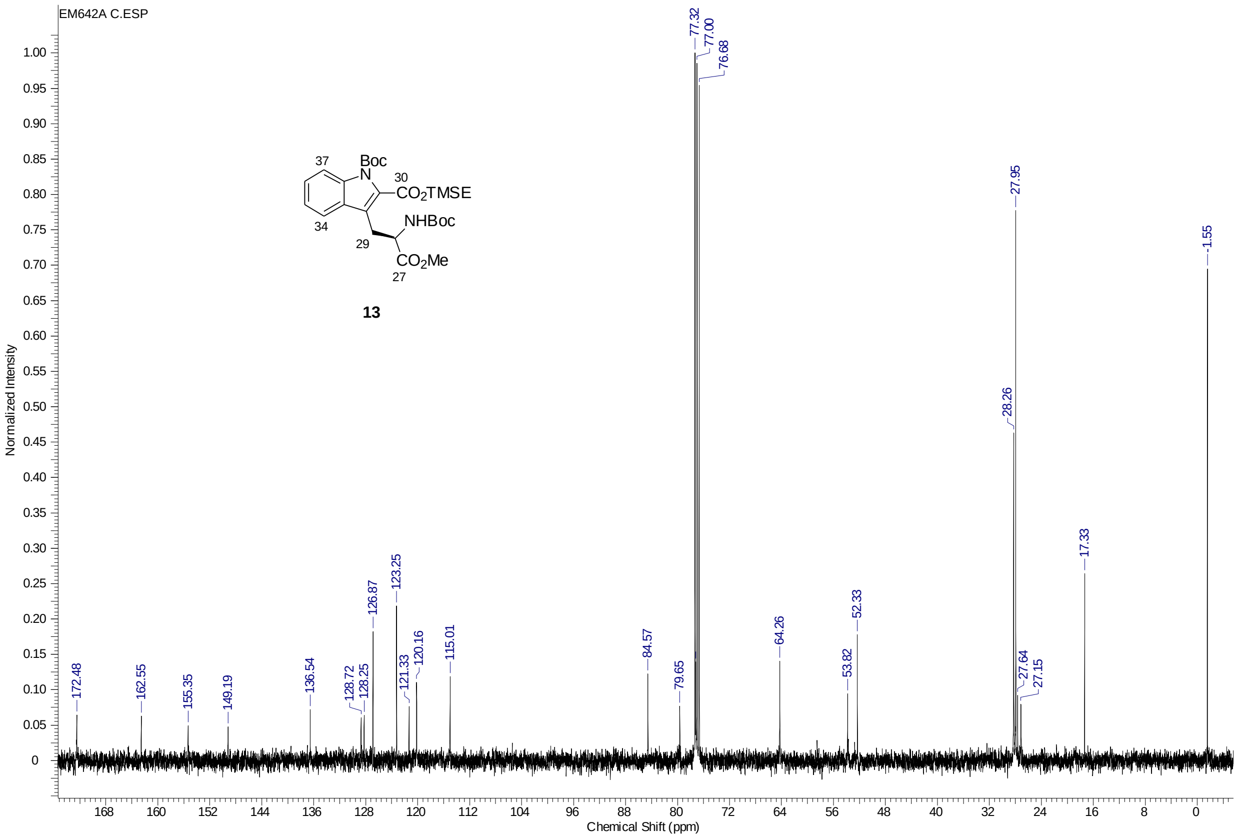
EM642A C.ESP
Normalized Intensity
Chemical Shift (ppm)
Boc
37
30
CO2TMSE
NHBoc
34
29
CO2Me
27
13
77.32
77.00
76.68
27.95
1.55
28.26
17.33
123.25
126.87
52.33
64.26
84.57
115.01
120.16
121.33
53.82
79.65
27.64
27.15
172.48
162.55
155.35
149.19
136.54
128.72
128.25

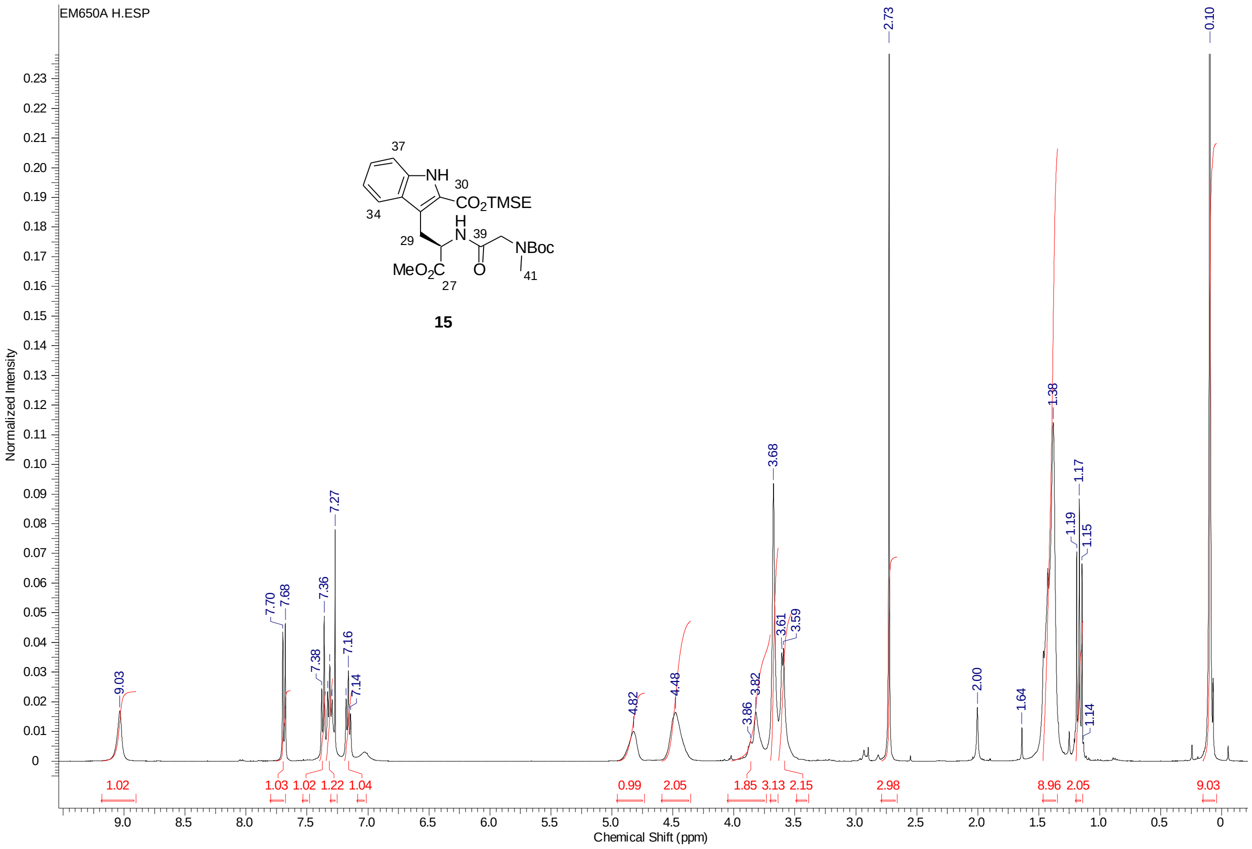
EM650A H.ESP
Normalized Intensity
Chemical Shift (ppm)
37
NH
30
CO2TMSE
34
H
29
N
39
NBoc
MeO2C
O
27
41
15
2.73
0.10
1.38
1.17
3.68
7.27
1.19
1.15
7.70
7.68
7.36
3.61
3.59
7.38
7.16
9.03
7.14
3.82
4.48
4.82
3.86
2.00
1.64
1.14
1.02
1.03
1.02
1.22
1.04
0.99
2.05
1.85
3.13
2.15
2.98
8.96
2.05
9.03

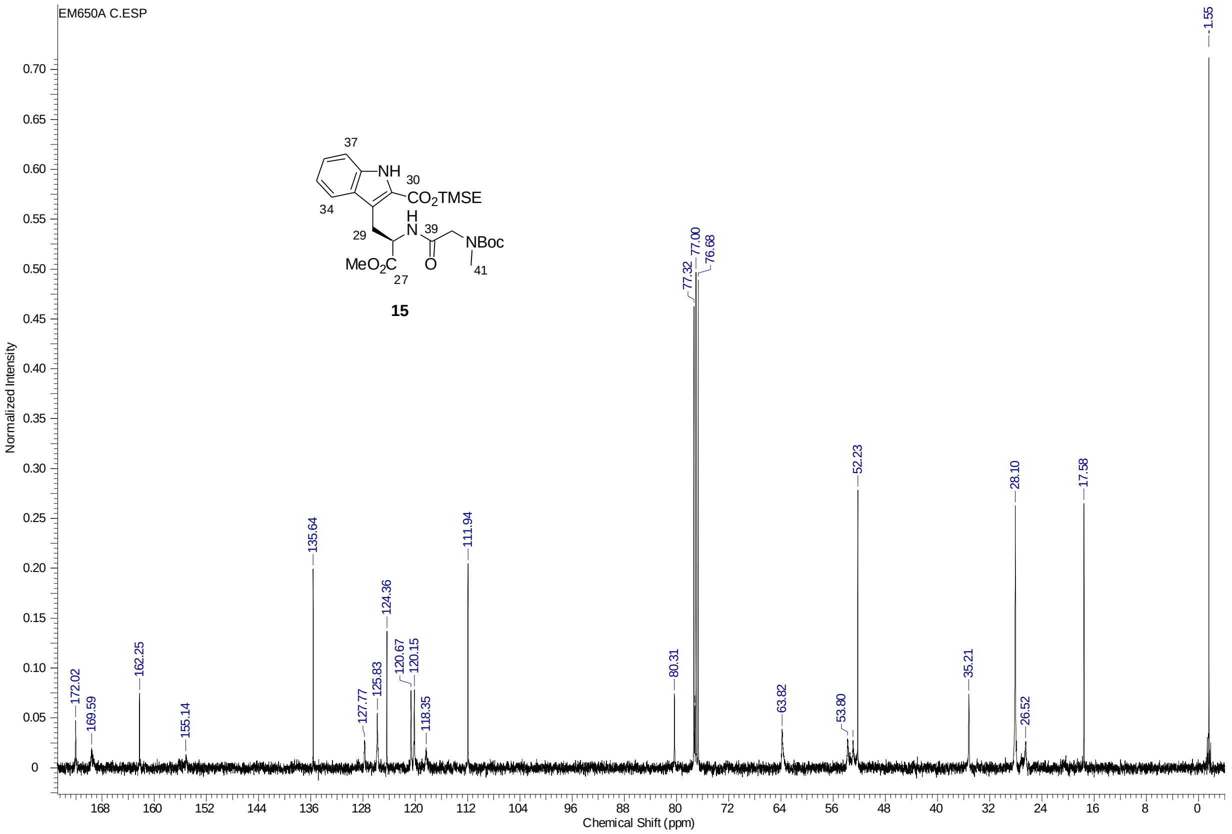
EM650A C.ESP
Normalized Intensity
Chemical Shift (ppm)
NH
30
37
CO2TMSE
34
H
N
39
29
NBoc
MeO2C
O
27
41
15

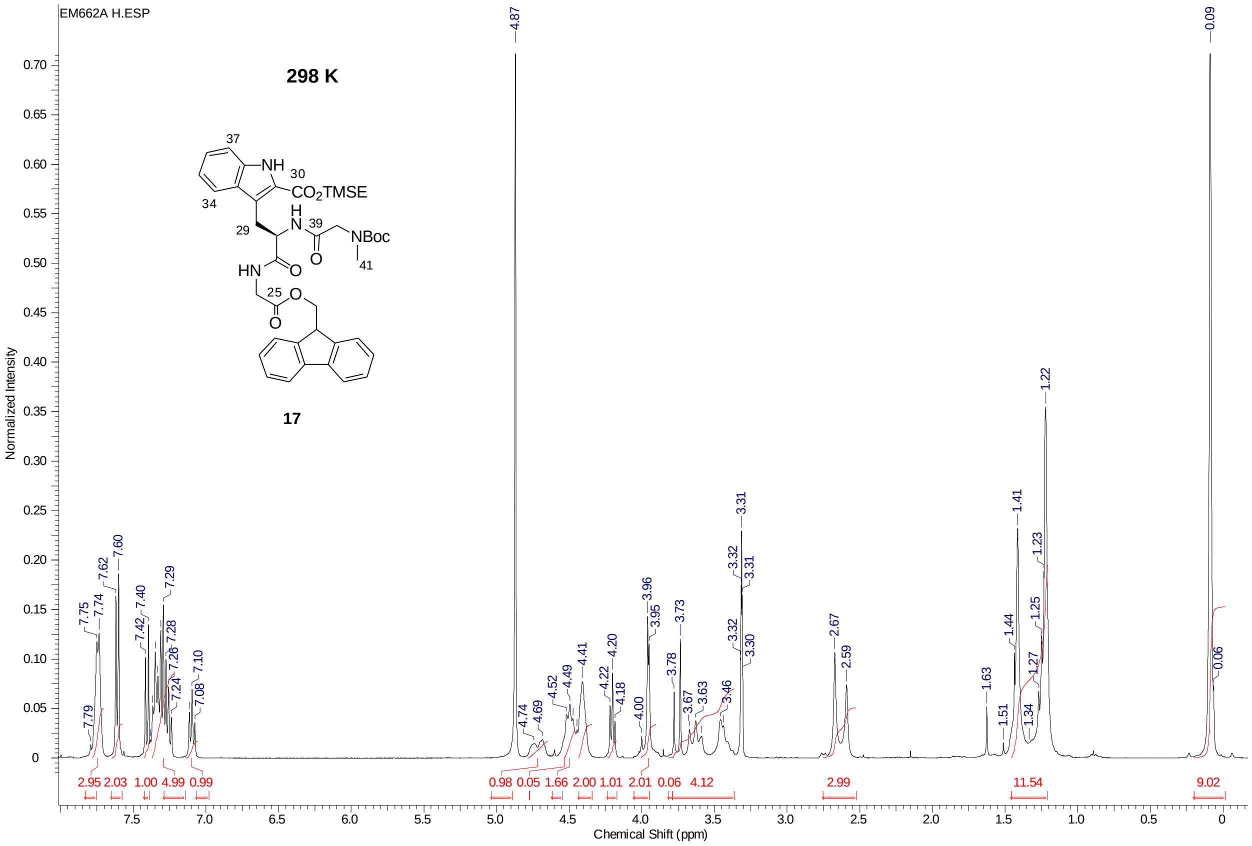
EM662A H.ESP
298 K
Normalized Intensity
Chemical Shift (ppm)
37
NH
30
CO2TMSE
34
H
N
39
29
NBoc
HN
O
41
25
O
O
17

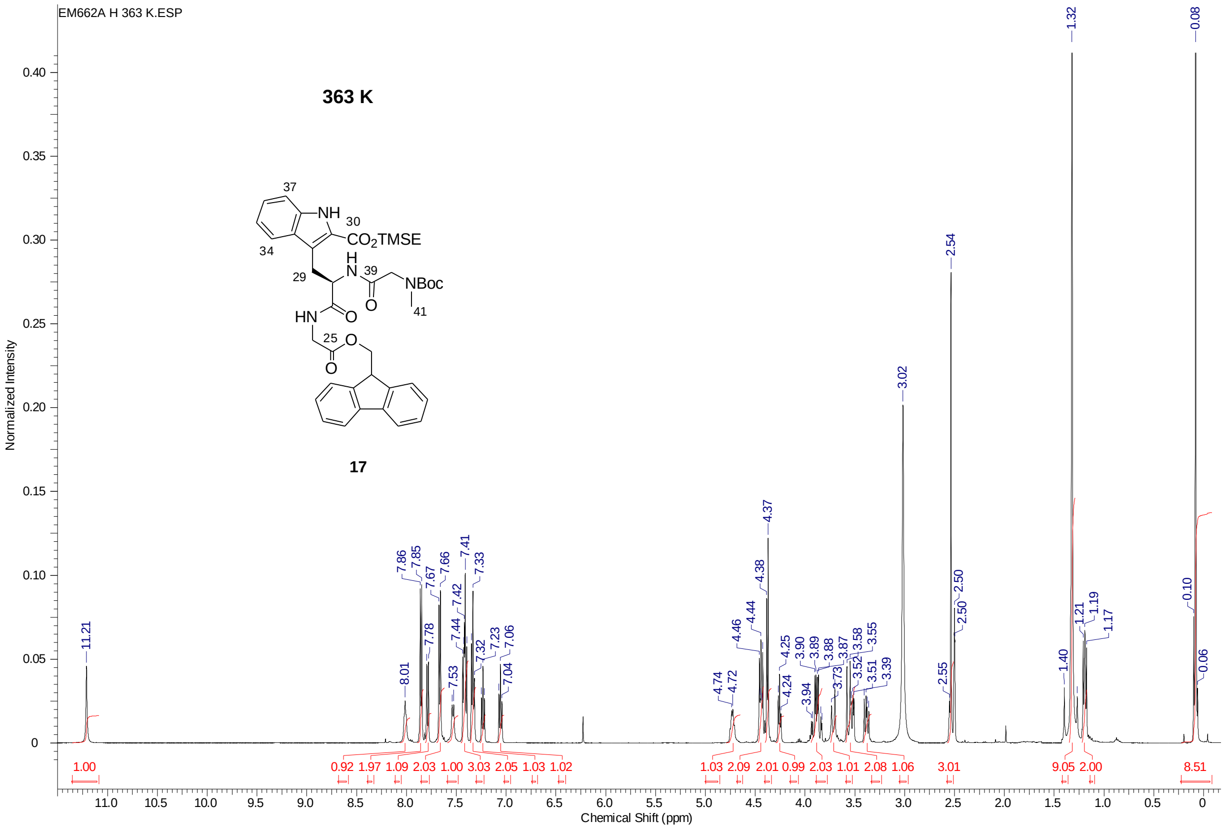
EM662A H 363 K.ESP
363 K
17
Normalized Intensity
Chemical Shift (ppm)
11.21
8.01
7.86
7.85
7.78
7.67
7.66
7.53
7.44
7.42
7.41
7.33
7.32
7.23
7.06
7.04
4.74
4.72
4.46
4.44
4.38
4.37
4.25
4.24
3.94
3.90
3.89
3.88
3.87
3.73
3.58
3.55
3.52
3.51
3.39
3.02
2.55
2.54
2.50
2.50
1.40
1.32
1.21
1.19
1.17
0.10
0.08
0.06
1.00
0.92
1.97
1.09
2.03
1.00
3.03
2.05
1.03
1.02
1.03
2.09
2.01
0.99
2.03
1.01
2.08
1.06
3.01
9.05
2.00
8.51

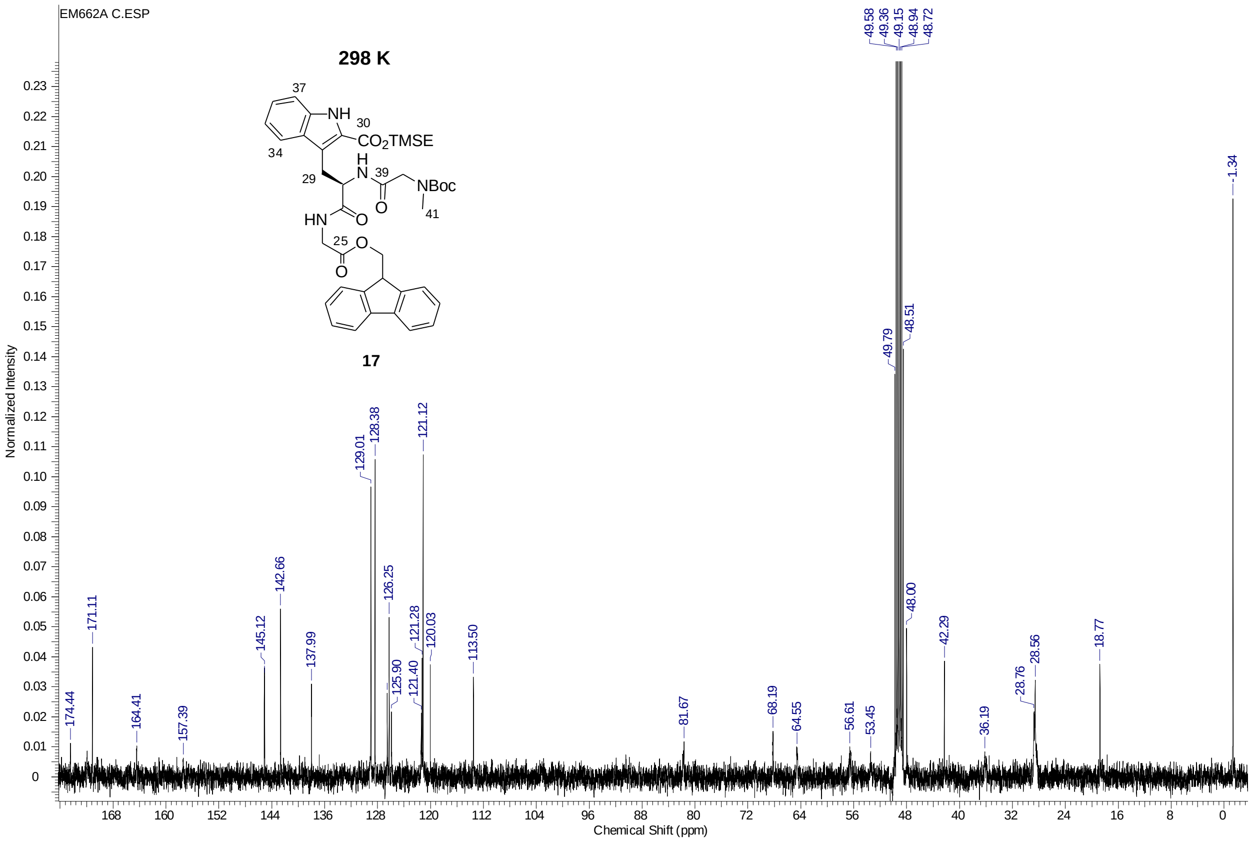
EM662A C.ESP
298 K
17
Normalized Intensity
Chemical Shift (ppm)
174.44
171.11
164.41
157.39
145.12
142.66
137.99
129.01
128.38
126.25
125.90
121.40
121.28
121.12
120.03
113.50
81.67
68.19
64.55
56.61
53.45
49.79
49.58
49.36
49.15
48.94
48.72
48.51
48.00
42.29
36.19
28.76
28.56
18.77
-1.34

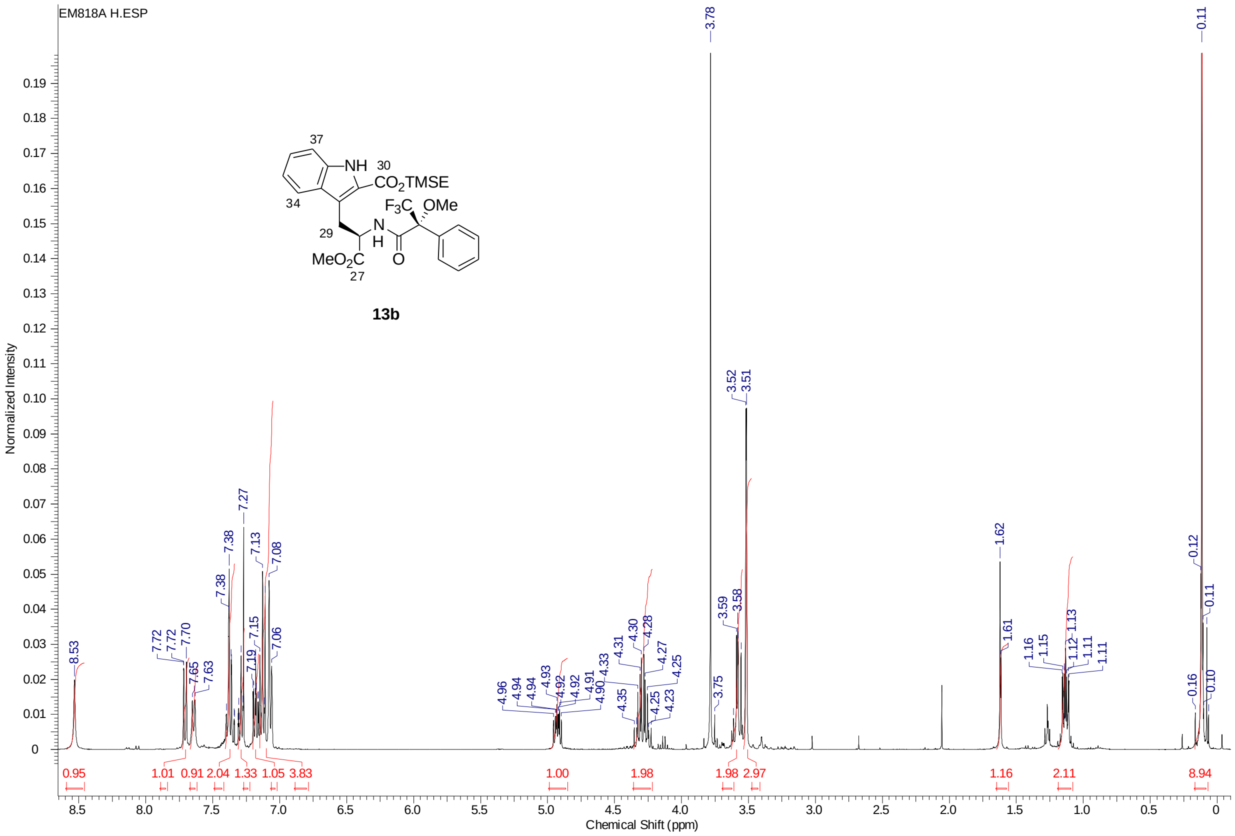
EM818A H.ESP
13b
37
NH 30
CO2TMSE
34
F3C OMe
29
N
H
MeO2C
O
27
Normalized Intensity
Chemical Shift (ppm)
0.95 1.01 0.91 2.04 1.33 1.05 3.83 1.00 1.98 1.98 2.97 1.16 2.11 8.94

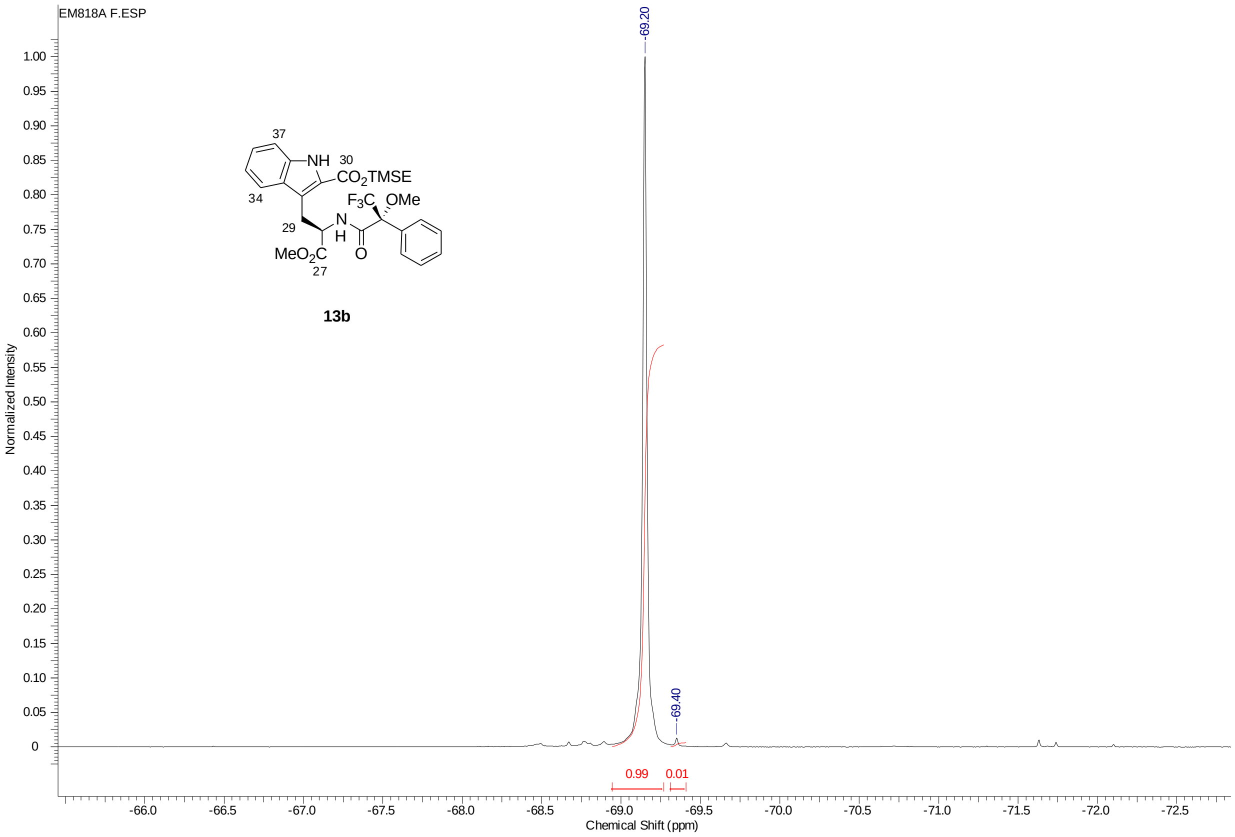
EM818A F.ESP
13b
-69.20
-69.40
0.99 0.01
Normalized Intensity
Chemical Shift (ppm)

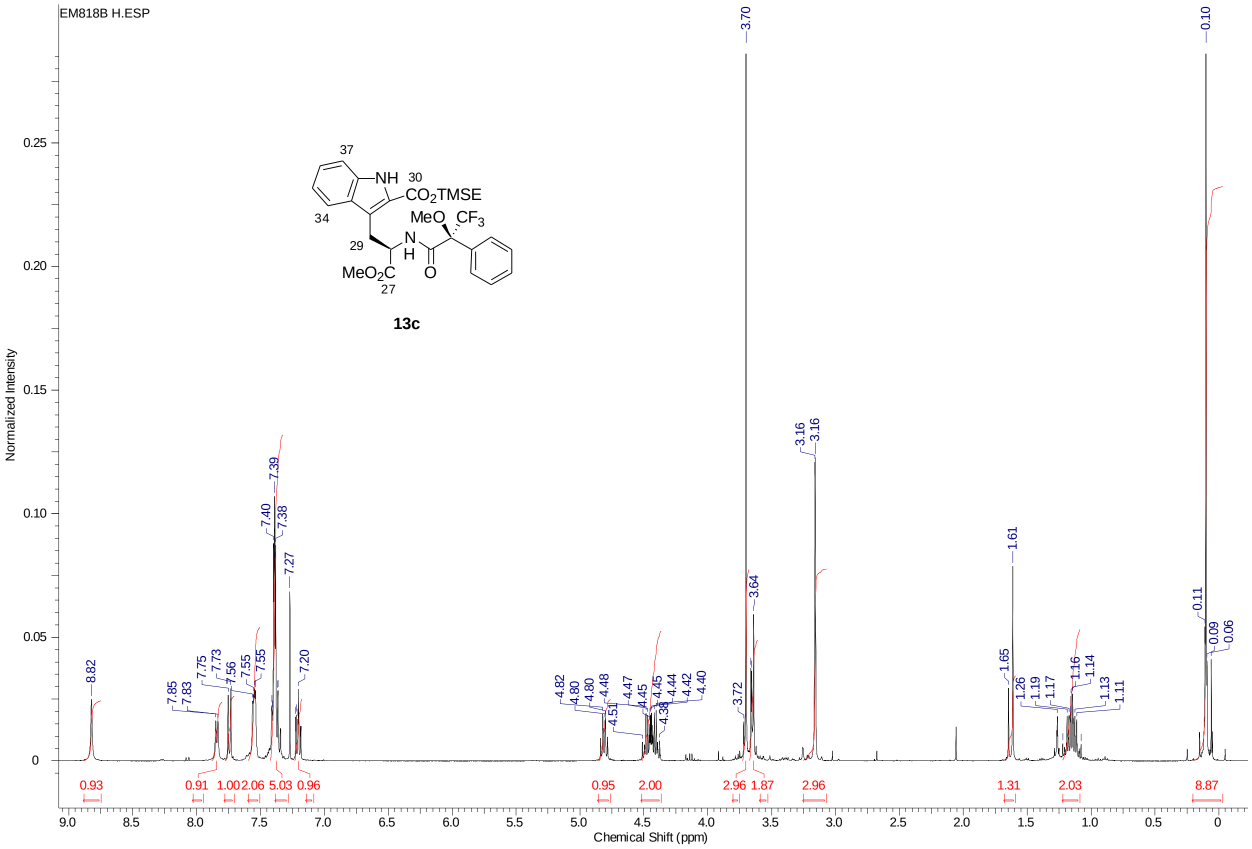
EM818B H.ESP
13c
Chemical Shift (ppm)
Normalized Intensity

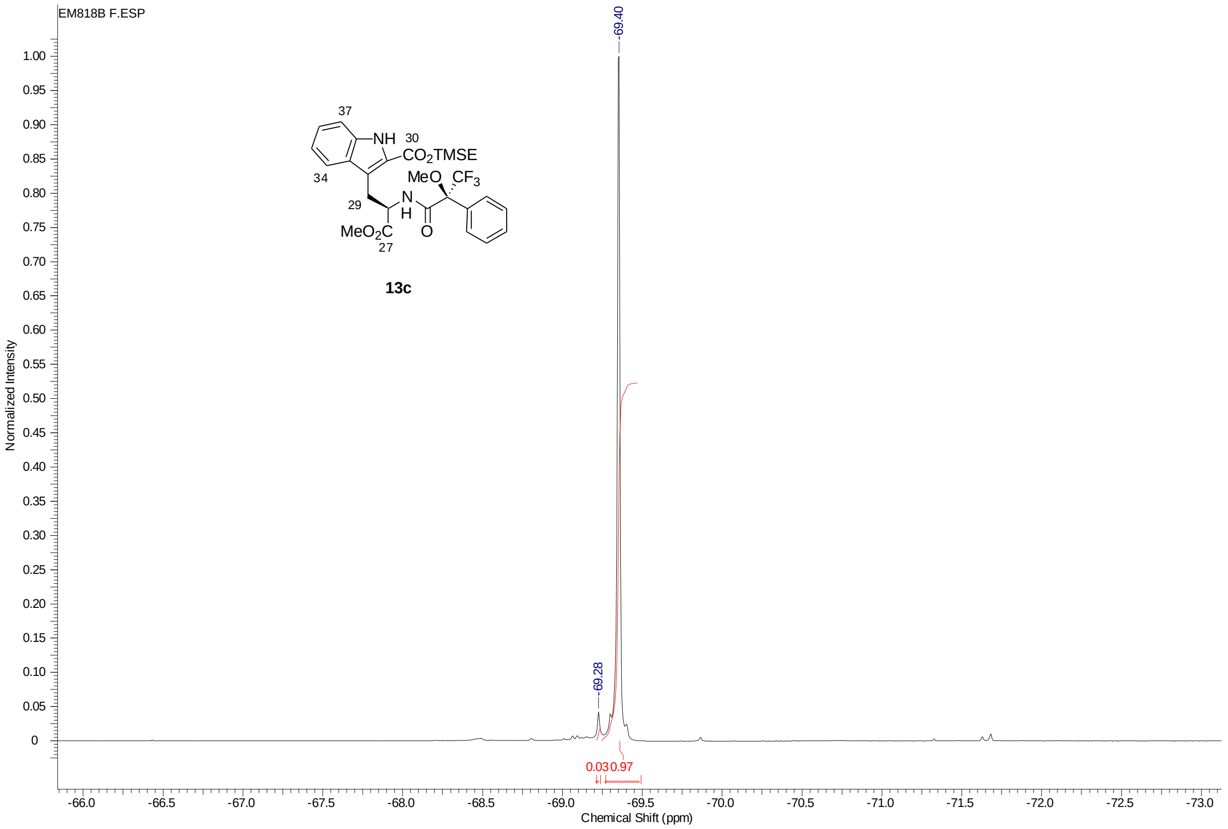
EM818B F.ESP
13c
Chemical Shift (ppm)
Normalized Intensity

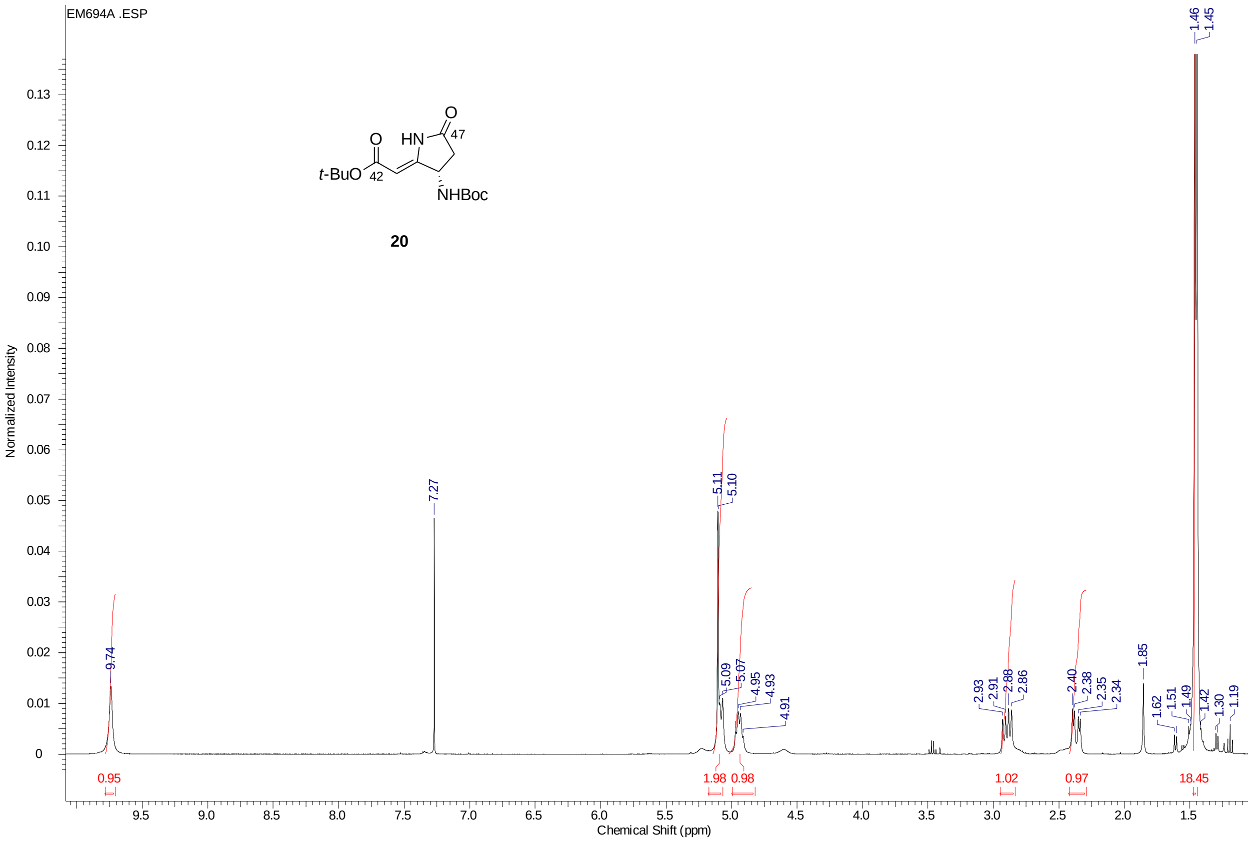
EM694A .ESP
Normalized Intensity
Chemical Shift (ppm)
t-BuO
42
HN
47
NHBoc
20
9.74
7.27
5.11
5.10
5.09
5.07
4.95
4.93
4.91
2.93
2.91
2.88
2.86
2.40
2.38
2.35
2.34
1.85
1.62
1.51
1.49
1.46
1.45
1.42
1.30
1.19
0.95
1.98
0.98
1.02
0.97
18.45

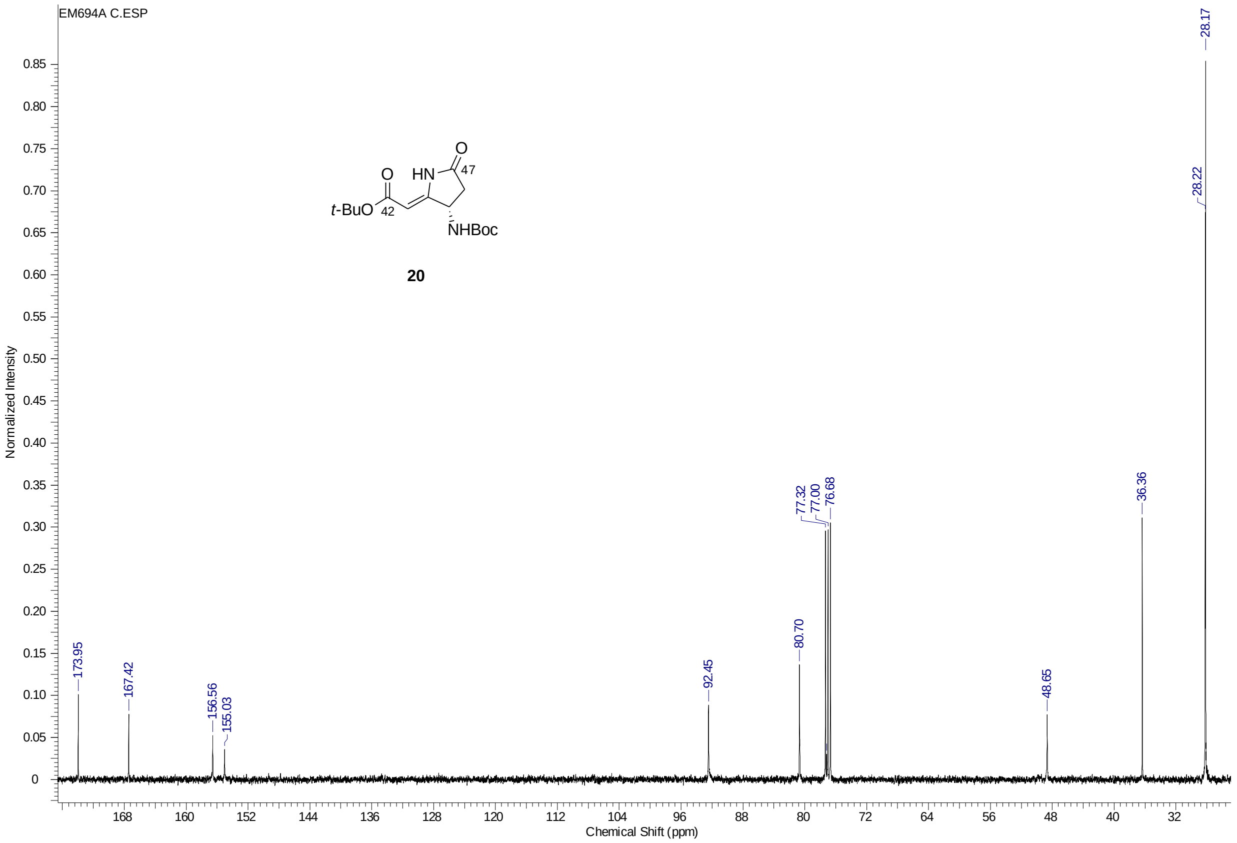
EM694A C.ESP
Normalized Intensity
Chemical Shift (ppm)
t-BuO
42
HN
47
NHBoc
20
173.95
167.42
156.56
155.03
92.45
80.70
77.32
77.00
76.68
48.65
36.36
28.22
28.17

HPLC traces of a racemic sample of 20 (left) and a sample of 20 obtained from the Blaise reaction on enantiopure starting material 19 (right)

Operator:tjdgroup Timebase:TJGSUPERCOMP_1 Sequence:Project AA — Page 1-1 — 14/9/2011 11:26 AM

1 CW107

Sample Name:	CW107	Injection Volume:	20.0
Vial Number:	0	Channel:	UV_VIS_1
Sample Type:	unknown	Wavelength:	254
Control Program:	10pc 1200mL	Bandwidth:	n.a.
Quantif. Method:	Trial	Dilution Factor:	1.0000
Recording Time:	14/9/2011 9:44	Sample Weight:	1.0000
Run Time (min):	30.00	Sample Amount:	1.0000

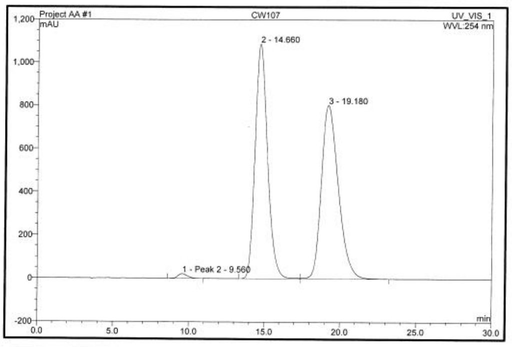

No.	Ret.Time min	Peak Name	Height mAU	Area mAU*min	Rel.Area %	Amount	Type
1	9.56	Peak 2	21.335	15.506	0.74	n.a.	BMB
2	14.66	n.a.	1087.476	1041.382	49.49	n.a.	BM
3	19.18	n.a.	803.225	1047.274	49.77	n.a.	MB
Total:			1912.036	2104.163	100.00	0.000	

DEFAULT/Integration — Chromeleon (c) Dionex 1996-2006 Version 6.80 SR8 Build 2623 (156243)

Operator:tjdgroup Timebase:TJGSUPERCOMP_1 Sequence:Project AA — Page 1-1 — 29/9/2011 9:07 AM

4 CW107-3

Sample Name:	CW107-3	Injection Volume:	20.0
Vial Number:	3	Channel:	UV_VIS_1
Sample Type:	unknown	Wavelength:	254
Control Program:	10pc 1200mL 10% IPA / heptane; 1.2 mL/min	Bandwidth:	n.a.
Quantif. Method:	Trial	Dilution Factor:	1.0000
Recording Time:	29/9/2011 8:39	Sample Weight:	1.0000
Run Time (min):	25.65	Sample Amount:	1.0000

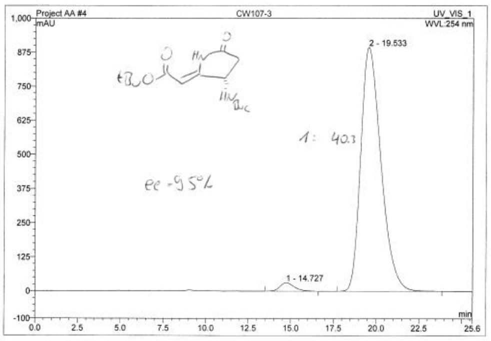

No.	Ret.Time min	Peak Name	Height mAU	Area mAU*min	Rel.Area %	Amount	Type
1	14.73	n.a.	31.400	30.352	2.42	n.a.	BMB
2	19.53	n.a.	893.652	1222.365	97.58	n.a.	BMB
Total:			925.052	1252.717	100.00	0.000	

DEFAULT/Integration — Chromeleon (c) Dionex 1996-2006 Version 6.80 SR8 Build 2623 (156243)

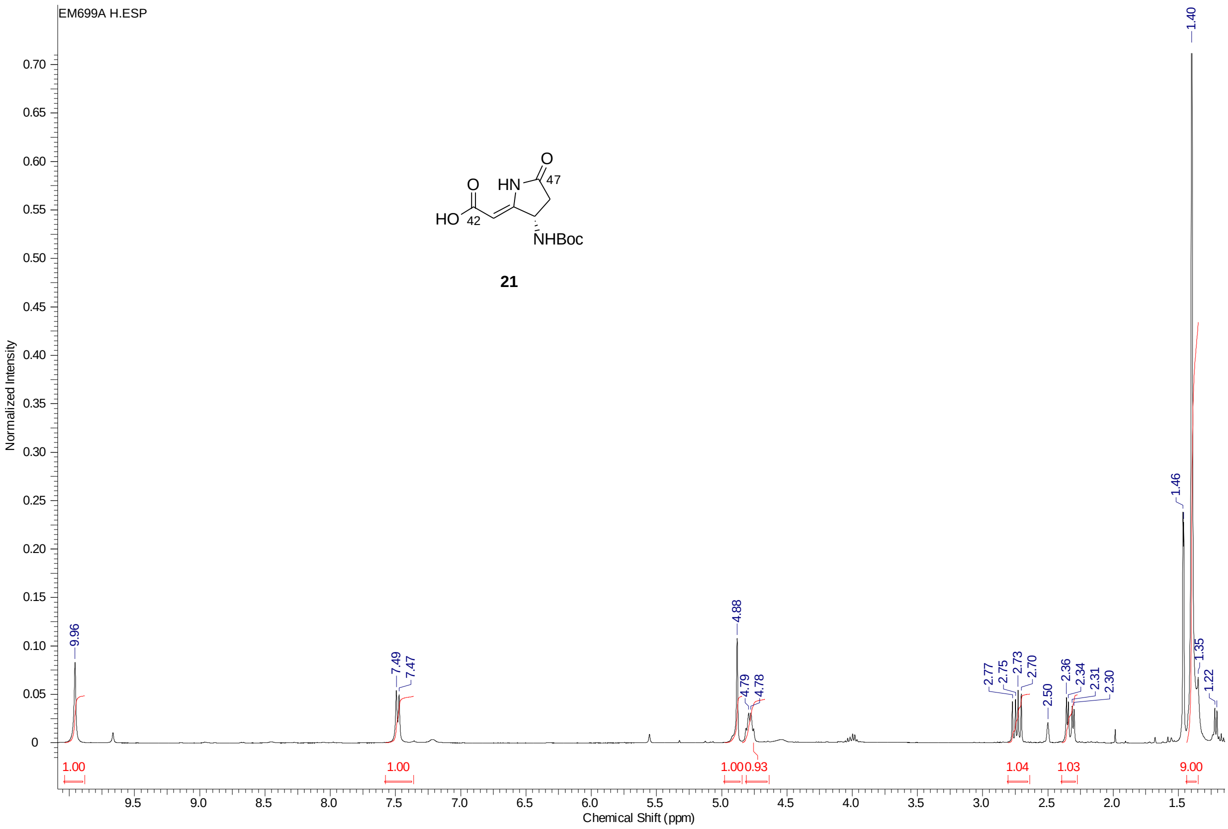
EM699A H.ESP
Normalized Intensity
Chemical Shift (ppm)
HN
O
HO
42
47
NHBoc
21
9.96
7.49
7.47
4.88
4.79
4.78
2.77
2.75
2.73
2.70
2.50
2.36
2.34
2.31
2.30
1.46
1.40
1.35
1.22
1.00
1.00
1.00
0.93
1.04
1.03
9.00

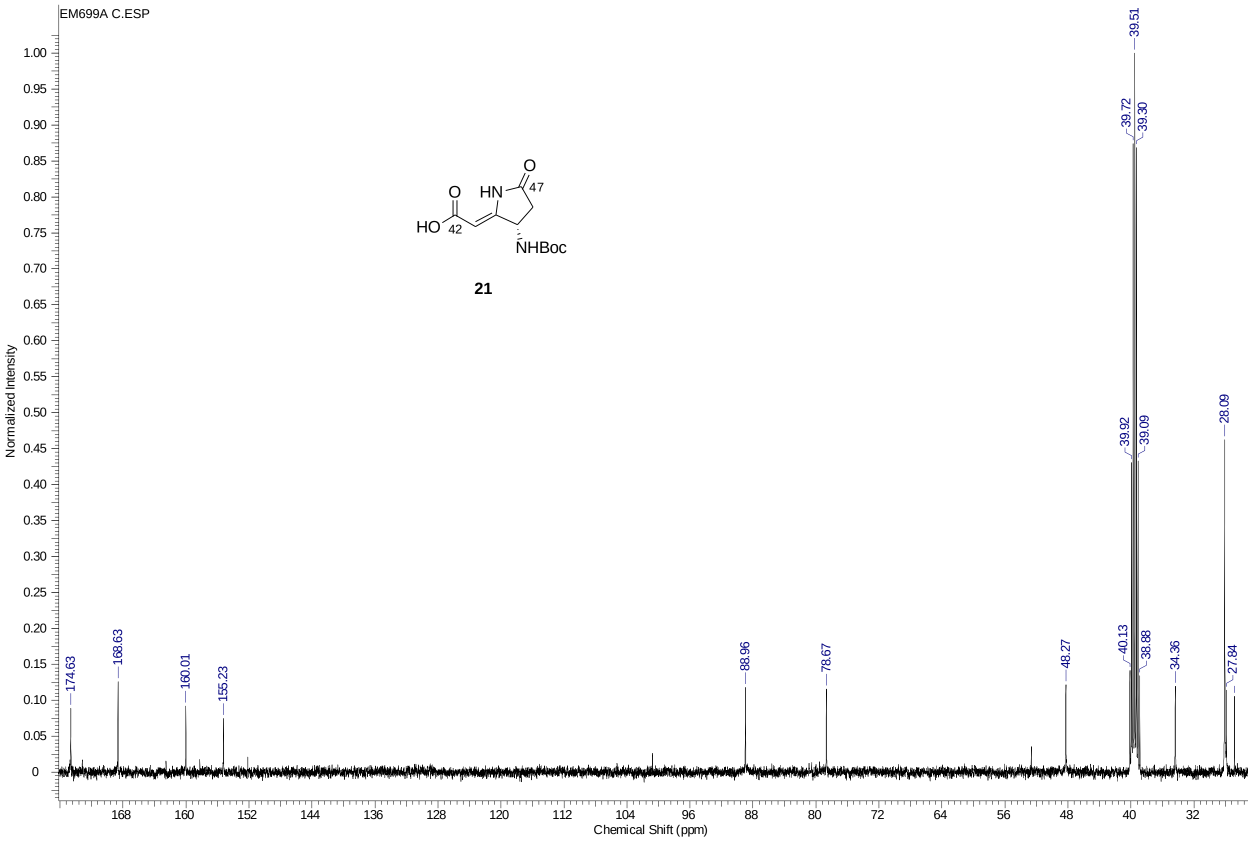
EM699A C.ESP
Normalized Intensity
Chemical Shift (ppm)
HN
O
HO
42
47
NHBoc
21
174.63
168.63
160.01
155.23
88.96
78.67
48.27
40.13
39.92
39.72
39.51
39.30
39.09
38.88
34.36
28.09
27.84

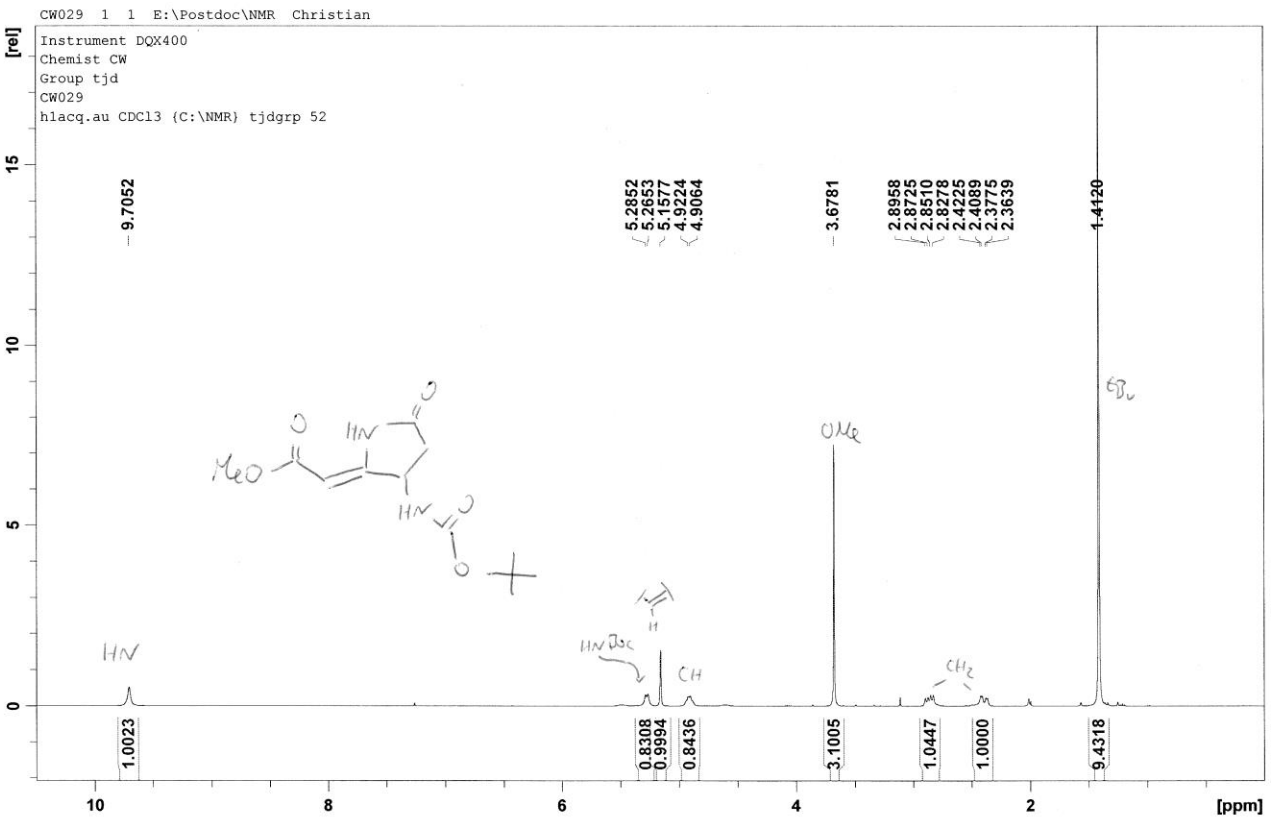

HPLC traces of a racemic sample of 21a (left) and a sample of 21a obtained from enantiopure pyrrolidinone acid 21 (right)

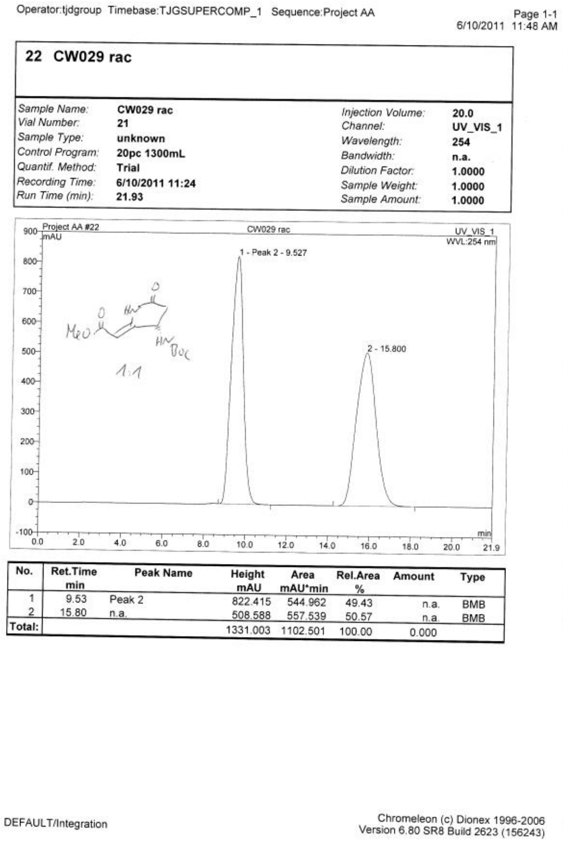

Operator:tjdgroup Timebase:TJGSUPERCOMP_1 Sequence:Project AA — Page 1-1, 6/10/2011 11:48 AM

22 CW029 rac

Sample Name:	CW029 rac	Injection Volume:	20.0
Vial Number:	21	Channel:	UV_VIS_1
Sample Type:	unknown	Wavelength:	254
Control Program:	20pc 1300mL	Bandwidth:	n.a.
Quantif. Method:	Trial	Dilution Factor:	1.0000
Recording Time:	6/10/2011 11:24	Sample Weight:	1.0000
Run Time (min):	21.93	Sample Amount:	1.0000

No.	Ret.Time min	Peak Name	Height mAU	Area mAU*min	Rel.Area %	Amount	Type
1	9.53	Peak 2	822.415	544.962	49.43	n.a.	BMB
2	15.80	n.a.	508.588	557.539	50.57	n.a.	BMB
Total:			1331.003	1102.501	100.00	0.000	

DEFAULT/Integration — Chromeleon (c) Dionex 1996-2006 Version 6.80 SR8 Build 2623 (156243)

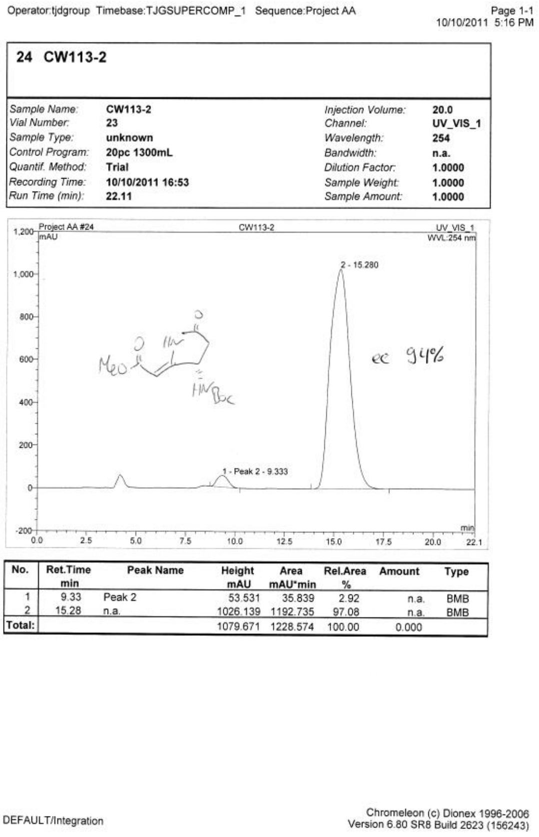

Operator:tjdgroup Timebase:TJGSUPERCOMP_1 Sequence:Project AA — Page 1-1, 10/10/2011 5:16 PM

24 CW113-2

Sample Name:	CW113-2	Injection Volume:	20.0
Vial Number:	23	Channel:	UV_VIS_1
Sample Type:	unknown	Wavelength:	254
Control Program:	20pc 1300mL	Bandwidth:	n.a.
Quantif. Method:	Trial	Dilution Factor:	1.0000
Recording Time:	10/10/2011 16:53	Sample Weight:	1.0000
Run Time (min):	22.11	Sample Amount:	1.0000

No.	Ret.Time min	Peak Name	Height mAU	Area mAU*min	Rel.Area %	Amount	Type
1	9.33	Peak 2	53.531	35.839	2.92	n.a.	BMB
2	15.28	n.a.	1026.139	1192.735	97.08	n.a.	BMB
Total:			1079.671	1228.574	100.00	0.000	

DEFAULT/Integration — Chromeleon (c) Dionex 1996-2006 Version 6.80 SR8 Build 2623 (156243)

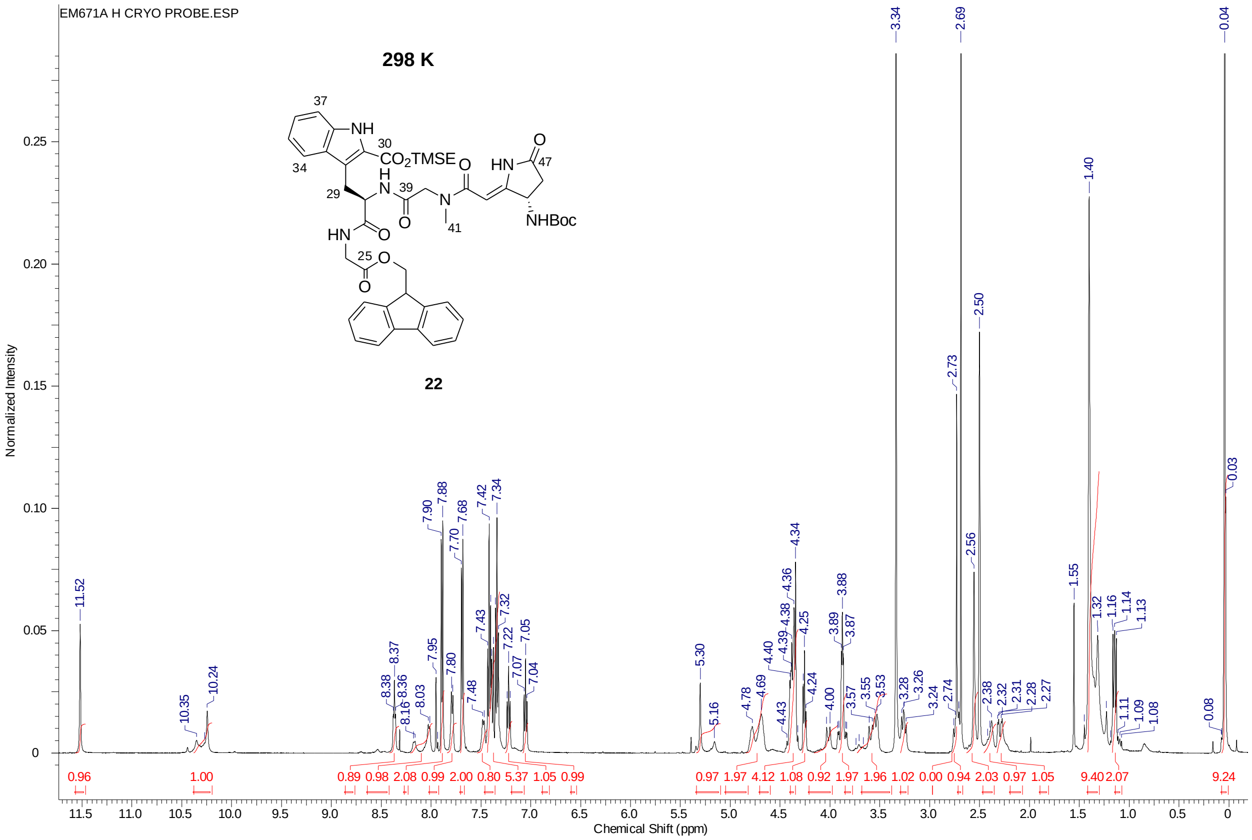

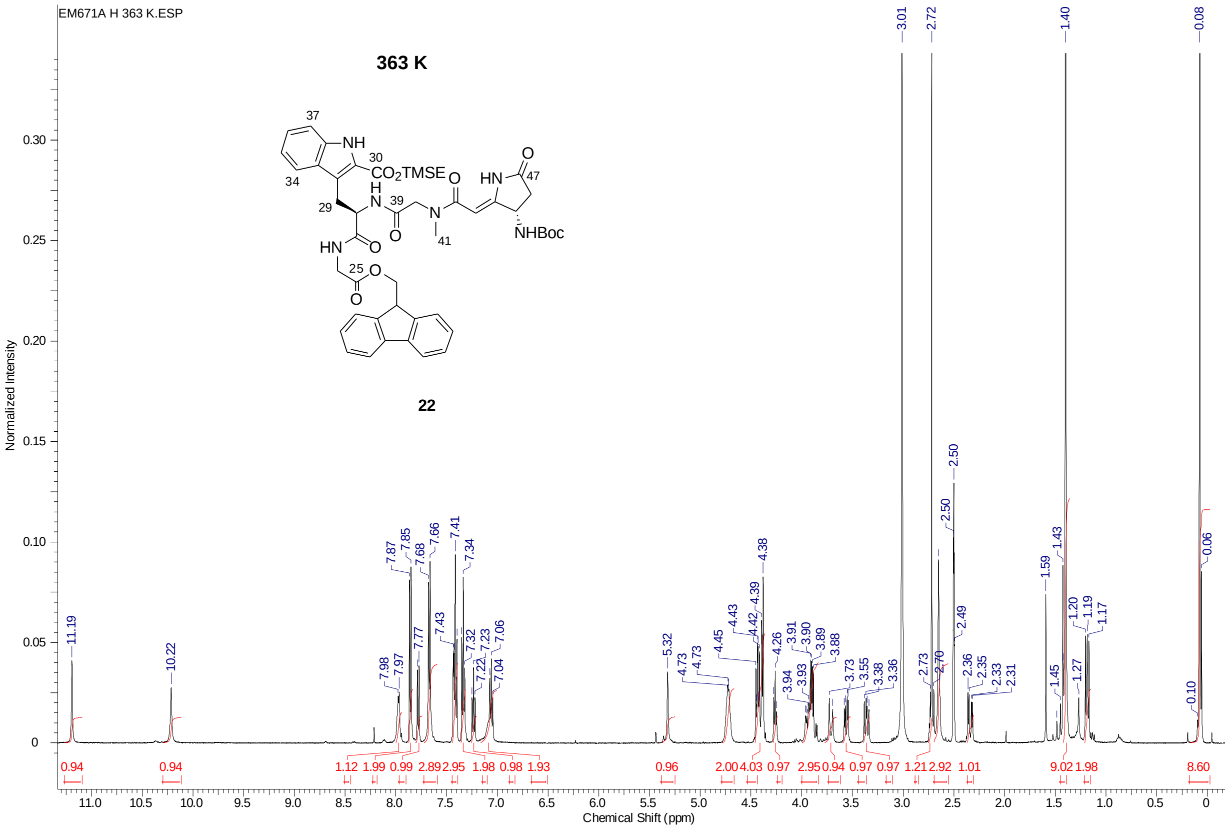

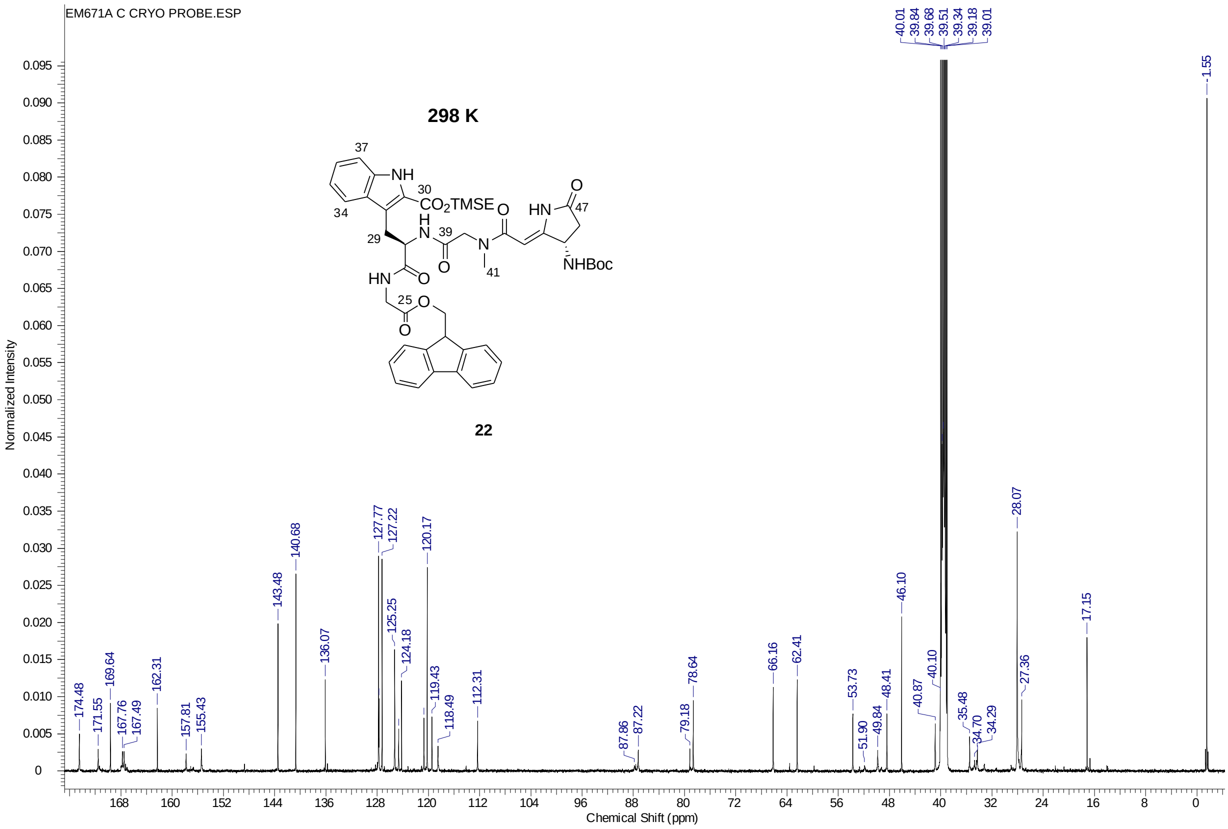

EM671A C CRYO PROBE.ESP
298 K
22
Normalized Intensity
Chemical Shift (ppm)

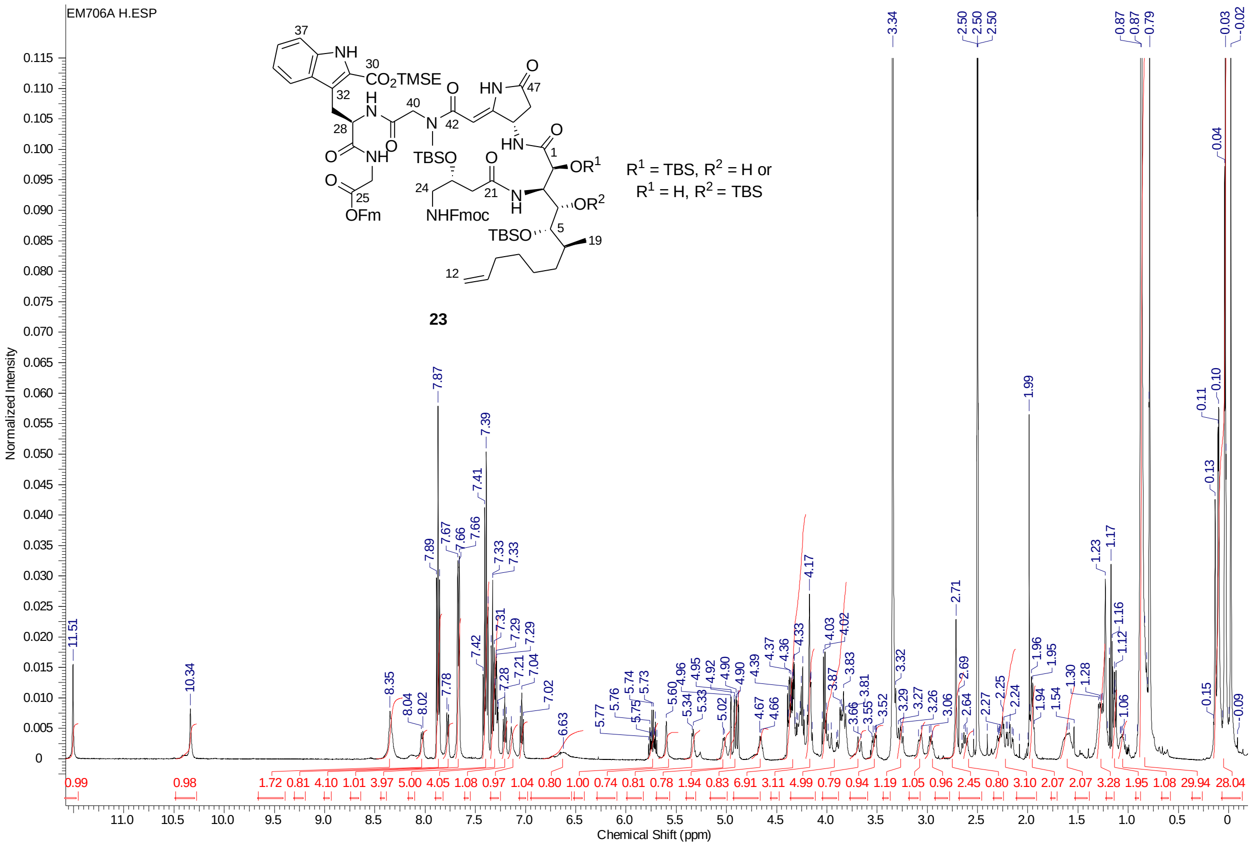

EM706A H.ESP
R1 = TBS, R2 = H or
R1 = H, R2 = TBS
23
Normalized Intensity
Chemical Shift (ppm)

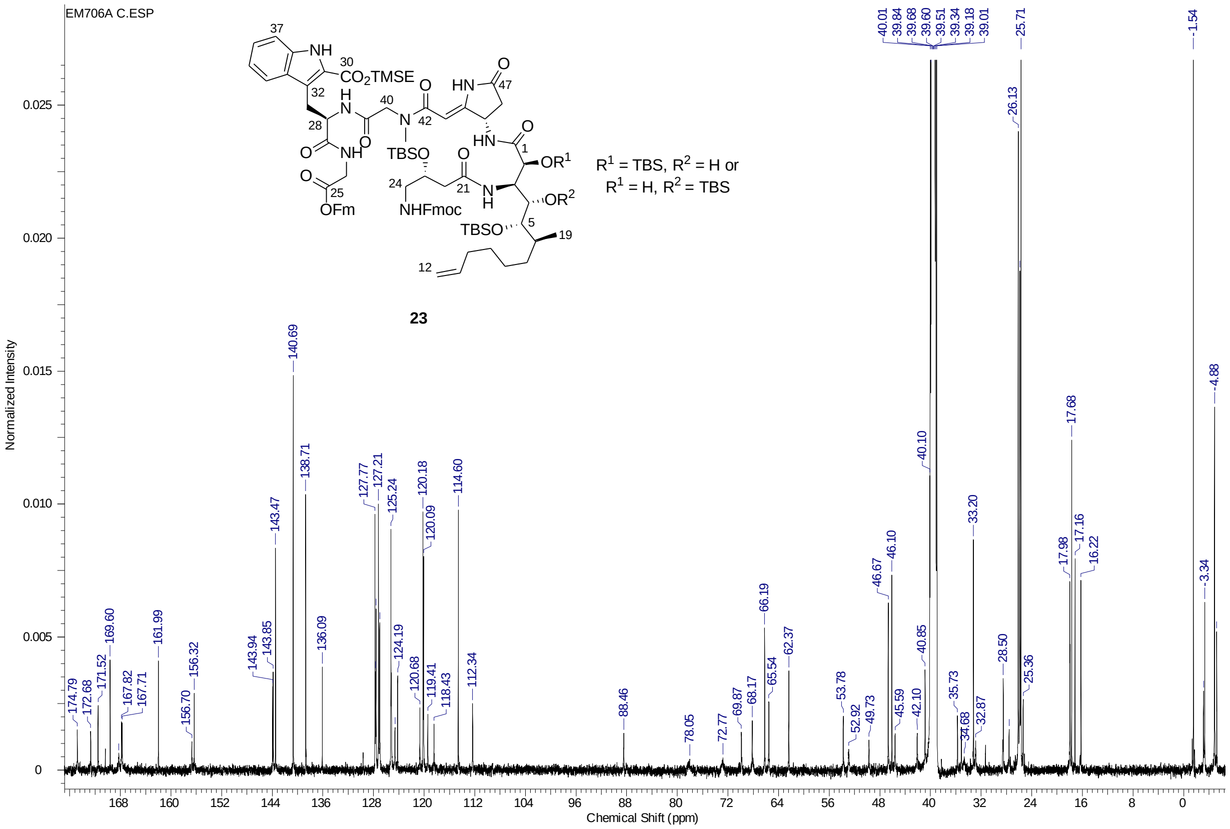
EM706A C.ESP
R^1 = TBS, R^2 = H or
R^1 = H, R^2 = TBS
23
Normalized Intensity
Chemical Shift (ppm)

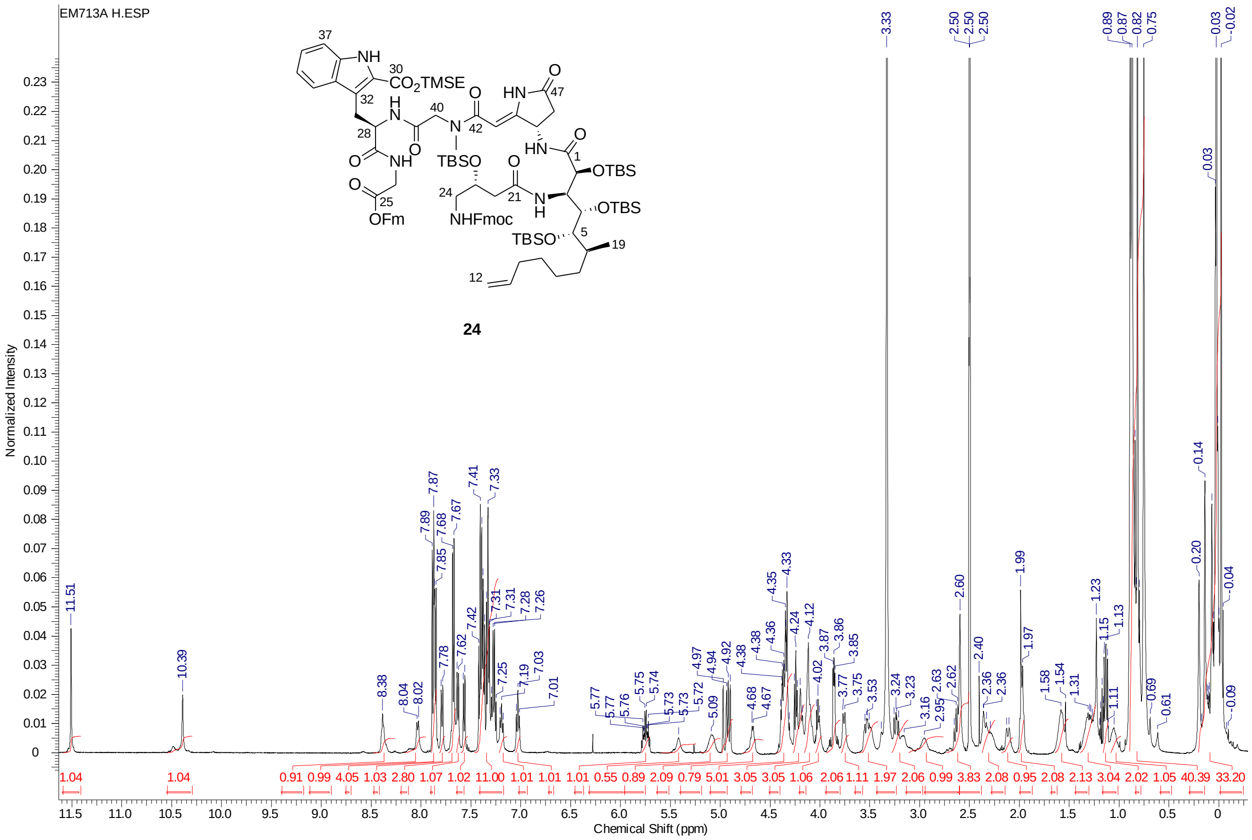
EM713A H.ESP
24
Normalized Intensity
Chemical Shift (ppm)

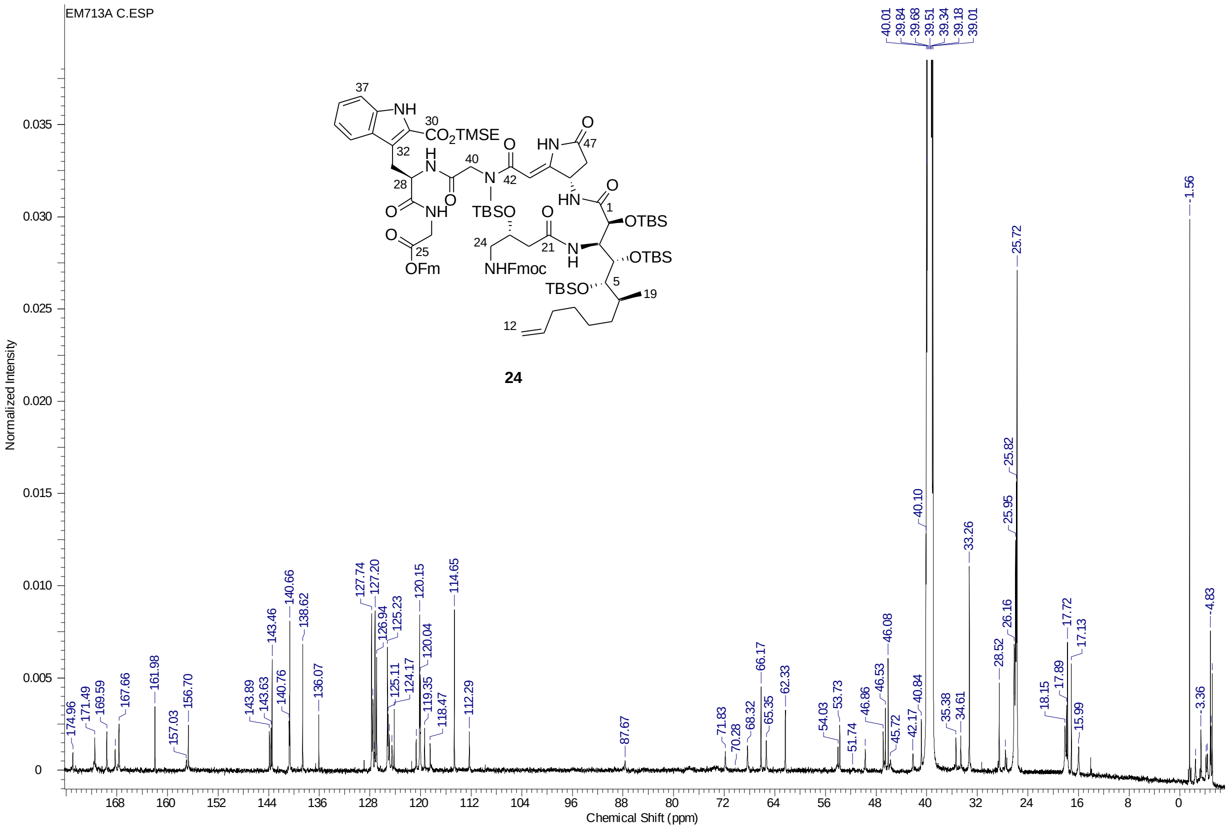
EM713A C.ESP
Normalized Intensity
Chemical Shift (ppm)
24
NH
CO2TMSE
HN
OTBS
TBSO
NHFmoc
OFm
40.01 39.84 39.68 39.51 39.34 39.18 39.01
174.96 171.49 169.59 167.66 161.98 157.03 156.70
143.89 143.63 143.46 140.76 140.66 138.62 136.07
127.74 127.20 126.94 125.23 125.11 124.17 120.15 120.04 119.35 118.47 114.65 112.29
87.67
71.83 70.28 68.32 66.17 65.35 62.33
54.03 53.73 51.74 46.86 46.53 46.08 45.72 42.17 40.84 40.10
35.38 34.61 33.26 28.52 26.16 25.95 25.82 25.72
18.15 17.89 17.72 17.13 15.99
-1.56 -3.36 -4.83

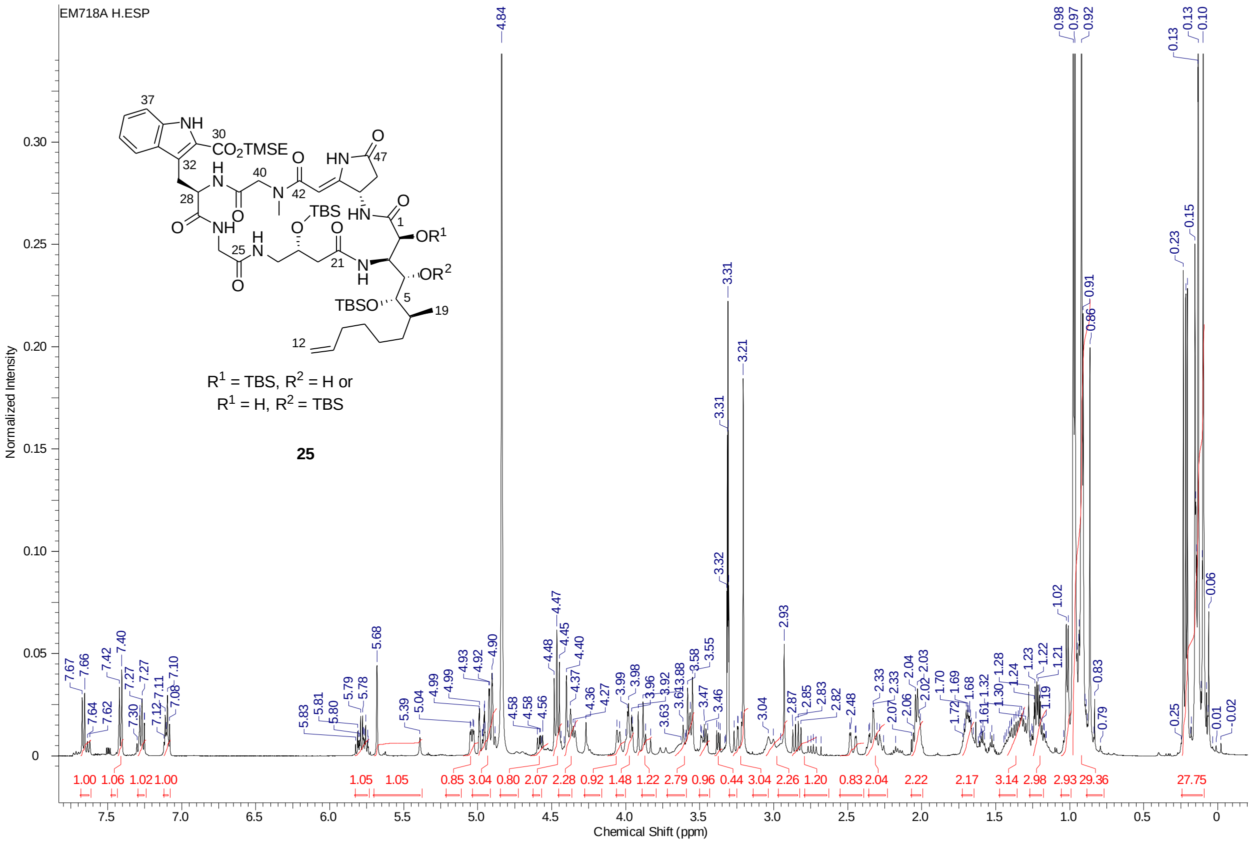
EM718A H.ESP
Normalized Intensity
Chemical Shift (ppm)
R1 = TBS, R2 = H or
R1 = H, R2 = TBS
25
NH
CO2TMSE
HN
TBS
OR1
OR2
TBSO
4.84
0.98 0.97 0.92
0.13 0.13 0.10
3.31
3.21
0.23 0.15
0.91 0.86
1.02
0.06
5.68
4.47 4.45 4.48 4.40
2.93

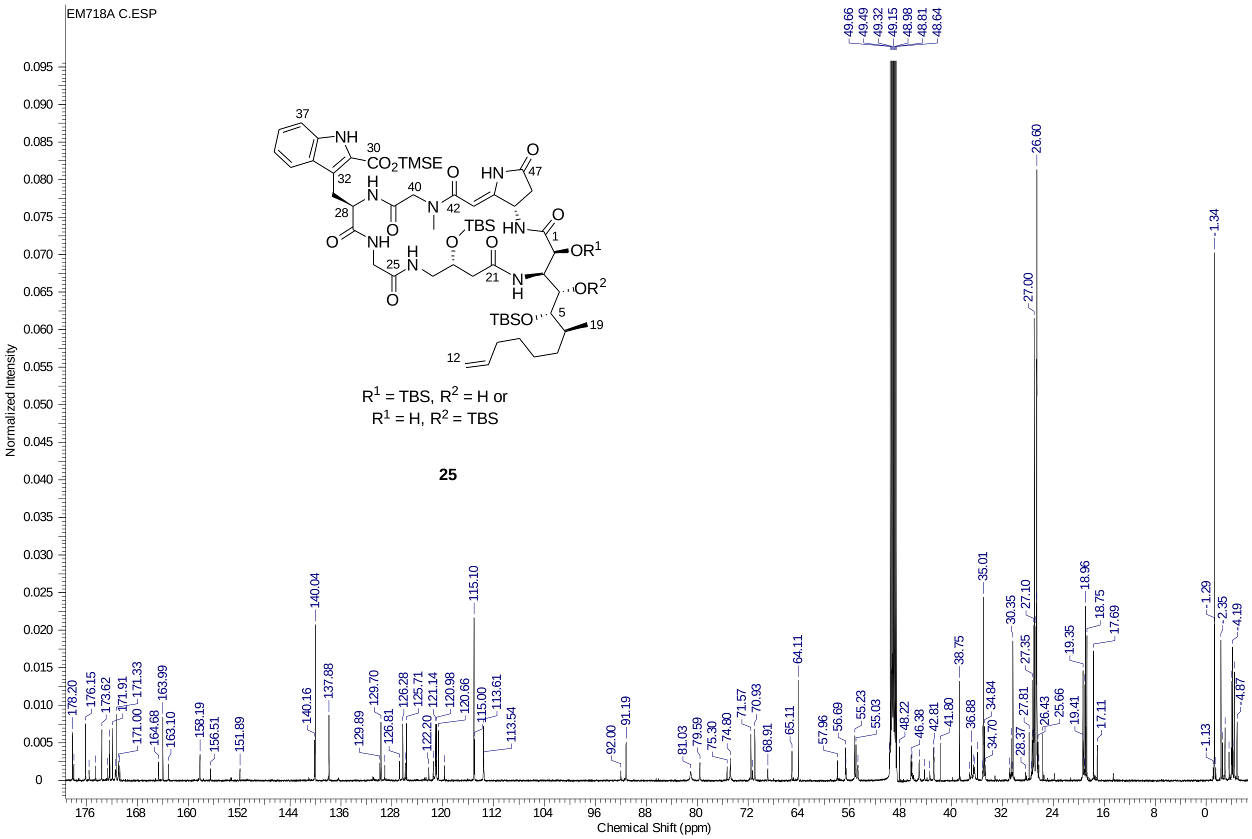
EM718A C.ESP
Normalized Intensity
Chemical Shift (ppm)
CO2TMSE
TBS
OR1
OR2
TBSO
R1 = TBS, R2 = H or
R1 = H, R2 = TBS
25

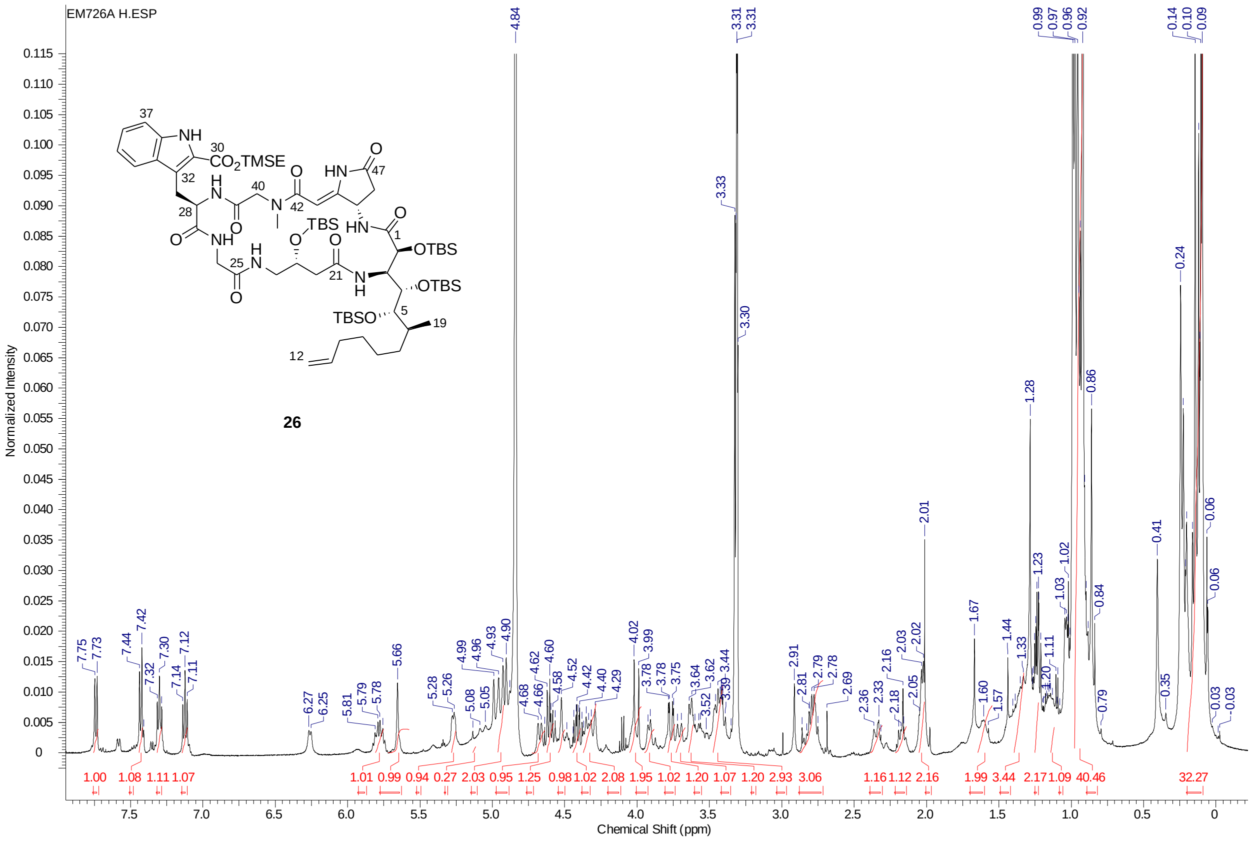
EM726A H.ESP
Normalized Intensity
Chemical Shift (ppm)
CO2TMSE
TBS
OTBS
OTBS
TBSO
26

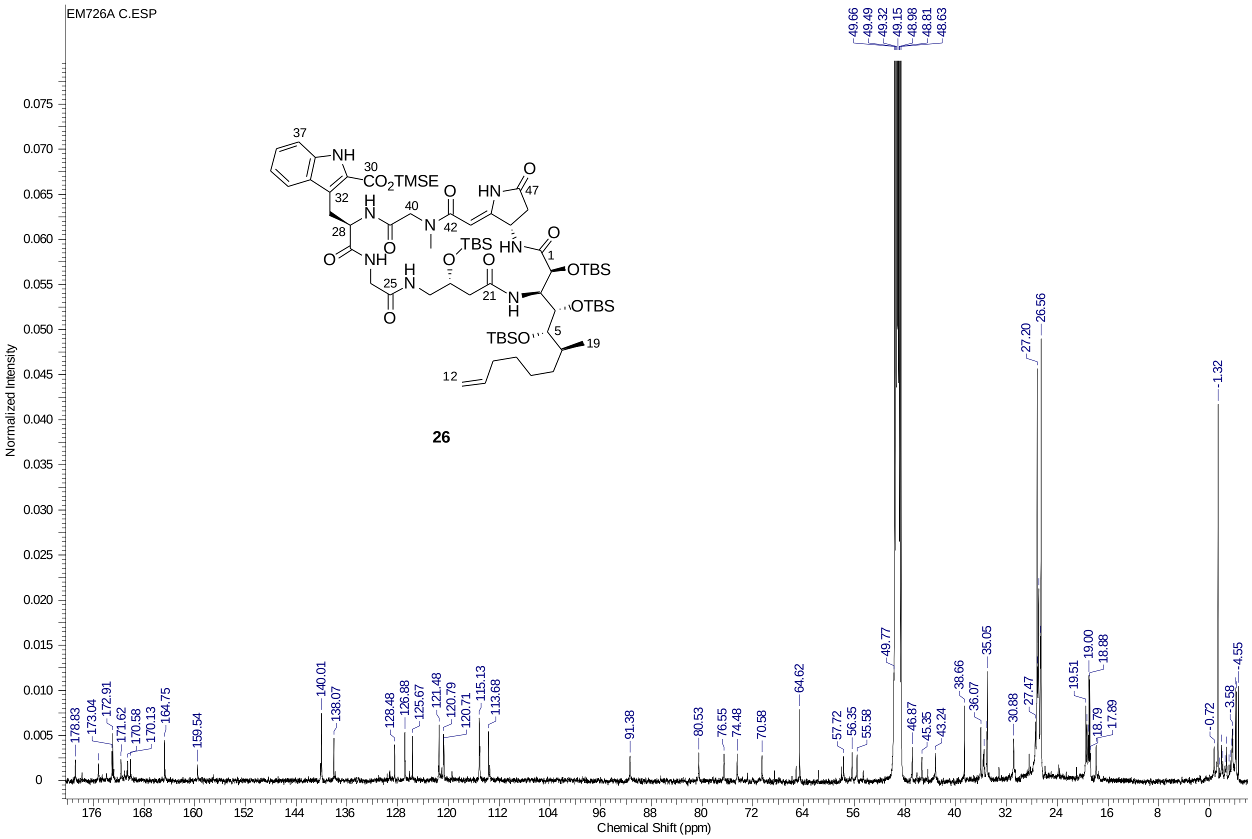
EM726A C.ESP
Normalized Intensity
Chemical Shift (ppm)
26

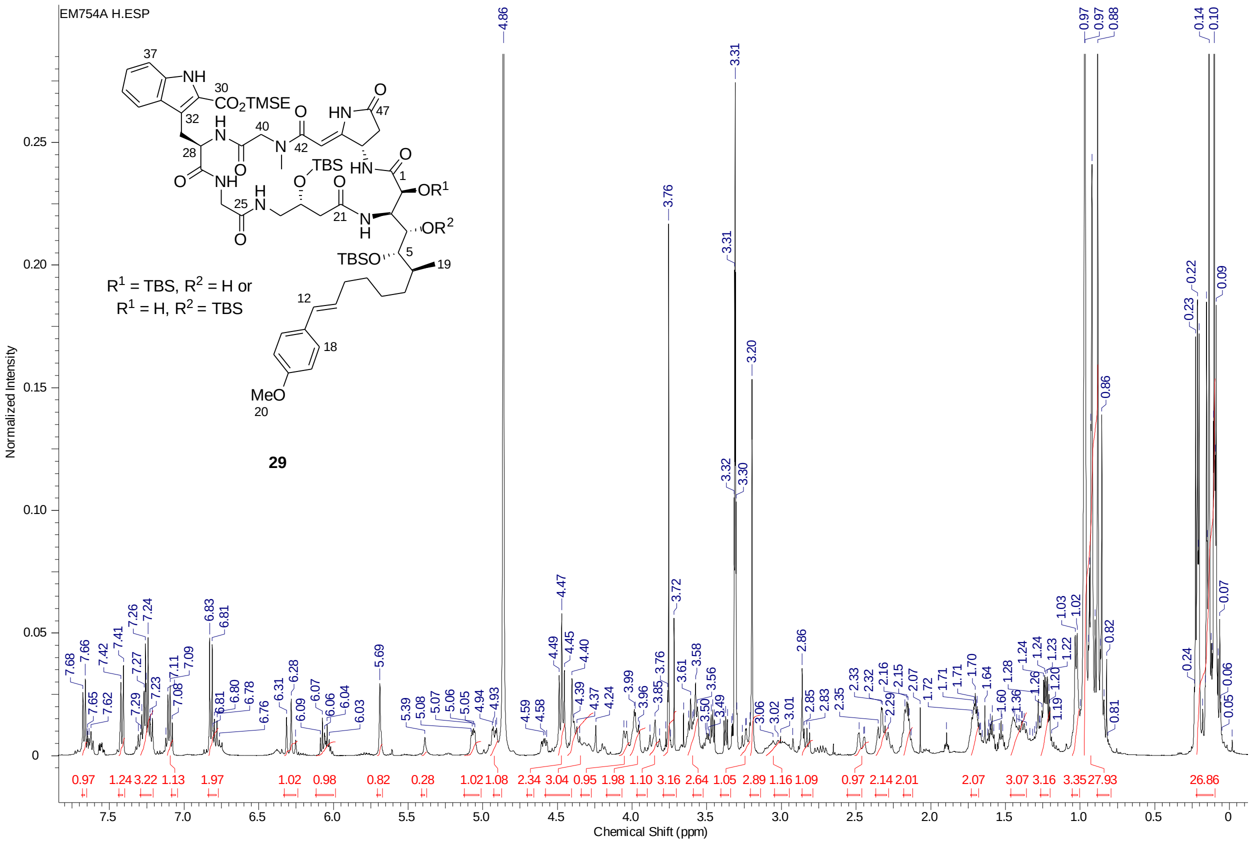
EM754A H.ESP
Normalized Intensity
Chemical Shift (ppm)
R1 = TBS, R2 = H or
R1 = H, R2 = TBS
29

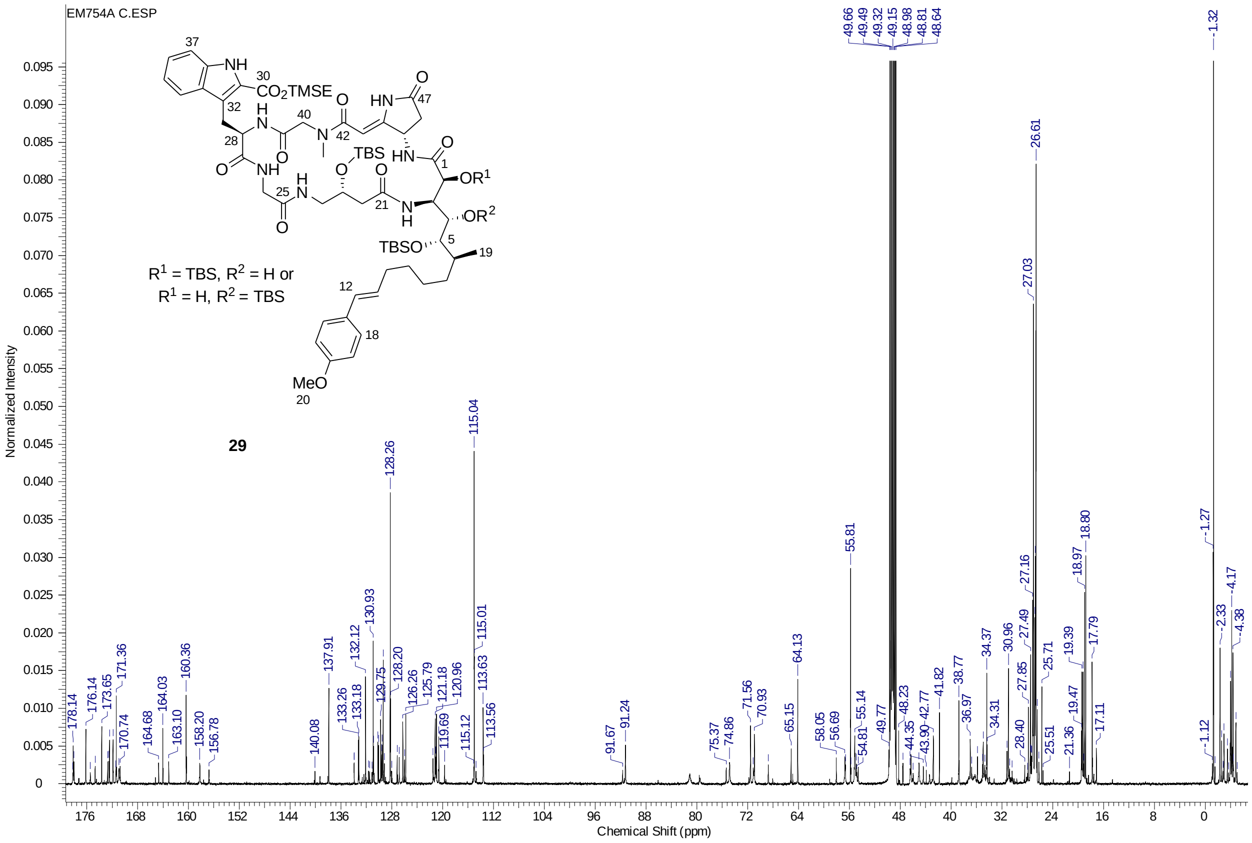
EM754A C.ESP
Normalized Intensity
Chemical Shift (ppm)
CO2TMSE
TBSO
OR1
OR2
R1 = TBS, R2 = H or
R1 = H, R2 = TBS
MeO
29

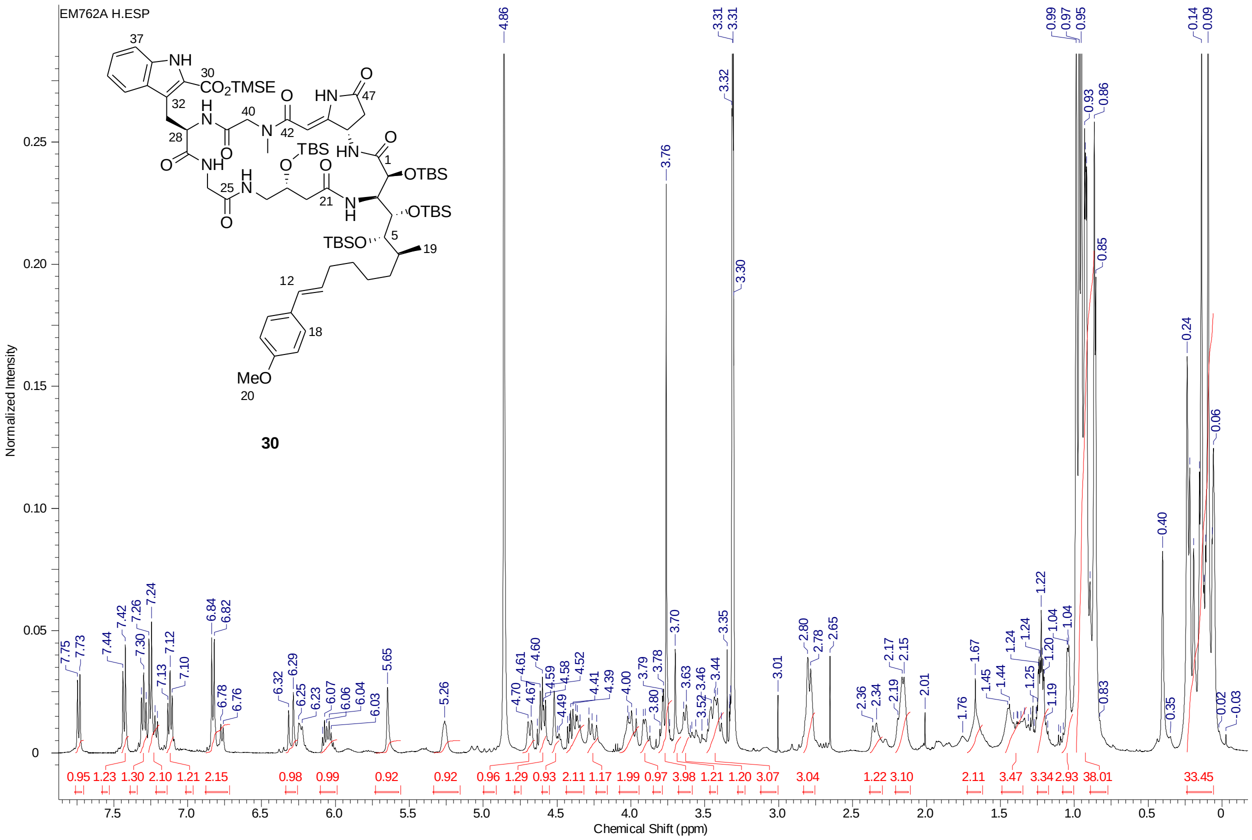
EM762A H.ESP
Normalized Intensity
Chemical Shift (ppm)
CO2TMSE
OTBS
OTBS
TBSO
MeO
30

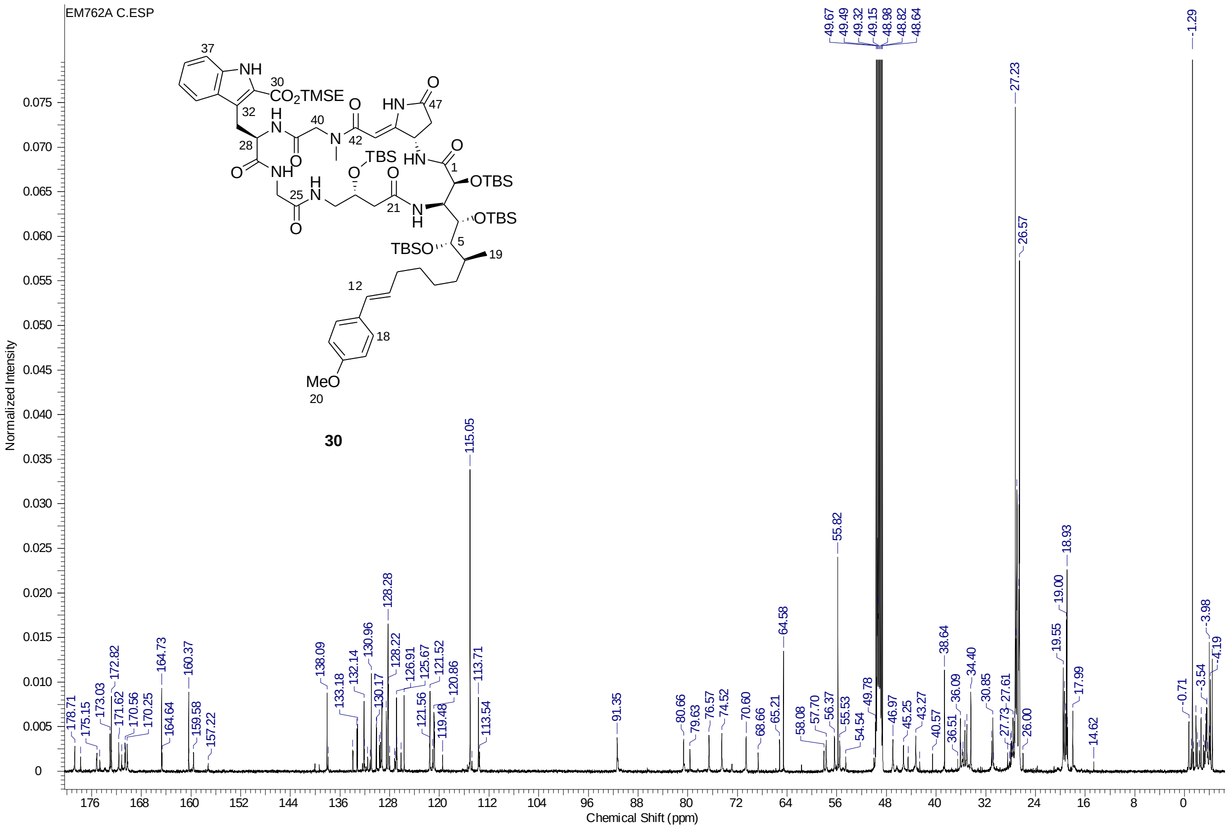
EM762A C.ESP
Normalized Intensity
Chemical Shift (ppm)
CO2TMSE
OTBS
TBSO
MeO
30

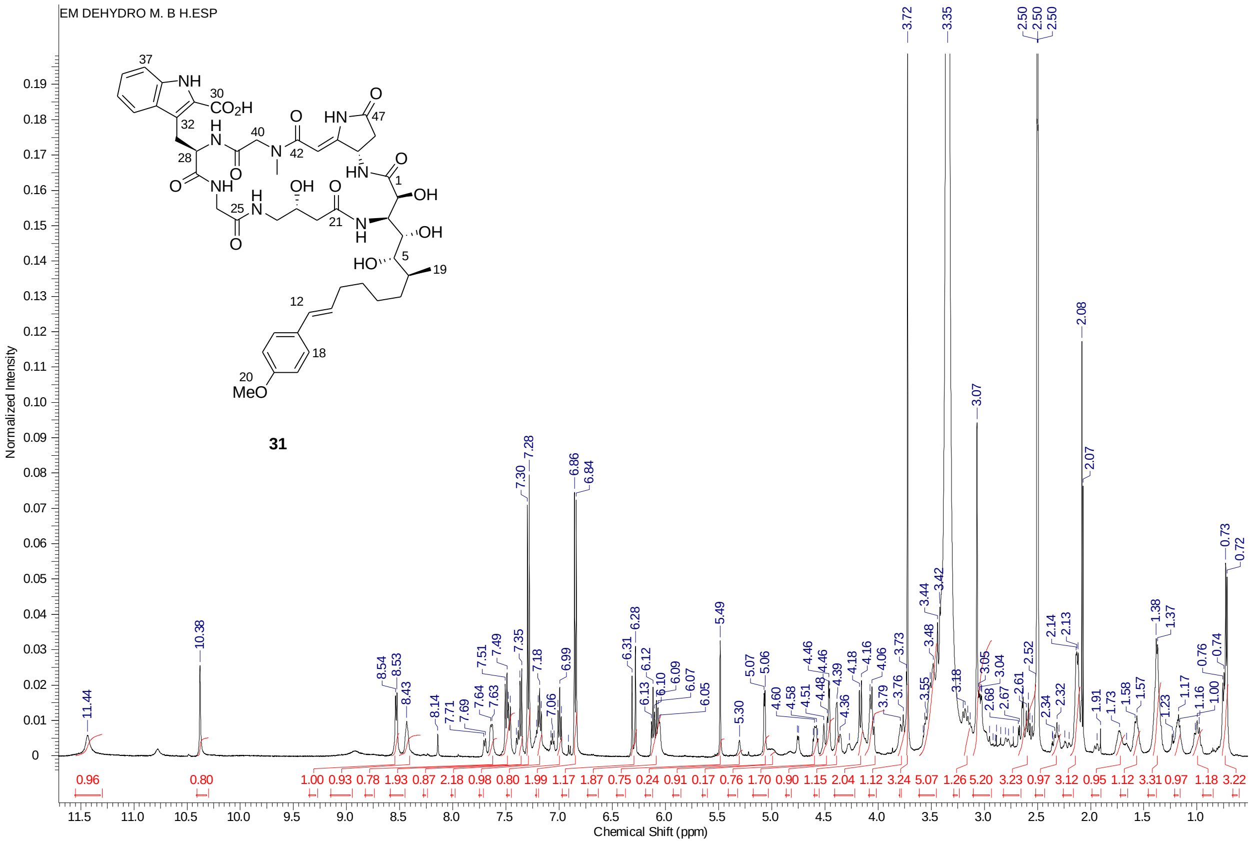
EM DEHYDRO M. B H.ESP
Normalized Intensity
Chemical Shift (ppm)
CO2H
OH
HO
MeO
31

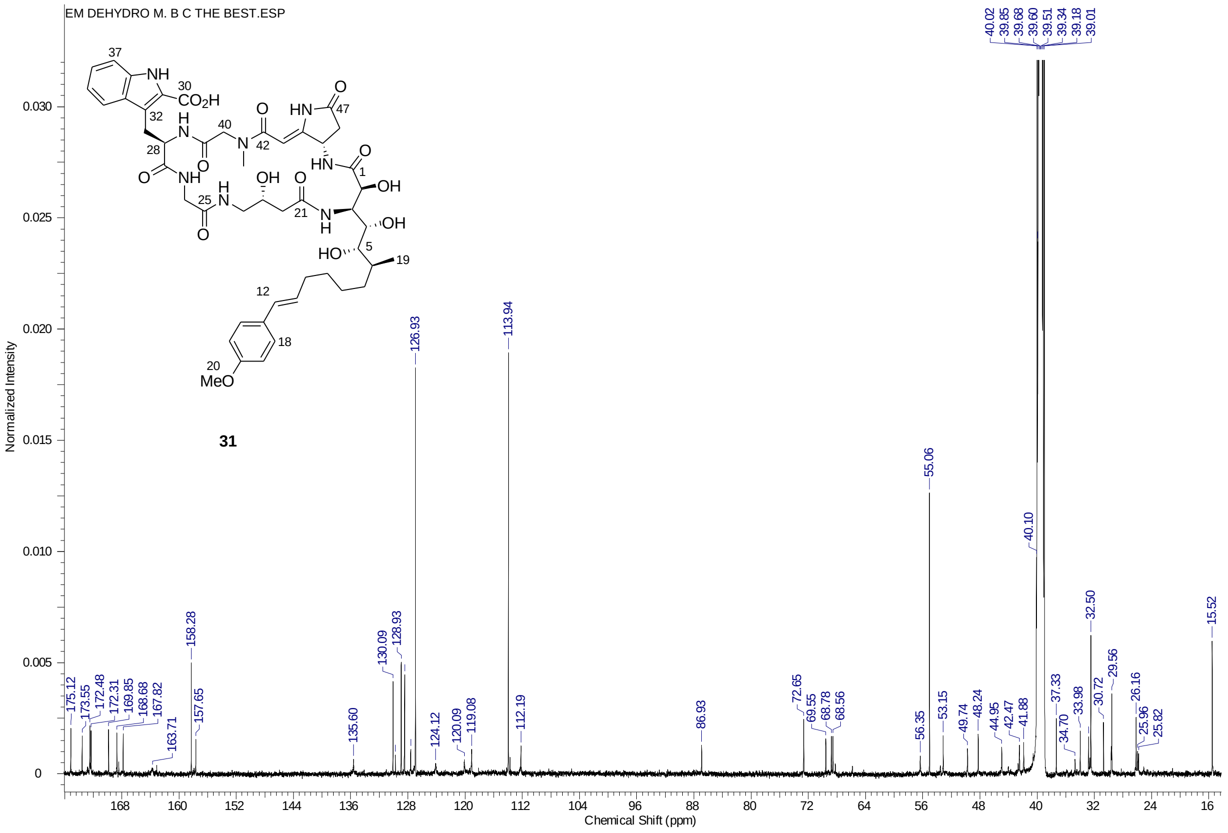

^{13}C NMR spectrum of natural dehydromicrosclerodermin B (provided by Li)

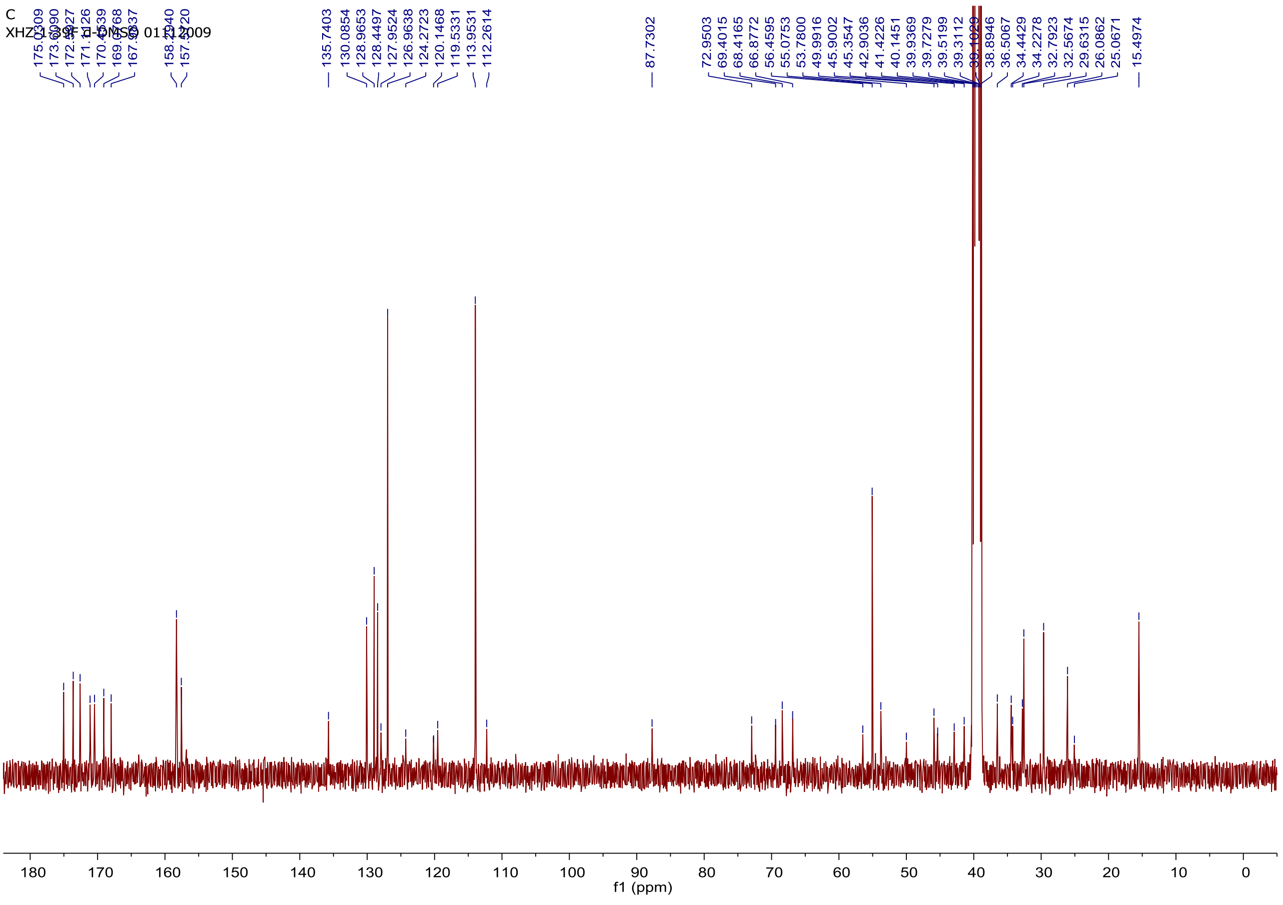

Comparison of ^{13}C NMR of synthetic 31 with natural dehydromicrosclerodermin B

Note: C30, C31 and C32 were not identified in the spectrum of natural dehydromicrosclerodermin B, C31 and C32 were not identified in the spectrum of synthetic **31**.

Carbon №	$\delta_{C(synthetic\ 31)}$	$\delta_{C(natural)}$	$\Delta\delta_c$, ppm
1	172.5	173.6	-1.1
2	69.6	69.4	0.2
3	53.2	53.8	-0.6
4	68.8	68.4	0.4
5	72.7	73	-0.3
6	34	34.4	-0.4
7	32.8	32.8	0
8	26.2	26.1	0.1
9	29.6	29.6	0
10	32.5	32.6	-0.1
11	128.5	128.5	0
12	128.9	129	-0.1
13	130.1	130.1	0
14	126.9	127	-0.1
15	113.9	114	-0.1
16	158.3	158.3	0
17	113.9	114	-0.1
18	126.9	127	-0.1
19	15.5	15.5	0
20	55.1	55.1	0
21	173.6	172.6	1
22	41.9	41.4	0.5
23	68.6	66.9	1.7
24	45	45.4	-0.4
25	168.7	169.1	-0.4
26	42.5	42.9	-0.4
27	172.3	171.1	1.2
28	56.4	56.5	-0.1
29	26	25.1	0.9
33	127.6	128	-0.4
34	120.1	120.2	-0.1
35	119.1	119.5	-0.4
36	124.1	124.3	-0.2
37	112.2	112.3	-0.1
38	135.6	135.7	-0.1
39	169.9	170.5	-0.6
40	49.7	50	-0.3
41	37.3	36.5	0.8
42	167.8	168	-0.2
43	86.9	87.7	-0.8
44	157.7	157.6	0.1
45	48.2	45.9	2.3
46	32.5	34.2	-1.7
47	175.1	175	0.1

HPLC traces of (A) synthetic 31, (B) natural dehydromicrosclerodermin B and (C) a 1:1 mixture.

HPLC conditions: Phenomenex Jupiter 4u Proteo 90A 250 x 4.60 mm 4 micron column at 1.0 mL/min with UV detection 254 nm: 2 mins – 5% CH_3CN in 0.05% aqueous TFA, linear increase; 3 mins – 42% CH_3CN in 0.05% aqueous TFA, isocratic; 23 mins – 42% CH_3CN in 0.05% aqueous TFA, linear increase; 24 mins – 95% CH_3CN in 0.05% aqueous TFA, isocratic.

A.

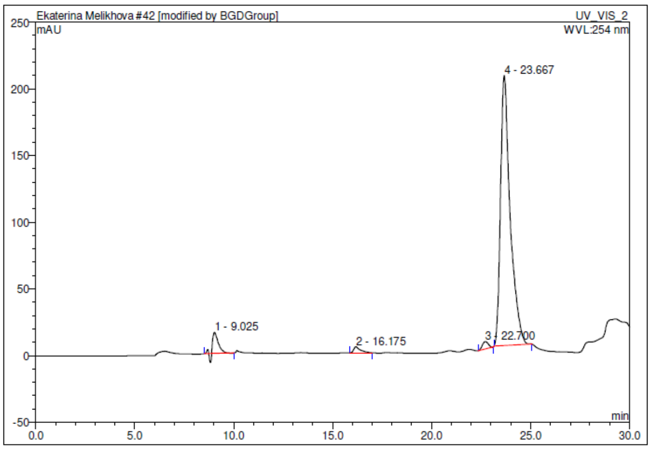

B.

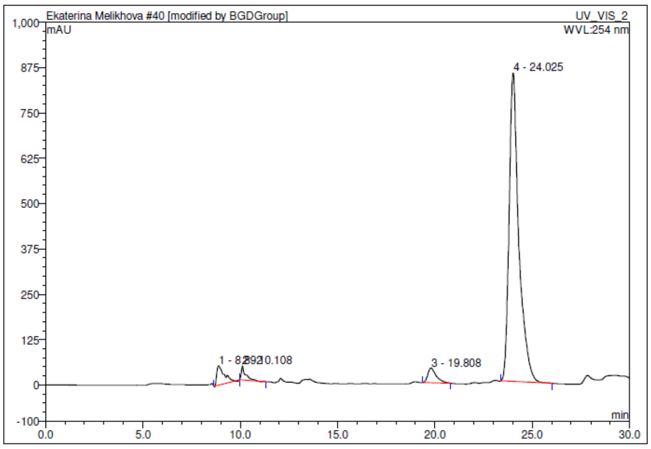

C.

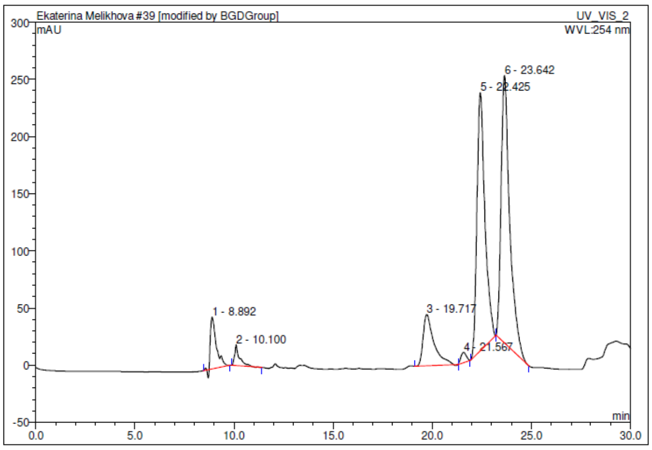

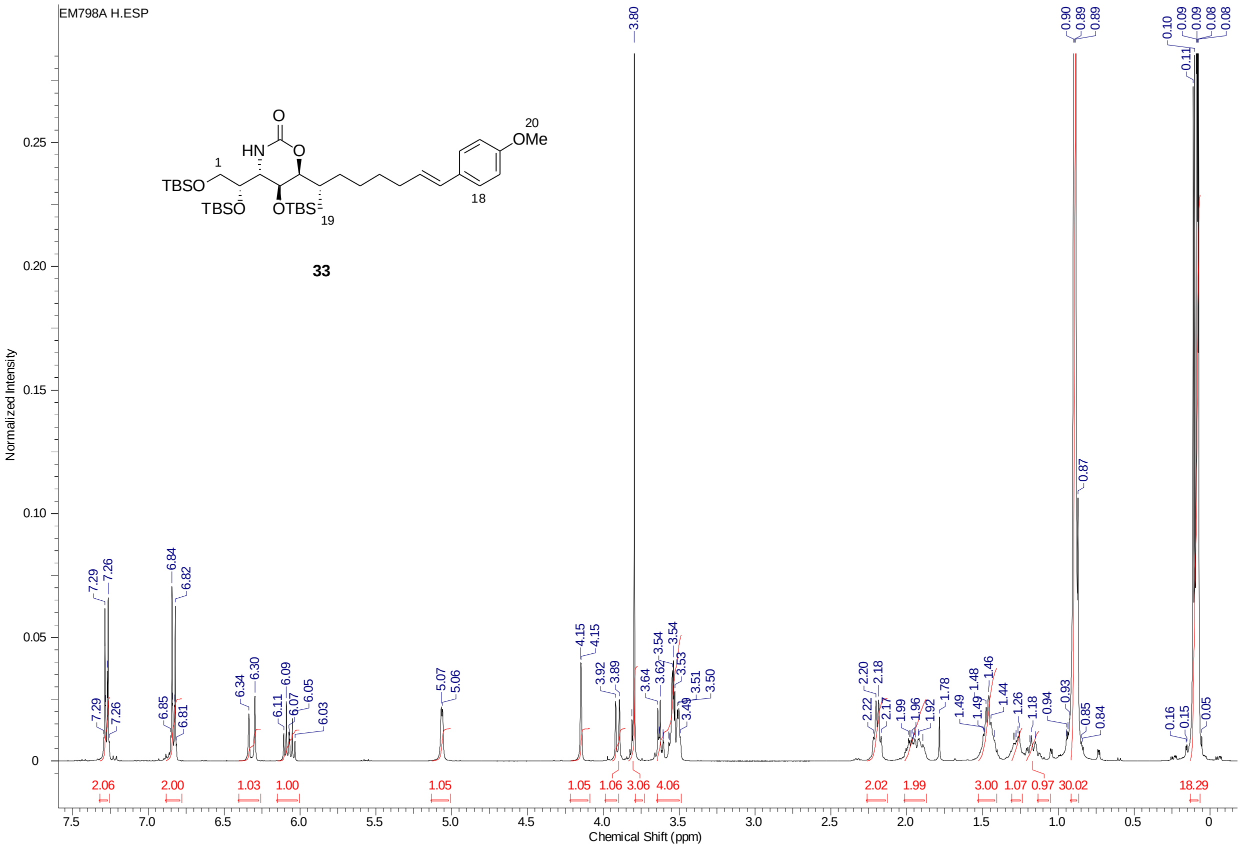
EM798A H.ESP
Normalized Intensity
Chemical Shift (ppm)
TBSO
TBSO
OTBS
HN
O
O
OMe
1
18
19
20
33

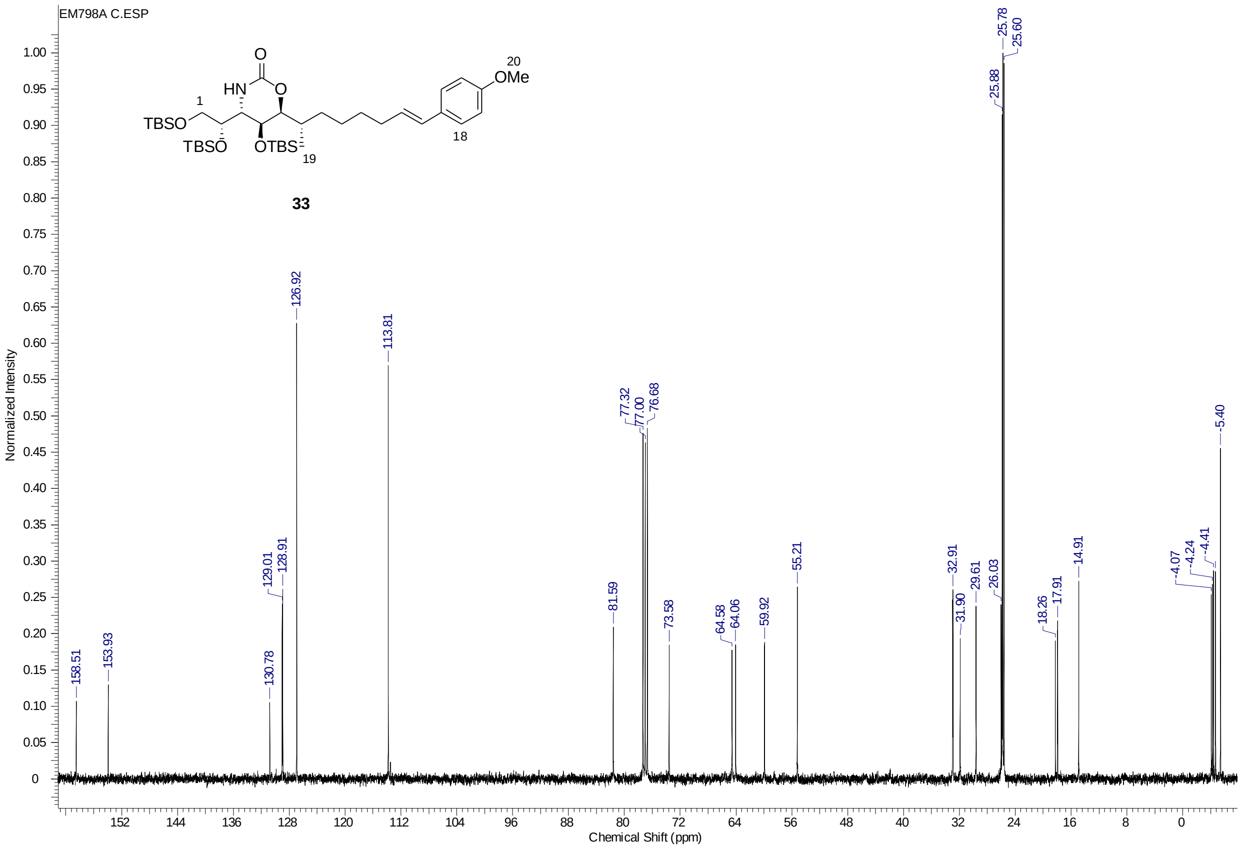
EM798A C.ESP
Normalized Intensity
Chemical Shift (ppm)
TBSO
TBSO
OTBS
HN
O
O
OMe
1
18
19
20
33

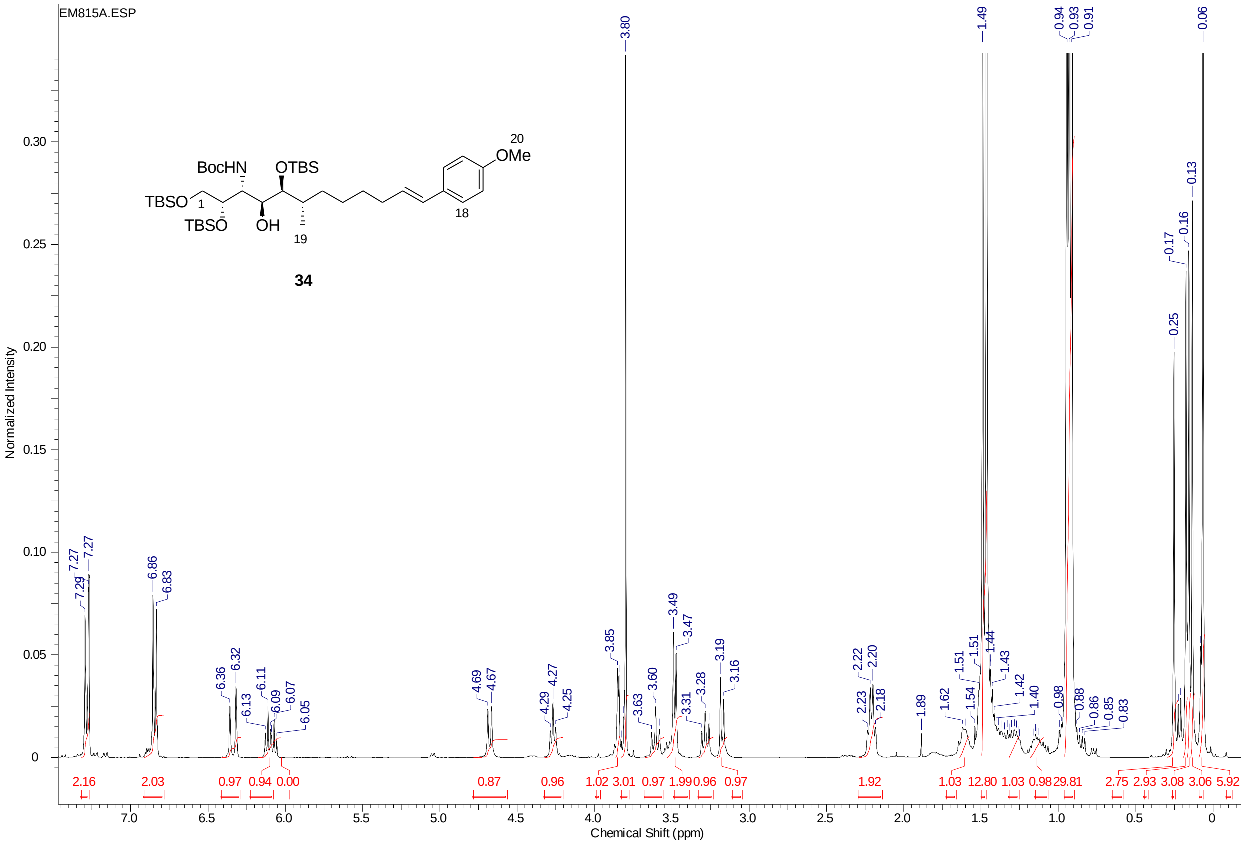
EM815A.ESP
BocHN
OTBS
OMe
20
TBSO
1
TBSO
OH
19
18
34
Normalized Intensity
Chemical Shift (ppm)

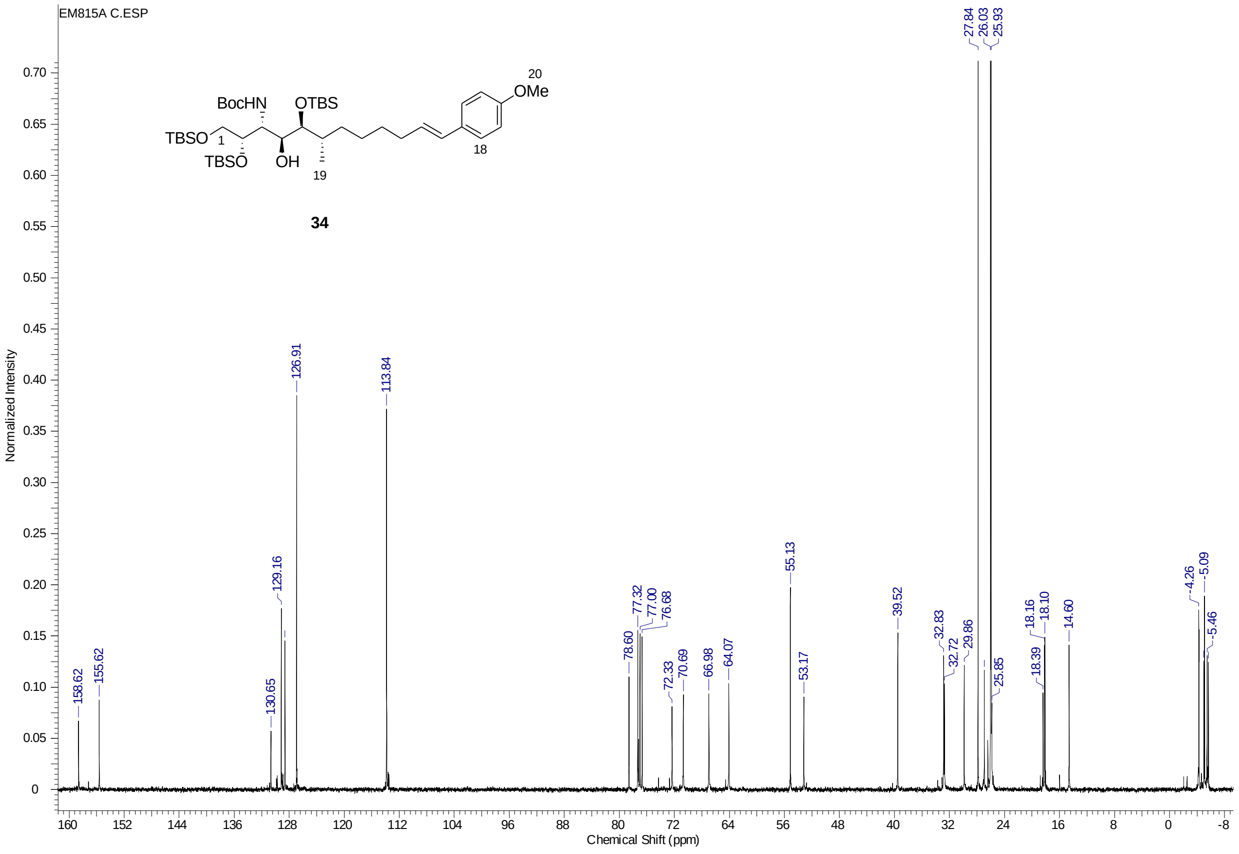
EM815A C.ESP
BocHN
OTBS
OMe
20
TBSO
1
TBSO
OH
19
18
34
Normalized Intensity
Chemical Shift (ppm)

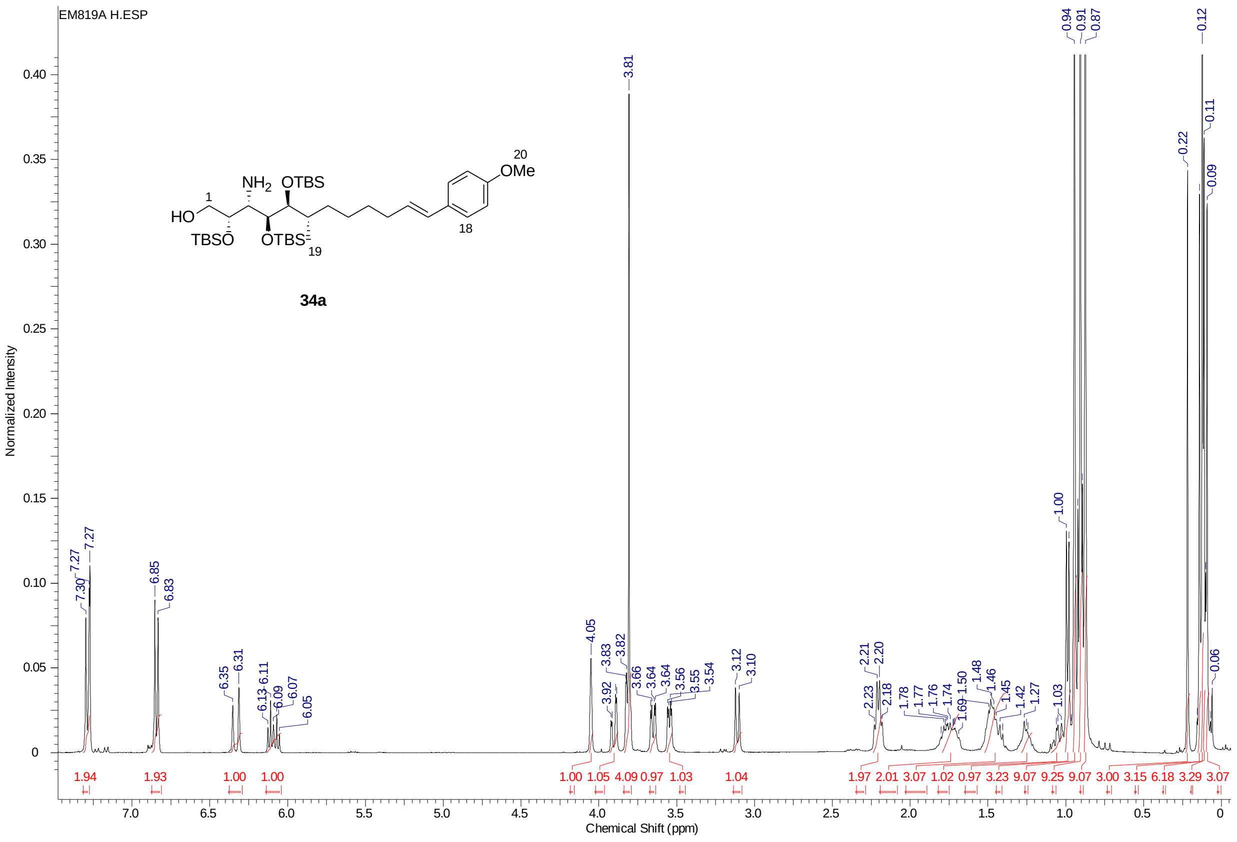
EM819A H.ESP
34a
Normalized Intensity
Chemical Shift (ppm)

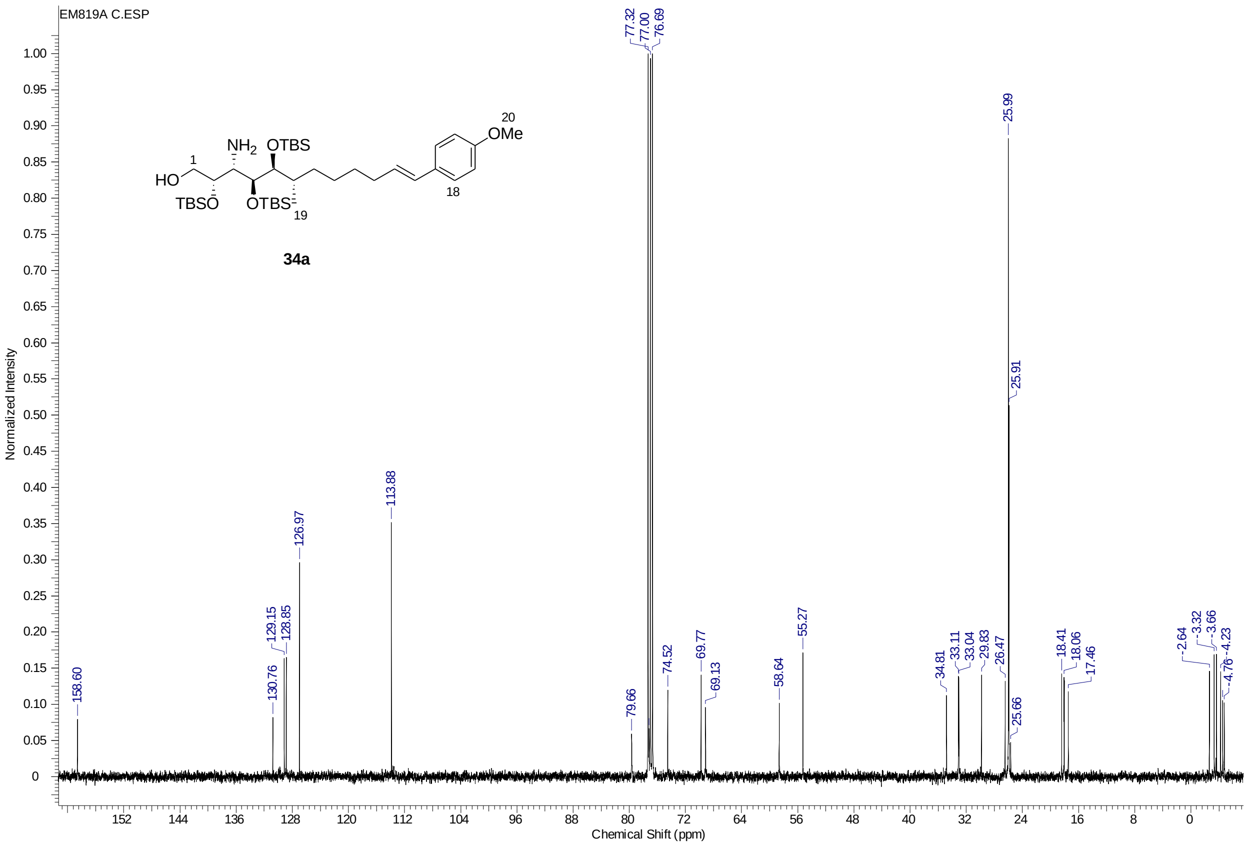
EM819A C.ESP
34a
Normalized Intensity
Chemical Shift (ppm)

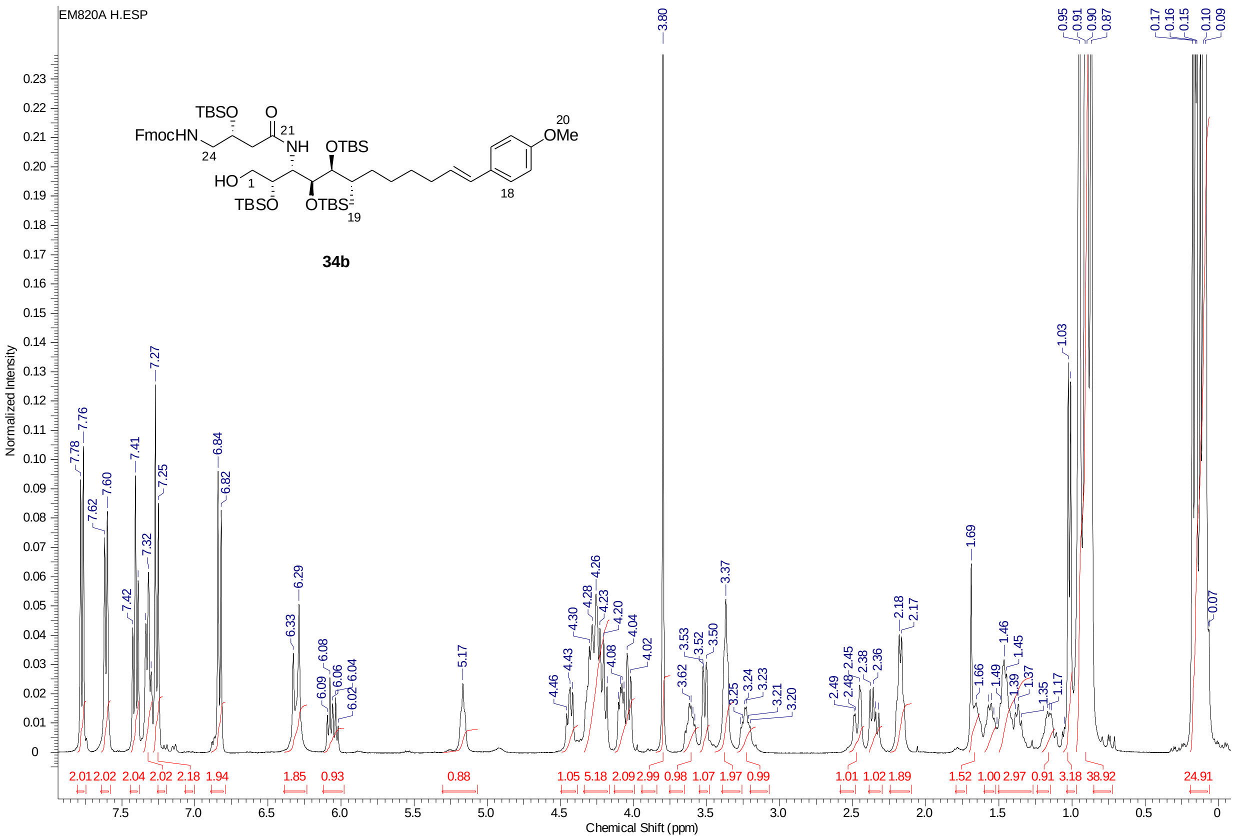
EM820A H.ESP
TBSO
O
21
FmocHN
24
NH
OTBS
20
OMe
HO
1
TBSO
OTBS
19
18
34b
Normalized Intensity
Chemical Shift (ppm)

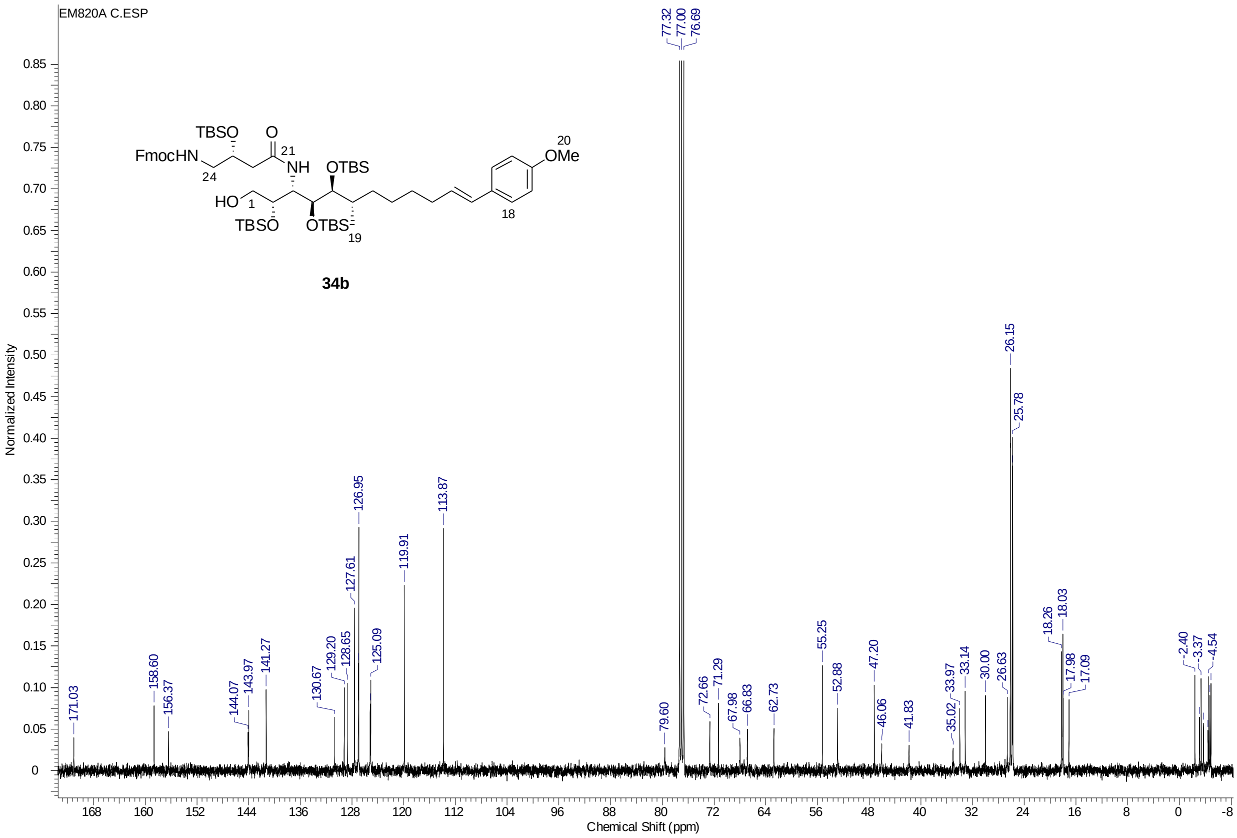
EM820A C.ESP
TBSO
O
21
FmocHN
24
NH
OTBS
20
OMe
HO
1
TBSO
OTBS
19
18
34b
Normalized Intensity
Chemical Shift (ppm)

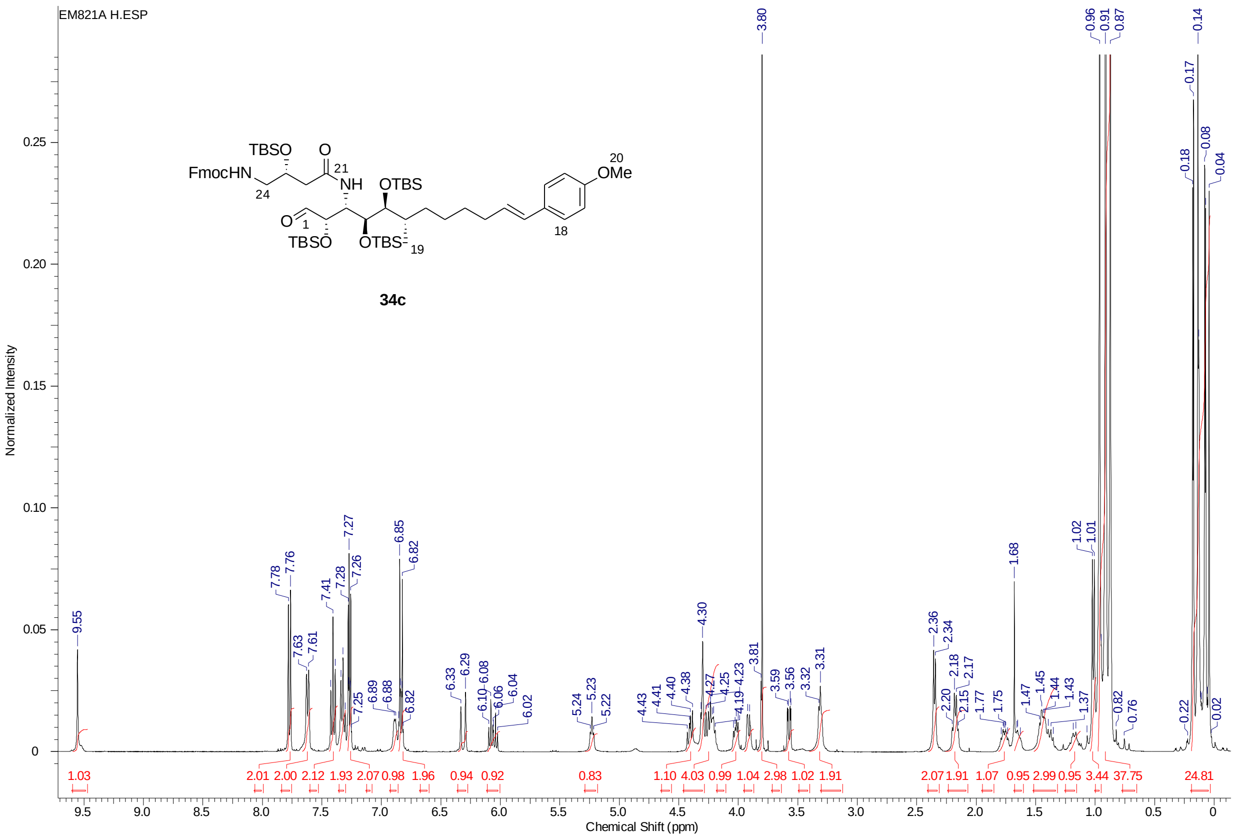
EM821A H.ESP
34c
Normalized Intensity
Chemical Shift (ppm)

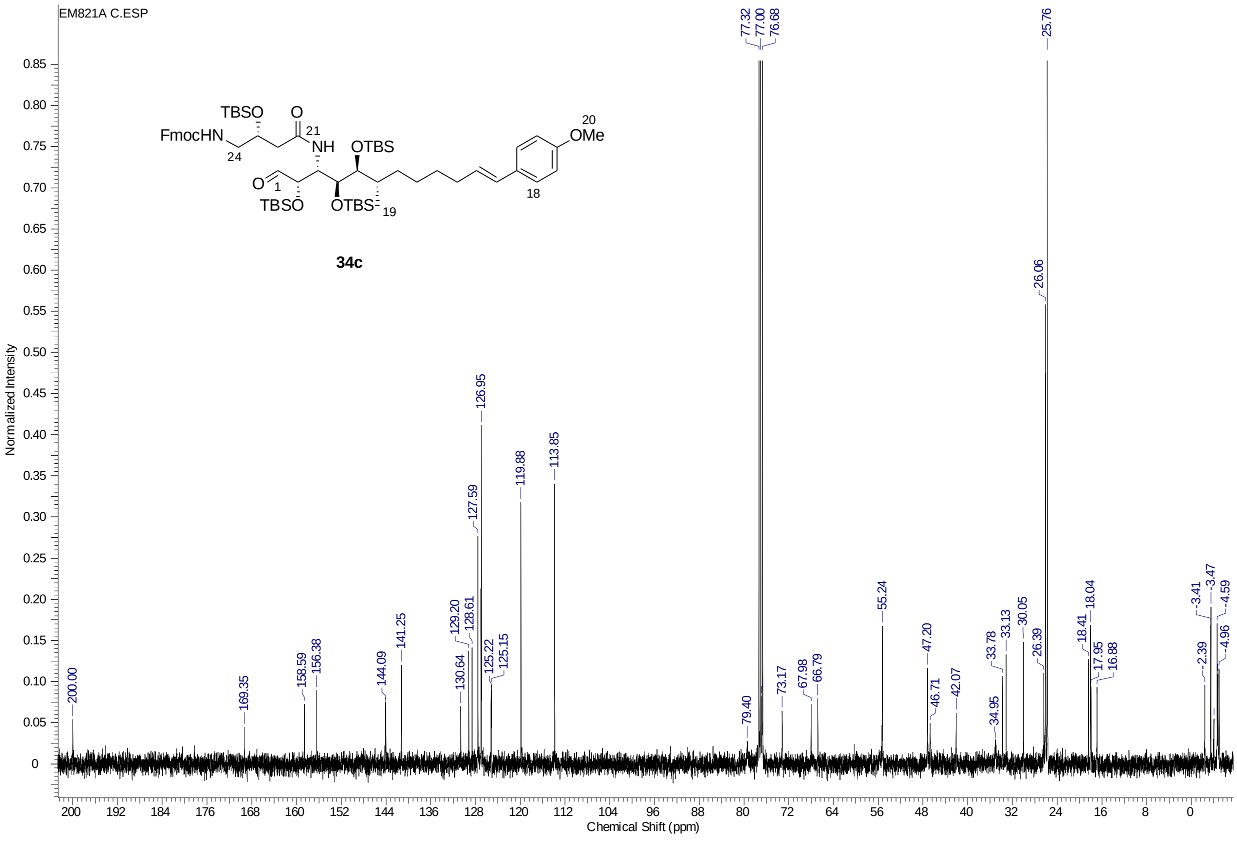
EM821A C.ESP
34c
Normalized Intensity
Chemical Shift (ppm)

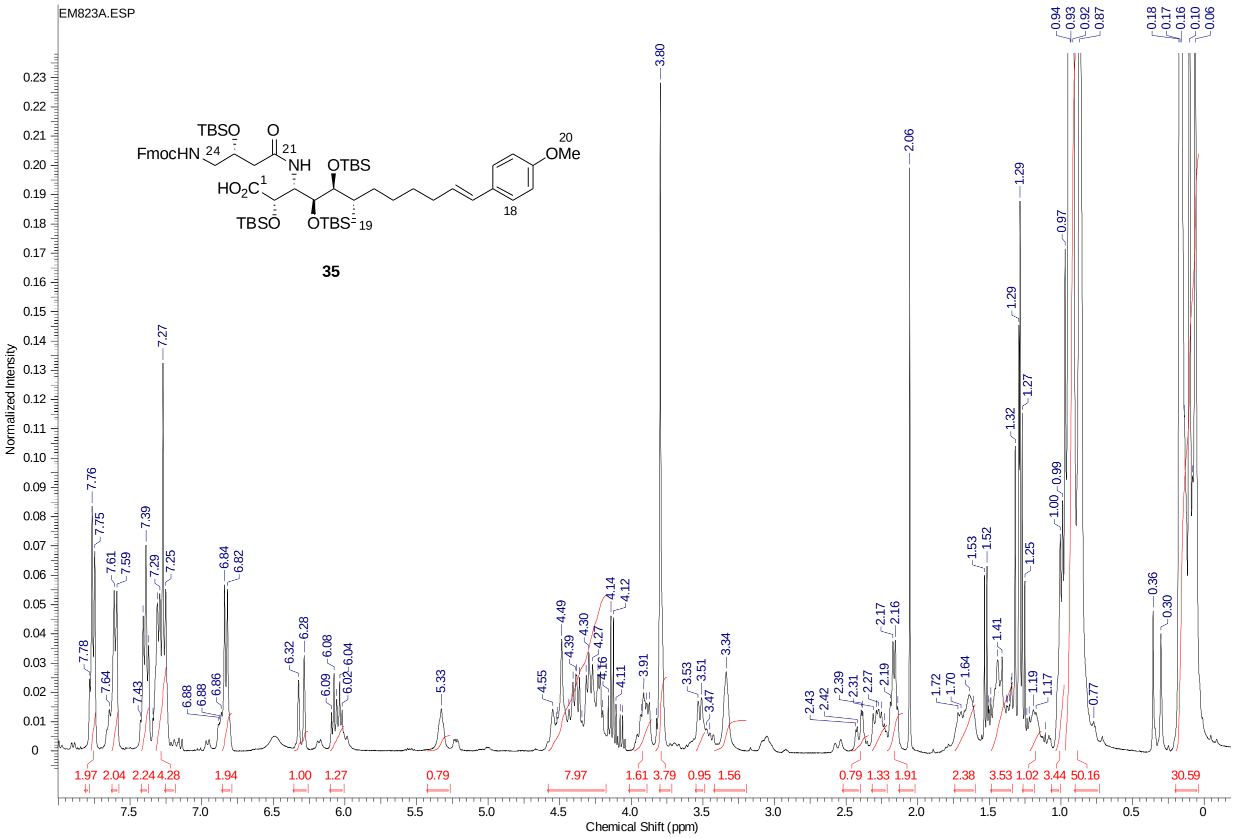
EM823A.ESP
FmocHN 24
TBSO
O
21
NH
OTBS
20
OMe
HO2C 1
TBSO
OTBS 19
18
35
Normalized Intensity
Chemical Shift (ppm)

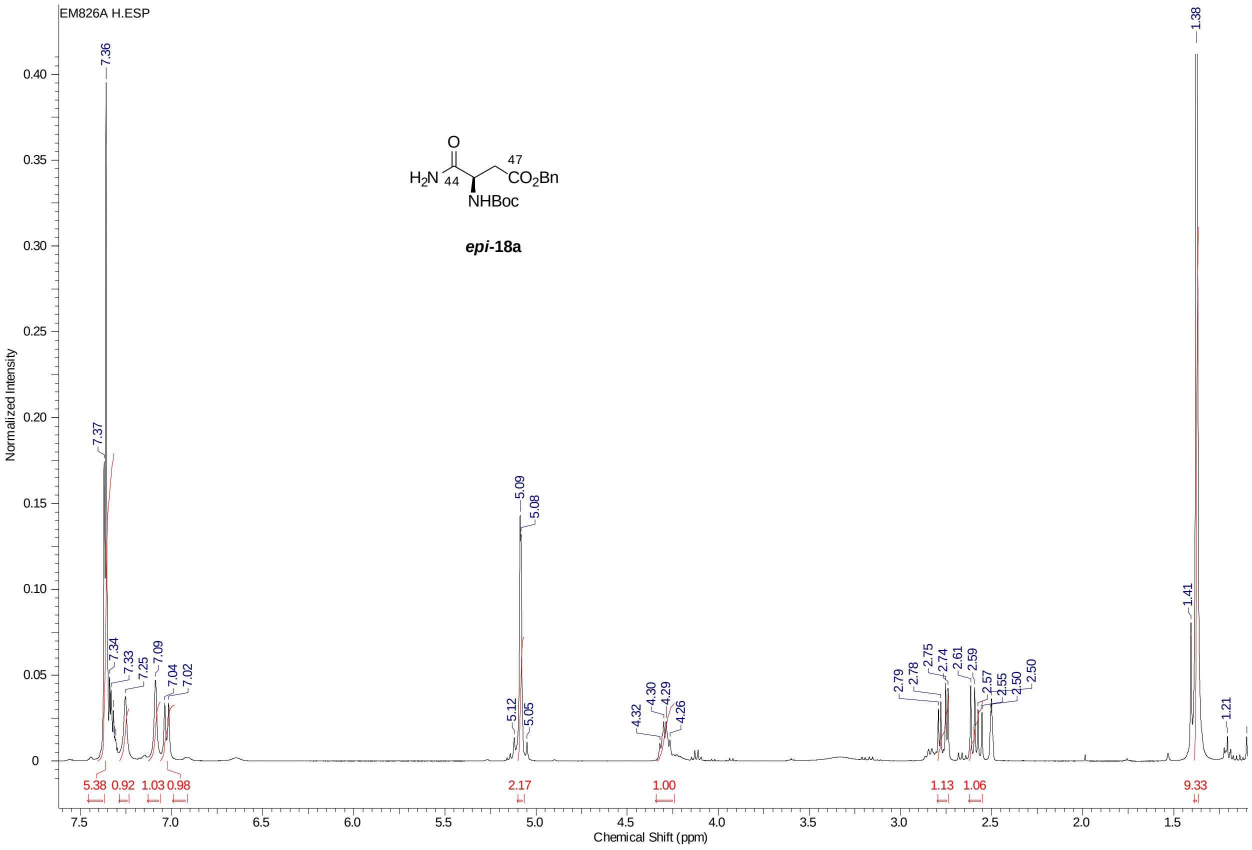
EM826A H.ESP
O
H2N 44
47
CO2Bn
NHBoc
epi-18a
Normalized Intensity
Chemical Shift (ppm)

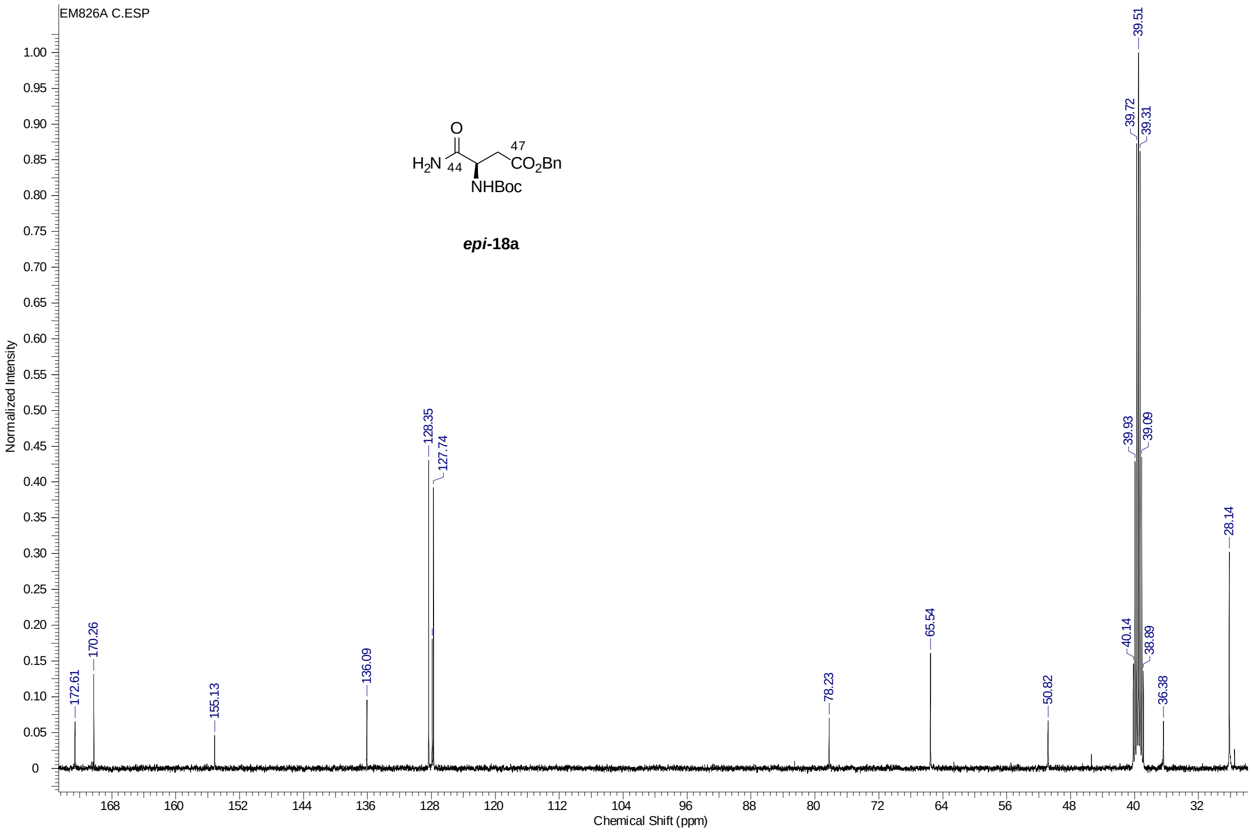
EM826A C.ESP
Normalized Intensity
Chemical Shift (ppm)
H2N
44
47
CO2Bn
NHBoc
epi-18a

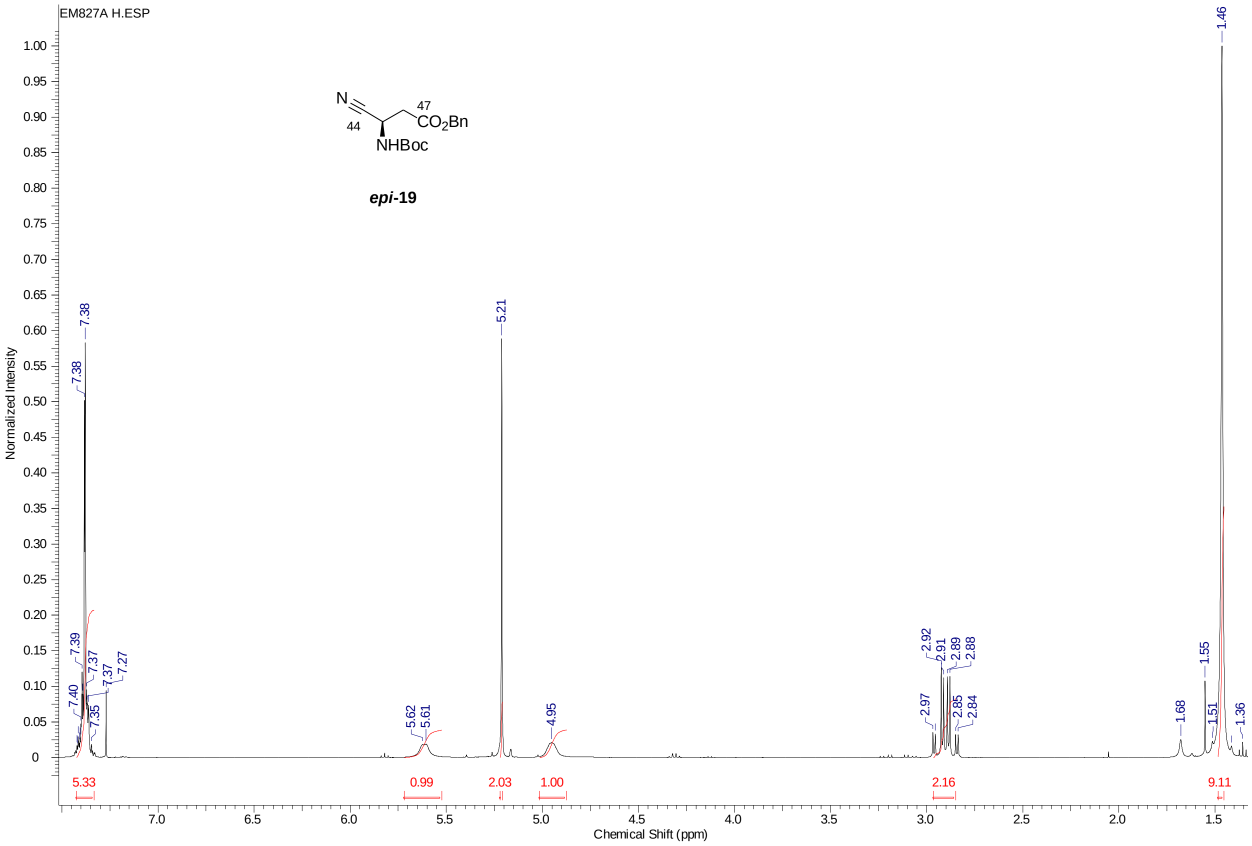
EM827A H.ESP
Normalized Intensity
Chemical Shift (ppm)
N
44
47
CO2Bn
NHBoc
epi-19

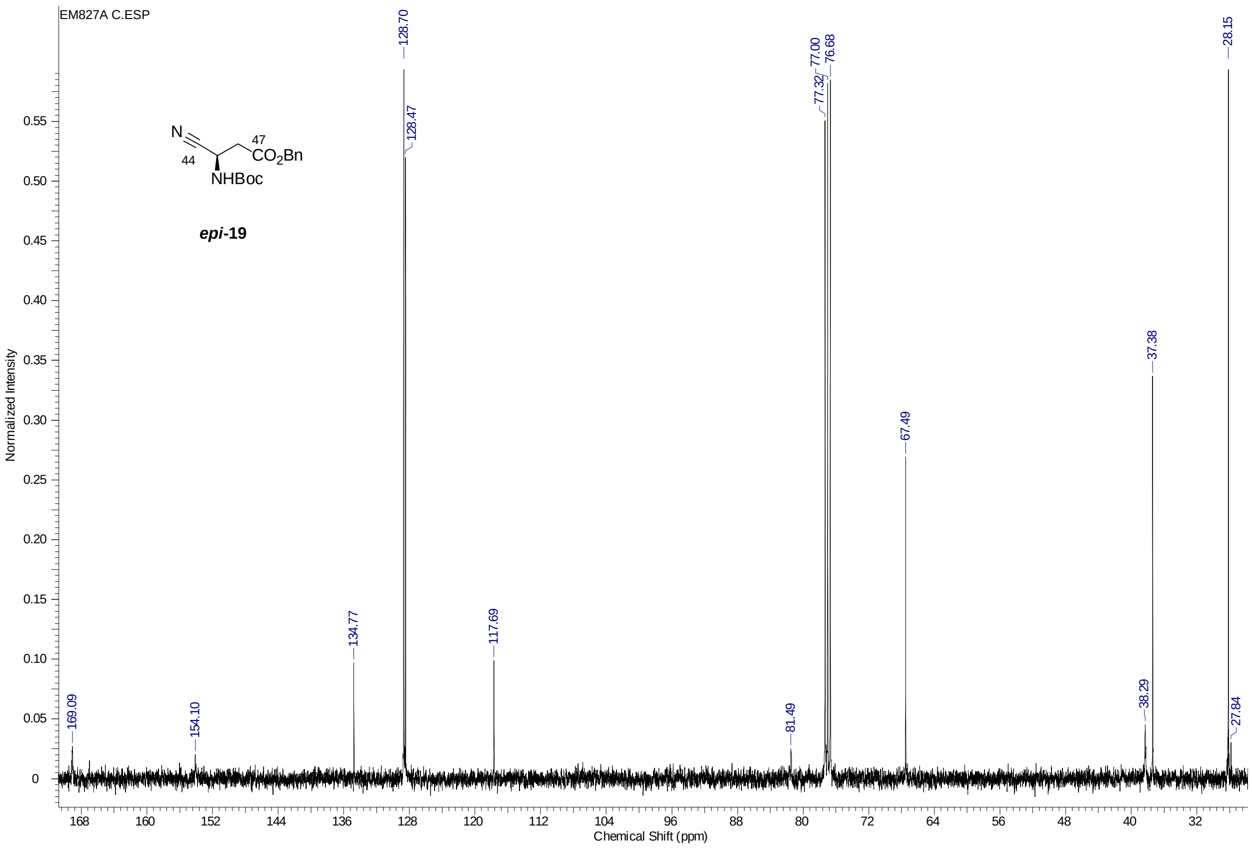
EM827A C.ESP
epi-19
NHBoc
CO_2Bn
44
47
Normalized Intensity
Chemical Shift (ppm)
169.09
154.10
134.77
128.70
128.47
117.69
81.49
77.32
77.00
76.68
67.49
38.29
37.38
28.15
27.84

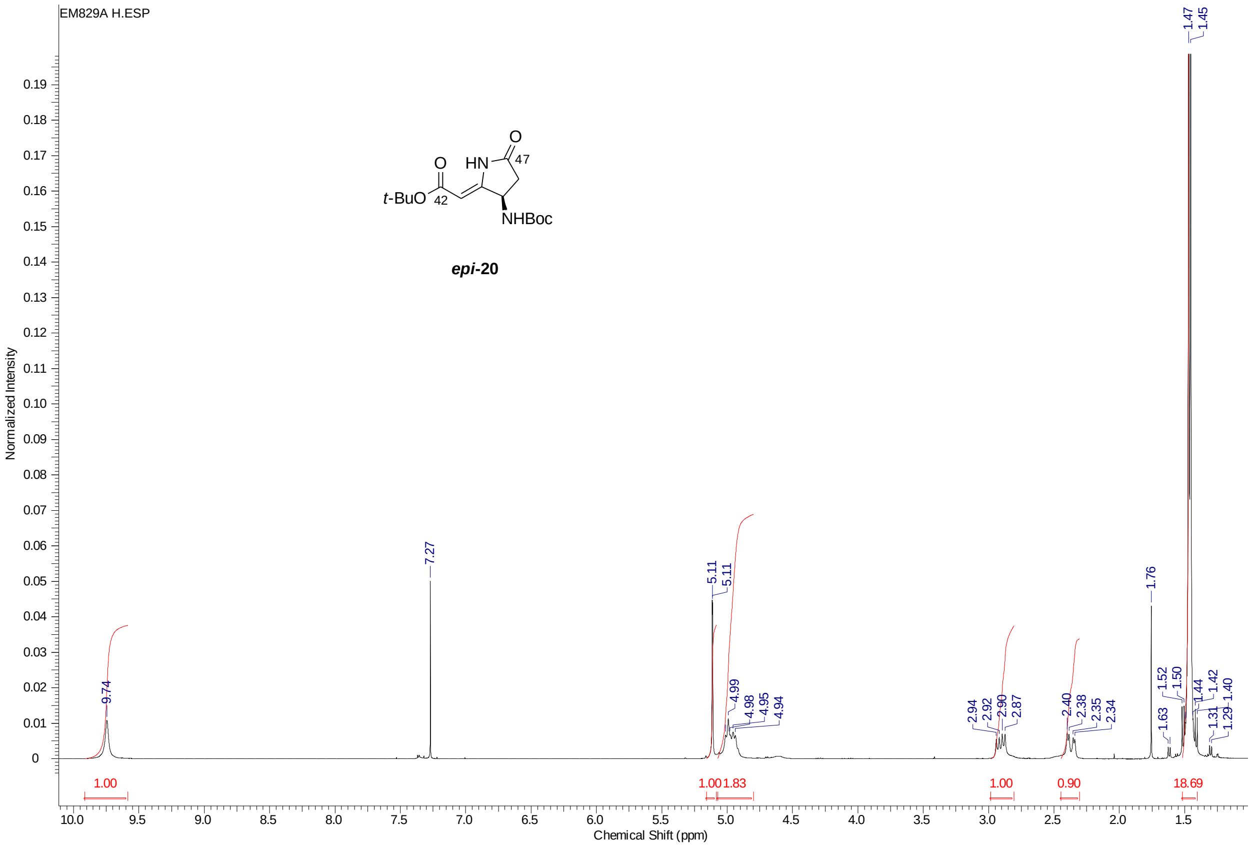
EM829A H.ESP
epi-20
t-BuO
HN
NHBoc
42
47
Normalized Intensity
Chemical Shift (ppm)
9.74
7.27
5.11
5.11
4.99
4.98
4.95
4.94
2.94
2.92
2.90
2.87
2.40
2.38
2.35
2.34
1.76
1.63
1.52
1.50
1.47
1.45
1.44
1.42
1.40
1.31
1.29
1.00
1.00
1.83
1.00
0.90
18.69

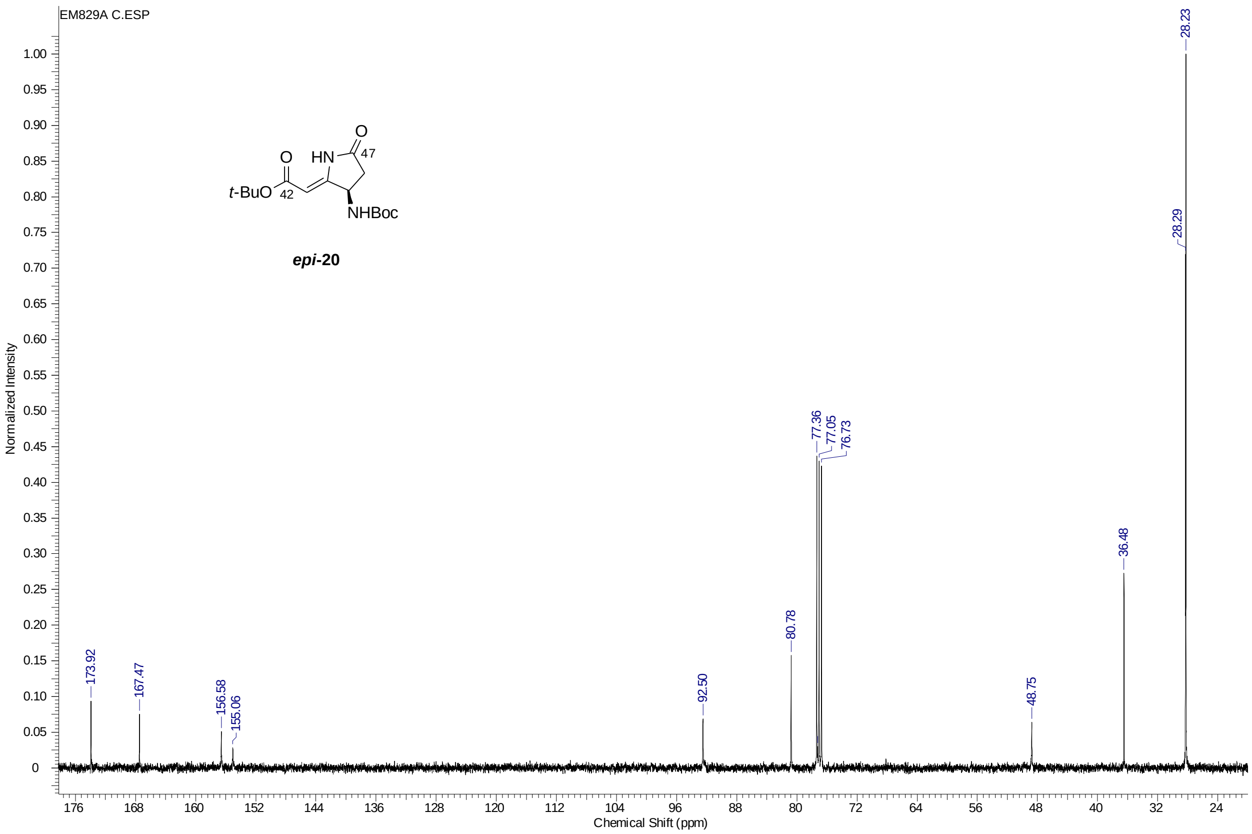

HPLC traces of a racemic sample of 20 (left) and a sample of *epi*-20 obtained from the Blaise reaction on enantiopure starting material *epi*-19 (right)

15 Racemic 10%IPA 1mL (3)

Sample Name:	Racemic 10%IPA 1mL (3)	*Injection Volume:*	20.0
Vial Number:	14	*Channel:*	UV_VIS_1
Sample Type:	unknown	*Wavelength:*	254
Control Program:	IPA 10% 1mL	*Bandwidth:*	n.a.
Quantif. Method:	Trial	*Dilution Factor:*	1.0000
Recording Time:	6/5/2015 16:42	*Sample Weight:*	1.0000
Run Time (min):	49.38	*Sample Amount:*	1.0000

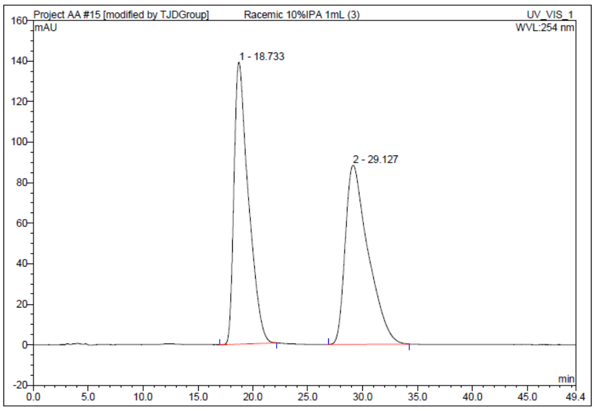

No.	Ret.Time min	Peak Name	Height mAU	Area mAU*min	Rel.Area %	Amount n.a.	Type
1	18.73	n.a.	139.213	212.016	49.81	n.a.	BMB*
2	29.13	n.a.	88.504	213.603	50.19	n.a.	BMB*
Total:			227.717	425.619	100.00	0.000	

13 Sample 10%IPA 1mL (2)

Sample Name:	Sample 10%IPA 1mL (2)	*Injection Volume:*	20.0
Vial Number:	12	*Channel:*	UV_VIS_1
Sample Type:	unknown	*Wavelength:*	254
Control Program:	IPA 10% 1mL	*Bandwidth:*	n.a.
Quantif. Method:	Trial	*Dilution Factor:*	1.0000
Recording Time:	6/5/2015 15:19	*Sample Weight:*	1.0000
Run Time (min):	39.99	*Sample Amount:*	1.0000

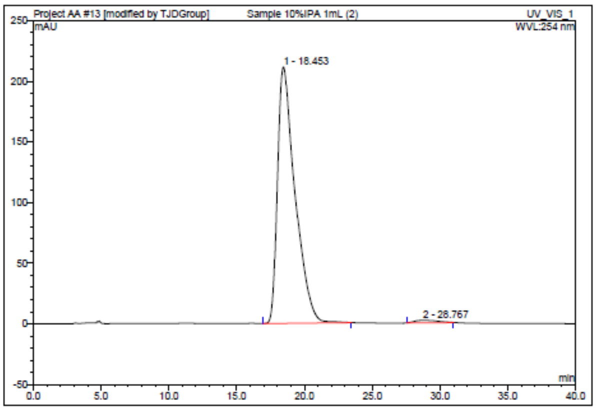

No.	Ret.Time min	Peak Name	Height mAU	Area mAU*min	Rel.Area %	Amount n.a.	Type
1	18.45	n.a.	211.405	316.697	98.77	n.a.	BMB*
2	28.77	n.a.	2.241	3.947	1.23	n.a.	BMB*
Total:			213.645	320.644	100.00	0.000	

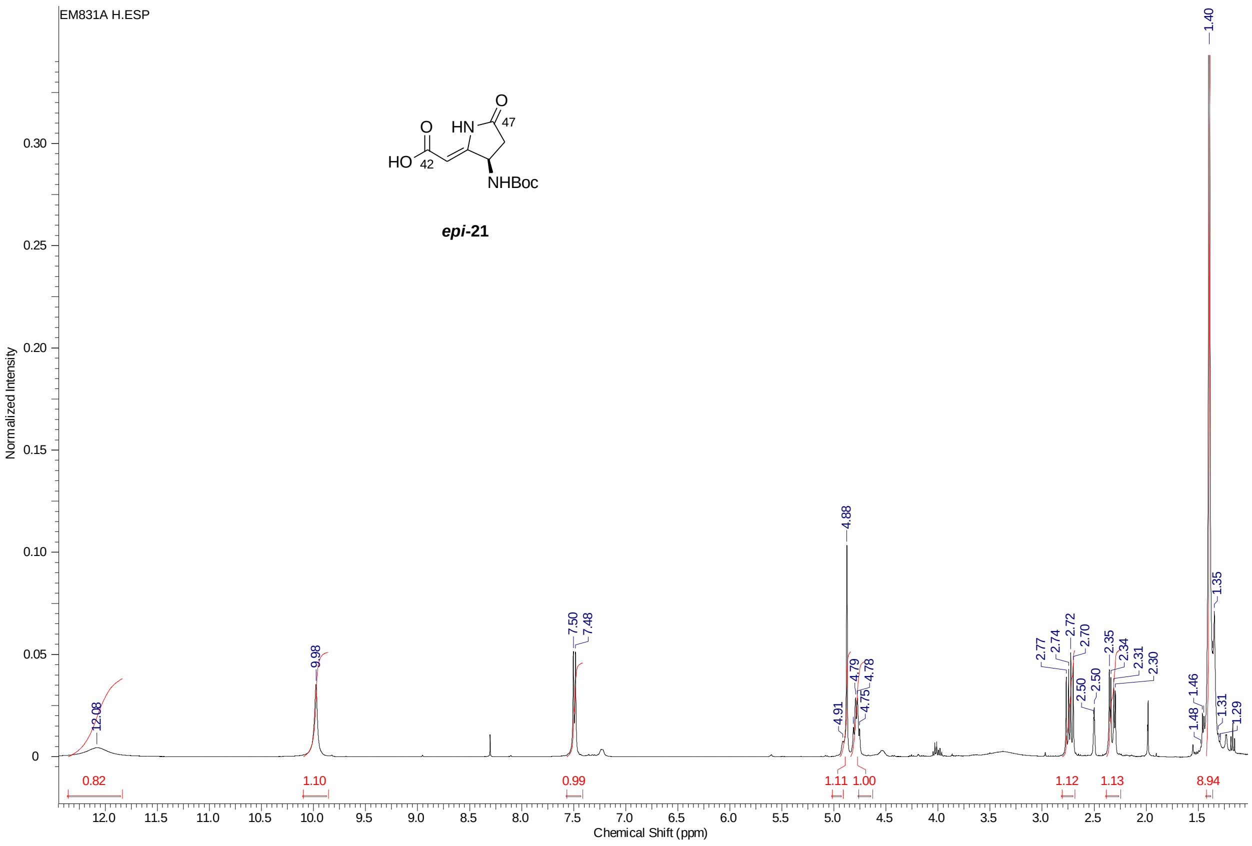
EM831A H.ESP
epi-21
Normalized Intensity
Chemical Shift (ppm)
12.08
9.98
7.50
7.48
4.91
4.88
4.79
4.78
4.75
2.77
2.74
2.72
2.70
2.50
2.50
2.35
2.34
2.31
2.30
1.48
1.46
1.40
1.35
1.31
1.29
0.82
1.10
0.99
1.11
1.00
1.12
1.13
8.94

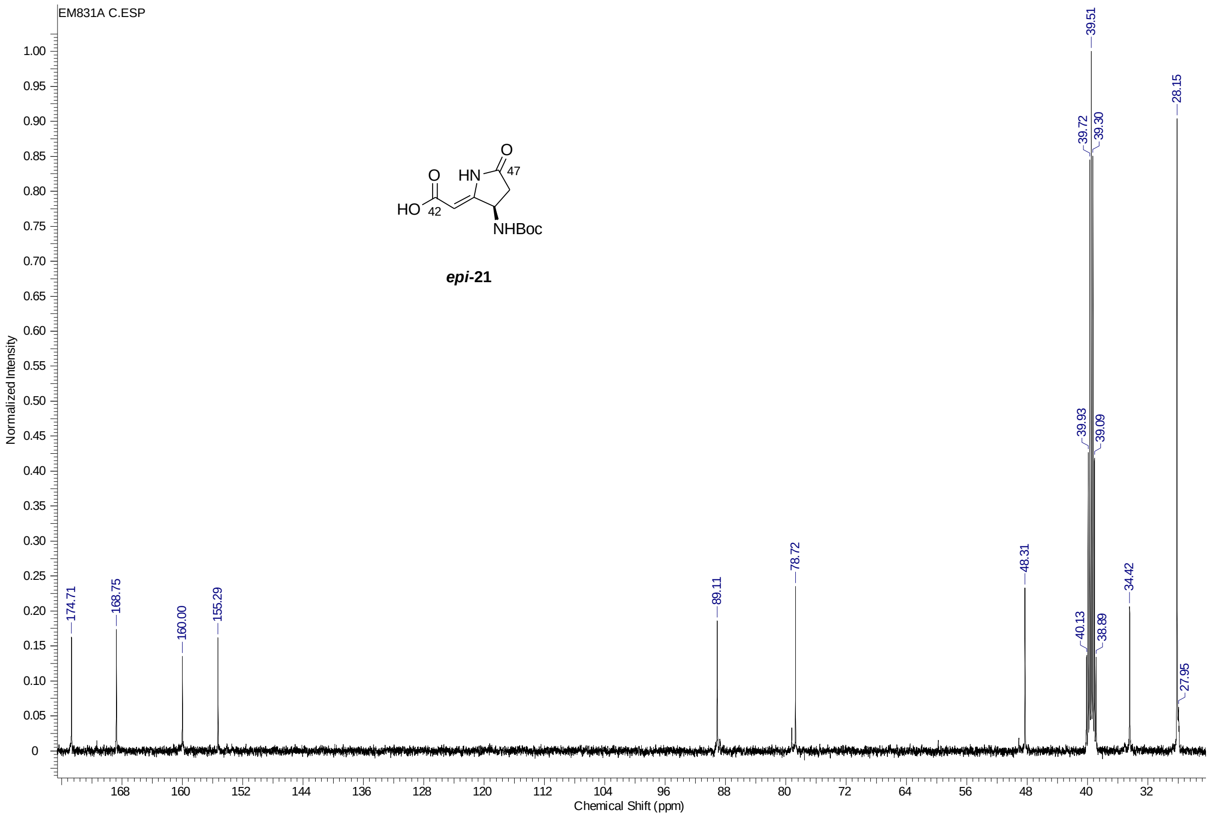
EM831A C.ESP
epi-21
Normalized Intensity
Chemical Shift (ppm)
174.71
168.75
160.00
155.29
89.11
78.72
48.31
40.13
39.93
39.72
39.51
39.30
39.09
38.89
34.42
28.15
27.95

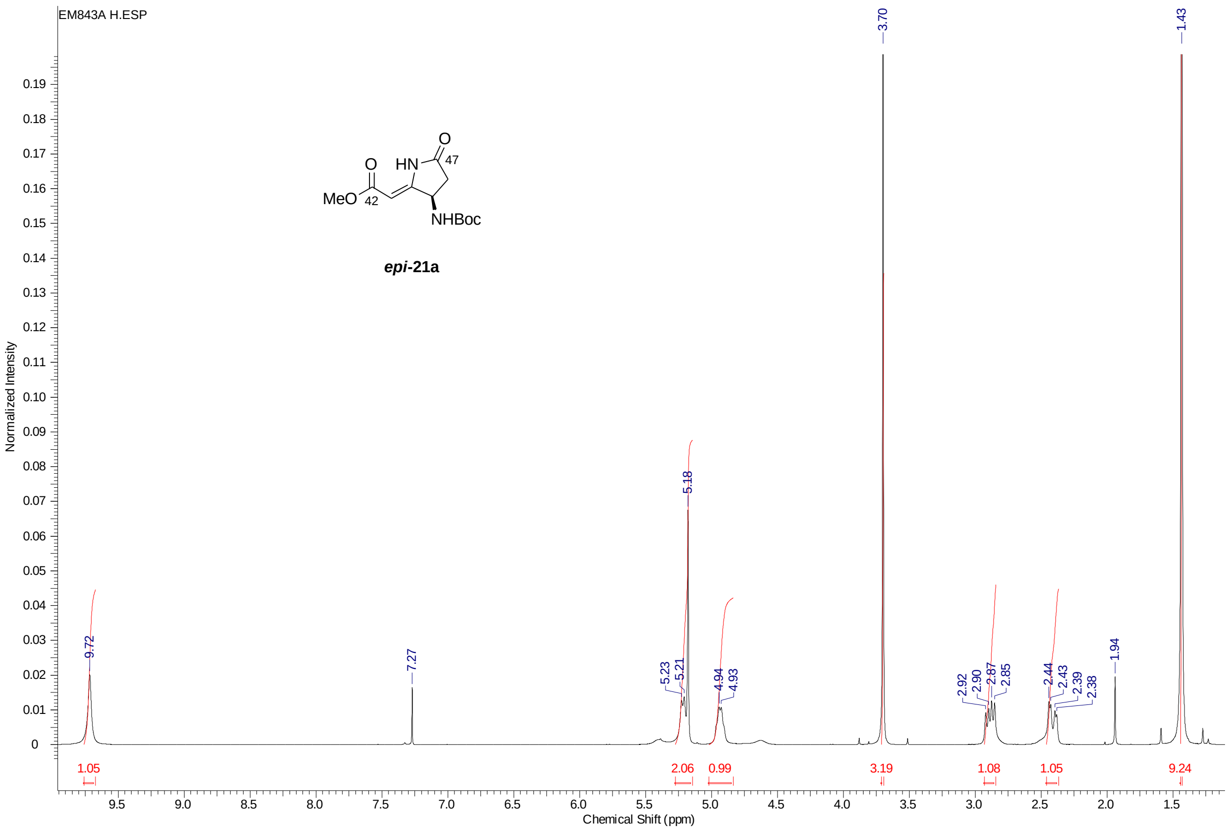

HPLC traces of a racemic sample of 21a (left) and a sample of *epi*-21a obtained from enantiopure pyrrolidinone acid *epi*-21 (right)

19 racemic Me ester

Sample Name:	racemic Me ester	*Injection Volume:*	20.0
Vial Number:	18	*Channel:*	UV_VIS_1
Sample Type:	unknown	*Wavelength:*	254
Control Program:	ipa 20%	*Bandwidth:*	n.a.
Quantif. Method:	Trial	*Dilution Factor:*	1.0000
Recording Time:	4/6/2015 16:45	*Sample Weight:*	1.0000
Run Time (min):	45.00	*Sample Amount:*	1.0000

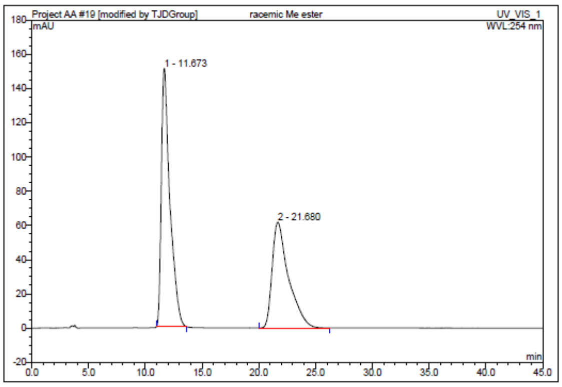

No.	Ret.Time min	Peak Name	Height mAU	Area mAU*min	Rel.Area %	Amount n.a.	Type
1	11.67	n.a.	150.810	131.647	56.01	n.a.	BMB*
2	21.68	n.a.	61.768	103.399	43.99	n.a.	BMB*
Total:			212.578	235.046	100.00	0.000	

18 em843

Sample Name:	em843	*Injection Volume:*	20.0
Vial Number:	17	*Channel:*	UV_VIS_1
Sample Type:	unknown	*Wavelength:*	254
Control Program:	ipa 20%	*Bandwidth:*	n.a.
Quantif. Method:	Trial	*Dilution Factor:*	1.0000
Recording Time:	4/6/2015 15:54	*Sample Weight:*	1.0000
Run Time (min):	45.00	*Sample Amount:*	1.0000

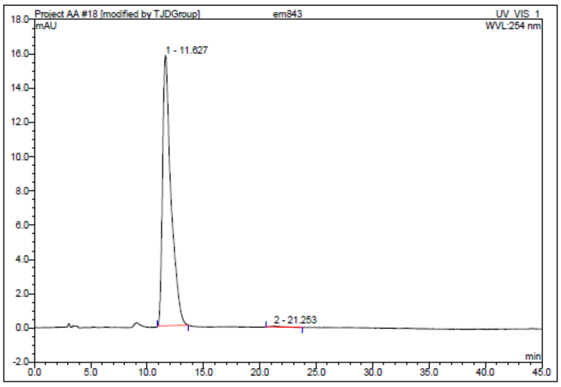

No.	Ret.Time min	Peak Name	Height mAU	Area mAU*min	Rel.Area %	Amount n.a.	Type
1	11.63	n.a.	15.796	13.709	99.51	n.a.	BMB*
2	21.25	n.a.	0.051	0.068	0.49	n.a.	BMB*
Total:			15.848	13.777	100.00	0.000	

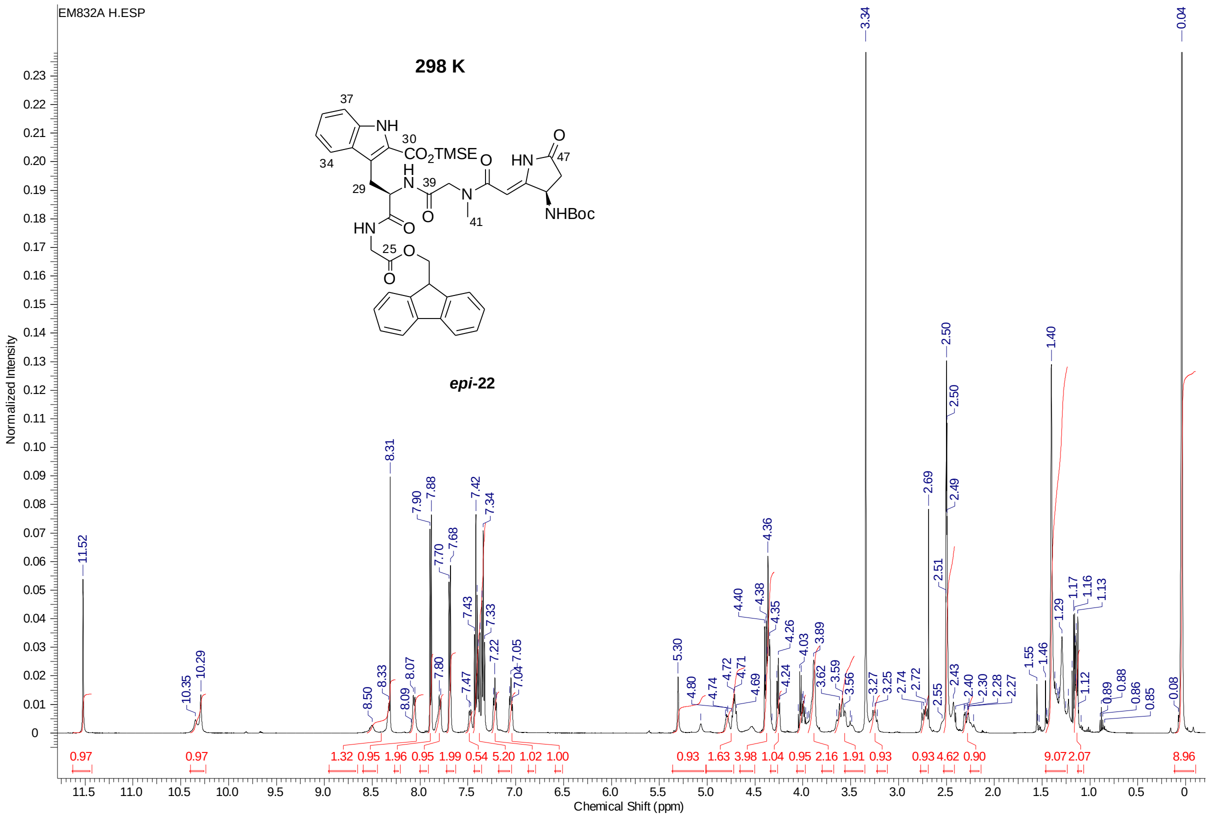
EM832A H.ESP
298 K
epi-22
Normalized Intensity
Chemical Shift (ppm)

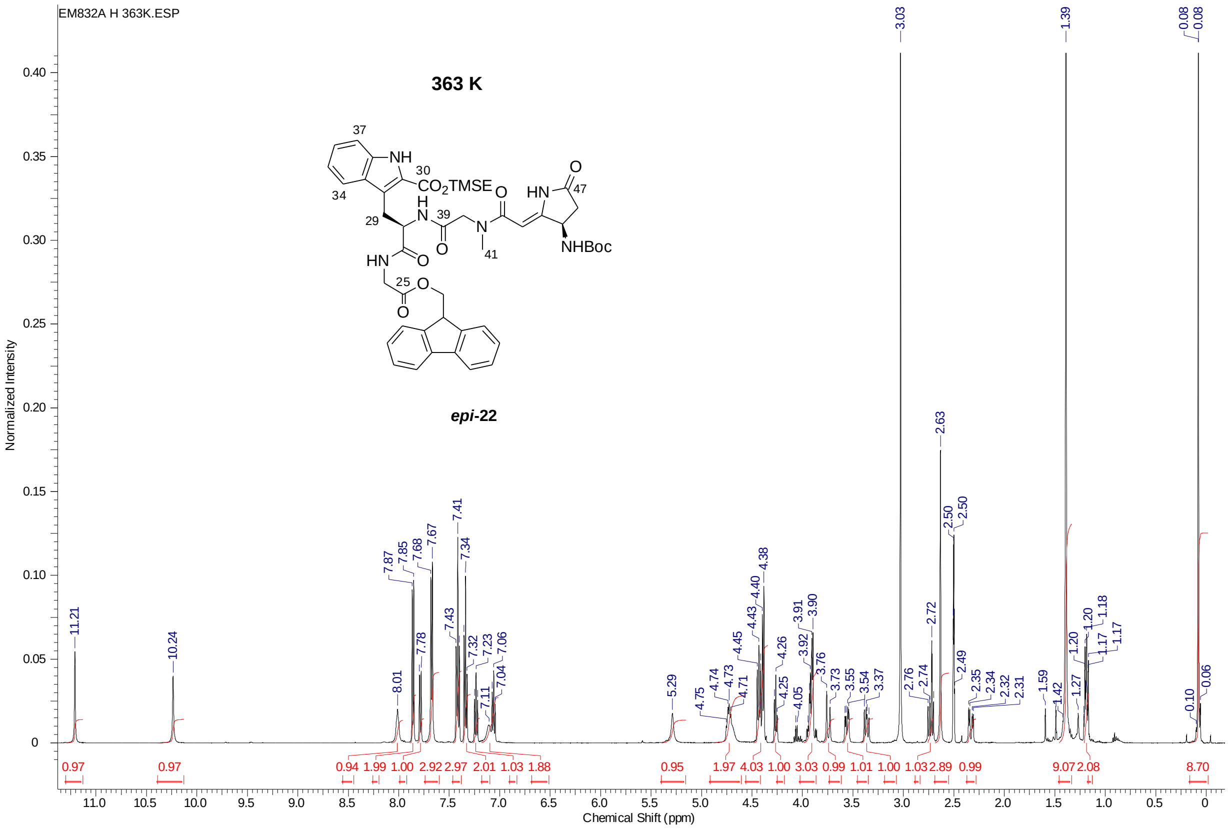
EM832A H 363K.ESP
363 K
epi-22
Normalized Intensity
Chemical Shift (ppm)

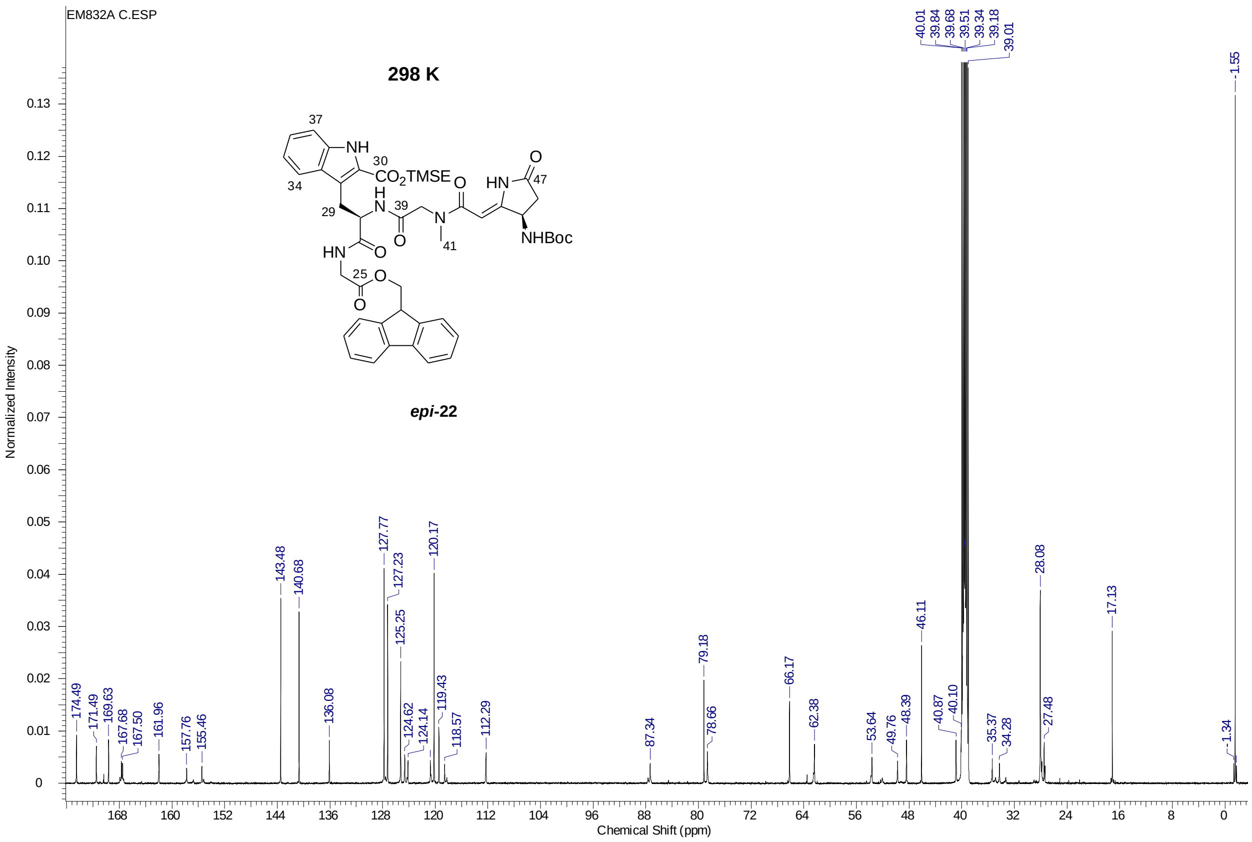
EM832A C.ESP
298 K
epi-22
Normalized Intensity
Chemical Shift (ppm)

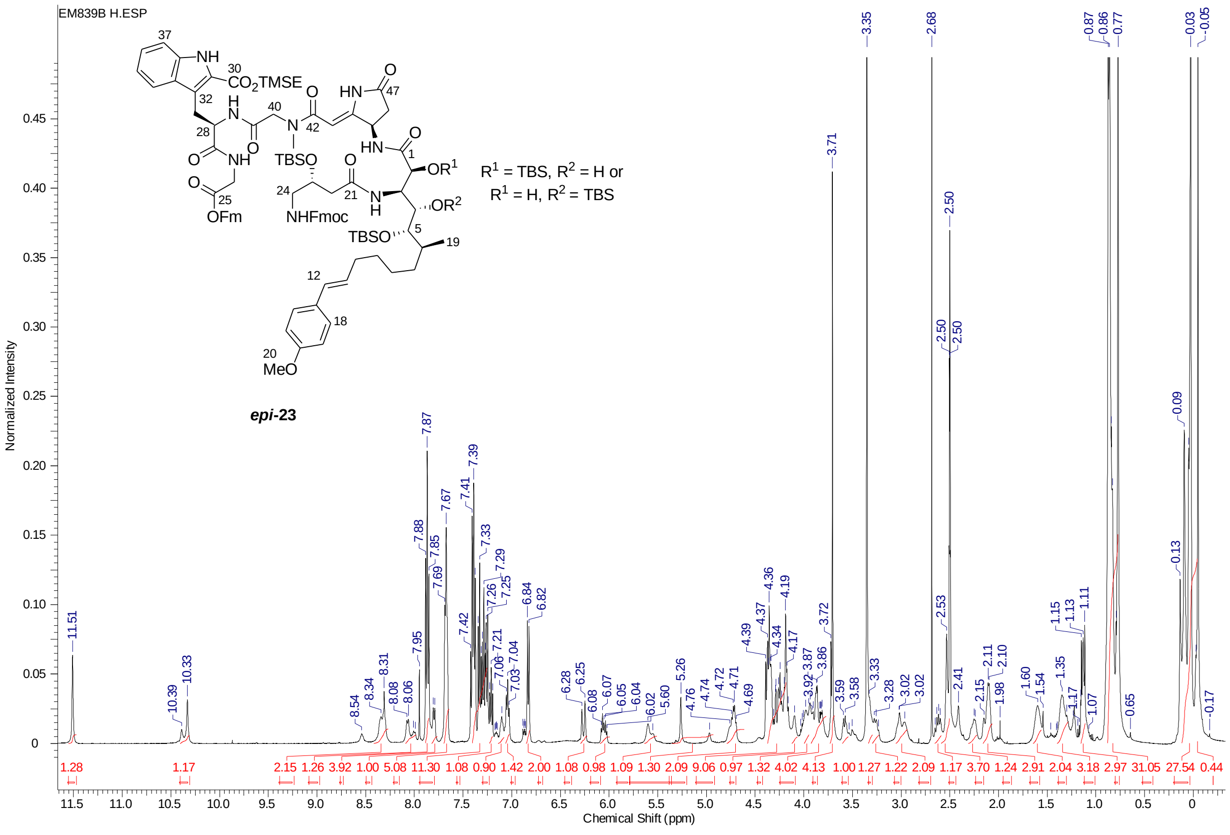
EM839B H.ESP
R1 = TBS, R2 = H or
R1 = H, R2 = TBS
epi-23
Normalized Intensity
Chemical Shift (ppm)

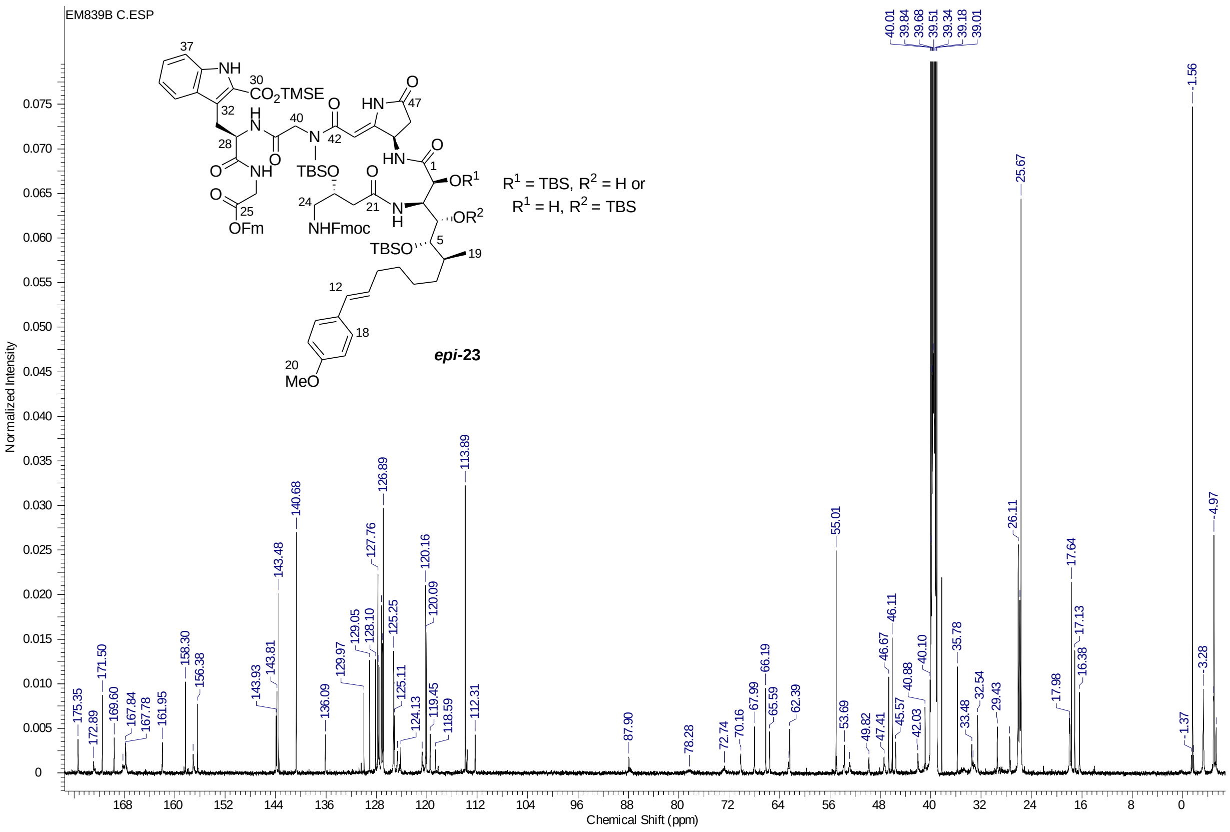
EM839B C.ESP
R1 = TBS, R2 = H or
R1 = H, R2 = TBS
MeO
TBSO
NHFmoc
OFm
CO2TMSE
epi-23
Normalized Intensity
Chemical Shift (ppm)
40.01 39.84 39.68 39.51 39.34 39.18 39.01
1.56
25.67
26.11
55.01
140.68
126.89
113.89
127.76
143.48
120.16
120.09
171.50
158.30
156.38
129.05 128.10
125.25
129.97
125.11
143.93 143.81
175.35 172.89 169.60 167.84 167.78 161.95
136.09
124.13
119.45 118.59
112.31
87.90
78.28
72.74 70.16 67.99 66.19 65.59
62.39
53.69
49.82 47.41 46.67 46.11 45.57 40.88 42.03 40.10
35.78 33.48 32.54 29.43
17.98 17.64 17.13 16.38
1.37 -3.28 -4.97

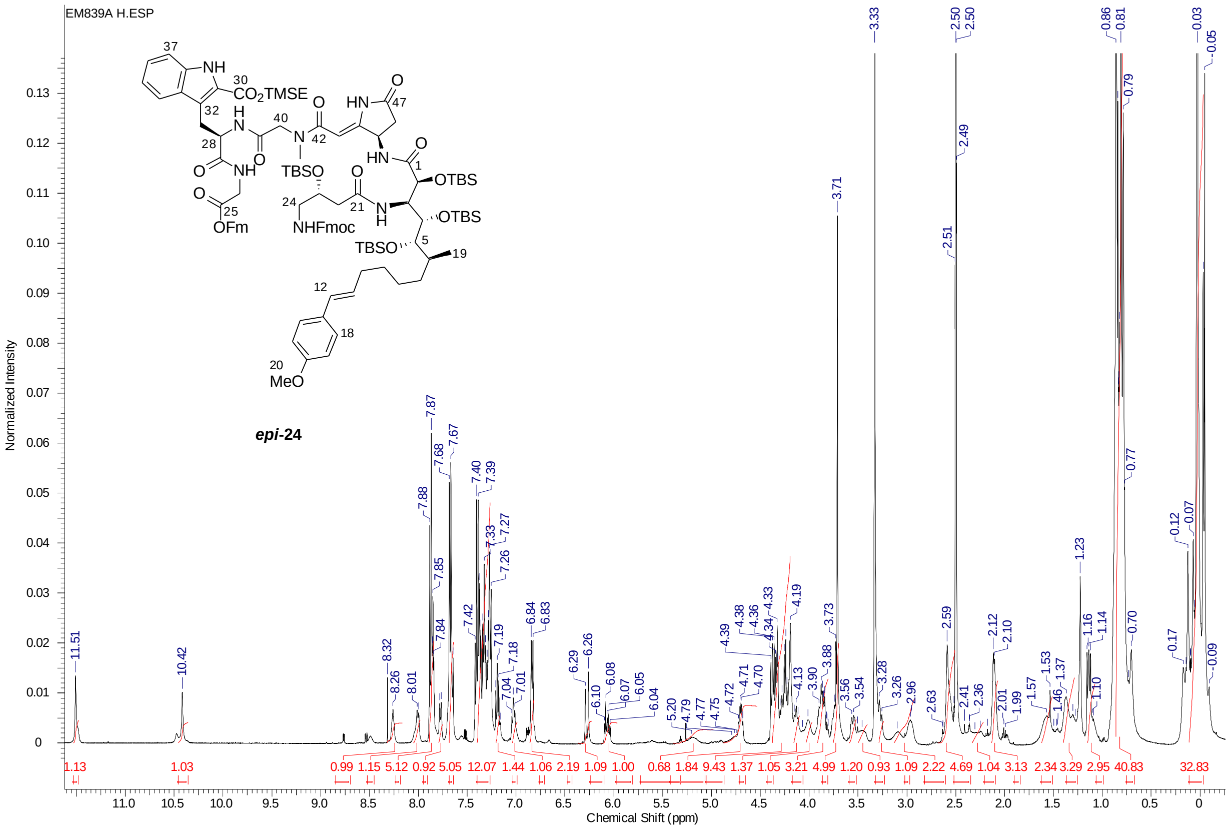
EM839A H.ESP
OTBS
OTBS
TBSO
MeO
NHFmoc
OFm
CO2TMSE
epi-24
Normalized Intensity
Chemical Shift (ppm)

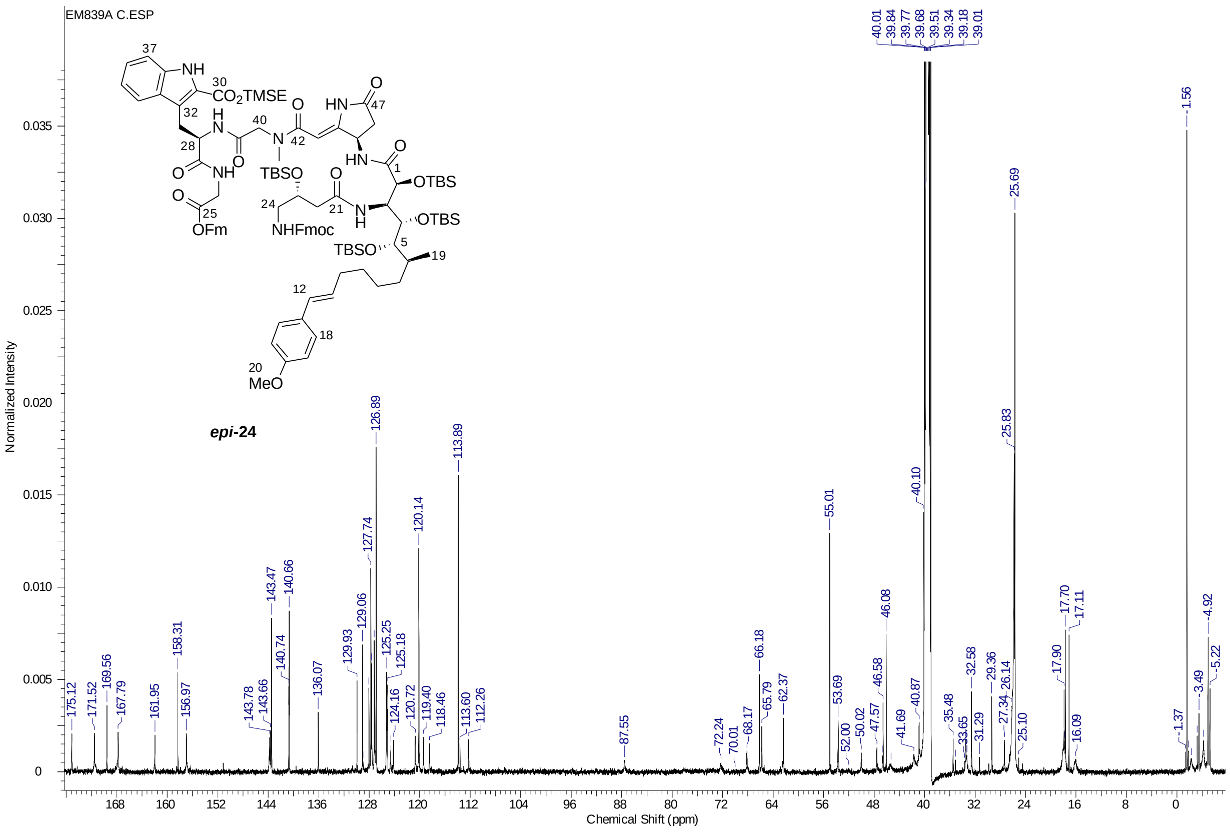
EM839A C.ESP
Normalized Intensity
Chemical Shift (ppm)
epi-24
CO2TMSE
NHFmoc
OFm
TBSO
OTBS
MeO

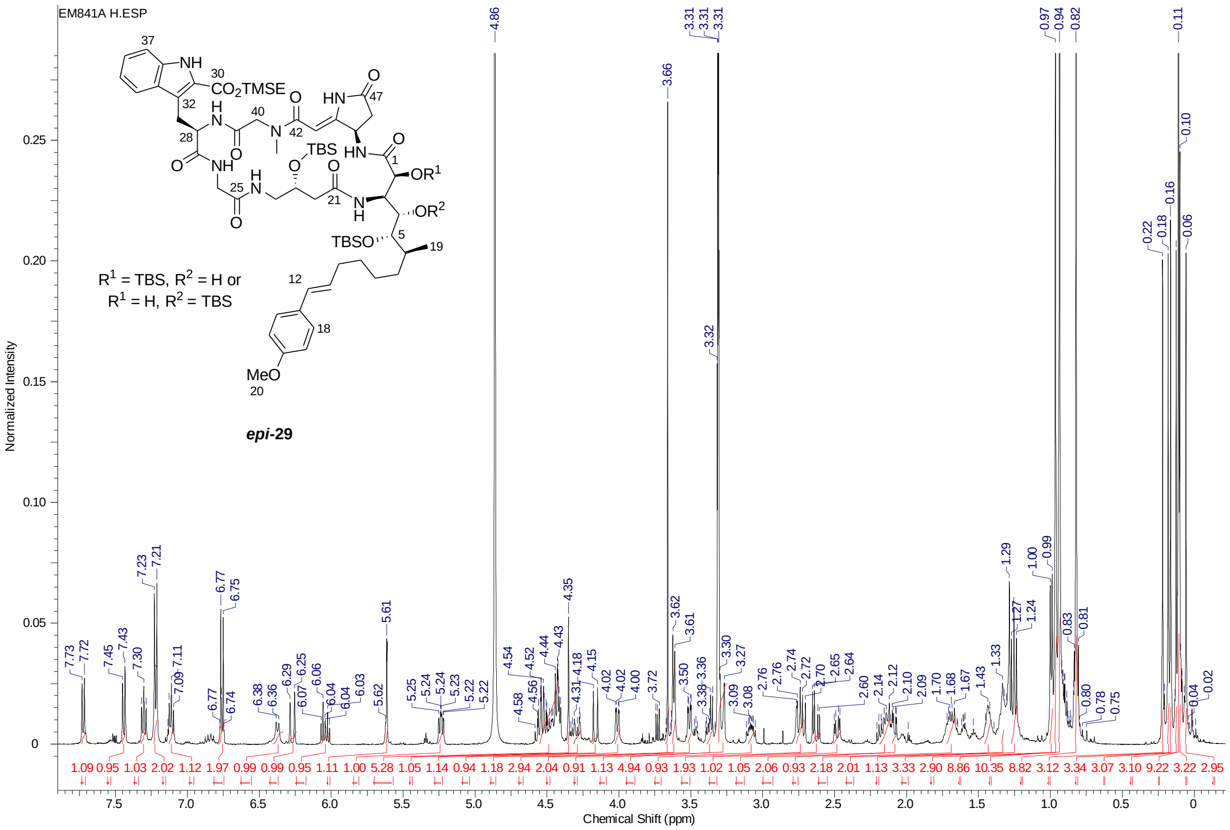
EM841A H.ESP
Normalized Intensity
Chemical Shift (ppm)
epi-29
R1 = TBS, R2 = H or
R1 = H, R2 = TBS
CO2TMSE
TBSO
MeO

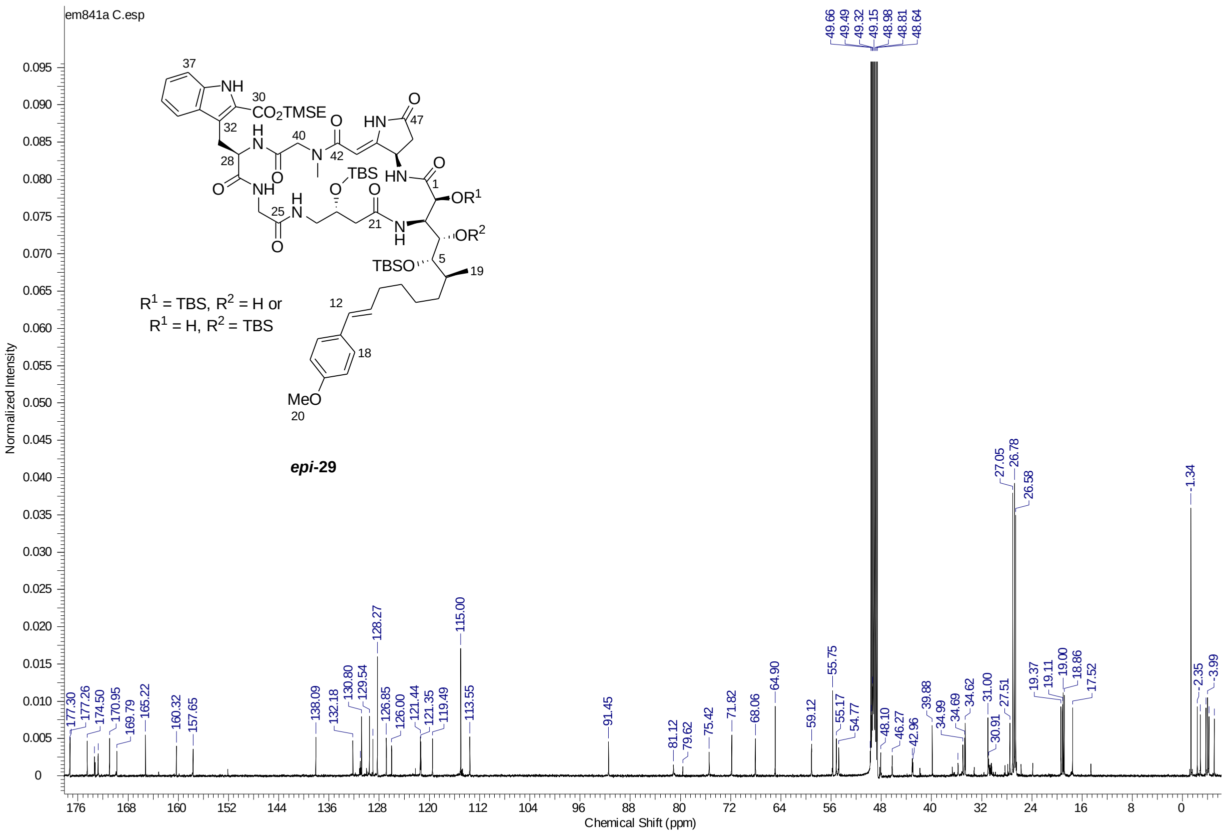
em841a C.esp
epi-29
R1 = TBS, R2 = H or
R1 = H, R2 = TBS
Normalized Intensity
Chemical Shift (ppm)

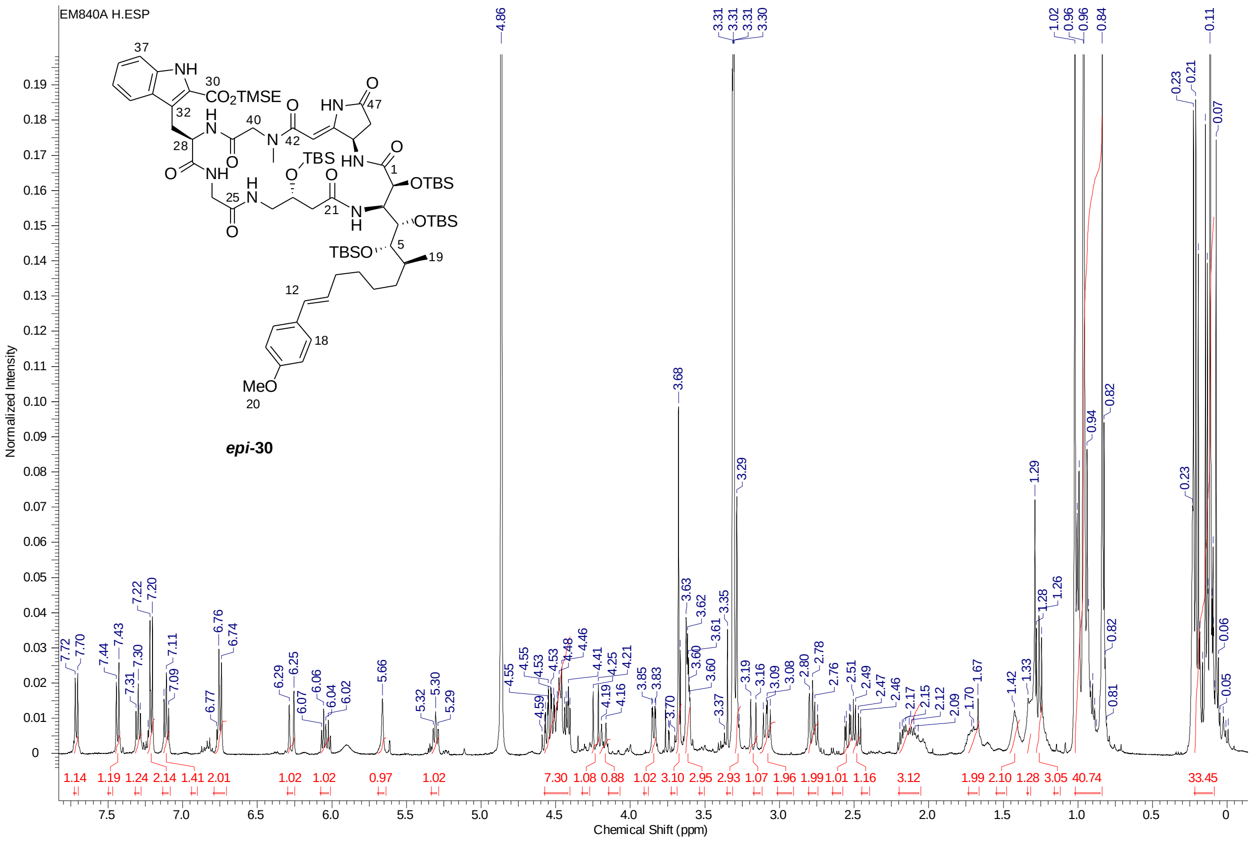
EM840A H.ESP
epi-30
Normalized Intensity
Chemical Shift (ppm)

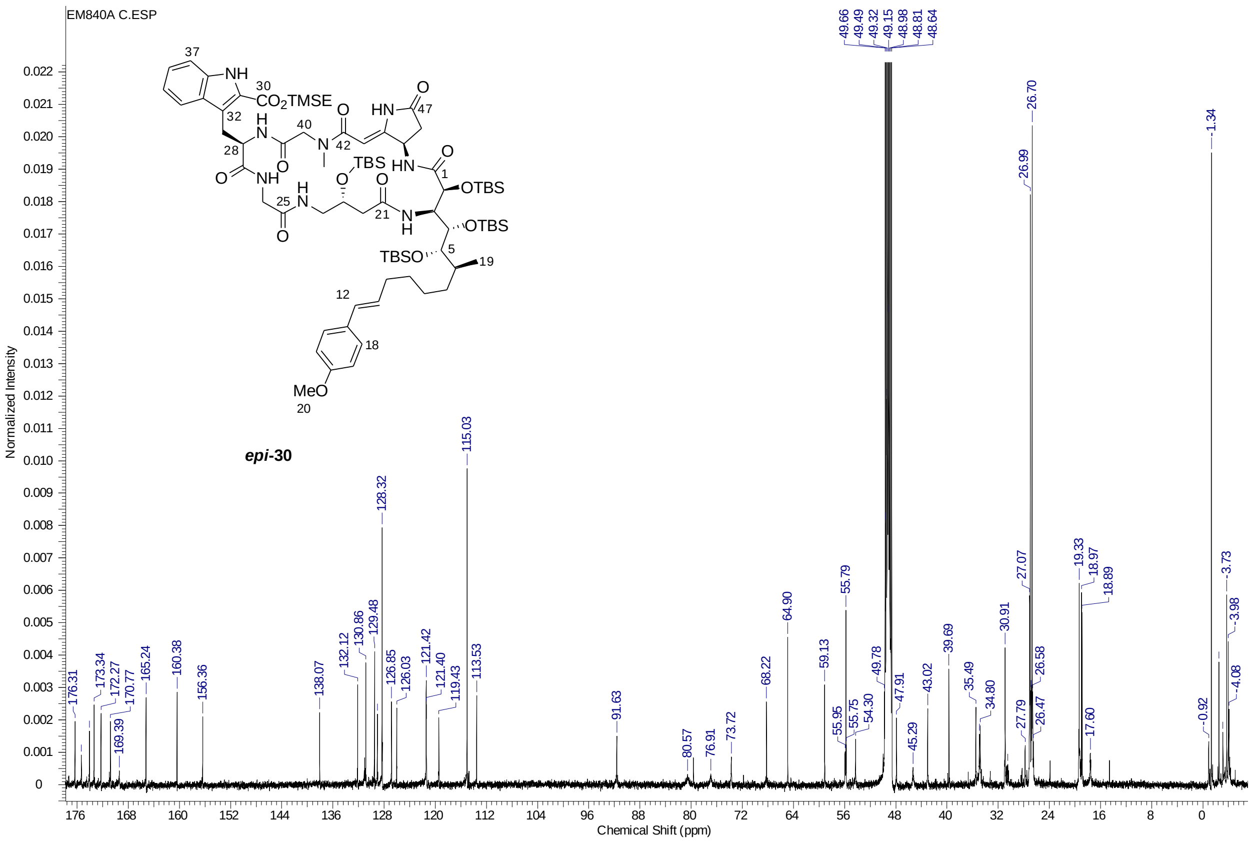
EM840A C.ESP
epi-30
Normalized Intensity
Chemical Shift (ppm)
CO2TMSE
OTBS
TBSO
MeO
176.31
173.34
172.27
170.77
169.39
165.24
160.38
156.36
138.07
132.12
130.86
129.48
128.32
126.85
126.03
121.42
121.40
119.43
115.03
113.53
91.63
80.57
76.91
73.72
68.22
64.90
59.13
55.95
55.79
55.75
54.30
49.78
49.66
49.49
49.32
49.15
48.98
48.81
48.64
47.91
45.29
43.02
39.69
35.49
34.80
30.91
27.79
27.07
26.99
26.70
26.58
26.47
19.33
18.97
18.89
17.60
-0.92
-1.34
-3.73
-3.98
-4.08

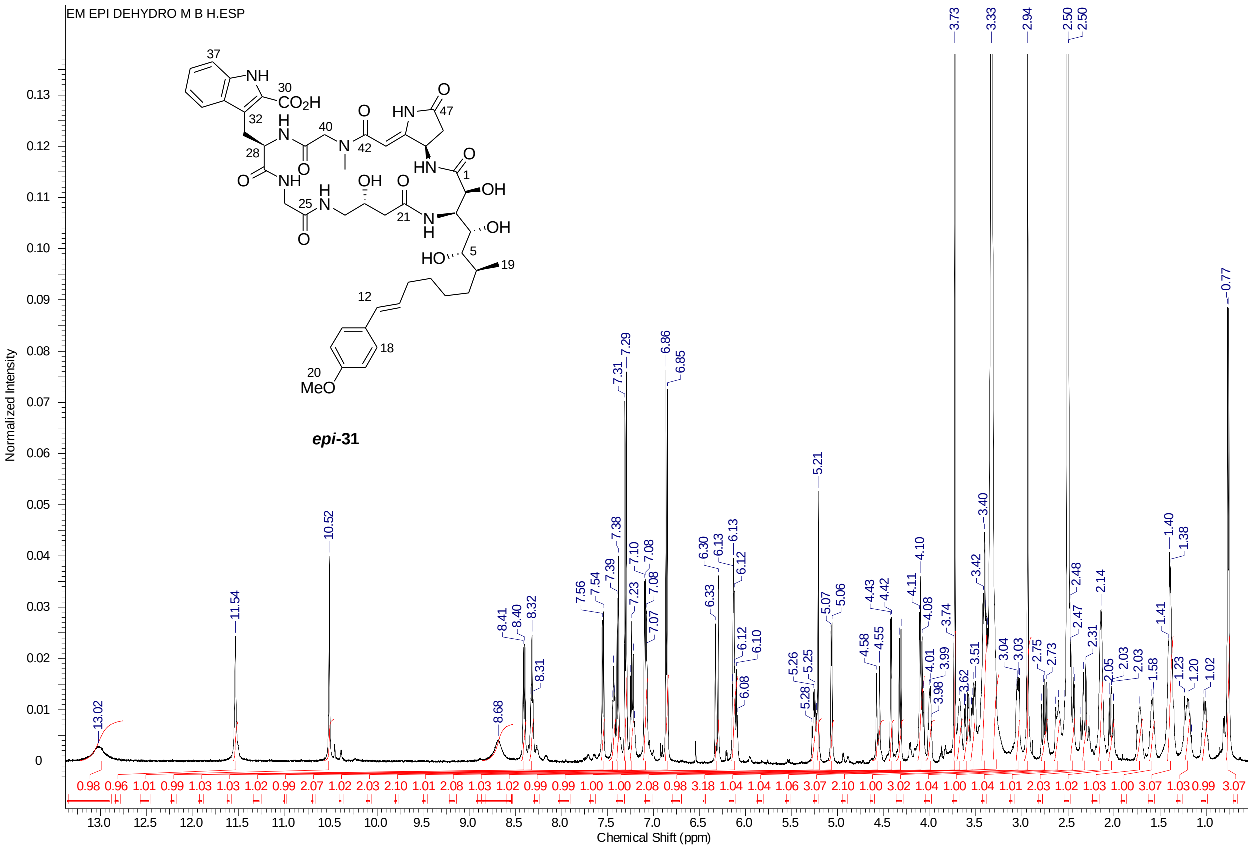
EM EPI DEHYDRO M B H.ESP
epi-31
Normalized Intensity
Chemical Shift (ppm)
CO2H
OH
HO
MeO
13.02
11.54
10.52
8.68
8.41
8.40
8.32
8.31
7.56
7.54
7.39
7.38
7.31
7.29
7.23
7.10
7.08
7.08
7.07
6.86
6.85
6.33
6.30
6.13
6.13
6.12
6.12
6.10
6.08
5.28
5.26
5.25
5.21
5.07
5.06
4.58
4.55
4.43
4.42
4.11
4.10
4.08
4.01
3.99
3.98
3.74
3.73
3.62
3.51
3.42
3.40
3.33
3.04
3.03
2.94
2.75
2.73
2.50
2.50
2.48
2.47
2.31
2.14
2.05
2.03
2.03
1.58
1.41
1.40
1.38
1.23
1.20
1.02
0.77

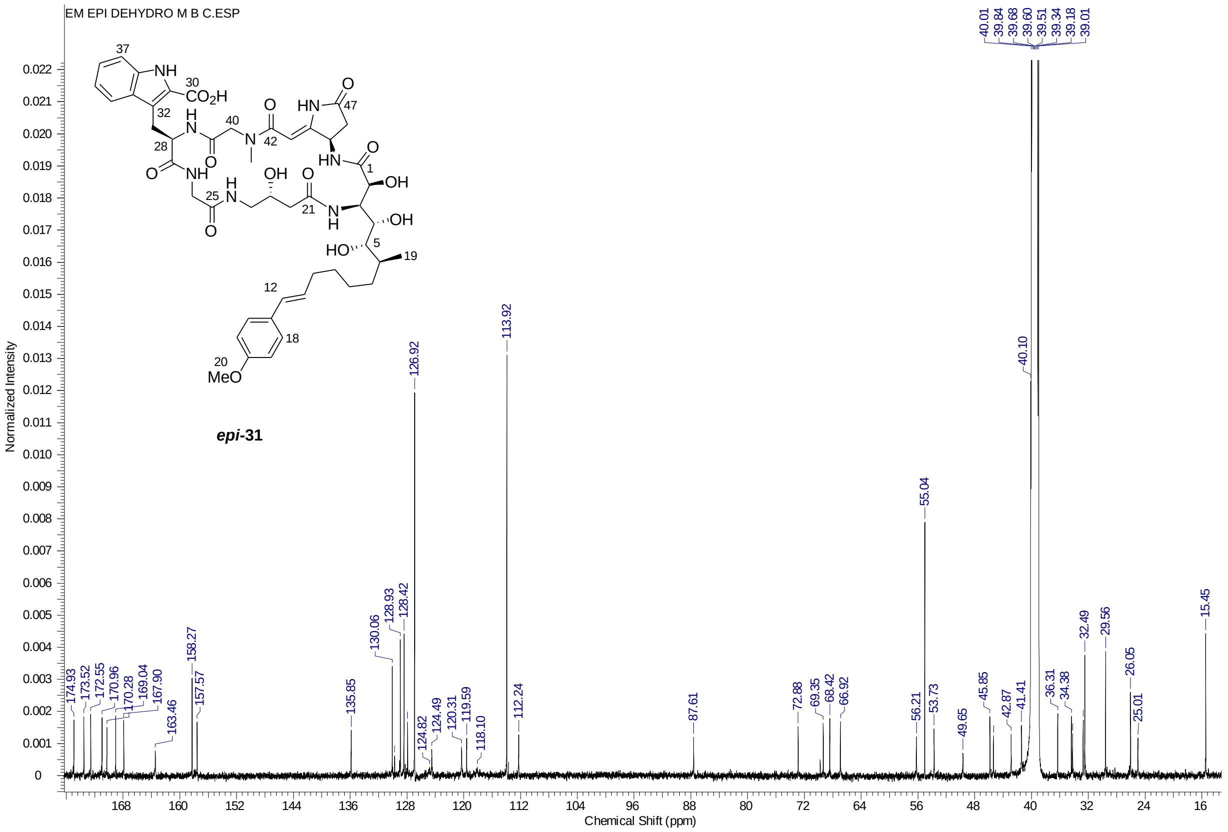
EM EPI DEHYDRO M B C.ESP
epi-31
Normalized Intensity
Chemical Shift (ppm)
174.93
173.52
172.55
170.96
170.28
169.04
167.90
163.46
158.27
157.57
135.85
130.06
128.93
128.42
126.92
124.82
124.49
120.31
119.59
118.10
113.92
112.24
87.61
72.88
69.35
68.42
66.92
56.21
55.04
53.73
49.65
45.85
42.87
41.41
40.10
40.01
39.84
39.68
39.60
39.51
39.34
39.18
39.01
36.31
34.38
32.49
29.56
26.05
25.01
15.45

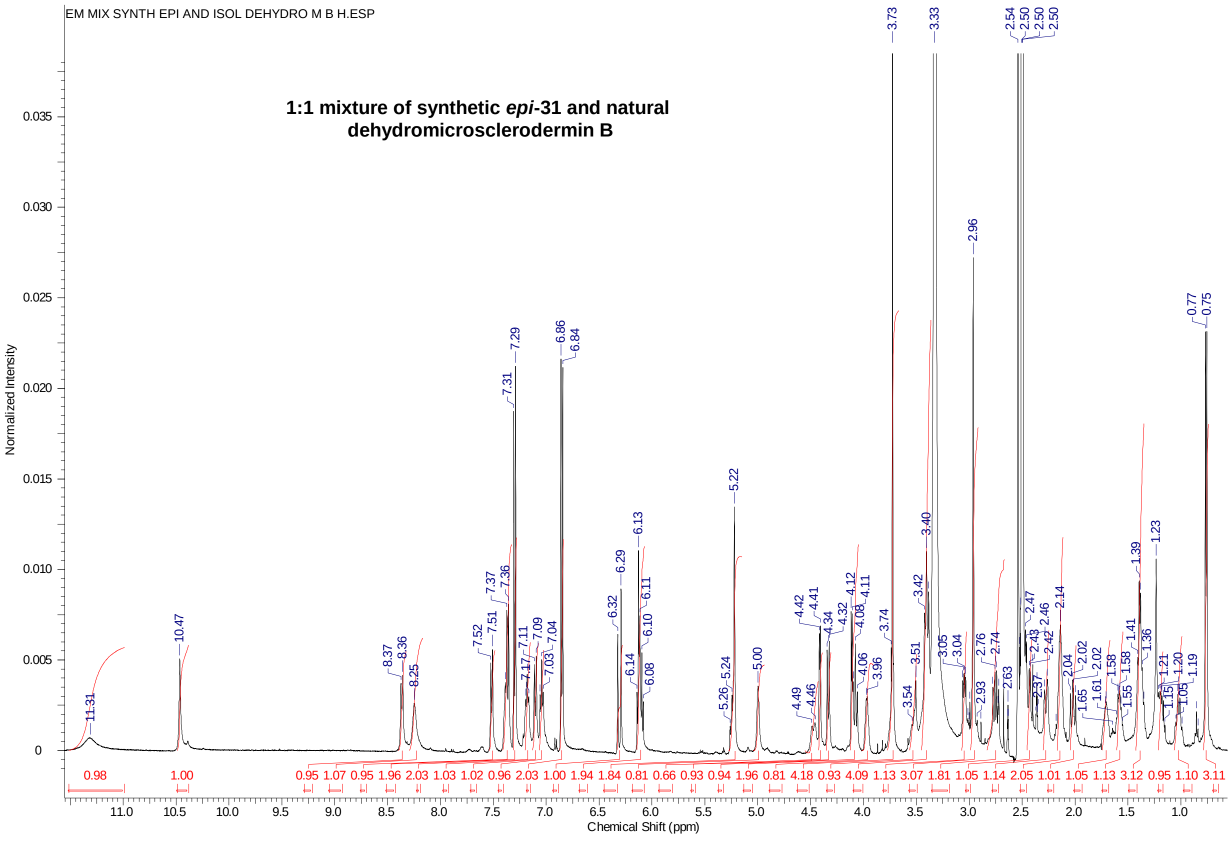
EM MIX SYNTH EPI AND ISOL DEHYDRO M B H.ESP
1:1 mixture of synthetic epi-31 and natural dehydromicrosclerodermin B
Normalized Intensity
Chemical Shift (ppm)
11.31
10.47
8.37
8.36
8.25
7.52
7.51
7.37
7.36
7.31
7.29
7.17
7.11
7.09
7.04
7.03
6.86
6.84
6.32
6.29
6.14
6.13
6.11
6.10
6.08
5.26
5.24
5.22
5.00
4.49
4.46
4.42
4.41
4.34
4.32
4.12
4.11
4.08
4.06
3.96
3.74
3.73
3.54
3.51
3.42
3.40
3.33
3.05
3.04
2.96
2.93
2.76
2.74
2.63
2.54
2.50
2.50
2.50
2.47
2.46
2.43
2.42
2.37
2.14
2.04
2.02
2.02
1.65
1.61
1.58
1.58
1.55
1.41
1.39
1.36
1.23
1.21
1.20
1.19
1.15
1.05
0.77
0.75

Comparison of ^{13}C NMR of synthetic *epi*-31 with natural dehydromicrosclerodermin B

Note: C30, C31 and C32 were not identified in the spectrum of natural dehydromicrosclerodermin B.

Carbon №	$\delta_{C(\text{synthetic } epi\text{-}31)}$	$\delta_{C(\text{natural})}$	$\Delta\delta_c$, ppm
1	173.5	173.6	-0.1
2	69.4	69.4	0
3	53.7	53.8	-0.1
4	68.4	68.4	0
5	72.9	73	-0.1
6	34.4	34.4	0
7	32.7	32.8	-0.1
8	26.1	26.1	0
9	29.6	29.6	0
10	32.5	32.6	-0.1
11	128.4	128.5	-0.1
12	128.9	129	-0.1
13	130.1	130.1	0
14	126.9	127	-0.1
15	113.9	114	-0.1
16	158.3	158.3	0
17	113.9	114	-0.1
18	126.9	127	-0.1
19	15.5	15.5	0
20	55	55.1	-0.1
21	172.6	172.6	0
22	41.4	41.4	0
23	66.9	66.9	0
24	45.4	45.4	0
25	169	169.1	-0.1
26	42.9	42.9	0
27	171	171.1	-0.1
28	56.2	56.5	-0.3
29	25	25.1	-0.1
33	127.9	128	-0.1
34	120.3	120.2	0.1
35	119.6	119.5	0.1
36	124.5	124.3	0.2
37	112.2	112.3	-0.1
38	135.9	135.7	0.2
39	170.3	170.5	-0.2
40	49.7	50	-0.3
41	36.3	36.5	-0.2
42	167.9	168	-0.1
43	87.6	87.7	-0.1
44	157.6	157.6	0
45	45.9	45.9	0
46	34.2	34.2	0
47	174.9	175	-0.1

HPLC traces of (A) synthetic *epi*-31, (B) natural dehydromicrosclerodermin B and (C) a 1:1 mixture.

HPLC conditions: Phenomenex Jupiter 4u Proteo 90A 250 x 4.60 mm 4 micron column at 1.0 mL/min with UV detection 254 nm: 2 mins – 5% CH_3CN in 0.05% aqueous TFA, linear increase; 3 mins – 42% CH_3CN in 0.05% aqueous TFA, isocratic; 23 mins – 42% CH_3CN in 0.05% aqueous TFA, linear increase; 24 mins – 95% CH_3CN in 0.05% aqueous TFA, isocratic.

A.

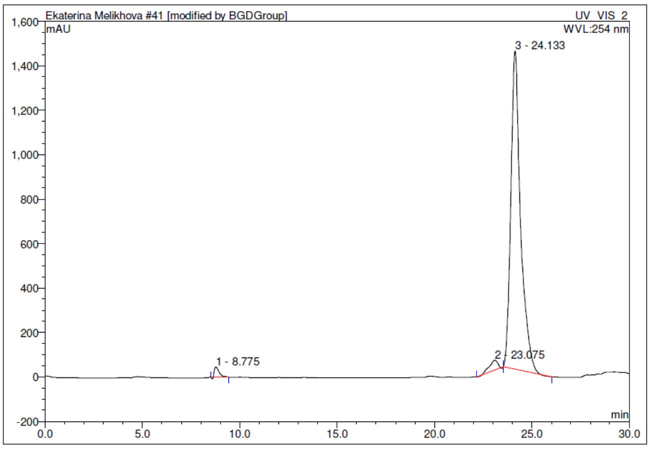

B.

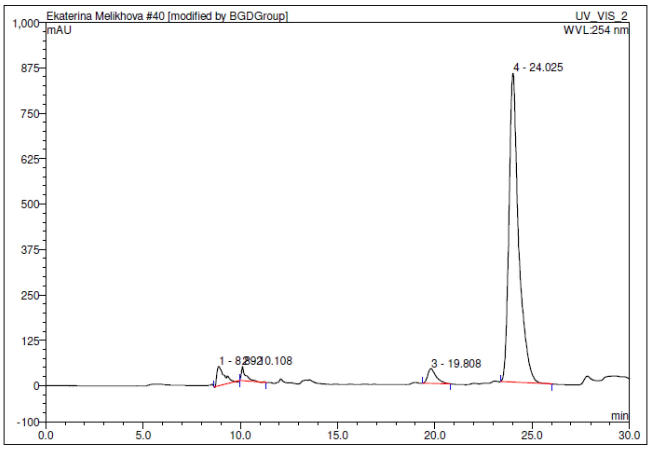

C.

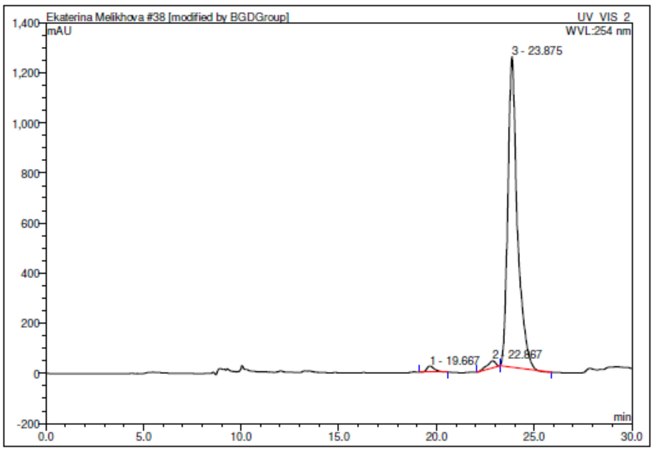

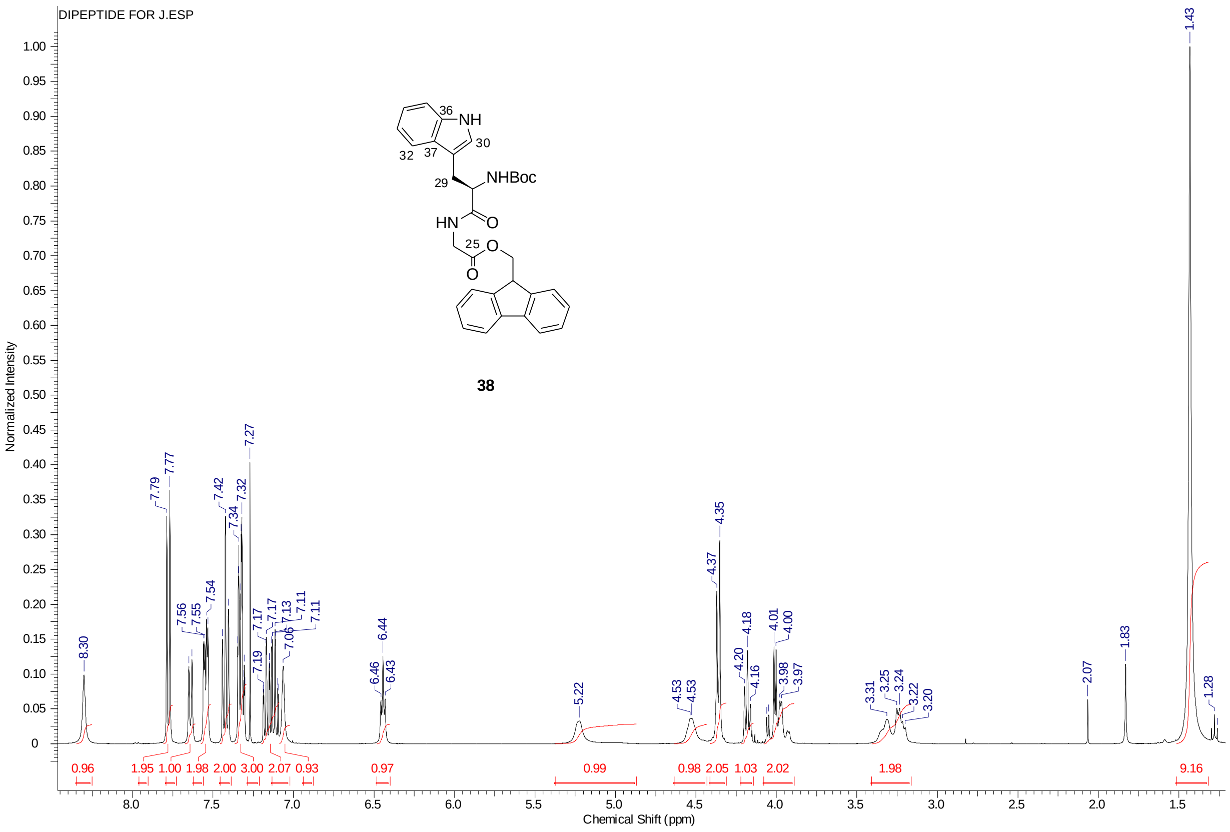
DIPEPTIDE FOR J.ESP
Normalized Intensity
Chemical Shift (ppm)
NH
NHBoc
HN
O
38

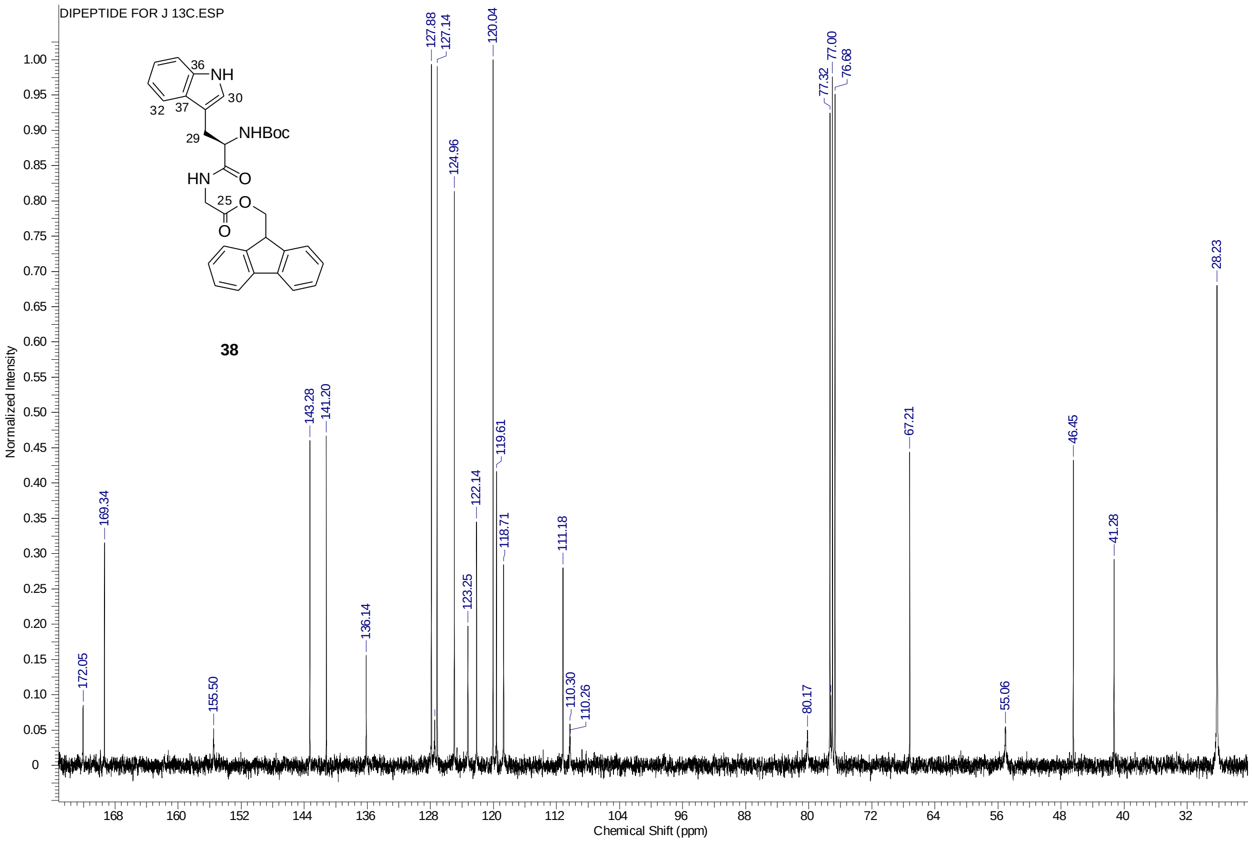
DIPEPTIDE FOR J 13C.ESP
Normalized Intensity
Chemical Shift (ppm)
NH
NHBoc
HN
O
38

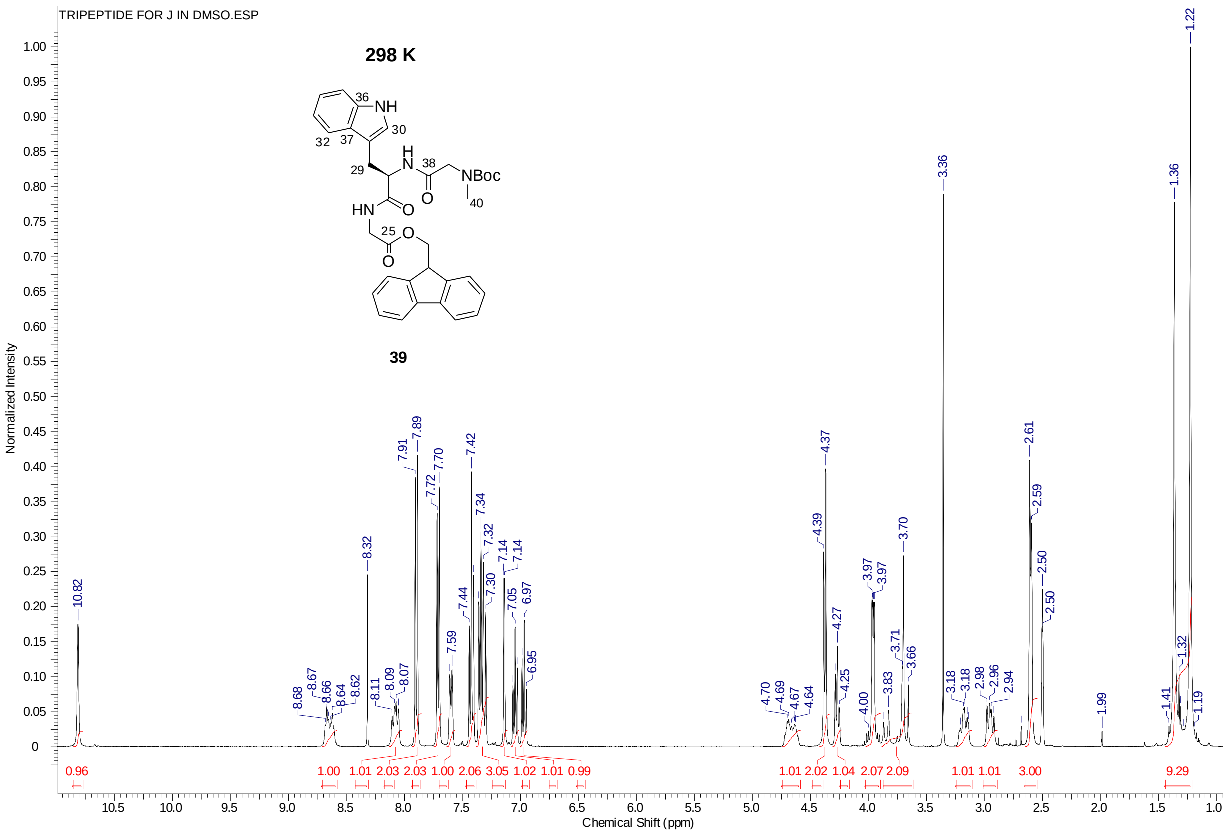
TRIPEPTIDE FOR J IN DMSO.ESP
298 K
39
Normalized Intensity
Chemical Shift (ppm)

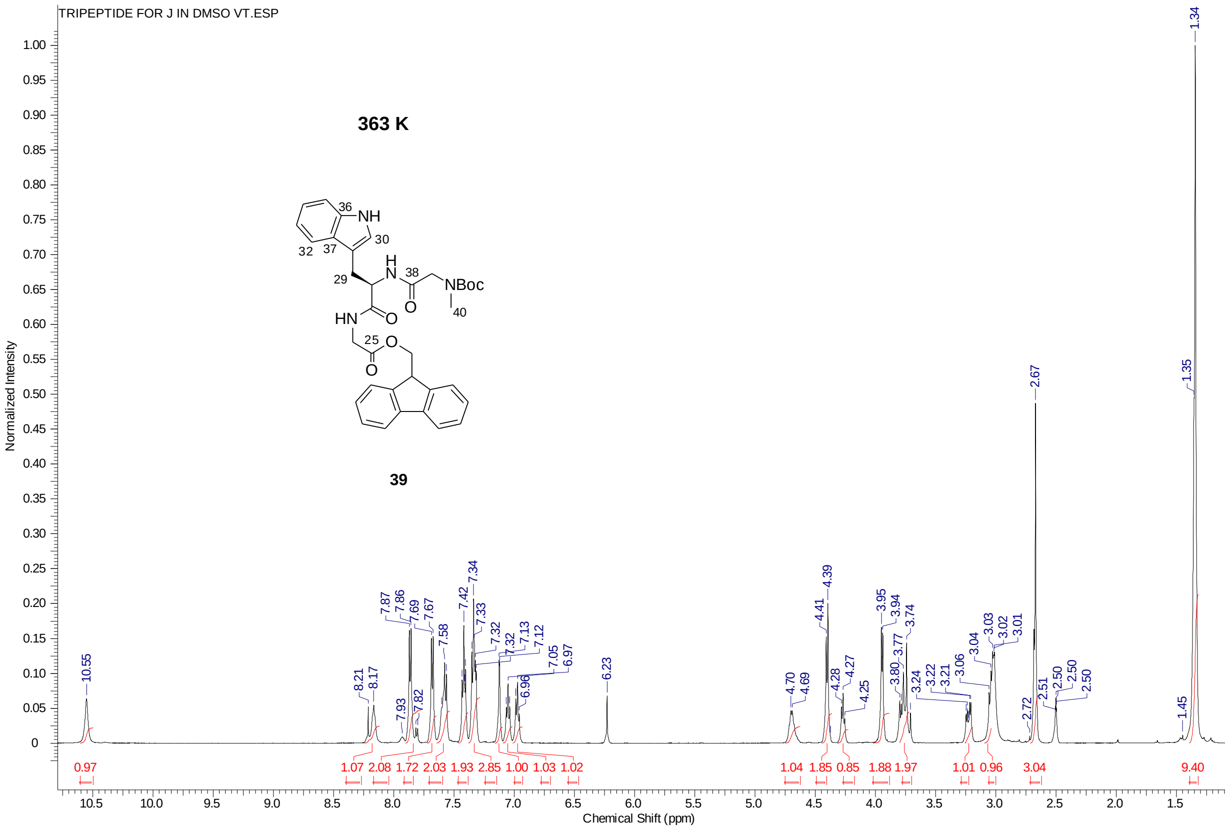
TRIPEPTIDE FOR J IN DMSO VT.ESP
363 K
39
Normalized Intensity
Chemical Shift (ppm)

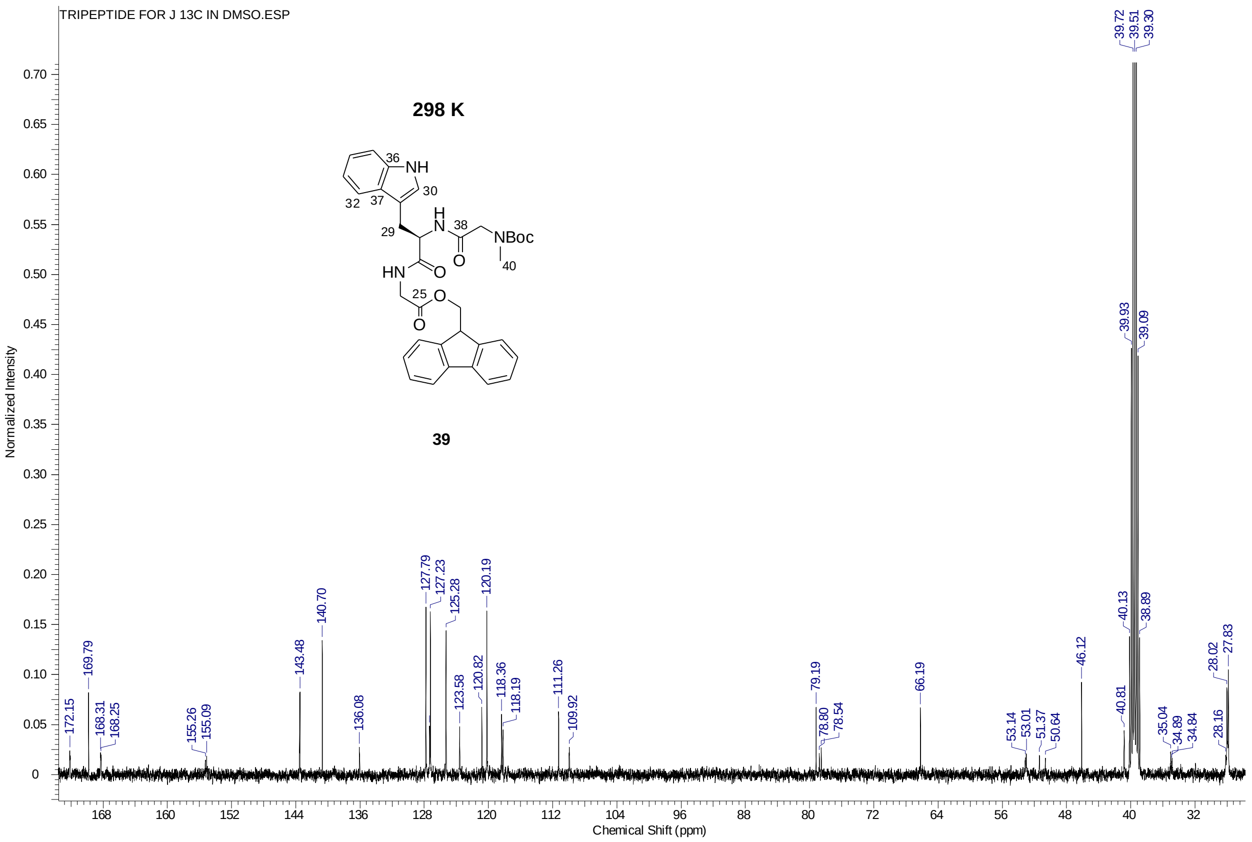
TRIPEPTIDE FOR J 13C IN DMSO.ESP
298 K
39
Normalized Intensity
Chemical Shift (ppm)

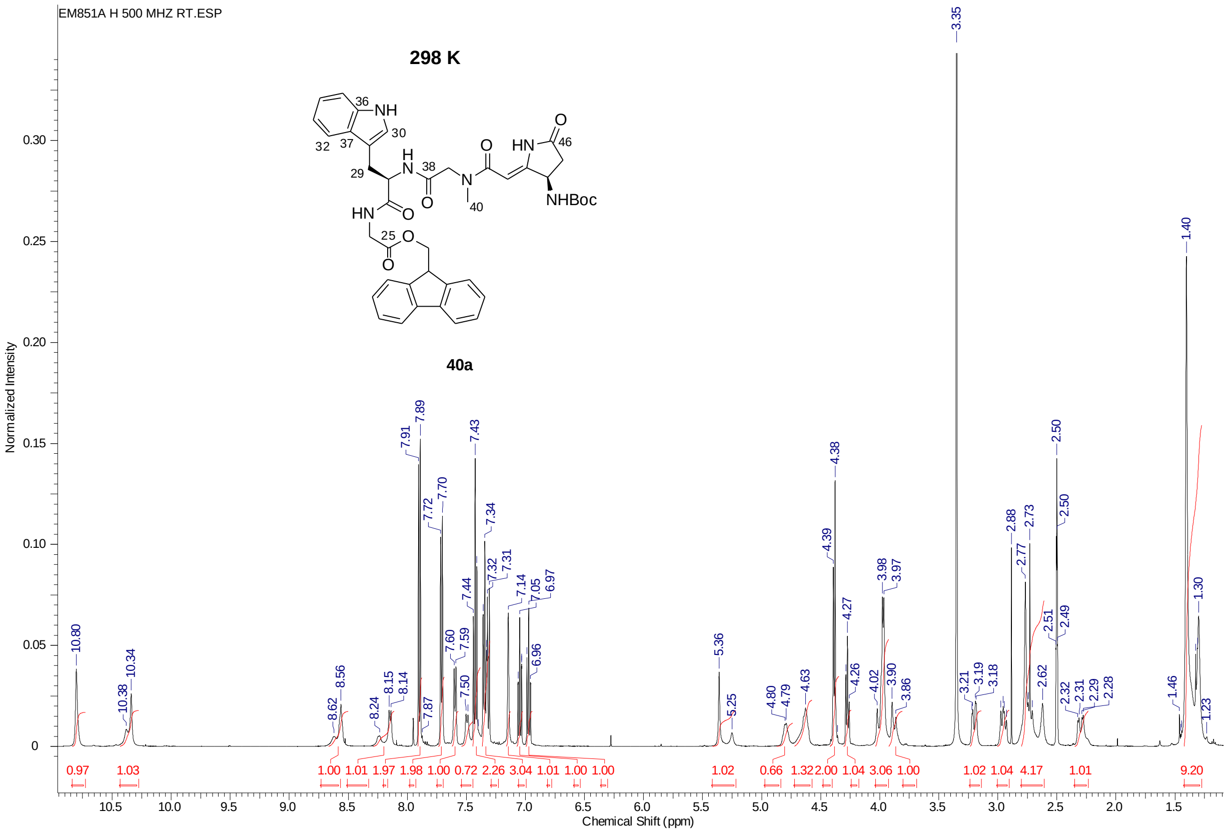
EM851A H 500 MHZ RT.ESP
298 K
40a
Normalized Intensity
Chemical Shift (ppm)

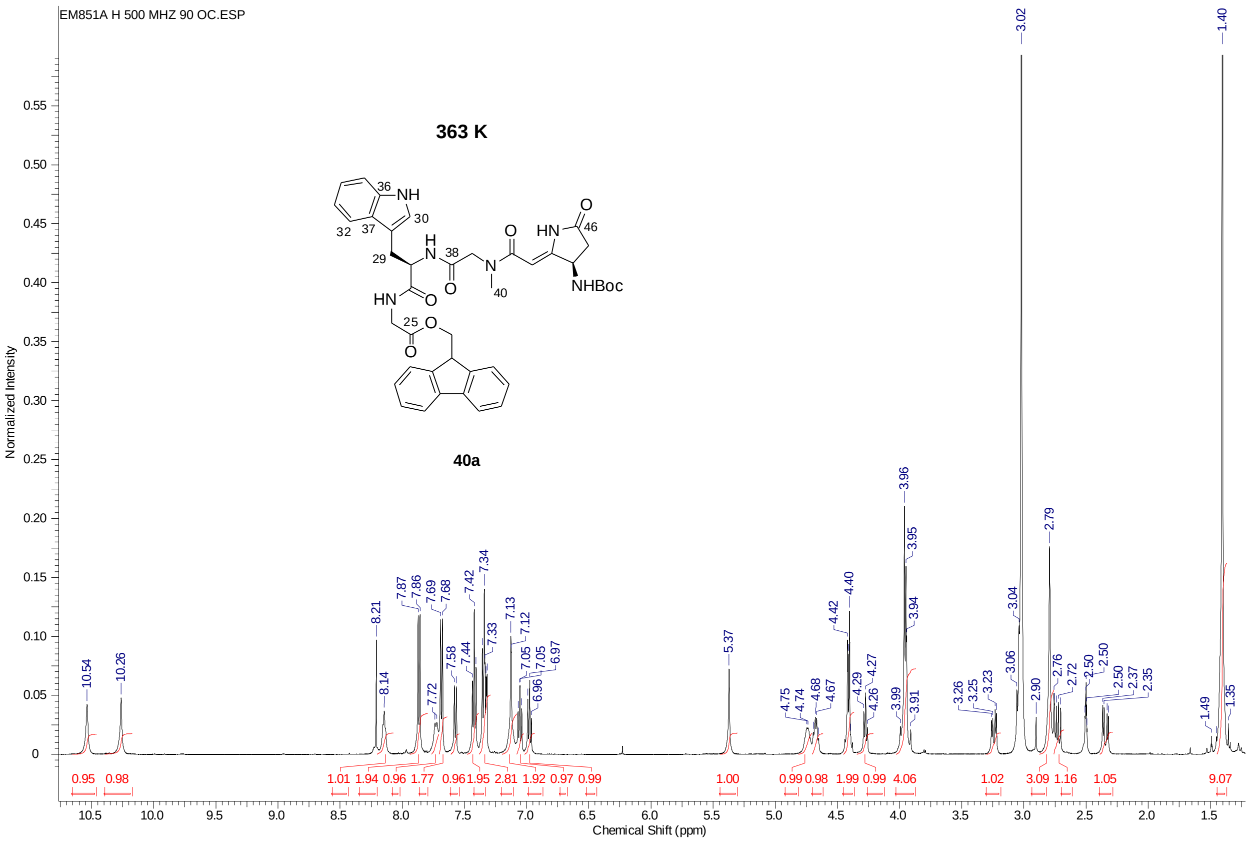
EM851A H 500 MHZ 90 OC.ESP
363 K
40a
Normalized Intensity
Chemical Shift (ppm)
10.54
10.26
8.21
8.14
7.87
7.86
7.69
7.68
7.72
7.58
7.44
7.42
7.34
7.33
7.13
7.12
7.05
7.05
6.97
6.96
5.37
4.75
4.74
4.68
4.67
4.42
4.40
4.29
4.27
4.26
3.99
3.96
3.95
3.94
3.91
3.26
3.25
3.23
3.06
3.04
3.02
2.90
2.79
2.76
2.72
2.50
2.50
2.50
2.37
2.35
1.49
1.40
1.35
0.95
0.98
1.01
1.94
0.96
1.77
0.96
1.95
2.81
1.92
0.97
0.99
1.00
0.99
0.98
1.99
0.99
4.06
1.02
3.09
1.16
1.05
9.07

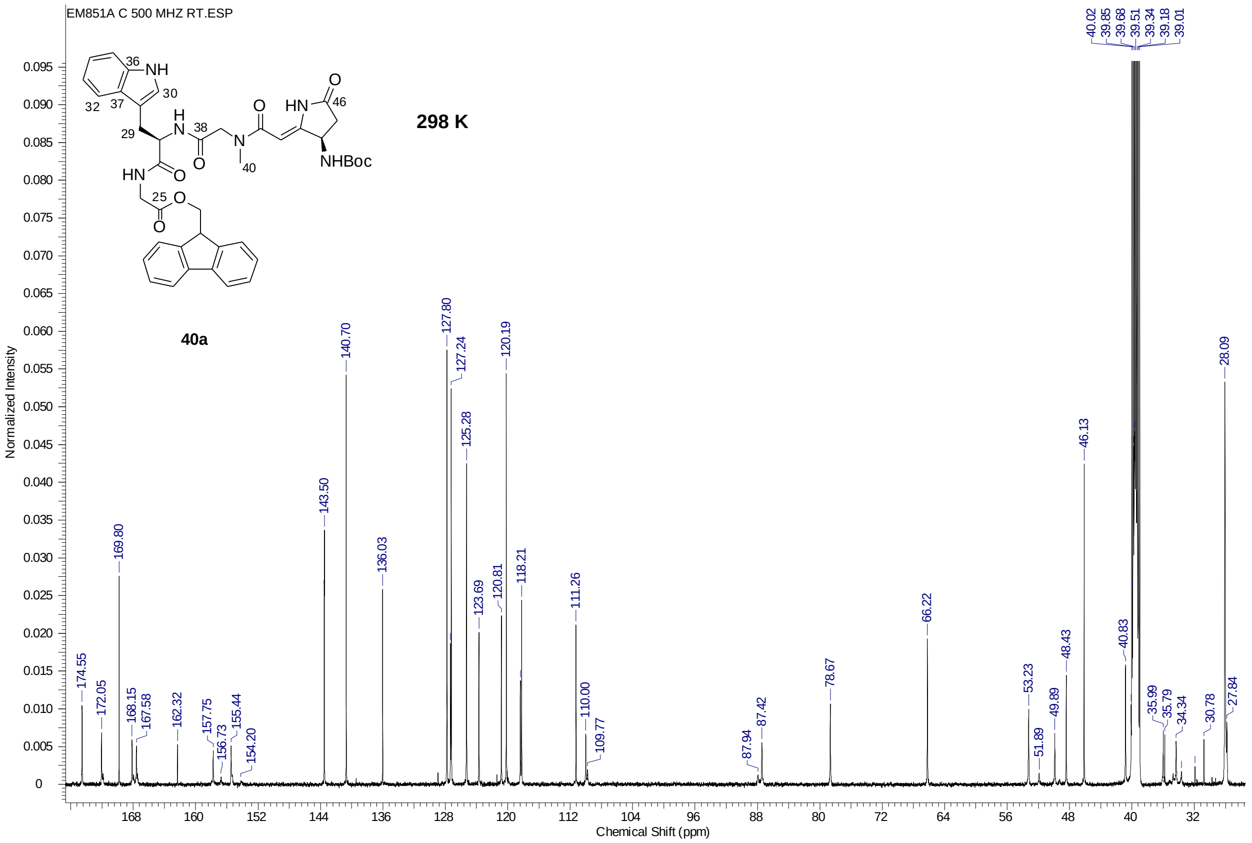
EM851A C 500 MHZ RT.ESP
298 K
40a
Normalized Intensity
Chemical Shift (ppm)
174.55
172.05
169.80
168.15
167.58
162.32
157.75
156.73
155.44
154.20
143.50
140.70
136.03
127.80
127.24
125.28
123.69
120.81
120.19
118.21
111.26
110.00
109.77
87.94
87.42
78.67
66.22
53.23
51.89
49.89
48.43
46.13
40.83
40.02
39.85
39.68
39.51
39.34
39.18
39.01
35.99
35.79
34.34
30.78
28.09
27.84

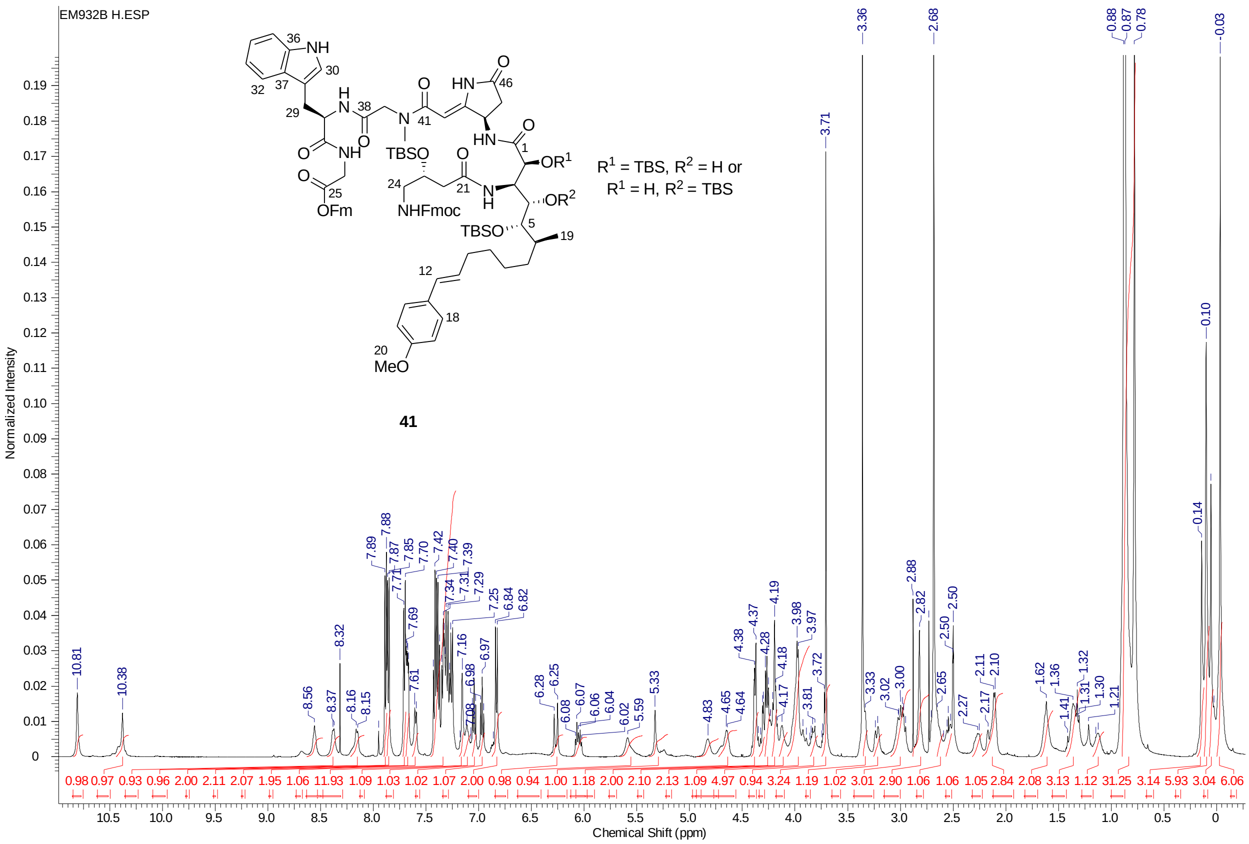
EM932B H.ESP
R¹ = TBS, R² = H or
R¹ = H, R² = TBS
41
Normalized Intensity
Chemical Shift (ppm)

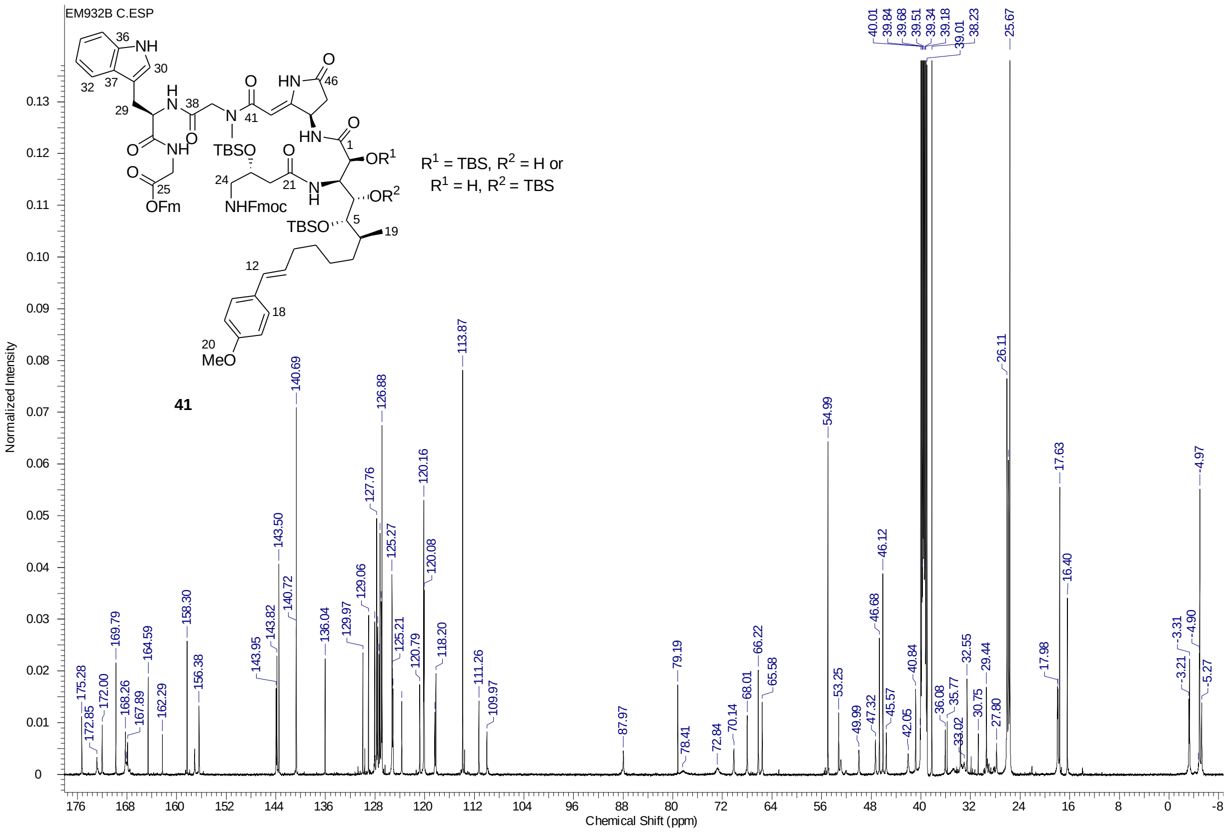
EM932B C.ESP
R¹ = TBS, R² = H or
R¹ = H, R² = TBS
41
Normalized Intensity
Chemical Shift (ppm)

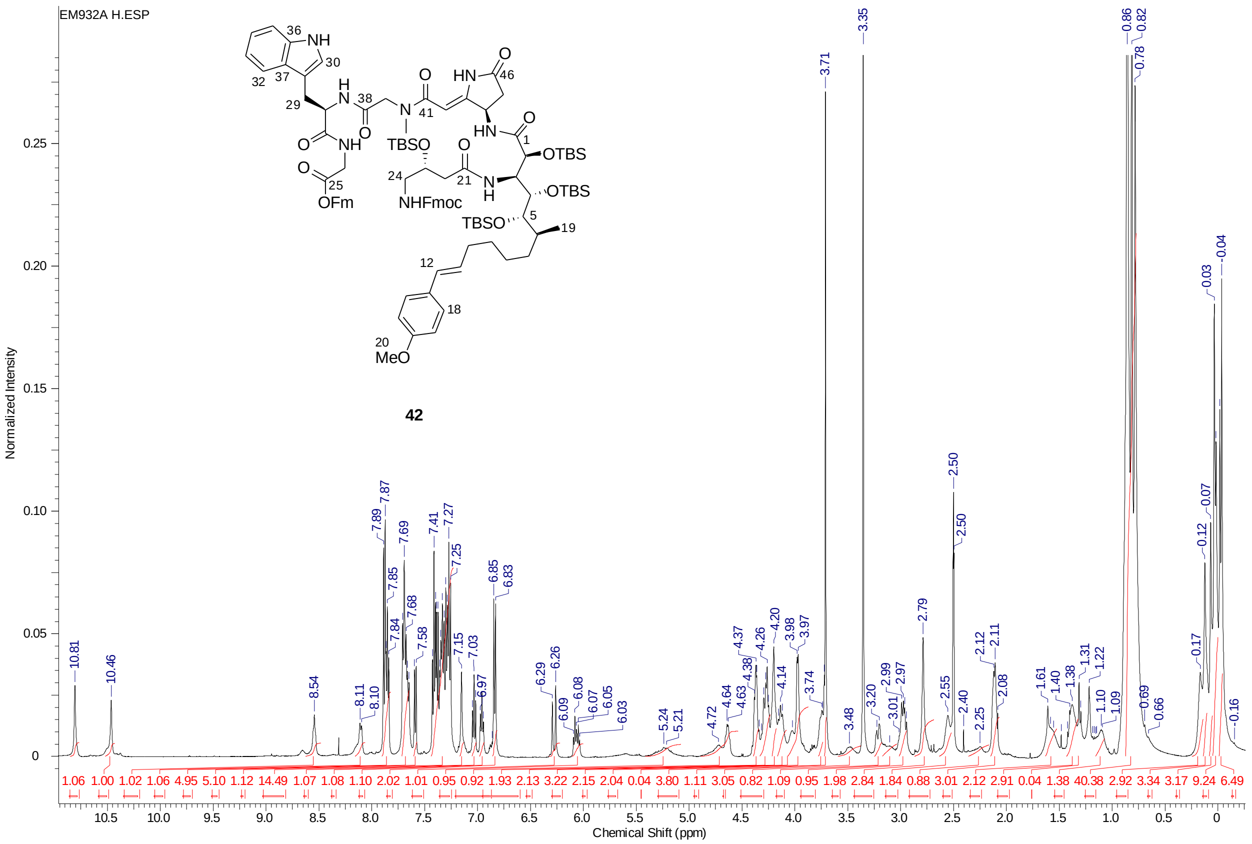
EM932A H.ESP
Normalized Intensity
Chemical Shift (ppm)
42

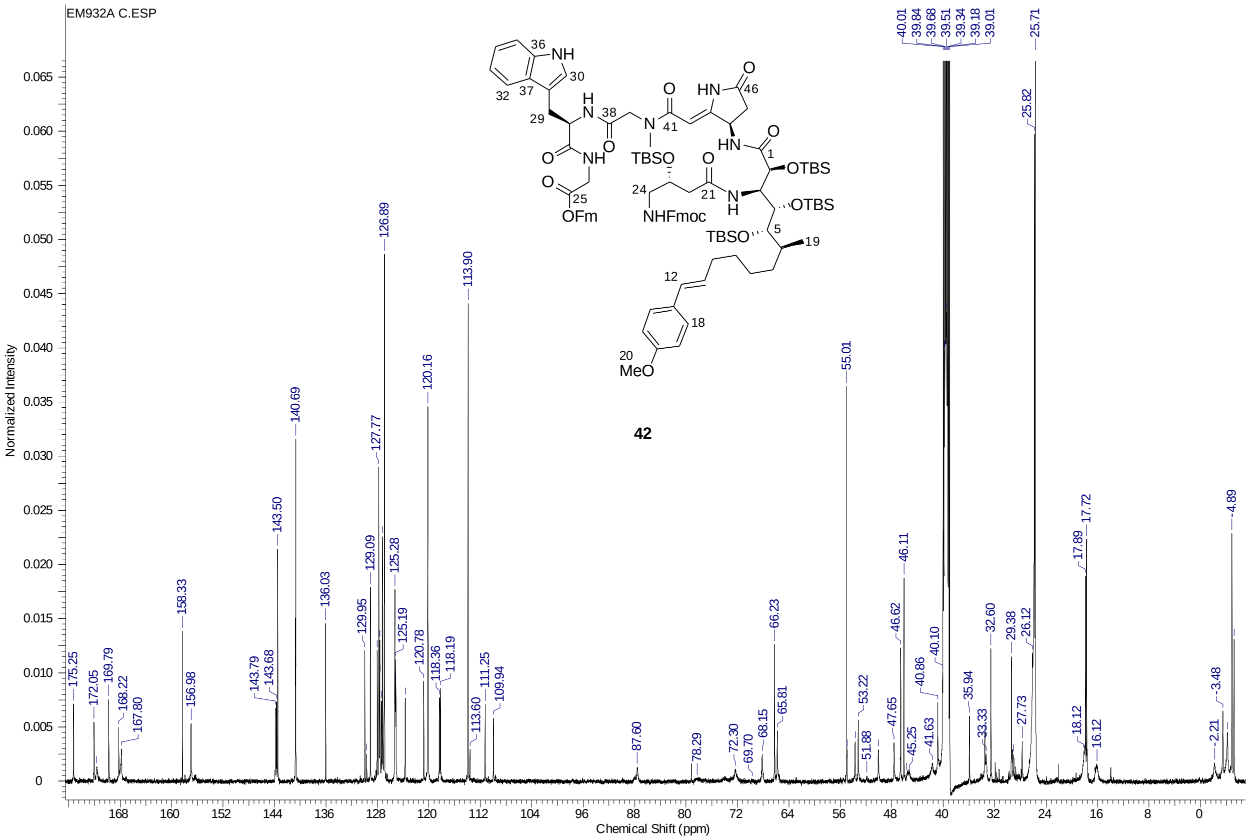
EM932A C.ESP
Normalized Intensity
Chemical Shift (ppm)
42

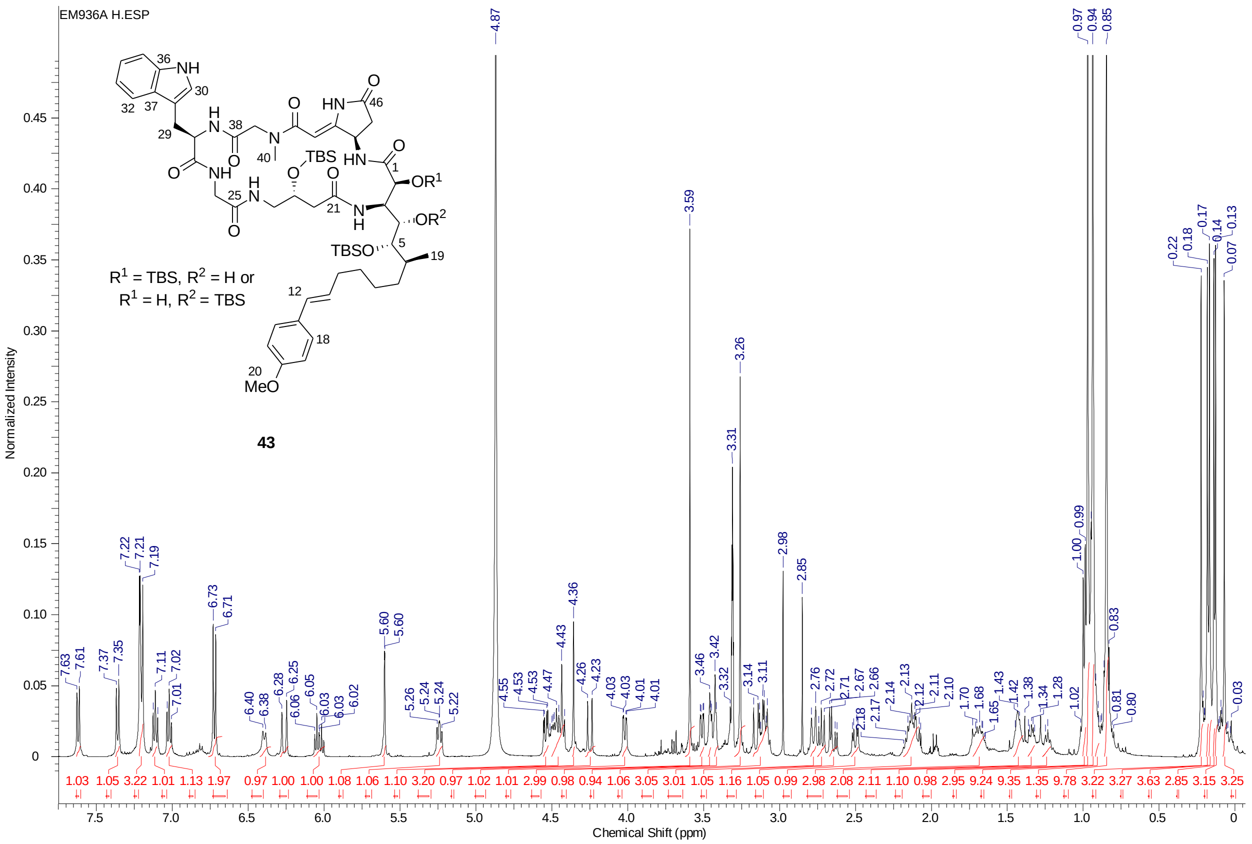
EM936A H.ESP
Normalized Intensity
Chemical Shift (ppm)
R1 = TBS, R2 = H or
R1 = H, R2 = TBS
43

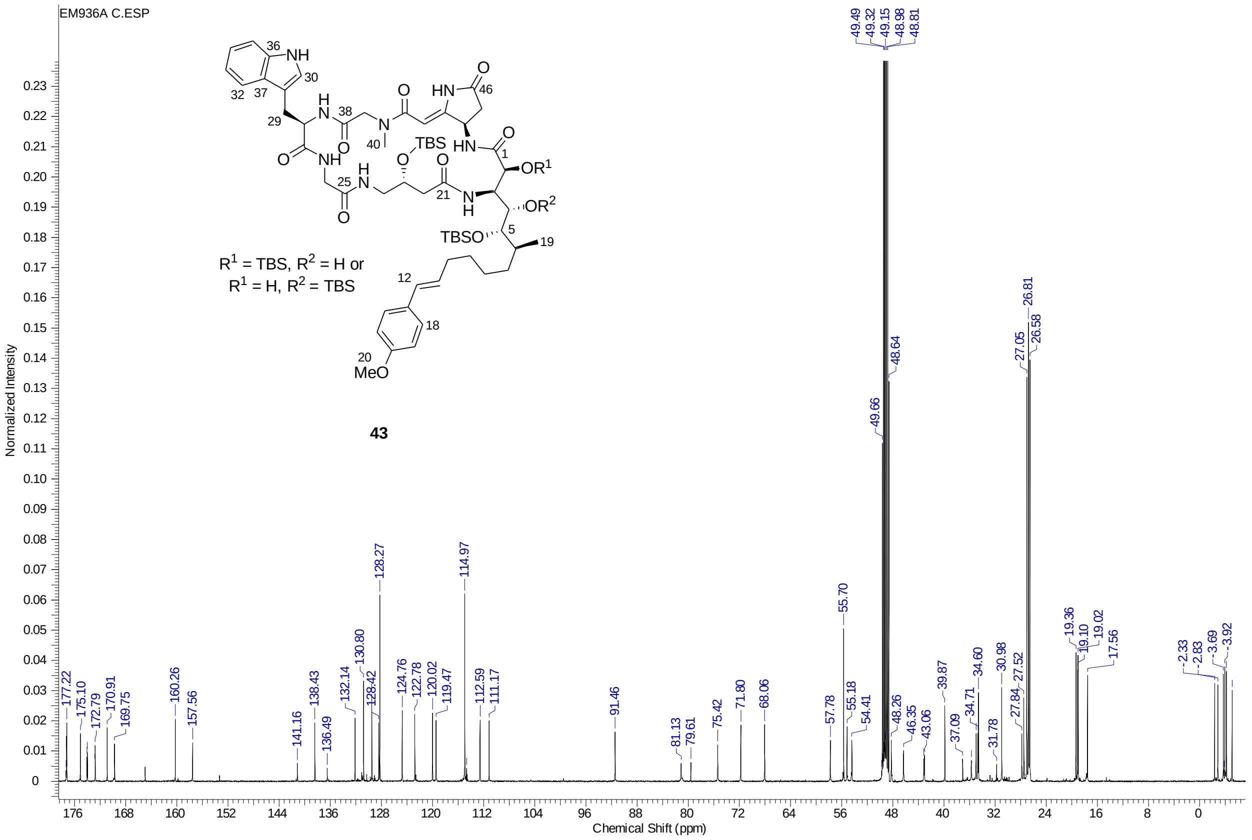
EM936A C.ESP
Normalized Intensity
Chemical Shift (ppm)
R1 = TBS, R2 = H or
R1 = H, R2 = TBS
43

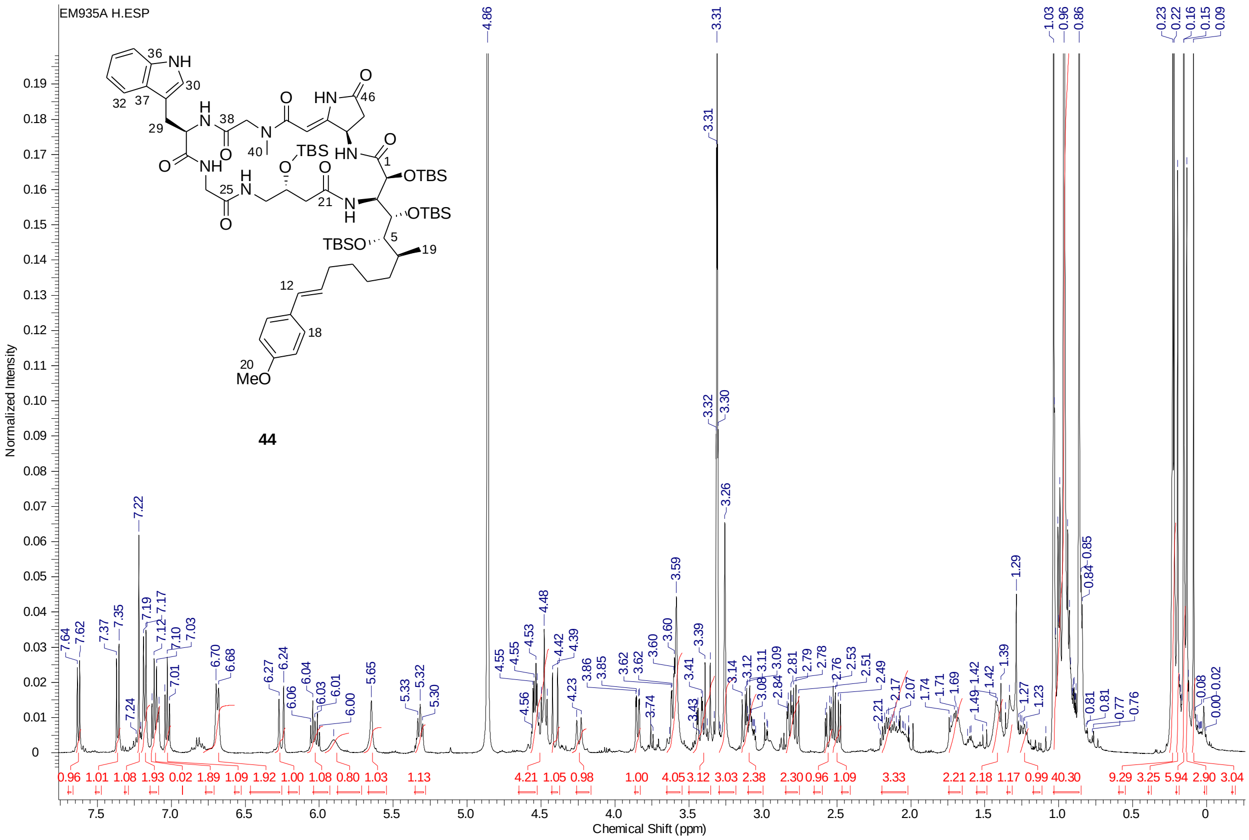
EM935A H.ESP
Normalized Intensity
Chemical Shift (ppm)
OTBS
OTBS
TBSO
TBS
MeO
HN
NH
44

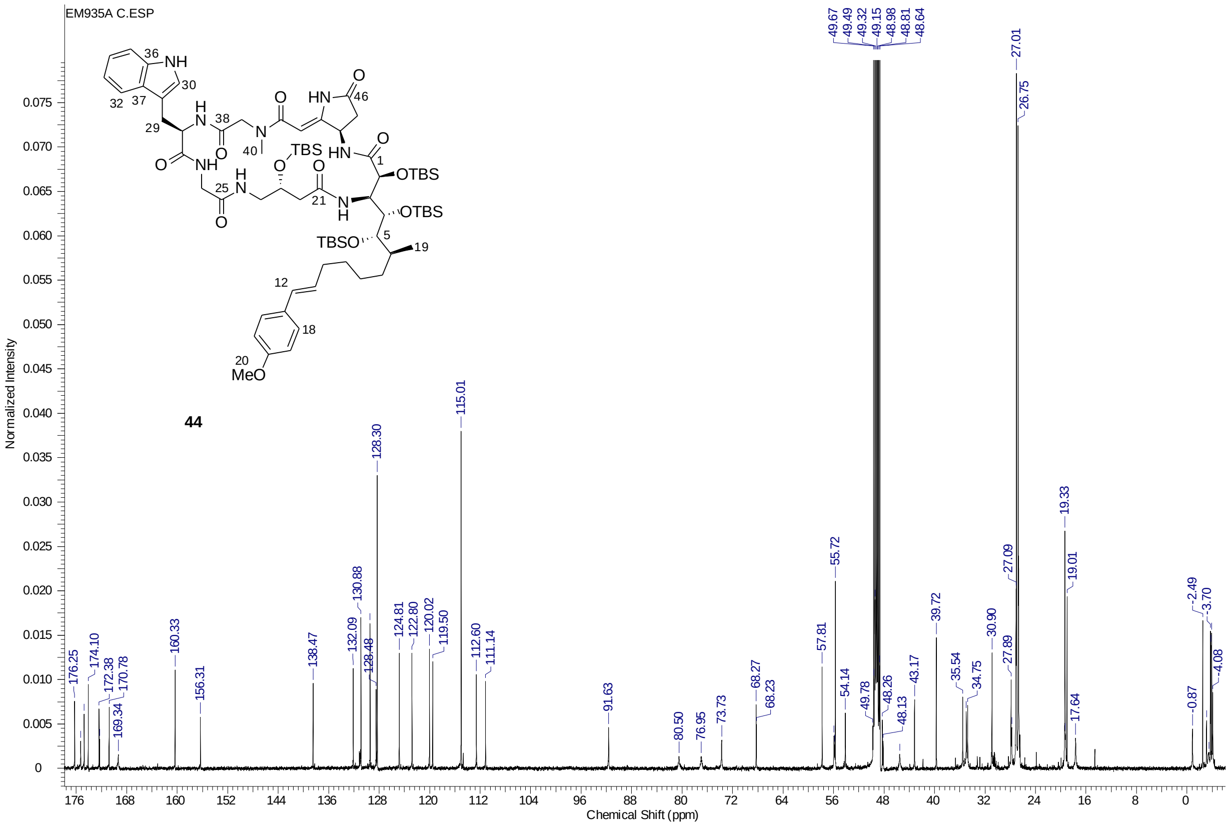
EM935A C.ESP
Normalized Intensity
Chemical Shift (ppm)
OTBS
OTBS
TBSO
TBS
MeO
HN
NH
44

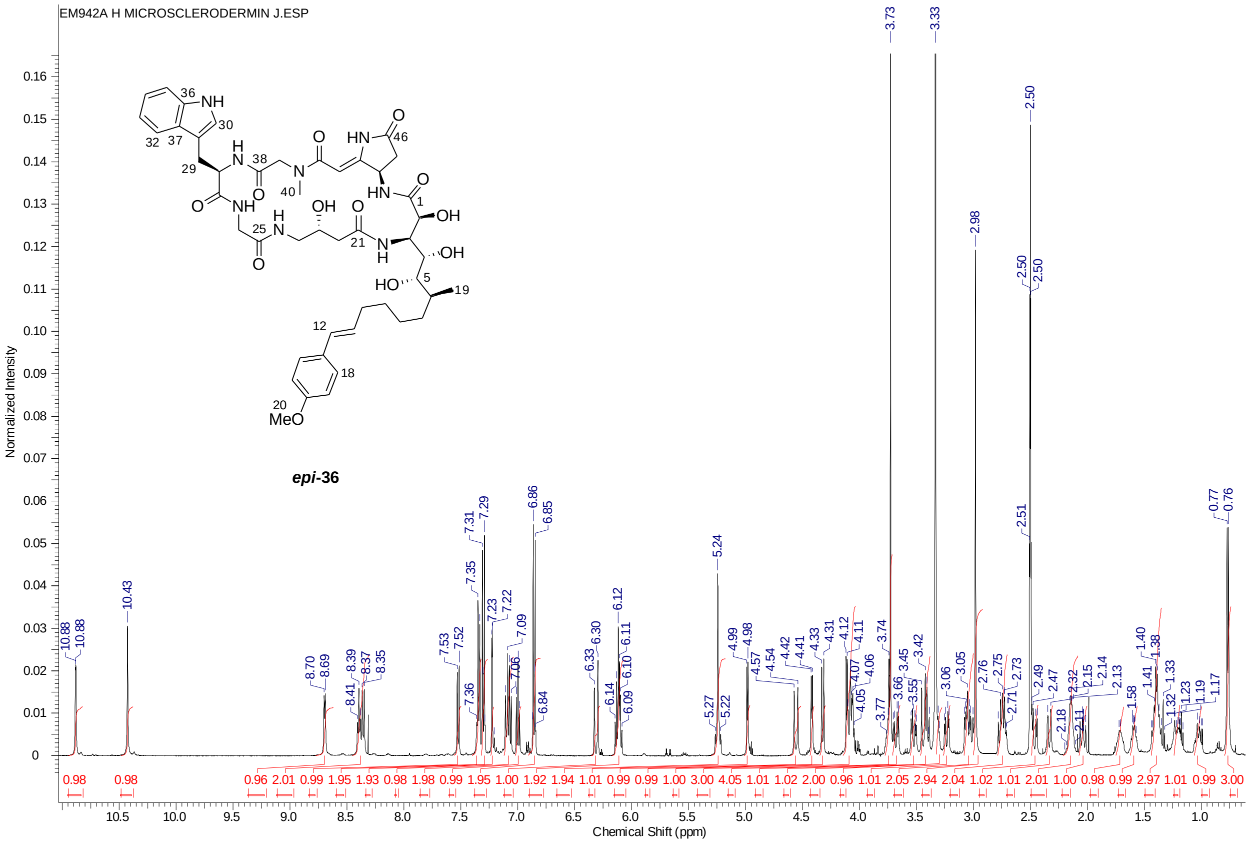
EM942A H MICROSCLERODERMIN J.ESP
Normalized Intensity
Chemical Shift (ppm)
epi-36

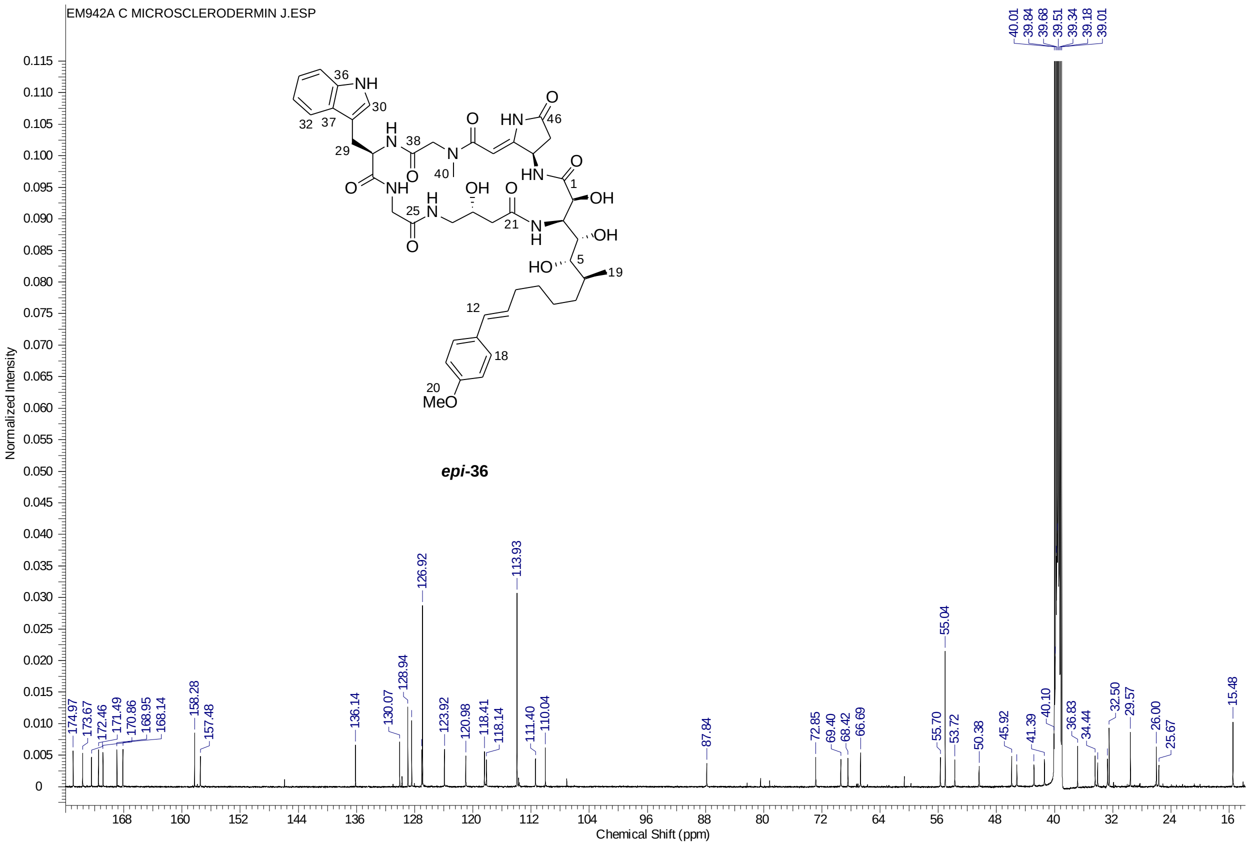
EM942A C MICROSCLERODERMIN J.ESP
Normalized Intensity
Chemical Shift (ppm)
epi-36

^{13}C NMR spectrum of natural microsclerodermin J (provided by Li)

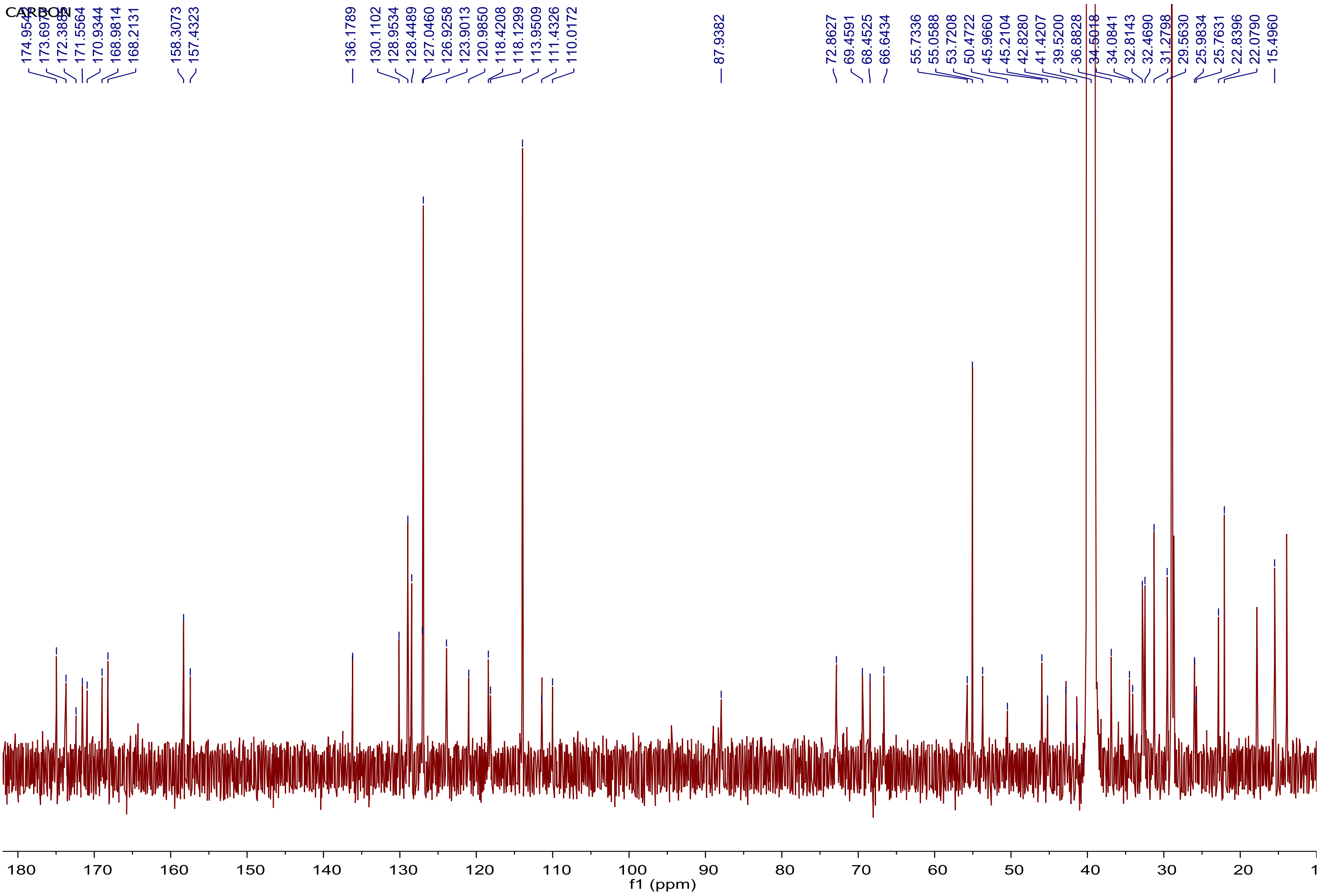

Comparison of ^{13}C NMR of synthetic *epi*-36 with ^{13}C NMR of natural microsclerodermin J (provided by Li)

Carbon №	$\delta_{C(synthetic\ epi\text{-}36)}$	$\delta_{C(natural)}$	$\Delta\delta_c$, ppm
1	173.7	173.7	0
2	69.4	69.5	-0.1
3	53.7	53.7	0
4	68.4	68.5	-0.1
5	72.9	72.9	0
6	34.4	34.5	-0.1
7	32.7	32.8	-0.1
8	26	26	0
9	29.6	29.6	0
10	32.5	32.5	0
11	128.4	128.5	-0.1
12	128.9	129	-0.1
13	130.1	130.1	0
14	126.9	126.9	0
15	113.9	114	-0.1
16	158.3	158.3	0
17	113.9	114	-0.1
18	126.9	126.9	0
19	15.5	15.5	0
20	55	55.1	-0.1
21	172.5	172.4	0.1
22	41.4	41.4	0
23	66.7	66.6	0.1
24	45.2	45.2	0
25	169	169	0
26	42.8	42.8	0
27	171.5	171.6	-0.1
28	55.7	55.7	0
29	25.7	25.8	-0.1
30	123.9	123.9	0
31	110	110	0
32	118.1	118.1	0
33	118.4	118.4	0
34	121	121	0
35	111.4	111.4	0
36	136.1	136.2	-0.1
37	127	127.1	-0.1
38	170.9	170.9	0
39	50.4	50.5	-0.1
40	36.8	36.9	-0.1
41	168.1	168.2	-0.1
42	87.8	87.9	-0.1
43	157.5	157.4	0.1
44	45.9	46	-0.1
45	34.1	34.1	0
46	175	175	0

Inaccuracies in the published ^{13}C NMR data for natural microsclerodermin J

Carbon №	δ_C published (incorrect),[6] ppm	δ_C original (correct), ppm
1	174.1	173.7
2	69.4	69.5
4	68.4	68.5
5	72.8	72.9
6	34	34.5
8	25.9	26
9	29.5	29.6
11	128.4	128.5
12	128.9	129
15	113.9	114
17	113.9	114
19	15.9	15.5
23	67.1	66.6
24	45.7	45.2
25	168.9	169
27	171.5	171.6
29	25.7	25.8
37	127.5	127.1
39	50	50.5
41	168.1	168.2
44	45.9	46
45	34.5	34.1
46	174.9	175
